# Proceedings of ‘FETP-ICON 2020’ Conference: Chennai, India. 3-5 March 2020

**DOI:** 10.1186/s12919-021-00223-6

**Published:** 2021-08-19

## A1 Need to involve traditional Indian medical systems - AYUSH, for Tuberculosis control in India

### Rakesh Roshan Bhardwaj^1^, Ravinder Kumar^2^, Rajesh Sood^1^, Beena Thomas^3^, Omesh K Bharti^1^, Rajesh Guleri^1^, Baria^1^, Sunder Sharma^4^

#### ^1^Heath and Family Welfare Department, Shimla, Himachal Pradesh, India; ^2^WHO Consultant National Tuberculosis Elimination Program (NTEP), Shimla, Himachal Pradesh, India; ^3^National Institute of Research in Tuberculosis (NIRT), Chennai, Tamil Nadu, India; ^4^Directorate of Ayurveda, Shimla, Himachal Pradesh, India

##### **Correspondence:** Rakesh Roshan Bhardwaj (rakesh9342@gmail.com)

**Background**

Tuberculosis (TB) is a significant public health problem in India. Early diagnosis and complete treatment of tuberculosis is the cornerstone of prevention and control strategy [1]. Only 65% of TB cases are notified globally, and about three million TB cases were either not diagnosed, not treated, or not reported to national TB programs [1]. India accounts for about one-third of these ‘missing’ millions.

Himachal Pradesh (HP) is a north Indian hilly state with a population of 7 million. The annual risk of TB infection for HP is 257 per lakh, but the annual case notification rate was 201 per lakh in 2014 [2]. There are two parallel health systems in HP; one is the Department of Health and Family Welfare. The other is traditional Indian medical systems, known as Ayurveda, Unani, Siddha, and Homeopathy (AYUSH). The AYUSH health facilities have comprehensive coverage [3] with registered strength of 6801 AYUSH doctors and 1230 AYUSH health centers in the public sector [3]. People living in villages, hilly regions, tribal areas, and difficult areas have easy access to AYUSH. Many presumptive TB patients may seek care from AYUSH who were neither trained nor formally engaged in NTEP, and many TB patients might be missed in this process. In HP, the case detection has plateaued to 14000 against the expected 18500 cases annually [2]. Although private practitioners' involvement in improving TB detection rates has been demonstrated to be a useful step in multiple studies across India [4,5], there is limited data on the participation of AYUSH practitioners in improving TB detection. We designed an intervention to increase the referral of presumptive TB cases from AYUSH to the public health facilities implementing NTEP in Shimla and Kangra Districts of HP. We compared the quality and number of presumptive TB referrals after intervention in 2017 compared to 2015-16.

**Methods**

We surveyed before and after the intervention in the two largest districts of HP, i.e., Kangra and Shimla. Both the districts have hard to reach areas. Shimla is a predominantly hilly district while Kangra has a moderate altitude with some hard to reach areas. Study participants included all 360 public sectors AYUSH doctors and the NTEP staff in these two districts. In phase 1, the intervention was planned, and data collection, monthly review, and monitoring of key indicators were done in Phase 2.

In phase 1, We conducted a state-level advocacy workshop with key stakeholders from AYUSH, Health and Family Welfare department, and Government to create an enabling environment for the intervention (July-Dec 2016). We developed standardized training modules, operational protocols, and educational material as per NTEP guidelines and trained the AYUSH doctors. We mapped and established linkages between NTEP facilities and AYUSH facilities. We assessed the change in knowledge of participants before and after the intervention.

In phase 2, Each AYUSH doctors was supplied with IEC material, referral algorithm, sputum cups, and color-coded (White, Red & Yellow) triplicate referral forms. Monthly meetings of AYUSH doctors and study staff were conducted to collect the data. A line list of presumptive cases who did not report for sputum testing was shared with respective Senior TB supervisors (STS) of NTEP and other stakeholders. The yellow copy of the referral form was used as a referral form for sputum microscopy at linked DMC. The information on the number of referrals who reached DMCs and the red copy was collected from AYUSH doctors monthly. The information on attrition was inferred by data triangulation at each TB Unit for tracking cases who did not report for sputum microscopy. AYUSH doctors retained the White copy to maintain the record and feedback about diagnosis and treatment. The Baseline secondary data on referrals was collected for 2015-16. Ethical approval was obtained from the Institutional Ethics committee of the National Institute of Epidemiology, Chennai, India.

**Results:**

A pre-and post-sensitization questionnaire-based test was conducted for all AYUSH doctors. The evaluation was done on a scale of 1 to 20, and 68% scored 16-20. In 2015 and 2016, AYUSH doctors in the pilot districts referred 318 and 321 presumptive TB cases constituting 1% of total referrals, which increased to 1492 in 2017, including 2% of total presumptive TB cases tested under NTEP in 2017. Sputum Positivity rate of presumptive TB cases referred by AYUSH was 16% as compared to 9.4% of NTEP. The overall attrition rate of referrals reaching the DMC for the test was 56%; after tracking it reduced to 53%. In the in-depth interviews, AYUSH doctors felt empowered with the latest technical and operational guidelines of NTEP. High attrition was attributed to the far distance of DMCs and faith-healing practices. They demanded in house DMCs.

**Conclusion**

The remarkable increase in the referral of presumptive TB cases by AYUSH doctors to nearest DMCs improved TB case detection in two pilot districts of Himachal Pradesh. The Government of HP adopted this TB training-cum-logistics linkage model for AYUSH doctors statewide under “*Mukhyamantri Kshay Rog Nivaran Yojana*” (Chief Minister TB Elimination Scheme). Also, the positivity rate among those referred by the AYUSH practitioners was higher than that in the cohort referred by Allopathic practitioners in these districts, which is an indicator of better case selection by AYUSH practitioners for presumptive TB testing. We recommend that NTEP should be mainstreamed in the AYUSH. DMCs should be institutionalized in AYUSH. We also recommend AYUSH doctors and pharmacists training on identifying presumptive TB cases and early referral for sputum examination throughout the country.

References

1. Global Tuberculosis Report 2016 [Internet]. [cited 2020 Jan 7]. Available from: https://apps.who.int/medicinedocs/en/m/abstract/Js23098en/

2. TB India 2016 : Central TB Division [Internet]. [cited 2020 Jan 7]. Available from: https://tbcindia.gov.in/index1.php?lang=1&level=2&sublinkid=4569&lid=3174

3. AYURVEDA Department Himachal Pradesh [Internet]. [cited 2020 Jan 8]. Available from: http://ayurveda.hp.gov.in/Ayush.aspx

4. Dewan PK, Lal SS, Lonnroth K, Wares F, Uplekar M, Sahu S, et al. Improving tuberculosis control through public-private collaboration in India: a literature review. BMJ. 2006 Mar 11;332(7541):574–8.

5. Bhardwaj RR, Oeltmann JE, Ravichandra C, Chadda VK, Das M, Kumar AMV. Engaging private providers and Ayurvedic practitioners in Bilaspur, India: did it increase TB case detection? Public Health Action. 2016 Jun 21;6(2):154–6.

## A2 Transmission of Human Immunodeficiency Virus among long-distance truckers in Purba Medinipur district, West Bengal; India

### Dr. Dilip Kumar Biswas^1^, Dr. Rama Bhunia^2^

#### ^1^Dy Chief Medical Officer of Health-II, Purba Medinipur district, West Bengal, India; ^2^District Maternal & Child Health Officer, Howrah district, West Bengal, India

##### **Correspondence:** Dr. Dilip Kumar Biswas (dilipbiswas29@gmail.com)

**Background**

Long-distance truckers (LDTs) are at risk of getting Human Immunodeficiency Virus (HIV) infection and Sexually Transmitted Infection (STI) [1]. While LDTs are on the roads, they could not meet with their regular sexual partners. LDTs may engage with the commercial sex partner and expose to STI and HIV [2]. Limited health care services towards LDTs have been observed, and it is required to improve the sexual life of LDTs [3]. We analyzed data of LDTs visiting Haldia industrial areas between April 2017 and March 2019 Purba Medinipur district, West Bengal, India. The objectives of the study were to estimate the prevalence of HIV and STI among LDTs.

**Methods**

We conducted the study at Haldia, an Industrial area of Purba Medinipur district, West Bengal, India. There were 20 -25 large and small-scale industries. LDTs visited at Haldia industrial area for loading and unloading of goods. Health check-ups and interviews were done at the "Satellite" clinic run by "Transport Corporation of India" as a targeted intervention project (TI). Counseling and HIV testing were done by the "Whole Blood Finger Prick" test (screening test) if they consented to the test. For confirmation, clients were sent to the nearest ICTC. STI cases were diagnosed as per the presence of syndromes. Medicines for minor ailments and STI treatment were provided at clinics. ART medicines were supplied from the ART center, Tamluk District Hospital, Purba Medinipur. Data were analyzed in MS excel.

**Result**

The total footfalls of LDTs were 21065. Among them, 22% (4650) were tested for HIV between April 2017 and March 2019. LDTs of 23 different states of India visited Haldia industrial area. The maximum LDTs were from Bihar, Jharkhand, Uttar Pradesh, and West Bengal. The average stay of LDTs was ten days to 20 days with a range of one to 60 days. Among 4650 tested for HIV, 54.2% (2520/4650) were in 18-30 years with a maximum age of 70. About 76% of the LDTs were drivers, 23% were helpers. Among the total tested, 26 (0.55%) were found reactive for HIV, and the maximum cases were among the age group of 31 – 45 years (0.80%). Among positive cases, none of them used a condom during their last sexual act. HIV reactive LDTs were tagged at their nearest ART center for medicines. The majority were started on Antiretroviral therapy [92.30% (24/26)]. Among all LDTs, 2.8% (591/21065) had STI, and 75% had a urethral discharge. All STI-infected LDTs were treated with STI medicines (Table 1).

**Conclusion**

Transmission of HIV and STI among LDTs was a public health problem in Haldia industrial area, Purba Medinipur district, West Bengal. HIV reactive cases did not use a condom during the last sexual act. The lack of use indicated poor knowledge about HIV transmission. The doctors advised them to check the HIV status of their spouse. STI was also common among LDTs. We arranged STI medicines for STI-affected LDTs. However, there was a lack of facility at the “satellite clinic” to test syphilis. There were no STI kits [Point of care test kit (POC)]. It was challenging to arrange a VDRL test (test for syphilis) among LDTs at the integrated counseling & testing center (ICTC) as ICTC was situated at a distance of 3 – 10 KM away from the satellite clinic. It was challenging to motivate them to visit ICTC for the VDRL test. So, we recommended arranging the supply of a POC kit or dual kit for both HIV and syphilis at the satellite clinic. Low use of condoms might have contributed to HIV transmission among LDTs. We recommended extensive awareness generation among truckers on HIV & STI transmission and sexual health. We should promote the proper use of condoms. We may conduct further qualitative in-depth research among LDTs to understand their needs and concerns regarding HIV/STI.

**References**

1. Pandey A, Benara SK, Roy N, et al. Risk behavior, sexually transmitted infections, and HIV among long-distance truck drivers: a cross-sectional survey along national highways in India. AIDS 2008; 22: S81-S90.

2. Lippman SA, Pulerwitz J, Chinaglia M, Hubbard A, Reingold A, Díaz J. Mobility and its liminal context: exploring sexual partnering among truck drivers crossing the Southern Brazilian border. Social science & medicine. 2007 Dec 1;65(12):2464-73.

3. Sharma V, Saggurti N, Bharat S. Health care coverage among long-distance truckers in India: an evaluation based on the Tanahashi model. HIV/AIDS Research and Palliative Care: 2015:7; 83-94

**Table 1 (abstract A2).** Age-wise distribution of HIV cases and prevalence of Sexually Transmitted Infections (STI) among truckers, Purba Medinipur district, West Bengal, India

**Age group**	**Tested for HIV** **N=4650**	**Reactive**	**Positivity Rate/ 1000**
< 17 Years	6	0	0.0
18 - 30 Years	2520	11	4.4
31 - 45 Years	1742	14	8.0
46 - 60 Years	362	1	2.8
> 60 Years	20	0	0.0
Overall	4650	26	5.6
**Sexually transmitted** **Infections (STI)**	**Number with STI N = 21065**	**%**	
Urethral Discharge (UD)	442	2.1	
UD (Non-herpetic)	128	0.6	
UD (Herpetic)	21	0.1	
Total	591	2.8	

## A3 Malaria outbreak investigation in a tribal area of Pratapgarh district, Rajasthan, India, 2016

### Prasoon Sheoran^1^, Chandrakant SMoghe^1^, ThekkevilayilG Thomas^1^, Chandrashekhar S Aggarwal^1^, Sachin Sharma^2^, Samir VSodha^3^

#### ^1^National Centre for Disease Control, New Delhi, India; ^2^Medical and Health Department, Pratapgarh, Rajasthan, India; ^3^U.S. Centers for Disease Control and Prevention, New Delhi, India

##### **Correspondence:** Prasoon Sheoran (prasoonsheoran12@gmail.com)

**Background**

Globally, an estimated 4.4 million deaths were reported due to malaria in 2016 [1]. Although India aims for malaria elimination by 2030, there were over a million malaria cases, with 331 deaths reported in 2016 [2]. Malaria cases in India are collected across multiple surveillance systems, including the integrated Disease Surveillance Program(IDSP), Health Management Information System (HMIS), and National Vector Borne Disease Control Program (NVBDCP) malaria surveillance system. The 2012–17 NVBDCP Strategic Action Plan sets out the malaria prevention and control components, including surveillance, case management, integrated vector management, epidemic preparedness, and early response with supportive interventions[2]. Malaria outbreak investigations are essential to identify programmatic gaps and plan local interventions to achieve elimination goals.

On 22 September 2016, a malaria outbreak in eight tribal villages of sub-center Pal, Pratapgarh district, Rajasthan, was reported to the central IDSP unit. The eight tribal villages are located in Sita Mata Wildlife Sanctuary, which is a government-protected forest area. We investigated the malaria outbreak, determined the epidemiological and entomological characteristics, and proposed recommendations to control the epidemic and prevent additional cases.

**Methods**

To confirm the malaria outbreak, we interviewed district and sub-center health workers, reviewed outpatient medical records and malaria survey records at the Pal subcentre, and reviewed health camps reports from the tribal villages from 1 September to 31 October 2016. We defined a suspect malaria case as an acute febrile illness in a Pal sub-center resident, rural Pratapgarh district, between 1 September and 31 October 2016. We prepared thick and thin blood smears to confirm parasite presence and species identification for all suspect cases. We defined a confirmed case as an acute febrile illness with detection of either *Plasmodium falciparum* or *P. viva* or both in a peripheral blood smear is a resident of Pal sub-centre, rural Pratapgarh block.

We reviewed monthly rainfall and temperature data from 2009–2016. We conducted an environmental entomological survey by collecting mosquito larvae and adults from four tribal villages, i.e., Balaliya, Richdi, Mandkala, and Pal. We also conducted a household entomological survey to identify potential mosquito larvae breeding sites, measure mosquito density, and identify species. To measure larval density, we used a 150-cc dipper to collect mature larvae. We collected adult mosquitoes from houses using both the total-catch method via pyrethrum space spray and the hand-catch method via aspirator and torch to measure mosquito density and prevalence.

**Results**

We identified 639 malaria cases: 441 suspects (69%) and 198 confirmed (31%). The overall attack rate (AR) for confirmed malaria cases across the eight villages was 3.1% (198/6429), and the median AR was 2.3% (range: 0.1–8.9%). The median age of confirmed malaria cases was 12 years (range: 3 months–75 years); 19% (37) of confirmed cases were children aged <5 years, 37% (73) were children aged 5–14 years, and 44% (88) were persons aged ≥15 years. Males were 55% of confirmed cases. Cases started on 20 September and peaked on 29 September; there was a secondary peak on 6 October, with cases declining after that (Figure 1). The overall slide positivity rate was 31%. *P. falciparum* was detected in 89%(177/198) of cases, *P. vivax* in 7% (13/198), and infection with both *P. falciparum* and *P. vivax*(i.e., mixed infection*)* in 4% (8/198).

In August 2016, the rainfall was almost 50% more than the average August rainfall from 2009–2015 (Figure 1). In the Pal sub-centre, we identified 150 handpumps without proper drainage as potential *Anopheles* larval breeding sites. The maximum larval density of 26.2 larvae per dip was recorded from a handpump area in the Pal sub-center compared to an average of 16.1 larvae per dip (range: 7.0–26.2) across the six sites surveyed. Of the 12 environmental sites surveyed, adult *Anopheles culicifacies* mosquitoes were found in 25%, and *Anopheles* larvae were found in 100%. Of the households surveyed by the total-catch method, per-catch density was 14 mosquitoes per household of *An. culcifacies.* By the hand-catch method, adult mosquitoes were identified as 85.7%*An. culcifacies*, 9.5% *An. subpictus*, and 4.7% *An. manculatus*. Per-man-hour density was highest for *An—culicifacies* at 2.3%.

**Conclusions**

We identified an outbreak of laboratory-confirmed *P. falciparum* malaria cases among primarily adolescents and adults in the Sita Mata Wildlife Sanctuary in Pratapgarh district, Rajasthan, probably due to excessive rainfall and poor drainage in August 2016. A lack of vector control measures following increased rainfall likely contributed to the outbreak. There was no entomologist posted at the district level in Rajasthan, and there had been no residual spraying by district authorities in the previous three years because the annual parasite index was reported to be less than 2.

The district health authorities set up health camps to reach the population after identifying the initial malaria cases. A house-to-house survey was conducted to find additional cases, and treatment was provided to all suspected cases. To prevent future outbreaks, we recommended indoor residual spraying with pyrethrum spray before the monsoon season. We also recommend using larvicidal oil or organophosphate larvicide at potential breeding sites around hand pumps per NCBDCP vector control measures [3]. The other suggested interventions include distributing long-lasting insecticidal bed nets to tribal village residents, educating communities, training local health practitioners to treat suspected malaria cases per standardized protocols, and cross-training the district epidemiologists on entomology for vector management to prevent future outbreaks in this malaria-endemic district.

**References**

1. World Malaria Report 2017. World Health Organization. [https://www.who.int/malaria/publications/world-malaria-report-2017/en/]. Accessed 9 February 2021.

2. National Vector Borne Disease Control Programme. Strategic Plan for Malaria Control in India2012-2017. New Delhi: Government of India, undated. [https://nvbdcp.gov.in/Doc/Strategic-Action-Plan-Malaria-2012-17-Co.pdf] Accessed 12 March 2020.

3. National Vector Borne Disease Control Programme Vector Control Measures. [https://nvbdcp.gov.in/index4.php?lang=1&level=0&linkid=448&lid=3722] Accessed 28 March 2020.

**Fig. 1 (abstract A3).**
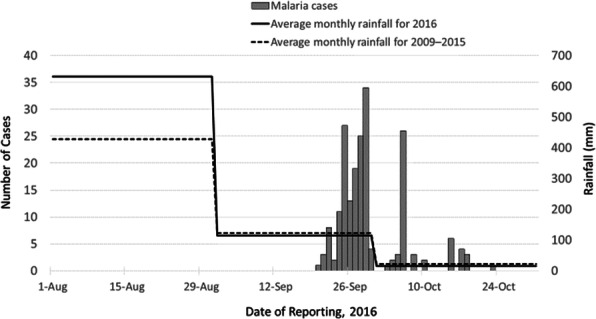
Confirmed malaria cases by reporting date to Pal sub-centre, Pratapgarh district, Rajasthan, India in September–October 2016

## A4 An outbreak investigation of acute diarrheal disease attributed to eating ice cream, in three villages of Kamareddy district, Telangana, India, 2019

### Seema Tabassum, Manikandanesan Sakthivel, Sailaja Bitrugunta

#### India Epidemic Intelligence Service Program, ICMR National Institute of Epidemiology, Chennai, India

##### **Correspondence:** Seema Tabassum (samarahmustafa786@gmail.com)

**Introduction**

Globally, the diarrhoeal disease leads to 3-5 billion cases and nearly 1.8 million deaths annually, mainly in young children, caused by contaminated food and water [1]. India’s Integrated Disease Surveillance Programme (IDSP), reported 572 acute diarrheal disease outbreaks in 2018, among them, 234 were foodborne [2]. Telangana reported 16 acute diarrheal disease outbreaks in 2018, including nine foodborne outbreaks [2]. Often foodborne outbreaks are under-reported and under-investigated [3]. On 26 March 2019, Kamareddy district surveillance unit in Telangana reported clustering of acute gastroenteritis cases involving three villages A, B and C. Epidemic Intelligence Service (EIS) officer from National Institute of Epidemiology along with the state rapid response team, initiated an investigation to describe the epidemiology of the outbreak, identify the risk factors associated with the outbreak and to implement evidence-based measures.

**Methods**

To confirm the outbreak, we compared 12-13th week IDSP reports from the last three years. We defined a case-patient as pain abdomen, or at least one episode of loose stool or vomiting in a resident of the three villages, A, B and C in Kamareddy district from 23-28 March 2019. We did active house-to-house case search in these three villages, and passive case search from the local hospital (catering affected villages) and health camp registers. Using the semi-structured questionnaire, we collected demographic information, date and time of onset of symptoms, treatment, food exposures, source of drinking water, travel history, and any history of attending village gathering. We also conducted a 1:1 age and gender-matched case-control study in the affected three villages to identify the risk factors for illness. A control was defined as a resident of villages A,B and C, without any symptom from 23-28 March 2019. We selected controls from the list available in the village household survey register of accredited social health activists (ASHA), and auxiliary nurse midwife (ANM), by simple random sampling using computer-generated random numbers. We collected information on the consumption of local street vendor ice cream. Stool samples were collected from eight suspected hospitalised case-patients (all cases had taken antibiotics) on 27 March 2019 and sent to Institute of Preventive Medicine laboratory in Hyderabad for Vibrio cholera, Salmonella and Staphylococcus culture. We tested twelve drinking water samples from household taps (four from each village collected randomly) for faecal coliforms count. We inspected the municipal drinking water supply pipelines for leakages in the affected villages. Data entry and analysis was done in MS Excel and Epi Info version 7. We analysed data by time (epidemic curve), place (attack rate and case fatality rate by village) and person (attack rate and case fatality rate by age and gender). Proportions were calculated for symptoms, hospitalisation and exposures. We selected one age and gender-matched control for every identified case-patient by simple random sampling using computer-generated random numbers. We calculated the odds ratio and 95% Confidence Interval for the exposures using Epi Info version 7. This outbreak investigation was part of the emergency public health response with all the required administrative approvals. We maintained the confidentiality of the data and did not collect any personal identifiers.

**Results**

We observed six times increase in acute diarrheal disease case-patients in the 12-13th week of IDSP reports in 2019 compared to the previous three years. We identified 181 case-patients (52% females) from 164 households in three villages between 24-27 March 2020, including three deaths (case fatality rate 1.6%). The median age of the case-patients was 14 years (range 3-72 years). The first case-patient was reported at 10 PM on 24 March 2020, the number peaked (34) on 25 March between 12-2 AM and another peak on 26 March between 10 AM-12 PM (Figure 1). Three deaths included two females (four years and 37 years) and one male (7 years) from Villages B and C. Overall attack rate was 4% (181/4427) in the three villages, with highest attack rate in Village C (6%, 125/2112), followed by Village B (4%, 45/1250), and Galipoor village (1%, 11/1065). The attack rate was highest in 0-14 age group (18%), lowest in above 50 age group (1%) but similar among males (4%) and females (4%) in the three villages. Cases reported pain abdomen, loose stools (100%), vomiting (76%), and fever (53%). Thirty-four percent (62/181) cases received treatment at local hospital (IV antibiotics, IV fluids, or oral antibiotics), 33% (59/181) at the village health camps (oral antibiotics and IV fluids), and 33% (59/181) were distributed oral antibiotics and ORS during home visits. About 10% (18/181) cases attended a religious feast on 24 March 2020 at a temple in Village C. None reported travel history outside the village in the last three days. In three villages, the drinking water sources were individual borewells 88% (160/181) and common water supply 12% (21/181). About 60% (108/181) case-patients reported eating ice cream from a local street vendor (44% on 24 March and 16% on 25 March). Case-control study shows that about 60% (108/181) of case-patients consumed ice cream (made from raw milk) compared to 10.5% (19/181) control persons (OR-46, CI: 11-186). All eight stool samples were negative for Vibrio cholera, Salmonella and Staphylococci bacterial growth. We also observed leakage in two pipelines (supplying water from community tanks) in villages B and C, 13% (23/181) consumed water from this pipeline. Two (25%) water samples from Villages B and C (Common water supply) tested positive for faecal coliforms (<2 colony units/100ml).

**Conclusion**

Our findings confirmed an acute gastroenteritis outbreak associated with ice-cream consumption from a street vendor affecting mostly children below 14 years. About one-third of the cases had to be hospitalised. Negative stool culture results may be due to antibiotic treatment before collection of samples. We recommended immediate restrictions on selling homemade ice cream by street vendors and inspection of ice-cream preparation practices by district food inspectors. We also recommended avoiding drinking water from government water supply without boiling or chlorination till leakages were repaired. Based on our recommendations and food inspector reports, the District Collector issued a circular to stop vendors from selling homemade ice cream.

**References**

1. World Health Organization. Diarrhoeal disease [Internet]. Available from: https://www.who.int/news-room/fact-sheets/detail/diarrhoeal-disease. [Accessed 2020 Aug 12]

2. Integrated Disease Surveillance Programme. Annual Report 2018 [Internet]. Available from: https://idsp.nic.in/showfile.php?lid=4134. [Accessed 2020 Aug 12]

3. World Health Organization. WHO estimates of the global burden of foodborne diseases. Geneva, Switzerland: World Health Organization; 2015. 254 p. Available from: https://www.who.int/foodsafety/publications/foodborne_disease/fergreport/en/. [Accessed 2020 Aug 12]

**Fig. 1 (abstract A4).**
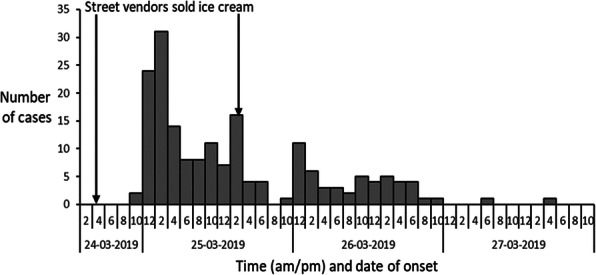
Distribution of case-patients by time and date of onset of symptoms in villages A, B and C, District Kamareddy, Telangana, 24-27 March 2019 (N=181)

## A5 Evaluation of National Leprosy Eradication Programme Surveillance System, Pandariya Block, Kawardha district, Chhattisgarh, April — June 2019

### Mohamed Azarudeen^1^, Sarwat Naqvi^2^, Amol Patil^3^, Tanzin Dikid^1^, Kanica Kaushal^3^ Rupali Roy^4^, Deepika Karotia^4^, SK Jain^1^, Sujeet Singh^1^, Anil Kumar^4^

#### ^1^National Centre for Disease Control, Delhi, India; ^2^National Leprosy Eradication Programme, Chhattisgarh & Maharashtra, India; ^3^South Asia Field Epidemiology and Technology Network (SAFETYNET); ^4^Central Leprosy Division, Ministry of Health and Family Welfare, Government of India

##### **Correspondence:** Mohamed Azarudeen (drazareis@gmail.com)

**Background**

Annually, 0.2 million new leprosy cases are reported globally [1]. WHO defines Grade 2 disability(G2D) of leprosy as visible deformity [2] and recommends the prevalence of G2D less than 1 case/ million. India contributes to 60% of the global burden of leprosy.[3]. India launched National Leprosy Eradication Programme (NLEP) to eliminate (prevalence<1 case/10000 population) leprosy[4]. Chhattisgarh state in India was among the top five states that reported more cases in 2016-2017 [5] and the prevalence of grade 2 disability was higher (>12.5%) in the Pandariya block, Kabhirdham district. We evaluated NLEP surveillance in Pandariya block to identify the gaps.

**Methods**

We conducted a cross-sectional survey in selected health facilities of Pandariya Block, Kabirdham (Kawardha) District in Chhattisgarh state. We selected health facilities based on high G2D rate (>12.5%). We reviewed programme guidelines, surveillance formats and conducted key informant interviews with stakeholders. Using Centers for Disease Control and Prevention guidelines, we evaluated leprosy surveillance system. We assessed qualitative attributes (simplicity, flexibility and usefulness) from key informant interviews. We assessed quantitative attributes (data quality, acceptability, predictive value positive, representativeness, timeliness and stability) from surveillance data collected between April – June 2019. We calculated proportions to evaluate all attributes.

**Results**

We interviewed 26 health care workers including medical officers, assistant medical officers, non-medical supervisors, non-medical assistants and female field-level health workers (mitanins) from 8 primary health centres and two community health centres in Pandariya block. NLEP was passive surveillance and health facilities submitted monthly progress report (MPR). At the peripheral level, suspected cases detected by field-level workers (mitanins) were immediately reported to the primary health centre. It was reported to block medical officer (BMO) through MPR format by 25th of every month at the block level. At the district level, MPR format was filled and sent to district leprosy officer by 30th of every month. From the districts, all reports from the reporting units were consolidated and sent to state leprosy division by 5th of every month and the state sent the report to central leprosy division. Center gave feedback to states every year. Among interviewed staff, 81% were aware of the case definition and, 69% were filling patient forms correctly. One new variable was added in reporting format in April 2018. All reporting units (RU) were reporting through a paper-based system. Monthly reports were received on time at block and district. Missing data was observed in all reporting forms. Predictive-value-positive was 12.5% and 5.2% for district and block respectively. There was no reporting from the private sector.

**Conclusion**

We had some limitations in our evaluation. The surveillance system's sensitivity was not assessed due to the unavailability of the true burden of disease in the community and limited resources available to undertake any survey during the evaluation period. We were not able to assess for data quality (validity) as referral forms were not available at the peripheral level (PHC). The Leprosy surveillance system in Pandariya block, Kawardha district was simple, flexible, timely, acceptable and useful. Data quality, representativeness, and positive predictive value need improvement. Gaps should be identified in terms of human resources, logistics, training and funds to improve government and private hospital reporting.

**Acknowledgements**

We thank Dr Madhav Raj Deshpande, State leprosy officer, Chhattisgarh, Dr B.L Raj, District leprosy officer of Kawardha district, Mr Sarwat Hussain Naqvi, NLEP Consultant Chhattisgarh & Maharashtra, Dr Sarosh Jamil, WHO Consultant, Mr Nafeez, data entry operator, all the Medical Officers, Assistant Medical Officers and other NLEP Staff for providing all the required data and their invaluable support in the conduct of this study.

**References**

[1]. Blok DJ, De Vlas SJ, Richardus JH. Global elimination of leprosy by 2020: are we on track? Parasit Vectors. 2015 Oct 22;8(1):548.

[2]. Leprosy:WHO Classification ,WHO Disability grading - www.medicoapps.org [Internet]. [cited 2021 Feb 8]. Available from: https://medicoapps.org/m-leprosywho-classification-who-disability-grading/

[3]. MO Training Manual.pdf [Internet]. [cited 2021 Feb 8]. Available from: https://www.dghs.gov.in/WriteReadData/userfiles/file/Leprosy/MO%20Training%20Manual.pdf

[4]. Directorate General Of Health Services [Internet]. [cited 2021 Feb 8]. Available from: https://www.dghs.gov.in/content/1349_3_NationalLeprosyEradicationProgramme.aspx

[5]. Leprosy Is Making A Comeback In India, But The Govt Wants to Deny It [Internet]. The Wire. [cited 2021 Feb 8]. Available from: https://thewire.in/health/leprosy-is-making-a-comeback-in-india-but-the-govt-wants-to-deny-it

**Table 1 (abstract A5).** Attribute wise evaluation of National Leprosy Eradication Programme Surveillance System at Kawardha district, Chhattisgarh, April — June 2019

Attribute	Indicators	Results (%)	Evaluation
Simplicity (April-June 2019)*	Awareness of key informants on case definition for leprosy case suspect	81% (21/26)	Good
	Proportion of key informants who found it easy to fill patient form	69% (18/26) Correct reporting	
Flexibility (April 2018-March 2019)	Change in reporting format during the reference period	New variable (patient from other state) added in reporting format in April 2018	Flexible
	Change in the reporting frequency and mechanism (paper/electronic)	No change (block and district level)	
Data quality - (Completeness)(April 2019-June 2019)	Proportion of completed MPR	None (0/12)	Poor
Acceptability (April-June 2019)	Proportion of health facilities reporting through routine surveillance (paper based)	100% (12/12)	Good
Predictive value positive (April-June 2019)	Proportion of cases confirmed by MO’s or assistant MO’s in district and block	District 12.5% (40/320) Block 5.2% (8/154)	Poor
Representativeness (April-June 2019)	Proportion of private facilities reporting in NLEP	None	Poor
Timeliness (April-June 2019)	Proportion of reports received on time at block, district and state	100%	Good
Stability (April -June 2019)	Proportion of NLEP DEO (data entry operator) trained	None Non-Medical Assistant (NMA) compiles the collected data at block	Not Stable
Usefulness (April 2016-March 2019)	Surveillance system helping in doing contact tracing	Routine surveillance and leprosy case detection campaign helped in detecting cases among contacts	Good

*Reference period based on the availability of data.

## A6 Descriptive epidemiology of acute encephalopathy syndrome outbreak in Muzaffarpur district, Bihar, India from May–July 2019

### Vaisakh T P^1^, Rajeev Kumar^1^, Abhishek Mishra^1^, Binoy S Babu^1^, Purvi Patel^1^, Tanzin Dikid^1^, Ramesh Chandra^1^, Rajesh Yadav^2^, Mohan Papanna^2^, Anoop Velayudhan^2^, Saurabh Goel^1^, Suhas Dhandore^1^, Ajit Shewale^1^, Manickam Ponnaiah^3^, Manoj Murhekar^3^, Ravindra Prasad^4^, SK Jain^1^, Sujeet Singh^1^

#### ^1^National Centre for Disease Control, New Delhi, Delhi, India; ^2^U.S. Centers for Disease Control and Prevention, New Delhi, Delhi, India; ^3^ICMR-National Institute of Epidemiology, Chennai, Tamil Nadu, India; ^4^Community Medicine Department, Shri Krishna Medical College Hospital, Muzaffarpur, Bihar, India

##### **Correspondence:** Vaisakh T P (vaisakhtp@gmail.com)

**Introduction**

Acute encephalopathy syndrome (AES) is characterized by sudden onset of seizures and altered sensorium of infectious or non-infectious origin. Seasonal outbreaks of fatal hypoglycaemic AES in children, associated with eating fruit from the *Sapindaceae* family (e.g., ackee, litchi), have been reported globally [1,2]. Since 1995, AES outbreaks have been reported during the litchi-harvesting season from May–July in Muzaffarpur, the largest commercial litchi-producing district of Bihar, India [3]. An AES outbreak investigation in Muzaffarpur in 2014 linked known toxins hypoglycin A and α-methylene cyclopropyl glycine (MCPG) in litchi fruit to hypoglycaemic AES in children [3]. Following the 2014 outbreak, the Government of Bihar implemented community-based interventions to prevent hypoglycemia in children. They also strengthened the clinical management of hypoglycaemic seizures in public health facilities [4]. The number of AES cases and deaths declined from 2015–18, suggesting that the interventions were effective. However, in May–June 2019, AES cases increased. We conducted a descriptive epidemiological analysis of the AES cases.

**Methods**

We identified AES cases from established hospital-based surveillance in the two tertiary referral hospitals in Muzaffarpur. We defined a suspected AES case as seizures *or* altered sensorium in a child aged ≤15 years admitted from 1 May to 2^nd^ July 2019. We excluded patients aged six months to 6 years who were admitted for fever and a single generalized convulsion of <15 minutes in duration and recovered consciousness within 60 minutes of seizure. We conducted a review of medical records and abstracted data using a structured tool for socio-demographics, clinical history, duration of hospitalization, treatment, and laboratory profile. We also assembled a prospective cohort of probable cases admitted to the hospital during the investigation. We defined a probable AES case as *new-onset* seizures or altered sensorium of *<7 days duration* in a child aged ≤15 years admitted t from 1 May to 2 July 2019. For the cohort of probable cases, we interviewed the caregivers using a structured clinical-epidemiological questionnaire for socio-demographics, anthropometry, illness characteristics, treatment-seeking behavior, meal assessment, exposure to litchi fruit, and exposure to health messages. For anthropometry, we calculated Z-scores using the World Health Organization 2006 standardized growth tables [5].

**Results**

Of the 655 suspected and probable AES cases identified, the case fatality rate (CFR) was 21% (139 deaths). The median age was four years (interquartile range: 3 months–14 years), and 58% (378) were females. The first case was reported on 5 May 2019, cases peaked on 15 June, and the last case on 2 July (Figure 1). Among cases with available data, 75% (389/518) had blood glucose levels of <70 mg/dL upon hospital admission, and 75% (476/638) were residents of Muzaffarpur district. We identified cases from 15 (94%) of 16 blocks in the Muzaffarpur district and calculated a district incidence of 22 per 100,000 children ≤15 years old.

The prospective cohort comprised 94 probable AES cases; CFR was 26%. Among probable cases, 63% (49/78) of caregivers were wage workers, and 34% (31/91) were of low socioeconomic status. Symptoms were reported in the early morning (3 am to 8 am) for 67% (62/93) of cases, and 97% (90/93) presented with seizures. Among probable cases with anthropometry data, 62% (43/69) were underweight (i.e., weight-for-age Z score <-2), 44% (25/57) stunted (i.e., height-for-age Z score <-2), and 43% (10/23) wasted (i.e., weight-for-height Z score <-2). Primary health facilities referred 46% (43/93) of probable cases to the two tertiary hospitals for admission. Among cases referred, only 30% (13/43) received hypoglycemia and seizure management at the primary health facility.

Eating litchis in the 24 hours and seven days before illness onset was reported by 57% (54/94) and 87% (59/68) of caregivers, respectively. Skipping any meal and skipping the evening meal in the 24 hours before illness onset was reported by 55% (48/88) and 44% (28/63) of caregivers, respectively. Among probable cases, 45% (27/60) of caregivers reported Government Supplementary Nutrition (GSN) programme enrollment. Sixty percent (50/83) of caregivers said a visit by health workers in the week before illness. Still, only 8% (7/83) reported receiving messages on AES prevention and early treatment by health workers in the past month.

**Conclusions**

The 2019 AES outbreak in Muzaffarpur district, Bihar, occurred among young children with hypoglycemia upon hospital admission and had high associated mortality. Although the Government of Bihar implemented community and clinical measures to prevent AES cases after the 2014 outbreak, a large proportion of the AES cases did not benefit from the prevention measures based on our investigation [4]. New state and district health leadership, turnover of community and facility-level healthcare workers, lack of ongoing training and focused community outreach, and competing health priorities might have been factors responsible for the resurgence. To prevent future AES cases, we recommended prompt emergency management of hypoglycemia and seizures at primary health facilities before referral. We recommend enrollment of all eligible children to GSN and enhanced community health communications to reinforce the importance of an evening meal for children and limiting the eating of litchi fruit during the harvesting season from May to July.

**References**

1. Spencer PS, Palmer VS. The enigma of litchi toxicity: an emerging health concern in southern Asia. The Lancet Global Health. 2017 Apr 1;5(4):e383-4.

2. Zhang LJ, Fontaine RE. Lychee-associated encephalopathy in China and its reduction since 2000. The Lancet Global Health. 2017 Sep 1;5(9):e865.

3. Shrivastava A, Kumar A, Thomas JD, Laserson KF, Bhushan G, Carter MD, Chhabra M, Mittal V, Khare S, Sejvar JJ, Dwivedi M. Association of acute toxic encephalopathy with litchi consumption in an outbreak in Muzaffarpur, India, 2014: a case-control study. The Lancet Global Health. 2017 Apr 1;5(4):e458-66.

4. Shrivastava A, Srikantiah P, Kumar A, Bhushan G, Goel K, Kumar S, Kumar T, Mohankumar R, Pandey R, Pathan P, Tulsian Y. Outbreaks of unexplained neurologic illness—Muzaffarpur, India, 2013–2014. MMWR. Morbidity and mortality weekly report. 2015 30 January;64(3):49.

5. WHO Multicentre Growth Reference Study Group, de Onis M. Assessment of differences in linear growth among populations in the WHO Multicentre Growth Reference Study. Acta Paediatrica. 2006 Apr;95:56-65.

**Fig. 1 (abstract A6).**
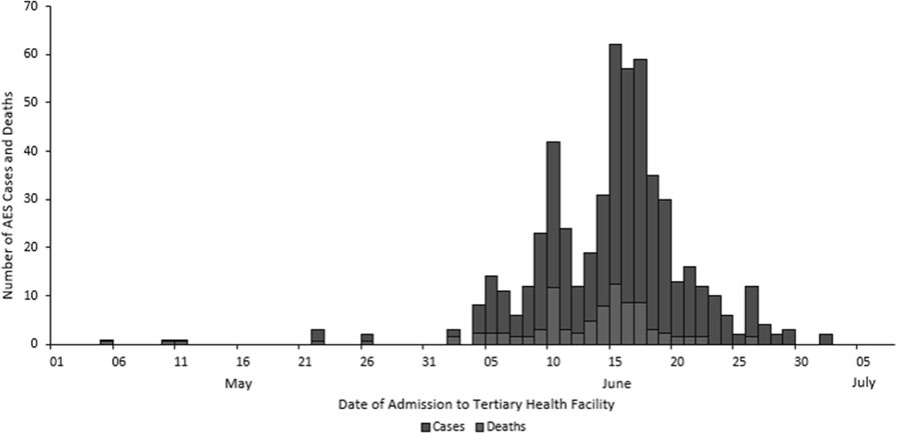
Distribution of acute encephalopathy syndrome cases and deaths by date of admission to two tertiary health facilities, Muzaffarpur, Bihar, India, from 1 May to 2 July 2019 (n=538)

## A7 Investigation of an acute diarrhoeal disease outbreak from contaminated drinking water supply, Village A, Patna, Bihar, India July–August 2019

### Ujjawal P Sinha, Kevisetuo A Dzeyie, Lucky Sangal, Pankaj Bhatnagar

#### National Public Health Surveillance Project, World Health Organization Country Office, New Delhi, India

##### **Correspondence:** Ujjawal P Sinha (drujjawal@gmail.com)

**Background**

Globally, acute diarrhoeal disease causes 525,000 deaths annually [1]. The Integrated Disease Surveillance Programme reported 1,850 acute diarrhoeal disease outbreaks during 2018 throughout India, of which 19 outbreaks occurred in Bihar [2]. On July 4, 2019, the sub-divisional hospital in Masaurhi Block, Patna, reported 28 acute diarrhoeal disease cases from Village A. This village has 164 houses with a predominantly underserved population of 1,074 persons. The district medical officer initiated the preliminary investigation, medical treatment, and referral of case-patients. During July 5–11, 2019, frontline health workers conducted active case searches in Village A and identified additional cases. On August 7, 2019, the World Health Organization Epidemic Intelligence Service Officer joined the outbreak investigation team to describe the epidemiology, identify risk factors, and recommend evidence-based prevention and control measures for the outbreak.

**Methods**

We defined a diarrhoea case as three or more loose stools in less than 24 hours in Village A resident during June 27–July 20, 2019. We searched for case-patients with the above definition by conducting a house-to-house survey and reviewed outpatient records of the block sub-divisional hospital and primary health centre. We conducted an unmatched case-control study and compared cases with the same number of randomly selected controls for risk factors assuming 90% power, odds ratio (OR) of 3, and 50% exposure among controls using Epi Info software [https://www.cdc.gov/epiinfo]. We included apparently healthy residents during the same period of Village A, Masaurhi Block as controls. We collected information about demographic characteristics, clinical presentation, treatment history, exposure factors (source of drinking water, method of purification, and sanitation) using a semi-structured questionnaire. We calculated proportions, median age, attack rate, case-fatality rate, and OR with 95% confidence intervals (CIs) for risk factors. Besides, we collected stool samples from 2 case-patients for bacteriologic culture, tested water samples collected from government-supplied tap water for residual chlorine, and observed drainage systems in the affected area.

**Results**

We identified 81 case-patients (68% female) during June 20–August 11; the median age was 25 years (range 1–85 years). The overall attack was 7.5% (81/1,074); persons ≥60 years of age had the highest attack rate (10/68, 15%). Four (5%) persons died in 4 different households. Of the four deaths, three (75%) occurred in females; three persons who died were >65 years of age, and three were admitted to a hospital. One died at home without any treatment. Cases were distributed throughout Village A and clustered in four households that each reported more than 1 case. The outbreak began on July 2, 2019, peaked on July 3, 2019, with 28 cases, and rapidly declined after that; the last case was reported on July 12, 2019. In addition to diarrhea, case-patients reported abdominal pain (57/81, 70%), fever (55/81, 68%), and vomiting (9/81, 11%). Eighty (99%) case-patients visited health facilities; 34 (41%) visited more than one health facility for treatment. A local nonmedical healer conducted initial diarrhoeal management of 34 (41%) case-patients, 61 (75%) visited the sub-divisional hospital, and 19 (23%) visited private clinics. Among case-patients, 73 (90%) reported using the government tap water supply as their drinking water source, and 30 (37%) boiled water before drinking. In the case-control study of 81 cases and 81 controls, drinking government-supplied tap water (OR 16.4 [95% CI 6.9–38.7]) was associated with illness. Drinking bore well water (OR 0.9 [95% CI 0.026–0.32), hand-pumped water (OR 0.1 [95% CI 0.05–0.3]), and water treated before drinking (OR 0.2 [95% CI 0.1-0.4]) were protective (*Table 1*). Laboratory investigation of 2 fecal samples did not detect any pathogenic organism. Testing one water sample did not detect bacteria, and the residual chlorine level was within the permissible limit of 250 mg/dL [3]. We observed a poor drainage system with waterlogging and overflowing that provided potential contamination of water supply pipes.

**Conclusions**

In this investigation of an acute diarrhoeal disease outbreak, the illness was probably more likely associated with government-supplied tap water contamination. We recommended that local health authorities create community awareness through frontline workers to boil water or use filtration methods before drinking government-supplied tap water. Besides, we recommended that the Masaurhi sub-district hospital in charge and district administration ensure a regular supply of clean drinking water by regular chlorination, conduct periodic quality checks, and immediately repair the damaged drainage system. Prompt response by the district health authorities in treating case-patients and decontaminating drinking water sources helped control the outbreak.

**References**

1. Diarrhoeal disease key facts [Internet]. World Health Organization. [cited 2020 Jan 2]. Available from: https://www.who.int/news-room/fact-sheets/detail/diarrhoeal-disease

2. Disease Alert 2018 [Internet]. Integrated Disease Surveillance Programme, National Centre for Disease Control. [cited 2020 Jan 2]. Available from: https://idsp.nic.in/index1.php?lang=1&level=1&sublinkid=6008&lid=3942

3. Chlorine in Drinking-water. Background document for development of WHO Guidelines for Drinking-water Quality [Internet]. World Health Organization; 1996 [cited 2020 Jan 2]. Available from: https://www.who.int/water_sanitation_health/water-quality/guidelines/chemicals/chlorine.pdf?ua=1

**Table 1 (abstract A7).** Risk factors for acute diarrhoeal disease in 162 persons, Village A, Masaurhi Block, District Patna, Bihar, India, 2019

Exposures	Cases n (%), N = 81	Controls n (%) N= 81	OR (95% CI)
No handwashing with soap after defecation	1 (98)	0 (100)	3.08 (0.3–30.2)
Being female	55 (68)	31 (38)	3.41 (1.8–6.5)
Drinking water source			
Government-supplied tap water	73 (90)	29 (36)	16.36 (6.9–38.7)
Borewell water	3 (4)	24 (30)	0.91 (0.03–0.3)
Hand pumped water	5 (6)	28 (34)	0.12 (0.05–0.3)
Treated water before drinking	30 (37)	60 (74)	0.21 (0.1–0.4)
Ingested non-homemade food in previous 3 days before illness	9 (11)	27 (33)	0.25 (0.1–0.6)
Drank water from alternate supply	8 (10)	30 (37)	0.19 (0.1–0.4)

## A8 Effect of community-led weekly pulse cleaning of water collections in controlling dengue outbreak at Alipurduar, West Bengal, India, 2019

### Puran Kumar Sharma, Subarna Goswami, Kousik Choudhury, Ananta Maji, Golam Mortuja

#### Office of the CMOH, Alipurduar, Department of Health & Family Welfare, Govt of West Bengal

##### **Correspondence:** Puran Kumar Sharma (puran.sharma611@gmail.com)

**Background**

Dengue fever is a recurrent problem in West Bengal (India), with numerous outbreaks during the monsoons and post monsoons[1]. India has an open international border with Bhutan at Jaigaon, and the transmission of dengue through infected travelers is a cause for concern. Dengue was first reported in Siliguri in the northern part of West Bengal in July 2005[2]. After the first outbreak of dengue in 2004 in Phuntsholing city in the Chukkah district, Bhutan has reported many dengue outbreaks[3]. Three serotypes (DEN1, DEN2, and DEN3) have been detected in Phuntosholing^2^, while only the DEN3 serotype is reported from Jaigaon.

Jaigaon Development Authority (JDA) is a notified area in the Kalchini block of Alipurduar district of West Bengal, India, with 88,214 and poor civic amenities but a robust peripheral health care system with multi-purpose health workers and health sub-centers. Phuntsholing city in the Chhukah district of Bhutan has 27,658 and no peripheral health care system. As dengue incidence was over the previous years (45, 239, and 2 cases in 2016, 2017, and 2018 respectively) compared to around 250 dengue cases in July 2019, an outbreak was declared in Jaigaon.

We investigated the outbreak to identify the etiology and source of it, to undertake coordinated vector control measures along with the public health team of Bhutan. We also compared the effectiveness of community-led weekly fixed day pulse cleaning in Jaigaon compared to routine interventions in Phuntsholing, Bhutan, to control the outbreak.

**Methods**

We reviewed the surveillance data for previous years to confirm the outbreak. A case was defined as a person with febrile illness of 2-7 days duration with headache, retro-orbital pain, arthralgia, myalgia, rash, and bleeding (any two) with ELISA confirmed NS1 or IgM for dengue during June-November 2019. Trained volunteers conducted an active house to house survey to identify febrile cases for dengue testing and management based on dengue case definition. Also, daily data was collected from the local Primary Health Centre (PHC) and district hospital.

Trained volunteers undertook weekly fixed day house to house visits to look for domestic, peri-domestic, and workplace water collections and detect mosquito larvae. They collected larvae and adult vector mosquitoes for entomological studies and dried all the water containers/collections after the weekly prefixed pulse cleaning drive.

Blood samples were taken from febrile people meeting the case definition of dengue and tested using ELISA based NS1 antigen and IgM antibody test to confirm dengue. Serum of NS1 or IgM confirmed dengue cases were sent for serotyping of the dengue virus to the Virology Unit of the National Institute of Cholera and Enteric Diseases (NICED) at Kolkata. Data of ELISA based NS1 or IgM positive cases and dengue virus serotype in Phuntsholing were collected from the office of the District Health Officer.

Attack rates (Total dengue cases/population x 100) and Case Fatality Ratio (CFR – Total deaths due to dengue/total dengue cases x 100) for Jaigaon and Phuntsholing were calculated. Epicurve was plotted to describe the time trend.

**Results**:

A total of 1249 cases and two deaths were recorded in Jaigaon with an attack rate of 1.4%, while a total of 3223 patients and five deaths were recorded at Phuntsholing with an attack rate of 11.7%.

An index case in Phuntsholing was reported on June 15, 2019 (27^th^ week), and the cases peaked on the 36^th^ week with 436 cases and were reduced to 78 cases a week in the 41^st^ week. The index case in Jaigaon was reported on July 14, with cases peaking in the 36^th^ week with 252 cases but drastically reduced following the weekly prefixed pulse cleaning activities to 13 cases in the 41^st^ week (Figure 1).

While 19 (95%) of the samples sent from Jaigaon were reported to be DENV 3, nine (39%) and seven (34%) samples sent from Phuntsholing were reported to be DENV 2 and DENV 3, respectively.

Major vector breeding sites were garages, tire repair shops, scrap shops, slaughterhouses, domestic water containers, and receptacles in openly disposed of solid waste. Adult *Aedes* species were identified from the adult collections. Larvae were reared in the entomological laboratory through different stages to identify the Aedes species using taxonomical identification keys. *Aedes albopictus* (70%) and *Aedes aegypti* (30%) were the major dengue vectors

Jaigaon has the basic dengue prevention and control measures, including the fortnightly house to house surveillance and vector control measures supported by the state health department. In addition, weekly fixed day pulse cleaning of water collections involving the community and other stakeholders was initiated. After implementing the weekly fixed day pulse cleaning activities with active community involvement, the attack rates (AR) in Jaigaon decreased from 0.28% (252 cases) in the 36^th^ week to 0.01% (13 cases) in the 41^st^ week. On the other hand, AR in the 36^th^ week at Phuntsholing was1.58% (436 cases) and reduced to 0.28% (78 cases) in the 41^st^ week.

**Conclusion**

Community-led weekly fixed day pulse cleaning activities resulted in effectively containing the dengue outbreak at Jaigaon. The absence of such public health intervention in Phuntsholing resulted in the delayed control of dengue outbreaks with high morbidity. We recommended adopting the cost-effective routine community-led weekly fixed day pulse cleaning activity in all areas for prevention and control of dengue from May to November every year. We also recommended using larvicides for stored water stored which could not be discarded in areas where the existing government supply was inadequate.

**References**

1. Bandyopadhyay B, Bhattacharyya I, Adhikary S, Konar J, Dawar N, Sarkar J, Mondal S, Singh Chauhan M, Bhattacharya N, Chakravarty A, Biswas A. A comprehensive study on the 2012 dengue fever outbreak in Kolkata, India. International Scholarly Research Notices. 2013;2013.

2. Taraphdar D, Sarkar A, Bhattacharya MK, Chatterjee S. Serodiagnosis of dengue activity in an unknown febrile outbreak at the Siliguri Town, District Darjeeling, West Bengal. Asian Pacific Journal of Tropical Medicine. 2010 May 1;3(5):364-6.

3. Dorji T, Yoon IK, Holmes EC, Wangchuk S, Tobgay T, Nisalak A, Chinnawirotpisan P, Sangkachantaranon K, Gibbons RV, Jarman RG. Diversity and origin of dengue virus serotype 1, 2, and 3, Bhutan. Emerging Infectious Diseases. 2009 Oct;15(10):1630.

**Fig. 1 (abstract A8).**
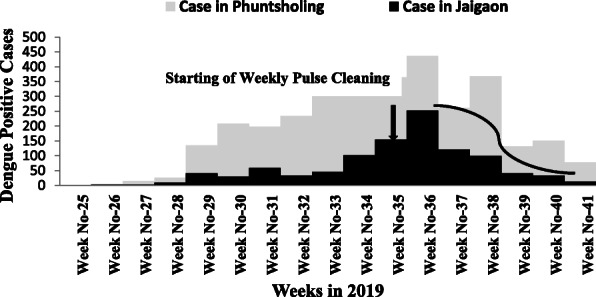
Number of cases of dengue by week, Alipurduar, West Bengal, India and Phuntsholing, Bhutan, 2019

## A9 Descriptive epidemiology of recent three Influenza AH1N1 outbreaks in Puducherry district, India, 2019

### Lakshmanasamy Ravivarman, Prabhdeep Kaur

#### ICMR-National Institute of Epidemiology, Chennai, India

##### **Correspondence:** Lakshmanasamy Ravivarman (drravivarman@gmail.com)

**Background**

Influenza viruses have reported frequent changes in strains in recent years. The current strain of AH1N1 that affected the world was in 2009. WHO declared it as a pandemic. The first case in India was reported in Hyderabad, Andhra Pradesh in May, 2009.(1) The pdm09 strain of influenza A H1N1 is in circulation across the globe and in India since 2009 with frequent outbreaks. Over a decade, the strain of influenza had varied morbidity and mortality across socio-demographic domains. The disease has become more an endemic with seasonal outbreaks and varied patterns of presentation.(2) Puducherry is a highly urbanized settlement with very high density of population in the region (3231 people per sq. km). Puducherry had three major outbreaks of H1N1 influenza in the past five years. We did an outbreak investigation of the cluster of cases reported in October 2018 with the objective to confirm the outbreak and to compare the outbreak with the previous two outbreaks and identify patterns, if any in Puducherry district, India

**Methods**

We defined ‘case’ as a person with fever and/or sore throat and throat swab positive for H1N1 A influenza through polymerase chain reaction at designated laboratories in Puducherry between September 2018 and January 2019. We described the cases by time, place and person.

Subsequently, we retrieved data of the previous two outbreaks from the state surveillance office, health department. We cleaned the data and consolidated the line list of laboratory confirmed H1N1 influenza cases by standard polymerase chain reaction assay. We described the outbreaks by time, place, person and examined for patterns. We calculated the overall attack rate and age-specific attack rate. We calculated overall and age-specific case fatality ratio. We drew epidemic curves of all three outbreaks. We calculated the proportion of various symptoms in the patients. We also explored for any unique characteristics in these outbreaks. We did the analysis using epiinfo software version 3.5.1.

**Results**

The three outbreaks occurred over the past five years. A total of 494 laboratory confirmed cases were reported. The overall attack rate ranged between 7 to 35 per 1,00,000 population and case fatality ratio ranged between 5.4% to 11.3% (Table 01). The attack rate and case fatality ratio were high in extremes of age in all three outbreaks. On spatial analysis, there was no urban and rural difference in incidence. Fever and cough were the predominant symptoms. On exploring for any unique characteristics, we found that the healthcare employees were the major case patients in the first half of epidemic curve in all the three outbreaks. It was observed that the deaths due to influenza A H1N1 were confined only to the first half of the epidemic curve in the recent two outbreaks.

**Conclusion**

Influenza A H1N1 outbreaks are frequent. The more common symptoms were fever, cough and sore throat. In all the three outbreaks studied in Puducherry, extremes of age groups were the most affected, unlike in a few studies in India, where mid-age population was more affected.(1,3) Deaths were more common in the elderly age groups in line with other studies.(1,4). On examination of the case patient details, it was observed that healthcare workers were more affected in the initial durations of the influenza spread. The initial spread happened in the healthcare settings and gradually spread to the community. Therefore, the vulnerability of the healthcare employees to the infection is highlighted. Infection control practices in the hospital settings should be strictly adhered to. Also proactive seasonal influenza vaccination of the health workers may be considered. Another observation common in two of the recent outbreaks was that the deaths that happened during the outbreaks have entirely occurred during the first half of the outbreak duration. It is probable that the knowledge of the onset of influenza outbreak is not there, so that patients are not started on due antiviral drugs in time or late presentation to healthcare setting due to delayed active surveillance. This is also observed in a study in Tamil Nadu, India in 2010.(4)

We recommend early detection of the outbreaks and cases by symptoms analysis, especially in the extreme age groups and vulnerable population like healthcare employees. More studies in these areas are needed to affirm the observations made in this study.

**References**

1. Allam RR, Murhekar MV, Tadi GP, Udaragudi PR. Descriptive epidemiology of novel influenza A (H1N1), Andhra Pradesh 2009-2010. Indian J Public Health. 2013 Sep;57(3):161–5.

2. Kulkarni SV, Narain JP, Gupta S, Dhariwal AC, Singh SK, Macintyre CR. Influenza A (H1N1) in India: Changing epidemiology and its implications. Natl Med J India. 2019 Apr;32(2):107–8.

3. Dwibedi B, Sabat J, Dixit S, Rathore S, Subhadra S, Panda S, et al. Epidemiological and clinical profile of Influenza A(H1N1) pdm09 in Odisha, eastern India. Heliyon. 2019 Oct 1;5(10):e02639.

4. Balaganesakumar SR, Murhekar MV, Swamy KK, Kumar MR, Manickam P, Pandian P. Risk factors associated with death among influenza A (H1N1) patients, Tamil Nadu, India, 2010. J Postgrad Med. 2013 Mar;59(1):9–14.

**Table 1 (abstract A9).** Comparison of age-specific attack rate and case fatality ratio for recent three outbreaks of H1N1 A influenza, Puducherry district. 2019

Age Group (In years)	2018		2017		2015	
	Attack rate (per 100,000)	Case fatality ratio (%)	Attack rate (per 100,000)	Case fatality ratio (%)	Attack rate (per 100,000)	Case fatality ratio (%)
0-5	46.7	3.5	19.8	0	14.1	10
5-15	15	-	8.5	0	3.27	0
15-25	21.4	-	10.7	0	3.15	0
25-35	31.6	-	14.7	7.7	6.76	8.3
35-50	26.3	3.9	15.9	8.8	4.22	11.1
50-60	49.5	7.7	27.1	17.4	10.6	11.1
>60	53.6	6.5	25.2	0	8.75	0

## A10 Medically certified causes of death and risk factors in mortality, Puducherry district, India, 2016-19

### Lakshmanasamy Ravivarman, Prabhdeep Kaur

#### ICMR-National Institute of Epidemiology, Chennai, India

##### **Correspondence:** Lakshmanasamy Ravivarman (drravivarman@gmail.com)

**Background**

India has about 9.5 million deaths a year, or about one in six of all deaths worldwide. Over three-quarters of deaths in India occur in the home; more than half of these do not have a certified cause [1]. Historically reliable, representative, routine, low-cost, and long-term mortality measurements are the key to monitoring trends in health conditions of the population. It includes detecting new epidemics, spurring research into avoidable causes of death, evaluating control programs' success, and improving accountability for disease control expenditures [2]. The causes of death in Puducherry is not studied before. Puducherry has a vibrant health infrastructure, which is easily accessible and affordable. Therefore the majority of the deaths happen only in major tertiary care health institutions. An analysis of the same would help understand population mortality characteristics and serve as a future trend analysis base. We conducted this study in a headquarters tertiary care hospital of Puducherry, which caters to the population's predominant section. The study was done to describe the causes of death based on medical certificates and to describe the pre-existing diseases and risk factors among deceased in Puducherry district, 2016-2019.

**Methods**

We conducted a cross-sectional study of the mortality data from the district headquarters hospital. We did a secondary data analysis of mortality data of the patients admitted and expired from the Government General Hospital of Puducherry district from 2016 to 2019 were obtained from the Medical Records department. The data was available in an MS Excel spreadsheet maintained in the Medical Records department. We cleaned the data and analyzed using R software. The causes of death were categorized into primary and secondary causes. The deaths were coded according to the International Classification of Diseases-10 (ICD-10). The proportion of deaths in various categories as per ICD-10 were calculated. The proportion of the pre-existing disease conditions among the deceased were calculated. The key risk factors based on the proportion of contribution to the number of deaths were identified and stratified by age and gender.

**Results**

A total of 4268 deaths were recorded between 2016 and 2019 in the Medical Records department. One thousand fifty-seven unique causes for deaths were obtained on splitting the cause of death into the primary cause and multiple preceding causes, declared in the death certificates of the total deceased by the treating doctors. About 31% died due to cardiovascular diseases (I00-I99) as the primary cause. Another 21% died due to external causes (S00-Y98) of death as categorized by ICD 10. The digestive system, respiratory system, and infectious diseases were the subsequent causes, in descending order of proportion (Figure.1). On analyzing the pre-existing causes, 20% of the deceased had hypertension, 19% had diabetes. Stroke, heart diseases, renal failure, and chronic alcoholism were the next preceding causes (in order). Deaths due to circulatory system causes were increasing over the years. Hypertension was identified as the critical risk factor among the deceased. Eight hundred thirty-four among the total dead had the code word 'hypertension' in the death records. On age group analysis, 52% were more than 65 years of age, and 80% were above 50. By age group-gender analysis of deceased, men with hypertension were in higher proportion in younger age group (40% men against 25% in women).

**Conclusions**

Our study is based on facility mortality data and therefore has certain limitations. A few departments like obstetrics and gynecology, pediatrics, and super-specialty services like the oncology department were not available in the facility. The mortality in these areas is probably underrepresented. Otherwise, with the results available, it is understood that, like other studies in the developing countries [3, 4], cardiovascular diseases were the major cause of mortality in the population. Next in line is the external causes like road traffic accidents and suicidal deaths. A significant finding is that alcohol-related deaths (10%) were among the leading cause in this population. The majority of the external causes of death, as per ICD-10 classification, which includes road traffic accidents and suicidal deaths, had alcoholism as a preceding risk factor. Hypertension and diabetes are the leading pre-existing diseases. Among the hypertensives identified in the deceased population, half were in a premature age group (less than 69 years of age). Stroke, heart diseases, and renal diseases were the next causes, in decreasing order of proportion. The proportion of deaths due to infectious causes were low over the years studied, that the epidemiological transition has already occurred in this population. We recommend that early non-communicable diseases screening will help in diagnosing the diseases at an earlier stage and prevent mortality at a premature age. Blood pressure control ratio in the population needs to be assessed and brought under control. Further, policies focussed towards road safety and mental health programmes need due attention in this small union territory of the country.

**References**

1. Jha P, Gajalakshmi V, Gupta PC, Kumar R, Mony P, Dhingra N, Peto R, RGI-CGHR Prospective Study Collaborators. Prospective study of one million deaths in India: rationale, design, and validation results. PLoS Med. 2005 Dec 20;3(2):e18.

2. Jha P. Reliable mortality data: a powerful tool for public health. Natl Med J India. 2001 May-Jun;14(3):129-31

3. Palanivel C, Yadav K, Gupta V, Rai SK, Misra P, Krishnan A. Causes of death in rural adult population of North India (2002-2007), using verbal autopsy tool. Indian J Public Health. 2013 Apr 1;57(2):78

4. Rai SK, Kant S, Misra P, Srivastava R, Pandav CS. Cause of death during 2009-2012, using a probabilistic model (InterVA-4): an experience from Ballabgarh Health and Demographic Surveillance System in India. Glob Health Action. 2014 Oct 29;7:25573

**Fig. 1 (abstract A10).**
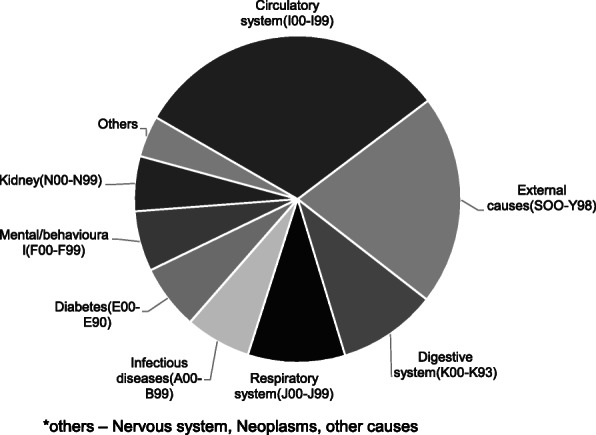
Causes of death categorised as per ICD 10, Puducherry district hospital, 2016-19 (N=4268). *others – Nervous system, Neoplasms, other causes

## A11 Malaria elimination in high transmission Hard-to-reach areas in the state of Odisha India

### Madan Mohan Pradhan^1,2^, Praveen Kishore Sahu^3^, Manoranjan Ranjit^4^, Ambarish Datta^5^, Sanghamitra Pati^4^

#### ^1^District Vector Borne Disease Control Programme, Boudh, Odisha, India; ^2^Ex. State Programme Officer, NVBDCP, Odisha, India; ^3^Molecular and Immunology Lab, Ispat General Hospital, Rourkela, Odisha, India^; 4^ICMR-Regional Medical Research Center, Bhubaneswar, Odisha, India; ^5^Public Health Foundation of India, Bhubaneswar, Odisha, India

##### **Correspondence:** Madan Mohan Pradhan (drmmpradhan@gmail.com)

**Background**

Malaria is a major public health crisis in the tropical region. Though the World Malaria Report indicated that malaria cases dropped from 239 million to 217 million between 2010 to 2017, the cumulative global response to malaria control is believed to be at the crossroads[1]. The rate of decline between 2016 to 2017 was particularly not encouraging at the global level. Meanwhile, India experienced a 24% decline in malaria during this period to which the state of Odisha was the highest contributor[1].

Due to a host of geo-ecological and social barriers, hard-to-reach or inaccessible areas get deprived of routine health care services, and often, the regular surveillance of infectious diseases like malaria gets hampered. In such under-surveillance villages, malaria cases are not detected and treated routinely and timely. Thus, there develops a herd-immunity in the community, and malaria infection does not manifest as febrile or other noticeable symptoms. These villages continue to act as ‘hotspots’ where malaria transmission persists due to asymptomatic infections [2]. These malaria hotspots appear to be the driving force, or so to say reservoirs, that maintain perennial malaria transmission; thus targeting them could prioritize the added benefits of reducing transmission to the whole community.

Most parts of Odisha state are conducive for malaria transmission. Different studies revealed a prevalence of 10 to 100% asymptomatic malaria infections in hardened reach villages in Odisha state. To address these hurdles, specifically in the hard-to-reach villages in high endemic districts, the state government took a unique stand. It launched a state-specific innovative program called DAMaN – ‘Durgama Anchalare Malaria Nirakaran’ meaning malaria eradication in inaccessible areas during mid-2017. This state-wide effort was based on the previous research experiences of the state’s malaria control program, “Comprehensive Case Management,” and experiences and findings of NGO have driven the “MITRA” project (unpublished). We describe whether DAMaN could significantly impact the malaria reduction in hard-to-reach areas of the malaria-riddled Indian state, Odisha.

**Methods**

DAMaN has three main components: (1) Mass screening and treatment (MSAT) of all positive malaria cases irrespective of fever as a symptom using bivalent rapid diagnostic test and anti-malarial drugs as per the National Drug Policy of India, (2) Enhancement of personal protection methods for Vector control, i.e., protection of entire population by Long-Lasting Insecticidal Nets and Indoor residual spray (LLIN/IRS) and (3) Intensive community awareness through the platforms of grass level health and ICDS workforces like Accredited Social Health Activists (ASHAs) and Anganwadi workers (AWW) and village volunteers. Simultaneously, the accessible villages were protected by LLINs along with other routine malaria control services, intensified community mobilization, and strengthened monitoring.

DAMaN is conducted in camp mode, 2-3 rounds annually, i.e., during pre-monsoon and post-monsoon months. In DAMaN camps and mass screening and treatment of all positive malaria cases irrespective of fever, vector control measures, health check-up of all <5 children, pregnant and lactating mothers to see their Haemoglobin nutrition status. From May to August 2017, >11 million LLINs were distributed to protect around 25 million population residing in all high malaria endemic districts and intensified community mobilization with multi-sectoral involvement. DAMAN camps immediately followed this. DAMaN camps were conducted in 2018 and 2019. Around 5000 DAMaN camps are held in each round, addressing >1.4 million population residing in hardened reach villages. Post follow up, activities are carried out by DAMaN volunteers to report any fever cases to the respective ASHA / Health worker along with mobilizing the community to sleep under LLIN regularly, Primary data is collected from community settings through serial surveys, and qualitative data related to DAMaN was collected from district malaria program and beneficiaries. Under this study, routine malaria data is collected from the State National Vector Borne Diseases Control Program (NVBDCP), both retrospective and prospective, besides published scientific literature.

**Results**

Each round of DAMaN screened ~1.4-million population of the state, mostly in the high endemic districts. The state-wide distribution of >11million LLINs protected ~25 million high-risk people from mosquito bites. There was a sharp decline of malaria cases after the initial implementation of DAMaN, in most of the Sub-centres with hard to reach villages by the NVBDCP of Odisha through its routine surveillance indicators, and the trend of decline continued throughout 2018 till 2019, and same is counting (Figure 1).

Overall, malaria case incidence in the state of Odisha. The drastic decline of malaria cases in Odisha interventions was reduced by 80-90% within two years (API >10 in 2016 to 1.48 in 2018) may be attributed to DAMaN and largescale LLIN distribution and reported malaria deaths were reduced from 27 in 2016 to 3 in 2018 (Figure 1).

Due to DAMaN intervention in communities residing in the remote and inaccessible pockets, the access to malaria-related services was significantly enhanced. Mass screening and treatment of all positive malaria infections reduced the parasite population, and LLIN reduced the anopheline mosquito population and prevented mosquito bites. Population having asymptomatic malaria could be identified and treated for parasite cleansing. Besides, nutritional deficiency and hemoglobin levels could also be characterized among under-five children and pregnant or lactating mothers.

**Conclusion**

The augmentation through a Government control program like DAMaN resulted in the sharp decline of malaria within a short span, which is historical in Odisha, India, and the entire world, demonstrating “High Burden High Impact.” This gives excellent hope towards malaria elimination. However, the rapid reduction of malaria cases by 80-90% has now put more challenging tasks for the program to deliver consistently in line with the National Framework for Malaria Elimination (NFME) goals by 2030 in India.

**References**

1. World malaria report 2017. Geneva: World Health Organization; 2017. Licence: CC BY-NC-SA 3.0 IGO.

2. Bousema T, Griffin JT, Sauerwein RW, Smith DL, Churcher TS, Takken W, et al. (2012) Hitting Hotspots: Spatial Targeting of Malaria for Control and Elimination. PLoS Med 9(1): e1001165. 10.1371/journal.pmed.1001165

**Fig. 1 (abstract A11).**
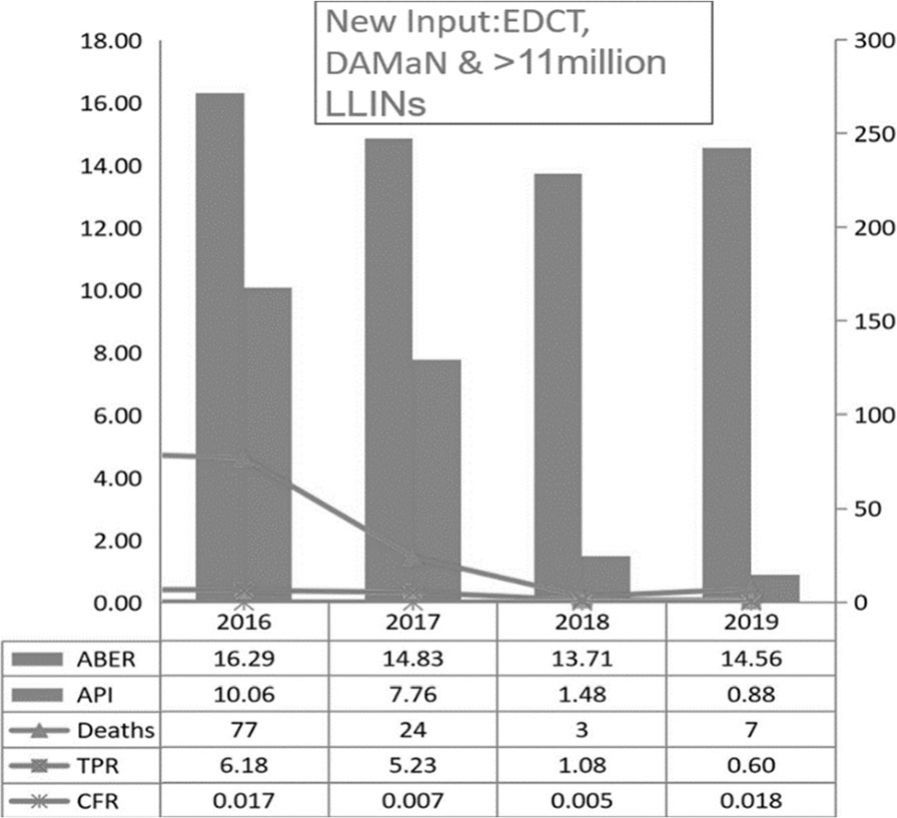
Depiction of the reduction of Annual Parasitological Index (API) from 10 to 0.83 & death cases 77 to 07 from 2016 to 2019 – a reduction of malaria incidence by 80-90% since 2016

## A12 Multi-specialty outpatient clinics (Polyclinics) for urban poor at Urban Primary Health Centers - Chennai, Tamil Nadu, India,2019

### Suganya Barani, Parasuraman Ganeshkumar, Tarun Bhatnagar

#### ICMR-National Institute of Epidemiology, Chennai, India

##### **Correspondence:** Suganya Barani (suganya.desmart@gmail.com)

**Background**: Chennai city is home to 7.9 million people, 32% of whom live in slums (1,2). The multi-specialty outpatient clinics called polyclinics were recently established within the Urban Primary Health Centers (UPHCs) to provide free outpatient services in the evenings, targeted towards the urban poor. In Chennai, 36 polyclinics were established by January 2018. The polyclinics are set convenient for the urban poor (4.30-8.30 PM) when they return from work(3). The urban poor does not seek the free morning OPDs in Urban Primary Health Centers due to loss of daily wages, and they incur high out-of-pocket expenditure accessing private clinics in the evenings (4). The polyclinics components include outpatient consultation by specialists, drugs and diagnostics, treatment and referral services. The specialty services are provided on a fixed day fixed specialty basis. General medicine, obstetrics, and gynecology, dermatology, ENT, ophthalmology, orthopedics, physiotherapy, dental treatment, psychiatry, and pediatrics are the specialties consulted in the polyclinic. We described and evaluated the provision of services in the polyclinics in 2019.

**Methods**: We conducted a cross-sectional study in Chennai using a logic model approach (5). We used two arms; namely, the provision of specialty services and information system, to evaluate the services (Figure 1). We developed input, process, and output indicators for both arms. For the provision of specialty services, we collected data on the availability of empaneled specialists, equipment, drugs, and clinical and diagnostic services. We also interviewed patients for their satisfaction using a semi-structured questionnaire. Assuming 50% expected satisfaction among patients, absolute precision of +/- 5%, and 95% confidence interval, the sample size was calculated at 420, including a non-response rate of 10%. We planned to interview 12 patients per polyclinic by consecutive sampling upon exiting from the consultation. We interviewed the empanelled specialists for their satisfaction and acceptability. Assuming satisfaction of 50% among specialists, absolute precision of +/- 10%, and 95%confidence interval, our sample size amounted to 82 with a non-responsive rate of 10%. For the arm on the information system, we collected data on the availability of materials for entering reports, a trained staff nurse, completeness of patient information in the web-based application

**Results**: We surveyed 36 polyclinics where 51900 patients accessed services between January to June 2019.

Input indicators: Of 36 polyclinics, 23(64%) had complete empanelment of specialists. Adequate funds for paying specialists in the past month were available in all 36 polyclinics. Availability of required equipment ranged from 39% to 100% in 36 polyclinics. All 36 polyclinics (100%) had received and installed dental equipment, while 31(86%) had physiotherapy and ophthalmology equipment. Only 14(39%) had ECG equipment, and 15(42%) had an Ultra sonogram. None of the polyclinics had a printed copy of polyclinic guidelines as specified in the guidelines. All the polyclinics (100%) had credentials for a web-based application. Desktop and internet connection was available in 33(91.7%) of the 36 polyclinics. In the 36 polyclinics, 28(77.8%) had a staff nurse with prior training in operating the web-based application.

Process indicators: 35(97.2%) polyclinics had installed IEC flex boards in the prescribed format. Recommended laboratory services were available and functional in evening polyclinic hours only in 5(14%) of 36 polyclinics. In other places, the patients are either asked to take the investigations outside or advised to visit the UPHC in the morning for the lab tests suggested by the specialist. Out of 473 patients interviewed, 43(9%) were prescribed drugs that were not available in polyclinic pharmacy, 71(15%) had accessed the polyclinic services after seeing advertisements for polyclinics and the IEC flex boards. The UPHC medical officers and the Urban Health Nurses referred 208(44%) and 85(18%) patients to the polyclinic from the morning OPDs. 29(81%) out of 36 polyclinics generated reports in the dashboard.

Output indicators: All the 473(100%) patients interviewed were satisfied with the polyclinic services, 27 patients (5.7%) were referred to a higher center for management. 14(39%) polyclinics functioned as per the fixed day fixed specialty mentioned in the polyclinic guidelines. Of the 29 reporting units, 25(86.2%) entered complete data in the application.

**Conclusions:** There is poor adherence to the polyclinic guidelines in maintaining the fixed day fixed specialty OPD services. Although the financial resources are adequately provided to meet the hiring charges of specialists, there is a need to ensure specialists' uniformity and availability on all days as per the guidelines. The non-availability of equipment like ECG and ultra-sonogram may be counterproductive for general physicians and OG specialists in managing cases. Drugs prescribed by the specialists need to be made available to ensure that patients don't spend on drugs out of pocket. There is a need to strengthen the laboratory services and information system in the polyclinic. Completeness of patient details in the web-based application will ensure quality data for analysis, review, and further planning of the polyclinics—utilization of polyclinics by urban low and associated factors to be further explored.

References

1. United Nations Department of Economic and Social Affairs. The World’s Cities in 2016. UN; 2016 (Statistical Papers - United Nations (Ser. A), Population and Vital Statistics Report). Available from: https://www.un-ilibrary.org/population-and-demography/the-world-s-cities-in-2016_8519891f-en

2. Slums in India: A statistical compendium, 2015. Available from http://nbo.nic.in/pdf/SLUMS_IN_INDIA_Slum_Compendium_2015_English.pdf

3. Health and Family Welfare Department, Demand no.19, Policy note 2018-19, Government of Tamil Nadu. Available from: https://cms.tn.gov.in/sites/default/files/documents/hfw_e_pn_2018_19.pdf

4. National Urban Health Mission, Framework for Implementation, Ministry of Health and Family Welfare, Government of India, May 2013. Available from: http://nhm.gov.in/images/pdf/NUHM/Implementation_Framework_NUHM.pdf

5. Handbook for Supporting the Development of Health System Guidance, Xavier Bosch‐Capblanch, On behalf of the project team, Swiss Tropical and Public Health Institute, July 2011. Available from: https://www.swisstph.ch/fileadmin/user_upload/WHOHSG_Handbook_v04.pdf

**Fig. 1 (abstract A12).**
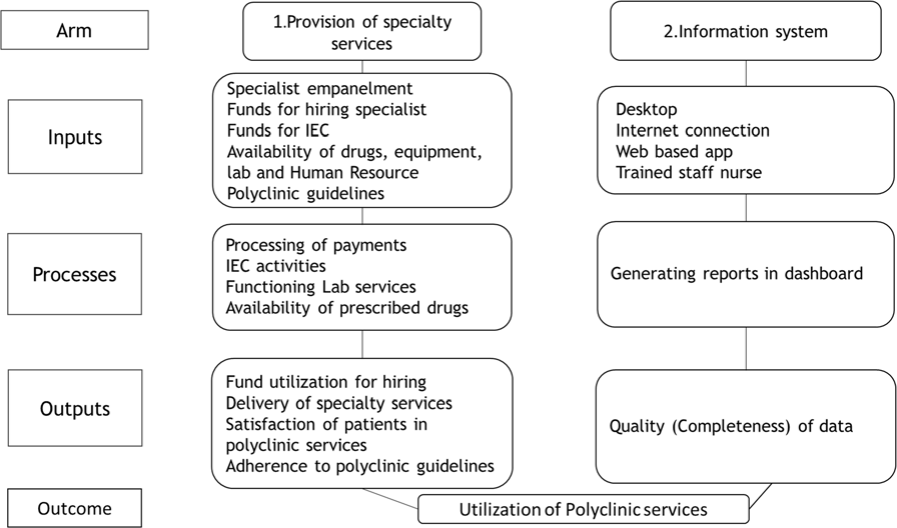
A logic model for evaluation of service provision in multi-specialty outpatient clinics at UPHCs, Chennai, Tamil Nadu, India, 2019

## A13 An Epidemiological Profile of Injury Patients Admitted in a Tertiary Care Hospital, New Delhi, India, 2015

### Naveen K. Rastogi^1^, Kapil Goel^2^, Tanu Jain^3^, Samir V. Sodha^4^, Chandra S. Aggarwal^1^, Srinivas Venkatesh^1^

#### ^1^National Centre for Disease Control, Ministry of Health and Family Welfare, New Delhi, India; ^2^Postgraduate Institute of Medical Education and Research, Chandigarh, India; ^3^Ministry of Health and Family Welfare, New Delhi, India; ^4^U.S. Centers for Disease Control and Prevention, New Delhi, India.

##### **Correspondence:** Kapil Goel (drkapil123@gmail.com)

**Background**

Globally, injuries have been neglected as part of the global health agenda despite being predictable and preventable. In 2015, 303 million disability-adjusted life years (DALYs) were lost from injuries [1]. Injuries also accounted for 4.9 million deaths, equating to 9% of all deaths [2]. In India in 2015, 23.5 million DALYs were lost from injuries [3]. Deaths from injuries were increased by 21%, from 342,309 in 2008 to 413,457 in 2015 in India [4, 5]. In 2014, injury surveillance was initiated by the National Injury Surveillance Centre (NISC) at a Tertiary Care Hospital in New Delhi to assess injury burden. The NISC enrolls cases of injury upon admission to a Tertiary Care Hospital in the casualty department. Data are collected by the trained data entry operators from the casualty department and hospital wards with a semi-structured questionnaire. We conducted a cross-sectional study of injury surveillance data to describe the epidemiology of injuries and contributing factors registered by NISC in 2015.

**Methods**

We conducted a cross-sectional descriptive study by retrieving NISC injury data from a Tertiary Care Hospital from 1 January 2015 to 31 December 2015. We extracted the 2015 injury data from the Tertiary Care Hospital to NISC in Excel Format for secondary data analysis. We cleaned data to exclude injuries with incomplete variables, and we removed all personal identifiers to maintain confidentiality. We analyzed data for Injury characteristics (demographics, mechanism and type of injury, intention of injury, site of injury, and place of occurrence), risk and safety features. We calculated counts, proportions, median age, and range for the injury characteristics using Epi Info 7 software (https://www.cdc.gov/epiinfo/index.html).

**Results**

There were 4,028 injuries and 15 (0.4%) injury-related deaths reported in 2015. Among people with injuries, 77% (3,085) were males. The median age was 28 years (range: 0–100), and 90% (3,630) were from urban areas. Ninety percent (3,645) of injuries were unintentional, and falls (40%, 1,607) and road traffic injuries (39%, 1,582) were most common (Table 1). Part of the body injured was reported in 3,260 (81%) of cases; head injuries accounted for 13% (419). Injuries were most frequently reported in February (769, 19%), March (643, 16%), and April (542, 13%).

Among road traffic injuries, two-wheel vehicles accounted for 66% (1,043), and 6% (60) of persons injured were not wearing a helmet. Among injuries from four-wheel and heavy vehicles, 30% (16) and 84% (145) of people were not using a seat belt, respectively. First aid was not received by 64% (1,019) of persons with road traffic injuries. Among persons with injuries due to falls, the median age was 25 years (range: 0–100), and they occurred most frequently at home (68%, 1,085). Assaults most commonly occurred on the road (40%, 111) and at home (39%, 107).

**Conclusions**

Based on analysis of NISC surveillance data, most injuries captured in 2015 were unintentional, related to falls and road traffic, and most frequently affected men. However, there are limitations to the data collected by NISC. Injury surveillance data was collected from only one hospital in New Delhi; therefore it cannot be generalized. In addition, NISC only captures people with injuries who were admitted to the casualty department or hospital. Thus, the data is not representative of all injuries because it only captures more serious injuries. We recommend expanding injury surveillance to other hospitals and clinic settings across India for more generalizable and representative injury surveillance data. Public health interventions to reduce injury-related morbidity and mortality should include expanding first aid and ambulance services in New Delhi and targeting road safety education to drivers to increase the use of helmets and seat belts.

**Acknowledgments**

We acknowledge and thank Dr. Anil Manaktala, Rupali Roy, and Dr. Shailja Sharma from Directorate General of Health Services, New Delhi; Dr. A. C. Dhariwal and Dr. Tanzin Dikid, from National Centre for Disease Control, New Delhi); Dr. A. K. Gadpayle and Dr. L.N. Gupta from Dr. RML Hospital) for their support in this study.

**References**

1. World Health Organization [Internet]. Global Health Estimates 2016,Disease Burden 2000–2016, DALY Estimates 2000–2016,for WHO Regions. [cited 2020 Jan 10]. Available fromhttps://www.who.int/healthinfo/global_burden_disease/estimates/en/

2. World Health Organization[Internet].Global Health Estimates 2016,Cause-Specific Mortality, 2000–2016, for WHO Regions. [cited 2020 10 January]. Available from: https://www.who.int/healthinfo/global_burden_disease/estimates/en/

3. World Health Organization [Internet].Global Health Estimates 2016, Disease Burden, 2000–2016, WHO Member States, 2015. [cited 2020 Jan10]. Available from: https://www.who.int/healthinfo/global_burden_disease/estimates/en/

4. National Crime Record Bureau Ministry of Home Affairs [Internet].Publications: Accidental deaths and suicides in India 2012 Report. Page 2. [cited 2020 12 January]. Available from: http://ncrb.gov.in

5. National Crime Record Bureau Ministry of Home Affairs [Internet].Publications: Accidental deaths and suicides in India 2015 Report. Page vii. [cited 2020 12 January]. Available from: http://ncrb.gov.in

**Table 1 (abstract A13).** Characteristics of patients with injuries admitted to the hospital in New Delhi, India in 2015

Characteristic	n	%
**All injuries(N=4028)**		
Male	3085	77
Urban	3630	90
Median age in years (range)	28*	0–100**
Death	15	<1
Unintentional	3645	90
Mechanism of injury		
Fall	1607	40
Road traffic	1582	39
Assault	276	7
Stab or cut	178	4
Burn	175	4
Others	210	5
Body part injured (n=3260)		
Head	419	13
Extremities	2366	73
Others	475	15
**Road traffic injuries (n=1582)**		
Male	1346	85
Median age in years (range)	25*	0–100**
Road use		
Two-wheel vehicle	1043	66
Pedestrian	263	17
Heavy vehicle	172	11
Four-wheel vehicle	53	3
No helmet, two-wheel vehicle	60	6
No seat belt, four-wheel vehicle	16	30
No seat belt, heavy vehicle	145	84
Did not receive first aid at scene	1019	64
Transported to health facility by private vehicle	591	37
**Falls (N=1607)**		
Male	1102	69
Urban	1461	91
Median age in years (range)	25*	0-100**
Death	1	<1
Unintentional	1531	95
Place of falls		
Home	1085	68
Road	253	16
Workplace	89	6
School or educational institute	69	4
Others	111	6
**Assault (N=276)**		
Male	220	80
Urban	253	92
Median age in years (range)	30*	1–82**
Place of assault		
Home	107	39
Road	111	40
Workplace	31	11
School or educational institute	2	1
Sport or athletic area	10	4
Others	15	5

*Median age in years; **Age range in years

## A14 An outbreak of malaria among the migrant construction workers traveled to a malaria-endemic area, Vanghur Village, Vellore District, Tamil Nadu, India, 2017

### Polani Rubeshkumar^1,2^, Manickam Ponnaiah^1^, KST Suresh^2^, Kolandaswamy Karumanagounder^2^

#### ^1^ICMR-National Institute of Epidemiology, Chennai, Tamil Nadu, India; ^2^Directorate of Public Health & Preventive Medicine, Tamil Nadu, India.

##### **Correspondence:** Polani Rubeshkumar (rubesh.pc@gmail.com)

**Background**

India contributes 76% of total malaria cases in the South-East Asia region [1-3]. Eleven percent of the Indian population lives in malaria-free areas [4]. Migration of population from low to high malaria transmission area makes them more susceptible to malaria [4]. Whereas migration from high to low malaria transmission area exposes malaria-free vectors to disease [4]. The cycle of reintroduction threatens the malaria control and elimination strategy of India [4].

Vellore District of Tamil Nadu State has been remaining malaria-free since 2012. In September 2017, Vellore District Surveillance Unit reported a cluster of Malaria cases in Vanghur village. We investigated the cluster to describe the cases by time, place, and person, identify the reason for occurrence, and recommend control measures.

**Methods**

We reviewed the last three years surveillance data of Vellore District to verify the malaria cases and changes in the surveillance system. We adopted National Vector-Borne Disease Control Programme (NVDCP) case definition. We defined a probable case of malaria as fever and chills/rigors/nausea/vomiting.^5^ Confirmed malaria case as a probable case with detection of malarial parasite in blood smear microscopy in any resident of Vanghur village, September-October 2017.

We actively searched for cases house-to-house and followed by stimulated passive surveillance through a medical camp. We collected data on demographics, clinical symptoms, and travel history. We computed proportions for the clinical symptoms reported by the case-patients. We calculated the attack rate of malaria by age and gender using the population of the village as a denominator. We drew epicurve by the time of onset of fever. We plotted the malaria cases on a spot map.

A team of entomologists conducted an entomological survey to identify the vectors. They enumerated breeding sites within a 500 m radius of the village. They collected larval samples by pipetting and netting techniques. They caught the adult mosquitoes indoors and outdoors by resting collection technique day and night. They identified species of mosquitoes using magnifying lenses and microscopy.

We interviewed key informants like case-patients and residents of the village about the illness to generate the hypothesis. We collected capillary blood from all the probable cases for smear microscopy.

**Results**

The population of the Vanghur village was 2774 with 1390 (50%) Males. We identified 311 probable malaria cases; among them, 56% (175/311) were male. The median age of the probable cases was 40 years (Range: 2-80 years). The attack rate was 112 per thousand (311/2774). The attack rate was higher among males [126 per 1000, (175/1390)] than females [98 per 1000, (136/1384)]. The attack rate was higher among the age group 25-39 years (156 per 1000 population, (110/705)] (Table 1).

Case-patients reported fever (100%), headache (100%), fatigue (94%), myalgia (88%), chills (78%), nausea (72%), vomiting (67%), rigors (21%) and giddiness (17%).

Out of 311 cases, seven cases were positive for *P.vivax*. The slide positivity rate was 2% (7/311). The attack rate of malaria was 3 per 1000 population (7/2774). The median age of the malaria case-patients was 39 years (Range: 18-70), and all were male. All seven cases belonged to the age group 15-59 years (Table 1).

The seven confirmed malaria cases reported the onset of symptoms between September 15, 2017 and September 28, 2017. The spot map shown no specific pattern or clustering of malaria confirmed cases residence. The residence of seven confirmed malaria cases was scattered across the Vanghur village.

The entomological team identified 62 mosquito-breeding sites within a 500 meters radius of Vanghur village. The larvae collected from the breeding sites and adult mosquitoes collected indoors and outdoors were identified as *Aedes* and *Culex* species. The entomological investigations were failed to identify *Anopheles* mosquitoes in the Vanghur village.

During the interview of key informants, we found that all the seven confirmed malaria case-patients were mason by occupation. They were working and staying at the same construction site in a malaria-endemic area. None of them reported the usage of bed nets or mosquito repellents. They reported that they developed symptoms during their stay at the construction site. As they were unaffordable for the treatment in the private healthcare facilities, they returned to their village with illness and sought Government Primary Health Center for treatment. We generated a hypothesis as staying or working in a malaria-endemic area could have caused the outbreak.

The probable cases were treated with anti-pyretic and supportive management. The confirmed malaria cases were treated with chloroquine, primaquine, anti-pyretic, and anti-emetics. We followed the cases till the complete recovery. We cross-notified the cases to the concerned public health authorities of the malaria-endemic area.

We could not estimate the strength of association between staying or working in a malaria-endemic area and the occurrence of malaria.

**Conclusions:**

The malaria cases in Vanghur village were imported from the malaria-endemic area. We recommended periodic entomological surveys in the Vanghur village and promoted personal protective measures for those traveling to the malaria-endemic area.

**References**

1. Ghosh SK, Rahi M. Malaria elimination in India—The way forward. Journal of Vector Borne Diseases. 2019 Jan 1;56(1):32.

2. World Health Organization. World malaria report 2019. Geneva: World Health Organization; 2019. Licence: CC BY-NC-SA 3.0 IGO [https://www.who.int/news-room/feature-stories/detail/world-malaria-report-2019]. Accessed 26 Aug 2020.

3. National Institute of Malaria Research. India. Estimation of true malaria burden in India Available from: [http://www.mrcindia.org/MRC_profile/profile2/Estimation%20of%20true%20malaria%20burden%20in%20India.pdf]. Accessed 26 Aug 2020.

4. Ministry of Health & Family Welfare, Government of India. National strategic plan for malaria elimination in India 2017-2022. Available from: [https://nvbdcp.gov.in/Doc/Strategic-Action-Plan-Malaria-2012-17-Co.pdf]. Accessed 26 Aug 2020.

5. Ministry of Health & Family Welfare, Govt. of India. 2013. Directorate of National Vector borne Disease Control Programme, Ministry of Health & Family Welfare, Government of India. Diagnosis and Treatment of Malaria 2013. [https://nvbdcp.gov.in/Doc/Diagnosis-Treatment-Malaria-2013.pdf]. Accessed 26 Aug 2020.

**Table 1 (abstract A14).** Malaria cases by age and sex, Vanghur village, Vellore District, Tamil Nadu, India, 2017

Characteristics		Population at risk	n (Attack rate per 1000)	
			Probable cases	Confirmed cases
Age (years)	<5	214	8 (37)	0 (0)
	5-14	451	56 (124)	0 (0)
	15-24	497	24 (48)	1 (2)
	25-39	705	110 (156)	4 (6)
	40-59	619	75 (121)	2 (3)
	≥60	289	38 (131)	0 (0)
Sex	Male	1390	175 (126)	7 (5)
	Female	1384	136 (98)	0 (0)
Total		2774	311 (112)	7 (3)

## A15 Influenza A (H1N1) outbreak in Irular colony, New Gummidipoondi village, Tiruvallur health unit district, Tamil Nadu, India, 2017

### Saraswathi VS^1^, Parasuraman Ganeshkumar^2^, Tarun Bhatnagar^2^, Manoj Murhekar^2^, Manickam Ponnaiah^2^, Prabakaran J^1^

#### ^1^Department of Public Health and Preventive Medicine, Govt. of Tamil Nadu; ^2^ICMR - National Institute of Epidemiology, Chennai

##### **Correspondence:** Parasuraman Ganeshkumar (ganeshkumardr@gmail.com)

**Background:**

H1N1 Influenza virus, a respiratory pathogen, causes severe respiratory illness, which may result in high mortality. Being its person to person transmission nature caused widespread pandemics and outbreak potential in crowded areas [1]. First influenza A H1N1 case in India was reported from Hyderabad on May 16, 2009 [2]. Prompt detection, the early response of outbreaks are critical. In this manuscript, we discussed an influenza outbreak in a rural area of Tamil Nadu. In January 2017, an Irular colony of New Gummidipoondi village of Tiruvallur district near the Andhra Pradesh border reported high number of cases of fever and cough of unknown cause. We investigated these high reported ILI (influenza-like illness) cases to confirm the outbreak, identify the agent, source, and propose control measures.

**Methods**

Irular colony is a slum near the banks of a lake in New Gummidipoondi village. It belongs to New Gummidipoondi subcenter of Kavarapettai primary health centre of Gummidipoondi block of Tiruvallur health unit district. The population of Irular colony was 57. We defined a suspected case of acute respiratory infection with measured fever of more than or equal to 38°C and cough in Irular colony, New Gummidipoondi village from December 31, 2016. We searched for suspected cases in the outbreak area. We reviewed the data on the previous occurrence of fever cases and found that not more than 5 cases per month were reported during the same period in the last three years in the same village. There was no migration of population in the recent past and no change in the surveillance system. We conducted door to door active surveillance to search new cases, and we collected data on demographic details, symptoms onset, type, associated co-morbidities, travel, and hospitalization details. We conducted a matched case-control study to identify the source of infection. For each identified case by definition, we selected one neighborhood-matched control. We obtained specimen from the case patients and sent for laboratory confirmation of H1N1. We also conducted an environmental investigation.

**Results**

The first case who was a pregnant woman was reported on December 31, 2016, at the nearest health facility and infection peaked with 19 cases on January 5, 2017. We identified 36 cases with an overall attack rate of 63% based on an active case search. There were four deaths reported in the study population with a case fatality rate of 11%. Thirty-five out of 36 cases were hospitalized. The median days of hospitalization of 35 cases were 9 (IQR 7-10.5). The median duration of hospitalization was one day among died and nine days (IQR 8-10) among recovered. Overall secondary attack rate was 62%.

Attack rate was highest in the age group 0-5 years (13/13,100%), followed by 6-15 years (10/15, 67%). Of the four who died, three belong to the age group 16-30 years, and one belongs to 45-60. Attack rate is more among male(AR-20/30,67%) than female(AR 16/27 59%). Influenza A (H1N1) virus was identified from throat swabs of 11 out of 33 specimens in Real-time Reverse transcriptase-Polymerase chain reaction(RT- PCR). Of the 36 probable cases, we could identify 21 neighborhood matched controls. Cases were ten times (Matched-Odds ratio:10.94; 95%CI 1.2-101.4) odds of association of contact with a fever case compared to neighborhood controls. Travel history was 2.5 (Matched-odds ratio:2.5; 95% CI 0.26-23.9) odds of association with cases than controls. We found no pig breeding around the houses during the environmental investigation; the study population lived in a small kutcha house and was overcrowded. Hence the possibility of spread of infection by close contact was very high.

**Conclusions**:

Evidence suggests that close person-to-person contact has been the primary cause of the spread of the virus. Health education regarding case isolation and avoiding close contact with fever cases might help prevent disease transmission.Information Education Communication(IEC) activities about the availability of the services were provided. Findings were shared with all the stakeholders and local health authorities.

**References**

1. Arbat S, Dave M, Niranjane V, Rahman I, Arbat A. Analyzing the clinical profile of swine flu/influenza A H1N1 infection in central India: a retrospective study. Virusdisease. 2017 Mar 1;28(1):33-8.

2. Choudhry A, Singh S, Khare S, Rai A, Rawat DS, Aggarwal RK, Chauhan LS. Emergence of pandemic 2009 influenza A H1N1, India. The Indian journal of medical research. 2012 Apr;135(4):534.

**Table 1 (abstract A15).** Frequency of exposure among cases and controls in H1N1 outbreak, New Gummidipoondi village, Tiruvallur district, Tamil Nadu, India, 2017

Factors	Cases (N=35)	Controls (N=21)	Odds ratio	95% CI
Contact with fever cases	35	16	10.94	1.2 to 101.4
Travel history	4	1	2.5	0.26 to 23.9
Tamiflu tablet issued	35	21	Undefined	0 to 32.5

## A16 Contact tracing during Nipah outbreak, Ernakulam District, Kerala, India 2019

### Hari Sankar^1^, Sugunan AP^2^, Tarun Bhatnagar^2^, Ganesh Kumar^2^, Manoj Murhekar^2^, Saritha Ragini Lohithakshan^3^, Sreedevi^3^

#### ^1^ICMR National Institute of Epidemiology, Chennai, India; ^2^Indian Council of Medical Research – National Institute of Epidemiology, Chennai, India; ^3^Kerala Health Services, Thiruvananthapuram, Kerala, India

##### **Correspondence:** Hari Sankar (shankarhar@gmail.com)

**Introduction**

Nipah virus (NiV) is an emerging zoonotic virus with high fatality. NiV is a member of the Henipavirus genus of the paramyxoviridae family and is listed as a highly infectious pathogen by the World Health Organization requiring urgent R & D[1]. Fruits bats of the Pteropus genus are the natural host of the virus [2]. NiV outbreaks have been reported from Malaysia, India, and Bangladesh [3]. In the Malaysian outbreak, pigs were intermediate hosts, whereas, in outbreaks in Bangladesh, the consumption of date palm sap contaminated with infected fruit bat was the main-spill over the virus's route [3]. Following infection in humans, subsequent transmission to close contacts, including health workers, occurred through the person-to-person routes during Bangladesh and Indian outbreaks. Close physical contact, exposure to body fluids, particularly respiratory secretions, or contaminated surfaces have been proposed as key transmission routes[4].

During the 2018 NiV outbreak in Kozhikode district, Kerala, more than 2600 contacts of laboratory-confirmed patients were identified and kept under quarantine [5]. These contacts were monitored daily for the development of febrile illness. A serosurvey conducted among the close contacts (family contacts and health care workers) of laboratory-confirmed NiV cases during the outbreak in Kerala in 2018 indicated that only 1.08 % had evidence of subclinical infection[3].

In May 2019, one resident of Ernakulam district in Kerala tested positive for NiV. We implemented the contact tracing protocol revised based on the 2018 NiV outbreak's experience to early detect suspected NiV patients.

**Methods**

The District Medical Officer activated contact tracing teams with one contact tracer for 1–3 households. Junior Health Nurses along with ASHAs listed people as contacts if they had (a) touched body fluids of a case, (b) direct physical contact with the case, (c) slept or ate in the same household as the case, (d) manipulated the clothing of or shared linens with the case or (e) a close interaction with a case that did not involve physical contact. Based on this information, all contacts were categorized as high and low-risk contacts. Individuals with (a) a history of contact with the bodily fluids of the affected patient and/or (b) who were in close proximity with laboratory-confirmed patients for >=12 hrs were categorized as ‘high-risk contacts’ and others into the low-risk category. All the high-risk contacts were monitored for 21 days post-exposure for the development of fever. High-risk contacts who developed fever were isolated and their blood specimens were collected for laboratory confirmation. Low-risk contacts were asked to self-report if they develop a fever. Contacts developing febrile illness were hospitalized in an isolation unit, and their oropharyngeal swabs were collected in a viral transport medium for NiV real-time RT-PCR.

**Results**

We identified 318 contacts, 52 (16%) of whom were high-risk contacts. Among high-risk contacts, 35 (67%) were healthcare workers, 6 (12%) were family members, and 11 (21%) were community contacts. Forty-eight percent of the high-risk contacts were exposed to the Nipah case in a private healthcare facility where he was treated. Six high-risk contacts developed fever and were isolated in a public health facility. All the isolated contacts were considered suspects and tested negative for NiV on RT-PCR. No contacts were lost during follow-up—none of the low-risk contacts self-reported fever.

**Conclusions**

Contact tracing was a critical intervention in quarantining the contacts and early detection of suspected cases, thereby preventing further transmission. The categorization of contacts based on risk assessment helped us focus on contacts with higher risk and suspected patients.

**References**

1. Calisher, Charles H., James E. Childs, Hume E. Field, Kathryn V. Holmes, and Tony Schountz. “Bats: Important Reservoir Hosts of Emerging Viruses.” Clinical Microbiology Reviews 19, no. 3 (July 2006): 531–45. 10.1128/CMR.00017-06.

2. Kumar, C.P. Girish, Attayur P. Sugunan, Pragya Yadav, Karishma Krishna Kurup, Renjith Aarathee, Ponnaiah Manickam, Tarun Bhatnagar, et al. “Infections among Contacts of Patients with Nipah Virus, India.” Emerging Infectious Diseases 25, no. 5 (May 2019): 1007–10. 10.3201/eid2505.181352.

3. Luby, Stephen P., Emily S. Gurley, and M. Jahangir Hossain. TRANSMISSION OF HUMAN INFECTION WITH NIPAH VIRUS. Improving Food Safety Through a One Health Approach: Workshop Summary. National Academies Press (US), 2012. https://www.ncbi.nlm.nih.gov/books/NBK114486/.

4. Thomas, Bina, Priya Chandran, M. P. Lilabi, Biju George, C. P. Sivakumar, V. K. Jayadev, V. Bindu, et al. “Nipah Virus Infection in Kozhikode, Kerala, South India, in 2018:

5. Epidemiology of an Outbreak of an Emerging Disease.” Indian Journal of Community Medicine : Official Publication of Indian Association of Preventive & Social Medicine 44, no. 4 (2019): 383–87. 10.4103/ijcm.IJCM_198_19.

## A17 An outbreak investigation of Dengue and Chikungunya in Thunikinoothala Thanda, Nalgonda District, Telangana, March 2019

### Seema Tabassum^1^, Sushma Choudhary^2^, Sailaja Bitrugunta^1^_,_ Manikandanesan Sakthivel^1^

#### ^1^ICMR - National Institute of Epidemiology, Chennai, India; ^2^South Asia Field Epidemiology and Technology Network, India

##### **Correspondence:** Seema Tabassum (samarahmustafa786@gmail.com)

**Introduction:**

World health organisation estimate 390 million dengue virus infections per year (95% credible interval 284–528 million), of which 96 million (67–136 million) manifest clinically [1]. India reported 101,192 cases, 172 deaths and 100 outbreaks in 2018 [2,3]. Telangana reported 4592 cases, two deaths, and four outbreaks in 2018 [2,3]. On 6 March 2019 Nalgonda district surveillance unit of Integrated Disease Surveillance Programme (IDSP) reported clustering of fever cases (47) in Thunikinoothala Thanda village for last two weeks. We initiated an investigation to confirm the outbreak, describe the outbreak in terms of time, place, person, and to implement evidence-based measures to control and prevent the outbreak.

**Methods:**

We confirmed the existence of the outbreak by comparing the IDSP data of week 9 and 10 with the same period reported by the respective reporting units in the last three years. We interviewed six case-patients identified by the district surveillance unit and prepared a case definition to search for cases. Suspect case-patient was defined as fever in a resident of Thunikinoothala Thanda, from 1 February to 21 March 2019. Probable case-patient was defined as fever with myalgia and any of the following symptoms (rash, joint pains, cold, vomiting) in a resident of Thunikinoothala Thanda, from 1 February 2019 to 21 March 2019. A suspect or probable case-patient positive for Dengue IgM or Chikungunya IgM antibodies was considered as confirmed case-patient. We searched for more case-patients by active surveillance. We formed four teams with two members in each team three accredited social health activists (ASHA), two auxiliary nurse midwife (ANM), two Anganwadi workers (AWW) one health supervisor. All were trained for case definition and questionnaire. We collected household data in a survey form, and the questionnaire was used to interview only the case-patients. In the questionnaire, we captured data about demographics, travel history in the last one month, date of onset of fever and duration, symptoms, treatment history and outcome of all suspected cases. In households, we captured data about the age and sex of the population. Data was collected on 7^th^ and 8^th^ of March 2019. We established a team with local Anganwadi supervisor to search for new cases till 15 March 2019 by the active house-to-house survey. Data were entered into MS Excel. We analysed in terms of time (epidemic curve), place (spot map) and person (attack rate by age and sex). Proportions were calculated for symptoms, hospitalisation. We calculated the median duration of fever and hospital stay. We collected blood samples from the suspect and probable case-patients whose date of onset of illness was > 5 days at the time of collection. We prepared thick and thin smears and applied the rapid test to identify the malarial parasite, IgM antibodies for dengue and chikungunya by Elisa method. As part of the environmental survey, we did house-to-house surveys for the number of containers, coolers, tyres, and disposable plastic things with storage and water collection. Observed for larvae in them. We calculated “House index”, using the formula, (number of houses positive for larvae/total no of houses surveyed) x 100. We calculated “Container index” as the number of containers positive for larvae divided by the number of containers surveyed and multiplying by 100. We also determined “Breteau index” using the formula (number of containers positive for larvae/number of houses surveyed) x100.

**Results:**

We surveyed 226 houses in Thunikinoothala Thanda and found case-patients in 63 houses. We identified 72 suspected case-patients, among whom 49 (68%) were probable case-patients. The median age of suspected case-patients was 35 years (range: 9- 60) with 27(55%) males. The first case-patient was reported on 2 February 2019, peaked on 19 March 2019 (16 cases), no case-patient reported after 15 March 2019 (Figure.1). The overall attack rate was 7%, the highest (10%) in >50 age-group and lowest (3%) in 0-10 age group. The attack rate was 8% in males and 7% in females. All probable case-patients had fever and myalgia (100%, 49/49), 38 (78%) had joint pain, 9 (18%) had rash, 7 (14%) had vomiting, and 3 (6%) had cold. The median (IQR) duration of fever was 7 (5-9) days. Among 49 probable case-patients, 25 (51%) took treatment in private hospitals, and 10 (20%) were treated as “in-patients”. The median (IQR) duration of hospital stay was 5.5 (4-7) days. All blood smears were negative for the malarial parasite. Out of 12 blood samples, 11 (92%) were positive for dengue IgM antibodies and 3 (25%) for chikungunya. House Index was 18, and the Container and Breteau Indices were 13 and 20 respectively. Borewells used to be the source of water previously, but during January 2019, nearly 80% of borewells dried. Hence, the residents of the village started using municipal water. Water was supplied once in four to six days, leading to water storage in open cement tanks and containers. The water tanks in 16 houses were left open and had mosquito breeding.

**Conclusions:**

A laboratory-confirmed Dengue and Chikungunya outbreak was confirmed in Thunikinoothala Thanda village, with the highest attack rate among >50 years age group. The outbreak was likely due to high vector indices in the village. We recommended to empty vessels, clean, scrub and dry at least weekly, and cover all vessels/containers and tanks with a fitting lid. We also recommended emptying all water containers before leaving the house for more than a week.

**References**

1. World Health Organization. Dengue and severe dengue [Internet]. Available from: https://www.who.int/news-room/fact-sheets/detail/dengue-and-severe-dengue. [Accessed 2021 Feb 8]

2. Dengue: National Vector Borne Disease Control Programme (NVBDCP) [Internet]. Available from: https://nvbdcp.gov.in/index1.php?lang=1&level=1&sublinkid=5776&lid=3690. [Accessed 2021 Feb 8]

3. Home: Integrated Disease Surveillance Programme (IDSP) [Internet]. Available from: https://idsp.nic.in/. [Accessed 2021 Feb 8].

**Fig. 1 (abstract A17).**
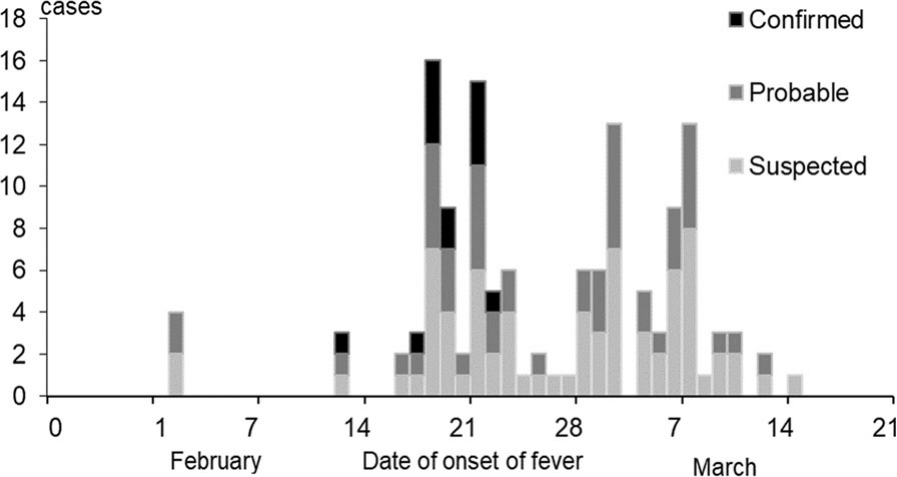
Distribution of Dengue/Chikungunya suspect case-patients by date of onset of fever in Thunikinoothala Thanda, District Nalgonda, Telangana, March 2019 (N=72)

## A18 Universal Health Coverage Information Technology (UHC-IT) Platform, an approach to have a population as the denominator to establish a digital cohort of the State, Tamil Nadu, 2019

### Viduthalai virumbi Balagurusamy, Beela Rajesh, Kolandaswamy Karumanagounder, Darez Ahamed, Senthil Raj K

#### Department of Health and Family Welfare, Government of Tamil Nadu, India

##### **Correspondence:** Viduthalai virumbi Balagurusamy (v.virumbi@gov.in)

**Introduction**

Information exchange from field staff to State level evolved over 300 years from messenger, paper, telegram, email, portal and all available mean. Health applications in the State are in silos of patient medical history and fragmented Information Technology (IT) systems, limiting the health provider to access the available patient information. Due to lack of learning health care system that can identify and respond to real-time needs at the individual and or population level, inadequate or non-existent monitoring of treatment and feedback insights, the State committed to developing a digital platform, where all the patient information would converge.

**Method**

Master Registry was conceptualized to develop a common list of address, health facilities, service area, family folders, masters, and users accessed by all health applications (Figure 1). The district health authorities listed 203,593 Streets of urban and rural areas mapped to villages, blocks, and districts to establish address hierarchy. 13,640 Health Sub Centres (HSC) were listed and mapped with corresponding Primary health Centres, Block Health Centres, Health Unit Districts to establish the organizational hierarchy of the State. HSC catering to each street was mapped under the Communicable Disease Surveillance Portal (CDSP) project, referred to as Service Area Mapping. About 66 million Public Distribution System's (PDS) digital families had been mapped to this service area mapping at 15 levels using 'Join attributes by location' plugin of QGIS. Demographic details of individuals were made available in both English and Local Language Tamil. Datasets were assigned with unique identifiers. User credentials for each health facility were assigned with attributes like institution type, the domicile of the facility. Lab tests, Provisional diagnosis mapped with services, drugs with formulations and strength were tabulated as 'Master of Masters'. Unique identifiers were used to create alias tables for linking with health and related datasets. The logic for transaction identifiers and modifications of master registry datasets were framed. State Programme Management Unit (SPMU) drafted the Health IT Standards for enabling the health application to have commonalities for convergence. In compliance with IT standards, the Master Registry (MR) was hosted in Amazon Web Services (AWS) Cloud Server with elastic search and managed database features. Master Registry database was shared as Application Programme Interfaces (API) with CDSP and UHC applications. Single-layer GIS map of Master Registry with continuous geographical locations was hosted by Tamil Nadu Geographical Information Systems (TNGIS). To reduce the dependency on IT staff, a frontend software application was developed for updating the datasets of Master Registry. Cascading trainings on the usage of CDSP and UHC applications were given to all users at block level by master trainers. State Health Department incurred 4474 Thousand Indian Rupees to establish this Platform under Universal Health Coverage (UHC) programme.

**Results**

A list of 66 million individuals was accessible to the health staff placed at Health Sub Centres while providing outpatient services through the progressive web application. The line list of outpatient services was downloaded with organization hierarchy and identifiers for applying analytical tools. The platform could sustain the service with two-times increase of users (787 in May to 1,650 in October 2019) and 3.7 times increase of footfalls (43,379 in May to 160,291 in October 2019). Ten outpatients per day at Health Sub Centre (HSC) and eight outpatients at Primary Health Centre (PHC) were entered as line list in the portal during October 2019. About 27% (104/722) of the outpatient entries were made by selecting the individuals made available from Master Registry during May to October 2019. A total of 149 lab-confirmed fever cases per day were reported from May to October 2019 through CDSP portal using Master Registry. The end users required enhancements such as offline mobile application for data collection and searching the walk-ins within 5 seconds. Auto-generation of key administrative indicators and communication of lab results and reminders through SMS to beneficiaries were the health authorities' requirements. Dataset collected under CDSP, and UHC applications were under process for integration with national counterpart applications.

**Conclusion**

Master Registry, a common list shared to health applications enabled convergence of health data at differential levels. Creating an Open Neutral Shared Infrastructure for hosting the Master Registry reduced the cost of establishing healthcare information technology platform to create newer applications and analyse the datasets. User Acceptance of digital transformation features to increase the platform response (offline, quick search and synchronization) and monetization components (payment gateways) have to be explored further. Health determinants, disease registries and user management tools linked to the master registry will help understand population outcomes for improved decision making. Designing the Master Registry at the State/National Scale and placing as core in enterprise architecture has given the leverage to integrating data generated under various health applications.

**Fig. 1 (abstract A18).**
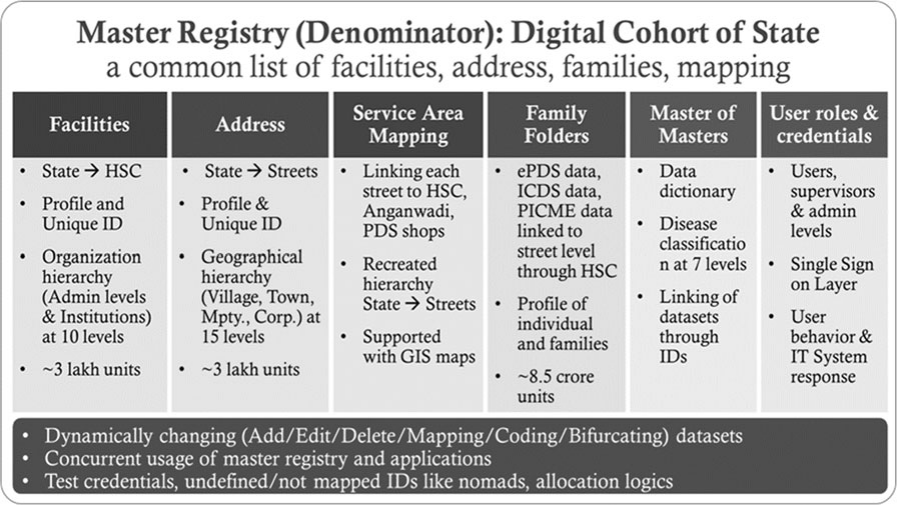
Building blocks of Master Registry Application

## A19 Barriers towards timely reporting of and response to outbreaks in West Bengal, India 2015-16

### Biswajit Dey^1^, Tarun Bhatnagar^2^, Manickam Ponnaiah^2^, Sharmistha Mitra^1^ Prabhdeep Kaur^2^

#### ^1^Department of Health & Family Welfare, Government of West Bengal, India; ^2^ICMR-National Institute of Epidemiology, Chennai

##### **Correspondence:** Biswajit Dey (deybisu@gmail.com)

**Background**

Globally the timeliness of outbreak detection has improved by 7.3% per year from 1996 to 2009 [1]. The median time from outbreak start to outbreak discovery was 13.5 days in 2009 [1]. These delays indicated some gaps in the existing surveillance system, which need to be identified and rectified to prevent widespread outbreaks in the future. We have limited data regarding delays in reporting in West Bengal. However, various district and state-level reviews documented the delayed reporting and response for several outbreaks. Our objective was to identify the potential barriers to timely reporting and responding to outbreaks in West Bengal, India 2015-16.

**Methods**

We conducted a cross-sectional, mixed-methods study. We divided the districts of West Bengal into three strata based on the number of outbreaks reported from 2012 to 2014 – high, low, medium. From each stratum, we randomly selected one district – Nadia district from the high stratum, Howrah from the low stratum, and Hooghly district from the middle stratum. From each district, we randomly selected three blocks. We surveyed Health Supervisors (HS), Primary Health Centre Medical Officers (PHC MO), and block-level workers in the selected blocks. We included all the district-level workers in the selected districts and State level officials. We selected Auxiliary Nurse and Midwives (ANM) and Health Assistant-Males (HAM) by simple random sampling from the selected blocks. We selected 8 – 10 Accredited Social Health Activist (ASHA) in the chosen districts by convenience. We did Focus Group Discussions with the ASHAs in each district and interviewed all the other workers using a semi-structured questionnaire. We analyzed qualitative data and categorized it according to major themes. We constructed a conceptual framework with the emerging themes related to delay in reporting and responding to outbreaks. We used five points Likert scale to quantify responses related to perceptions and beliefs related to outbreak preparedness. The NIE's Institutional Ethics Committee approved the study.

**Results**

We interviewed 167 ANMs/HAMs, 65 HSs (combined as 232 field level workers from now on), 29 PHC & Block level workers, 11 District level workers, and all the four State level workers. Out of the 232 field level workers, 192 participated in outbreaks. Among them, 92 (50%) had always received reports on time. Out of 27 PHC and block-level workers, 18 (77.5%) received reports late. Nine (81.8%) of the 11 district-level workers and three out of four state-level workers reported receiving delayed reports. Out of the 232 field level workers, only 10 (4.3%) had good knowledge about outbreak detection and response. None of the 29 PHC and block-level workers and five (33.3%) of the 15 district-level workers had good understanding of outbreak investigation and response. Overall, 143 (62%) of the field level,19 (66%) of the PHC and block level, and 11 (73%) of the District level workers had received IDSP induction training. ASHAs had a working knowledge of outbreaks, but their training was inadequate. After receiving reports of outbreaks, 139 (83.2%) of the 167 ANMs/HAMs, 35 (53.8%) of the 65 HSs, 5 (45.5%) of the 11 PHC MOs, 8 (44.4%) of the 18 block-level workers, and one (9.1%) of the 11 district-level workers did the recommended actions. The majority (8/11) of the district-level workers gave seven different compositions of the District Rapid Response Team (RRT), and none of that matched with the composition stated by the State Surveillance Officer. Overall (4/5), BMOs reported functional RRTs in their blocks, but all of them gave different formations. The main reasons behind the delay in reporting were vacancies of ASHAs and Health Assistant (Male)s, inadequate supervision by field level workers and PHC MOs, poor interaction between villagers and health workers, inadequate training of ASHAs and block-level workers, and occurrence of the outbreak at inaccessible place or time (Figure 1). Causes of the delayed response included district-level workers' engagement in multiple programmes, an inadequate workforce, delayed reporting from the field, and the district authorities' reluctance (Figure 1). Only 65 (24%) were confident about safety in their work role. Nearly all (96%) workers felt that psychological support would be necessary during the outbreak, and 191 (69%) felt that they would get psychological support from their superiors. Three-fourths of the staff [193 (70%)] said getting incentives or recognition for work done during an outbreak would be important, but only 41 (15%) believed that they would receive any recognition.

**Conclusions**

The main barriers to timely reporting of outbreaks were insufficient human resources, lack of knowledge, and inadequate training. Poor understanding of roles led to delay in reporting and responding to outbreaks. Other reasons for delayed response were the engagement of District lever workers in multiple programmes and delayed reporting of epidemics from the field. Lack of motivation and recognition were barriers to satisfactory performance. We recommend adequate workforce, guidelines regarding roles and responsibilities, practical hands-on training, simplifying the field-based reporting systems, engagement with the communities, and incentives to field workers for early reporting. We can replicate the lessons from other Low-Middle income countries to improve response to outbreaks [2]. At the district level, establishing the Emergency Operations Center (EOC), strengthening the field epi training, and forming RRT with clearly defined structure and roles will improve outbreak response.

**References**

1. Chan EH, Brewer TF, Madoff LC, Pollack MP, Sonricker AL, Keller M, Freifeld CC, Blench M, Mawudeku A, Brownstein JS. Global capacity for emerging infectious disease detection. Proceedings of the National Academy of Sciences. 2010 Dec 14;107(50):21701-6.

2. Oleribe OO, Crossey MM, Taylor-Robinson SD. Nigerian response to the 2014 Ebola viral disease outbreak: lessons and cautions. The Pan African medical journal. 2015;22(Suppl 1).

**Fig. 1 (abstract A19).**
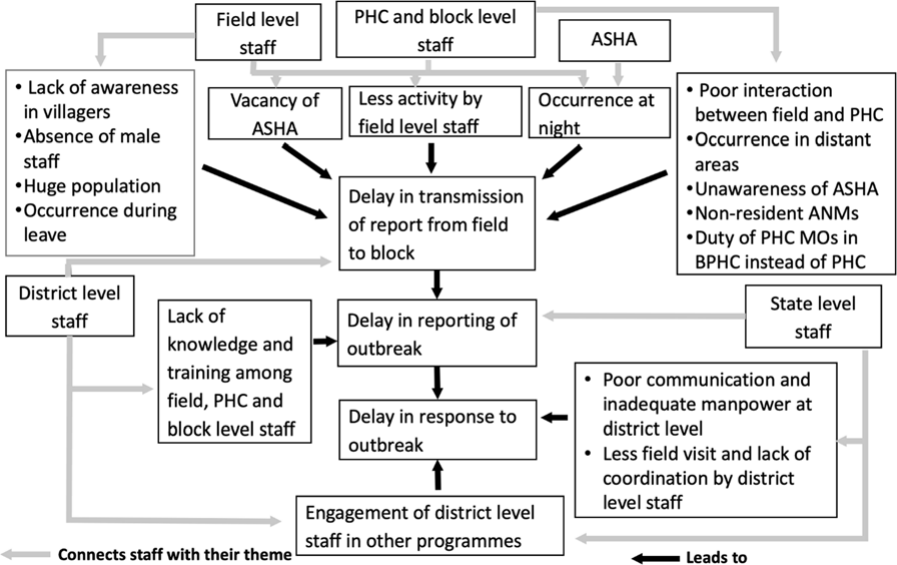
Pathway of delay in reporting and responding to outbreaks as narrated by public health workers in West Bengal, 2015-16

## A20 Establishing Media Surveillance to monitor post-flood Public Health Response in Kerala, India, August- September 2018

### Rontgen Saigal^1^, Parasuraman Ganeshkumar^2^, Lakshmi GG^3^

#### ^1^FETP NCD Fellowship scholar, ICMR-National Institute of Epidemiology, Chennai; ^2^Scientist D, ICMR National Institute of Epidemiology, Chennai, India; ^3^Medical Officer, Kerala Health Services, Thiruvananthapuram, Kerala, India

##### **Correspondence:** Parasuraman Ganeshkumar (ganeshkumardr@gmail.com)

**Background**

Floods account for about 40% of all-natural disasters and cause about half of all deaths worldwide [1]. Kerala, the southernmost state in India, faced an unprecedented rainfall in August 2018, resulting in extensive floods and landslides. Catastrophic damage to life and property occurred in 13 out of 14 districts of the state and claimed 483 lives. The most recorded public health issue during the flood and related calamities was the spread of infectious diseases and the victims' poor mental health [2]. Surveillance of the same during the post-disaster period helps the health system pick up emerging outbreaks quickly and report the same to the authorities [3].

Kerala's public health surveillance system is implemented through the Integrated Disease Surveillance Project (IDSP). It gets monitored under the State Public Health wing at the Directorate of Health Services. However, during the flood and the immediate post-disaster period, the routine surveillance systems were not fully functional owing to the damage to the hospital infrastructure and telecommunication systems. This warranted the use of alternate strategies fr enhancing the failed system. Media Surveillance, a type of surveillance using events reported via radio, television, newspaper, and the internet, had been used effectively by countries like Peru, Nepal, Bangladesh to detect outbreaks. This method is sensitive, low cost and highly time-saving, and sustainable [4]. This paper talks about how we established media surveillance to identify the health-related events/news from various enlisted media sources. We analyzed the same to arrive at decisions to aid epidemiological surveillance, to predict and prevent any potential disease outbreaks in the disaster control phase.

**Methods**

The Media Surveillance center was established at the state headquarters on August 20, 2018. This center worked 24×7 and had 24 hour Internet Connectivity, Television, Radio, and various Local and National Newspapers. The center consisted of two Medical Officers, ten staff for technical support, one nodal person from all districts for local news reporting, and a group of volunteers for helping out with media scanning. For the ease of data capture and documentation, the staff and volunteers were assigned into four teams: WhatsApp Helpline Team, the Television team, the Social Media team, and the Newspaper team. We developed Standard Operating Procedures (SOP) and collected data from August 20 to September 15, 2018. An online reporting form was filled on a real-time basis, and the medical officers analyzed this to generate alerts for various departments. The system disseminated the signals based on urgency. Situation analysis and the report of the actions taken were prepared and maintained for further references and improvements. Recommendations were drafted based on the day to day experience and were sent to the state and district officials on the same day for further course correction.

**Results**

The team investigated 951 health-related events in media surveillance between August 20, 2018, and September 14, 2018. Among the media surveillance events collected, 62% was from print media, 23% was from social media, 14% was from television, and 1% from other media sources. The health events captured were further categorized into 18 events. A total of 184 alerts were raised during the study period. The breakup of the same include deaths (30%), communicable diseases (19%), pollution, and other environmental issues (23%). The alerts were also mapped to the respective geographical area and disseminated accordingly through the available communication channels. Follow up communications regarding the public health actions taken based on the alerts were also monitored, documented through standardized formats.

**Conclusions**

The media surveillance supplemented the public health surveillance system and helped the health system prioritize its public health response during these challenging times. Based on the alerts generated and communicated, prompt public health responses happened. The feedback loop helped in learning and relearning from within the system, thereby exploring this sensitive tool's robustness in public health surveillance.

**References**

1. Ohl CA, Tapsell S. Flooding and human health. BMJ. 2000 Nov 11;321(7270):1167-8. doi: 10.1136/bmj.321.7270.1167. PMID: 11073492; PMCID: PMC1118941.

2. Noji EK. The public health consequences of disasters. Prehosp Disaster Med. 2000 Oct-Dec;15(4):147-57. PMID: 11227602.

3. Health Studies - Disaster Epidemiology | CDC [Internet]. 2020 [cited 2020 Sep 27]. Available from: https://www.cdc.gov/nceh/hsb/disaster/epidemiology.html

4. Ao TT, Rahman M, Haque F, Chakraborty A, Hossain MJ, Haider S, Alamgir AS, Sobel J, Luby SP, Gurley ES. Low-Cost National Media-Based Surveillance System for Public Health Events, Bangladesh. Emerg Infect Dis. 2016 Apr;22(4):720-2. doi: 10.3201/eid2204.150330. PMID: 26981877; PMCID: PMC4806969.

5. Sharma R, Karad AB, Dash B, Dhariwal AC, Chauhan LS, Lal S. Media scanning and verification system as a supplemental tool to disease outbreak detection & reporting at National Centre for Disease Control, Delhi. J Commun Dis. 2012 Mar;44(1):9-14. PMID: 24455910.

**Fig. 1 (abstract A20).**
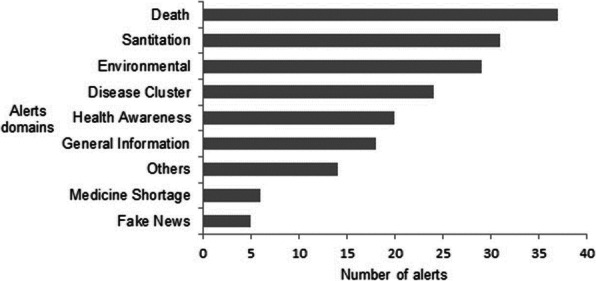
Category of alerts generated in media surveillance activity in the state of Kerala, India during the months of August & September 2018 (N=184)

## A21 Eliciting drug abuse status in the fast-urbanizing Solan district of Himachal Pradesh, India, 2019

### Ajay Kumar Singh^1^, Kushel Verma^2^, Vaishali Sharma^3^

#### ^1^District Programme Officer, Department of Health and Family Welfare, Office of Chief Medical Officer, district Solan, Himachal Pradesh, India; ^2^Psychiatrist, Department of Health and Family Welfare, Office of Medical Superintendent, District Hospital Solan, Himachal Pradesh, India; ^3^Psychologist, Department of Health and Family Welfare, Office of Chief Medical Officer, district Solan, Himachal Pradesh, India

##### **Correspondence:** Ajay Kumar Singh (ajay7777singh@yahoo.com)

**Background**

Substance use disorder (SUD) affects individuals across various strata of society and is considered a bio-psychosocial problem. United Nations World Drug Report 2019 states that in 2017, about 5.5% of the global population (271 million) had used drugs, and 35 million were estimated to be suffering from drug use disorders [1]. Opioids cause the most harm and account for two-thirds of the deaths attributed to drug use. Adolescence (12-17 years) and early adulthood (18-25 years) are a significant period of transition, a period for physical and psychological development highly vulnerable to the use of drugs [2]. The Global burden of disease study 2017 estimated 42 million years of healthy life lost due to drug abuse and the peak level of drug use in the 18-25-year age group [3]. The prevalence of SUD, including alcohol and tobacco, is high in India's general population [4]. Solan, the district with the second-largest urban population in Himachal Pradesh, has witnessed mushrooming of educational institutes and rapid industrialization closely knitted with migratory population dynamics. Police and media records showed a surge of drug abuse cases in the district in recent times. Many screening tools have been employed worldwide to identify individuals with drug abuse problems in a relatively short period [5,6]. These instruments are a set of standardized questionnaires designed to have a high degree of sensitivity. The absence of a regional prevalence survey in the Solan district on SUD poses difficulty planning interventions to address this urging issue. Hence, we undertook the burden estimation of SUD and the associated morbidity pattern present in the region.

**Methods**

We undertook an analysis of the data from the outdoor patients registered (OPD) of six months during the year 2019 in the de-addiction clinic of the Solan district hospital. After this, a cross-sectional study was conducted amongst 194 students of 18-25 years among one randomly selected university from among the seven universities present in the region. The students were sensitized about physical and mental health with two information, education and communication (IEC) camps held in the university. Confidence building task among the identified students was undertaken through these techniques. Subsequently, an assessment of current substance use was undertaken by ensuring confidentiality amongst the participants. Informed written consent was sought from all the participants, and necessary administrative approvals were obtained before the study. We used the standardized self-administered questionnaires of the Drug Abuse Screening Test (DAST), the Fagerstrom Test for Nicotine Dependence (FTND), the Fagerstrom Test for Nicotine Dependence- Smokeless Tobacco (FTND-ST), and the Alcohol Use Disorder Identification Test (AUDIT) for assessing substance use. Participant information collected was analyzed using Epi Info version 7 and Statistical Package for the Social Sciences version 21 software. The results were expressed in proportions. Chi-Square values (χ^2^) were estimated, and p values of less than 0.05 were considered statistically significant.

**Results**

Secondary data of the OPD records revealed a 76% prevalence of substance use among adolescents. Following this, a cross-sectional study was undertaken. The mean ± SD age of the study group was 22.20 ± 2.21 years. The study group had 138 (71.1%) males and 56 (28.9%) females. Prevalence of various drugs used by the age group of 18-25 was 69.4% (Chitta: a recent synthetic opioid), 16% (alcohol), 9% (heroin), 3.5% (chewable tobacco), 1.4% (cannabis), and 0.7 (prescription drug abuse). These differences were found to be statistically significant with a χ^2^=303.00 and a *p-value of* 0.00. The overall prevalence of substance abuse was observed as 38.9% (χ^2^=7.1, *p*=0.00). The DAST score varied significantly (χ^2^=445.07, *p*=0.00) among low (80%), moderate (9.7%), substantial (1.5%) and severe drug abuse potential (0.5%) among the students. The Fagerstrome scale (FTND) elicited 96.9% (very low), 2.6% (low), and 0.5% (medium) dependence for smoking, respectively (χ^2^=354.95, *p*=0.00). Modified Fagerstrome scale (FTND-ST) inferred 99.5 and 0.5% as very low and low smokeless tobacco dependence (χ^2^=191.02, *p*=0.00). The AUDIT scale revealed a harmful level of alcohol use in 28.7% of the study participants, whereas 3.1% and about 1% had medium level and high-level use potential, respectively (χ^2^=222.16, *p*=0.00).

**Conclusions**

The student community of Solan is affected by various kinds of drugs that are available in the market, including alcohol. They are also under nicotine dependence. The matter needs to be discussed with the concerned authorities to reduce the further impact on adolescents' psychosocial development. The screening tools employed helped in identifying the drug abuse immediately and initiating the required counseling and support. Deriving mileage from the study, the university authorities installed strict administrative measures, including policing, electronic camera monitoring and surveillance, and organizing parent- guardian meetings. The university authorities have made recommendations for initiating pharmacovigilance in the region to the Deputy Commissioner of the district.

**References**

1. United Nations Office on Drugs and Crime. World Drug Report 2019: Executive Summary, Conclusions, and Policy Implications. 2019. https://wdr.unodc.org/wdr2019/prelaunch/WDR19_Booklet_1_EXECUTIVE_SUMMARY.pdf Accessed 1 Oct 2020.

2. United Nations Office on Drugs and Crime. World Drug Report 2018: Drugs and Age- Drugs and Associated issues among Young People and Older People. 2018. https://www.unodc.org/wdr2018/prelaunch/WDR18_Booklet_4_YOUTH.pdf Accessed 1 Oct 2020.

3. Institute for Health Metrics and Evaluation (IHME). Findings from the Global Burden of Disease Study 2017. Seattle, WA: IHME, 2018. http://www.healthdata.org/sites/default/files/files/policy_report/2019/GBD_2017_Booklet_Issuu_2.pdf Accessed 1 Oct 2020.

4. Murthy P, Manjunatha N, Subodh BN, Chand PK, Benegal V. Substance use and addiction research in India. Indian J Psychiatry. 2010 Jan;52(Suppl 1):S189-99. doi: 10.4103/0019-5545.69232. PMID: 21836677; PMCID: PMC3146212.5.

5. Rakesh Lal. Substance Use Disorder: Manual for Physicians. http://www.antoniocasella.eu/archila/Lal_Manual_2005.pdf Accessed 1 Oct 2020.

## A22 Evaluation of the Leprosy Surveillance System in Raipur, Chhattisgarh, 2018

### Manish Gawande^1^, Amol Patil ^2^, Pankaj Bhatnagar^1^

#### ^1^National Public Health Surveillance Project, World Health Organization, New Delhi, India; ^2^South Asia Field Epidemiology and Technology Network, New Delhi, India

##### **Correspondence:** Manish Gawande (manish.gawande@gmail.com)

**Background**

Globally, the prevalence of leprosy had dropped between 1985 to 2000(21/10000 population to 1/10000 population) [1]. India contributes to more than 50% of newly detected leprosy cases (1). India launched the National Leprosy Elimination Programme in 1983 to eliminate leprosy as a public health problem(to reduce prevalence to less than 1 case/10000 population)[2]. Prevalence of leprosy cases was higher than the national average (<1 case/10000 population) in Chhattisgarh state of India (2 to 4 cases/10000 population)[3]. In 2014, 41 districts in 11 states of India had a prevalence of more than 2 cases/10000 population. Out of these 41 districts, 11 districts were in Chhattisgarh [3]. We evaluated the NLEP in Chhattisgarh district to identify the gaps in leprosy surveillance.

**Methods**

We did a cross-sectional study in Chhattisgarh state, between April 2017- March 2018. We defined a case of leprosy as hypo-pigmented or reddish skin lesion(s) with definite sensory deficit or involvement of the peripheral nerves, as demonstrated by thickening with loss of sensation and weakness of the corresponding muscles, or demonstration of Mycobacterium leprae in the lesions. We followed the U.S. Centers for Disease Control and Prevention guidelines for surveillance system evaluation. We selected Raipur district by convenience sampling and selected Aarang block, primary health centre Mandir Hasaud and health sub-centre Paloud by simple random sampling. We assessed selected attributes including simplicity, flexibility, data quality, acceptability, positive predictive value, representativeness, timeliness, stability and usefulness. We interviewed ten health personal at the health sub-centre, two health personnel at the primary health centre, and the District Leprosy Officer. We also reviewed monthly reports and patients’ treatment cards.

Simplicity was considered good if >80% of staff felt the referral form was simple or were aware of the case definition. The system was considered flexible if any health staff used alternate modes of reporting. Data quality was considered good if >80% of the data fields were completed on the monthly reports, and the system was considered acceptable when >80% of the health facilities submitted monthly reports.

We considered sensitivity as good if >80% of leprosy cases from government health facilities were reported in the system. The positive predictive value was considered good if >80% of the reported cases had the disease and representativeness was considered good if >10% of cases were reported by the private sector and >90% of government health facilities in the primary health centre reported cases. Timeliness was considered good if >90% of the monthly reports were submitted on time and stability was considered good if there was no shortage of staff, no system breakdown, and if forms were not out of stock.

**Results**

Village health workers identified suspected cases of leprosy during house visits and referred them to the primary health centres where Medical Officers confirmed the diagnosis and initiate treatment. The surveillance data flew from the health sub-centres and were compiled at primary health centres. The primary health centres, in turn, sent the data to the District Leprosy Office once a month. The districts sent the data once every quarter to the state, which in turn compiled and shared with the National Leprosy Division once a year. A designated person was responsible for reporting at each level. All ten health sub-centre staff members interviewed found the reporting form simple, and all were aware of the leprosy case definition. The system was flexible in that all staff referred cases using an alternate mode of reporting (telephone/message). Monthly data were captured on forms that include different categories of variables, and data quality was poor. We found 20% to 63% of the variables on the reporting forms to be completely filled. All (30/30) reporting units that we assessed submitted monthly reports, as per guidelines, indicating acceptability. Sensitivity could not be assessed because the total number of leprosy cases is not available. The positive predictive value was poor, as 19 of the 40 reported cases (48%) actually had the disease. Representativeness was mixed in that no case was reported from the private sector, whereas all five government health facilities that were assessed reported cases. Timeliness could not be assessed as the date of monthly report arrival at the district office, and the report dispatch date was not available. The system was stable in that all staff positions related to leprosy surveillance were filled, there were no system breakdowns, and reporting forms were not out of stock. The surveillance system helps to detect leprosy cases with a grade-II disability that require reconstructive surgery (Table 1). During 2017-18, seven cases had reconstructive surgery, illustrating the usefulness of the system.

**Conclusions**

We conclude that the leprosy surveillance system of Raipur is simple, flexible, acceptable, stable and useful; however, data quality, positive predictive value and representation of private health facilities need improvement. We recommend training of block program managers on monthly report preparation by District Leprosy Officer, training of block and district data managers about the timeliness of reports including date of dispatch at reporting unit or date of receipt at district office and sensitization of private health facilities for case reporting through meetings by Government Medical Officers.

**Acknowledgements**

We acknowledge the support of the State Leprosy Officer, Chhattisgarh, Chief Medical Officer and District Leprosy Officer of Raipur and Block Medical Officer, Health staff of PHC Aarang. We thank Dr Pauline Harvey, Team Leader, National Polio Surveillance Project, World Health Organization India and U.S. Centers for Disease Control and Prevention (US CDC), and Dr Rajesh Yadav, Division of Global Health Protection, US CDC, India, for their technical assistance and scientific review.

**References**

[1]. World Health Organization. Global Leprosy Strategy 2016−2020: Accelerating towards a leprosy-free world [Internet]. [cited 21 February 2020]. Available from: https://www.who.int/lep/resources/9789290225096/en/

[2]. Ministry of Health and Family Welfare. National Leprosy Eradication Programme. [cited 21 February 2020]. Available from: https://www.nhp.gov.in/national-leprosy-eradication-programme_pg

[3]. German RR, Lee LM, Horan JM, et al. Updated guidelines for evaluating public health surveillance systems: recommendations from the Guidelines Working Group. MMWR Recomm Rep. 2001;50(RR-13):1‐CE7

**Table 1 (abstract 22).** Number and proportion of cases with grade-II deformities, by year

Year	Number cases with grade-II deformity	Proportion of cases with grade-II deformity
2013-14	85	10
2014-15	85	10
2015-16	137	13
2016-17	88	9.1
2017-18	79	9.6

## A23 Dengue outbreak investigation in East Delhi, India, 2015

### Somashekar Dundaiah, Venkatesh Bhadravathi Govindappa

#### Public Health Department, East Delhi Municipal Corporation, Delhi, India

##### **Correspondence:** Somashekar Dundaiah (asksoma@gmail.com)

**Background**

Dengue, a mosquito-borne arboviral disease, is a significant public health problem globally. The principal vector for transmission is Aedes agypti. In India, dengue is endemic in almost all states and reported all four dengue virus serotypes (DENV1, DENV2, DENV3 & DENV4) [1]. Since 1996, the capital city, Delhi, had reported six outbreaks in cyclical trend (3-4 years interval), all during monsoon and post-monsoon months (Vector-borne diseases, annual reports 1996-2014, Municipal Corporation Delhi; unpublished data). In 2015, the largest ever dengue outbreak occurred. We investigated the dengue outbreak in East Delhi to describe the epidemiology of the outbreak and assess risk factors for transmission.

**Methods**

We adopted the standard National Vector Borne Disease Control Programme case definition of confirmed dengue [2]. We defined a case of dengue as a fever of 2-7 days duration in a resident of East Delhi area from May 14 to December 18, 2015, with at least two symptoms (headache, retro-orbital pain, myalgia, arthralgia, rash, or hemorrhagic manifestations) and positive lab test by IgM MAC ELISA or NS1 antigen test. Cases were ascertained from the hospitals of East Delhi. We collected data with variables like age, gender, address, name of the hospital, date of onset of fever, date of admission, date of notification, and type of lab test performed for dengue detection. A line list was prepared and analyzed for descriptive analysis.

Based on the descriptive analysis, a null hypothesis postulated that there is no risk of dengue transmission in the presence of domestic and peri-domestic breeding sites.

We conducted an unmatched 1:2 case-control study with 107 cases and 214 controls, assuming 30% exposure among controls; 95% confidence limit, 80% power, and risk ratio (OR) of 2. A control was defined as "any person residing in the same neighborhood as the case with no history of fever between May 14 and December 18, 2015. Case-patients were selected randomly from each of the 64 municipal wards. We set the first neighborhood control from the 50^th^ house of case house and 2^nd^ control from the 50^th^ house of the 1^st^ control. The structured questionnaire included demographic information, address of workplace/school/educational institute, clinical manifestations, laboratory test, travel history, presence of overcrowding (persons per room: in 1 room with >2 persons, two rooms with >3 persons, three rooms with >5 persons, four rooms with >7 persons) [3], presence of domestic breeding sites, use of temephos granules in desert cooler, use of mosquito repellent coil/liquid/cream, wearing full sleeves and pants and windows screened with mesh. We trained Malaria Inspectors of East Delhi and collected data between December 17 and 22, 2015. We also collected Aedes larval surveyed data from January-October, 2015. Established criteria for the larval survey were adopted to assess the trend. Data entry and analysis was done in Epi-info 7.1.2.0 software. We took the institutional clearances and have not collected personal identifiers and maintained the collected information's confidentiality.

**Results**

We identified 1775 dengue cases (61% were males) and six deaths. The median age was 21 years (range: 7 months-88 years). Age group 25 to 44 years was the most affected, with 504 cases (30%). The attack rate of dengue was 4 per 10,000 populations. The median duration of fever in cases was six days (range: 2 days - 30 days). The first case was reported in the last week of May, a sharp rise observed from the first week of September. Cases declined after the 2^nd^ week of October, and the last case was reported in the first week of December. The average duration between hospital admission and reporting of dengue cases was 6.6 days (Range: 1-31 days). Dengue cases confirmed by NS1 antigen ELISA test were 1169(66%) and 606 (34%) by MAC IgM ELISA tests.

Clinical symptoms among the enrolled 107 cases in the case-control study included headache (84%), restlessness (73.5%), muscle pain (70%), joint pain (58%), pain behind eyes (52%), rashes over the body (28%), bleeding gum (8%). Eight percentage of cases also experienced unconsciousness, and 6% reported bleeding from the nose. A case-control study showed that over-crowding at home was significantly associated with dengue transmission (OR 2.02, 95% CI 1.2-3.2). Use of temephos granules in desert cooler is protective(OR 0.3, CI 0.08-1.1) (Table 1). We observed high larval indices during monsoon months preceded the dengue outbreak. Breteau index was 15 (< 5, low risk of transmission) in the second week of August 2015 and up to the first week of September 2015 and persisted above five till the end of October 2015.

**Conclusions**

We confirmed the dengue outbreak in East Delhi due to a surge in larval indices during post-monsoon months and the presence of overcrowding. This reinforces that dengue transmission also depends on the human density and can potentiate the epidemiological significance of Aedes breeding habitats [5]. We recommended awareness campaigns on the use of temephos granules in desert coolers to prevent and control Aedes mosquito breeding.

**Reference**

1. Kumari R, Kumar K, Chauhan LS. First dengue virus detection in Aedes albopictus from Delhi, India: its breeding ecology and role in dengue transmission. Trop Med Int Health. 2011 Aug;16(8):949-54. doi: 10.1111/j.1365-3156.2011.02789.x.

2. Guidelines for Clinical Management of Dengue Fever, Dengue Haemorrhagic Fever and Dengue Shock Syndrome [Internet]. Directorate of National Vector Borne Control Programme, Directorate General of Health Services; 2008 [cited 2020 January 2]. Available from: https://nvbdcp.gov.in/Doc/Clinical%20Guidelines.pdf

3. UNCHS (Habitat) 1995 Human settlement interventions addressing crowding and health issues. Nairobi: United Nations Centre for Human Settlements.

4. Padmanabha H, Durham D, Correa F, Diuk-Wasser M, Galvani A. The interactive roles of Aedes aegypti super-production and human density in dengue transmission. PLoS Negl Trop Dis. 2012;6(8):e1799. doi: 10.1371/journal.pntd.0001799.

**Table 1 (abstract A23).** Risk factors for dengue cases in East Delhi, 2015

Exposures	Case N=107	Control N=214	Odds Ratio OR (95% C.I)
Use of temephos granules in desert coolers	71 (32%)	149 (68%)	0.3 (0.1-1.1)
Wearing full sleeves clothes and pants	77 (72%)	167 (79%)	0.6 (0.4-1.1)
Using mosquito net	14 (13%)	35 (17%)	0.7 (0.3-1.4)
Window screened with mesh	46 (44%)	102 (48%)	0.8 (0.5-1.3)
Use of desert cooler	77 (72%)	153 (71%)	0.9 (0.5-1.6)
Overcrowding at home	62 (58%)	83 (40%)	2.0 (1.2-3.2)

## A24 Measles outbreak in a marginalized population of Jogapatti block, West Champaran district, Bihar, India, February 2019

### Vishesh Kumar^1^, Sanjay Kumar Singh1, Ankur Nair^1^, Nihar Ranjan Ray^1^, Sushma Choudhary^2^, Pauline Harvey^1,3^

#### ^1^National Public Health Surveillance Project, World Health Organization Country Office, Delhi, India; ^2^South Asia Field Epidemiology and Technology Network, Delhi, India; ^3^Global Immunization Division, U.S. Centers for Disease Control and Prevention, Atlanta, Georgia, USA

##### **Correspondence:** Vishesh Kumar (vikumar@who.int)

**Background**

The World Health Organization (WHO) South-East Asia Region, including India, resolved to eliminate measles and rubella by 2023 [1]. In 2018, India reported 20,925 measles cases with 15.4 per million population, and the Bihar state reported 5,414 cases with an incidence of 27.2 per million population [2]. The 2016 National Family Health Survey-4 reported measles vaccine coverage for Bihar state of 79.4% for children ages 12–23 months [3]. On 2 February 2019, a suspected measles outbreak was reported in Jogapatti block in West Champaran district, Bihar, India. We investigated to describe the epidemiology, estimate vaccine coverage, vaccine efficiency, and to identify risk factors for the outbreak.

**Methods**

We did a retrospective cohort study among children aged less than five years in Dhadhwa village, Jogapatti block. We reviewed the weekly vaccine-preventable disease surveillance data for West Champaran district and Bihar state from 1 October 2018 to 1 February 2019 using Government of India measles outbreak guidelines [4]. We defined a probable measles case as fever and maculopapular rash with cough or coryza or conjunctivitis in a Jogapatti block resident from 1 October 2018 to 31 March 2019. We defined a confirmed case as a probable case with detection of measles-specific IgM by enzyme-linked immunosorbent assay (ELISA). We defined a probable measles death as occurring within 30 days of rash onset unless the death was from an injury or other unrelated cause in a Jogapatti block resident during the reference period. We did active surveillance for case detection. We reviewed medical records in health facilities catering to Jogapatti block. After obtaining verbal informed consent from the head of household, we enumerated all individuals residing in the household since 1 October 2018. For all household members (alive or who had died) with reported fever and maculopapular rash during the study period, the individual, caregiver, or head of the household was interviewed using a pre-tested, semi-structured questionnaire to collect demographics, socioeconomic status, clinical symptoms, treatment, vaccination status, and reasons for non-vaccination. Vaccination status was determined by reviewing the vaccination card or based on the mother's or caregiver's recall if the vaccination card was not available. We collected five serum specimens from probable cases with rash onset within 4–28 days of collection in Jogapatti block. All serum specimens were sent to the National Measles Laboratory for measles-specific IgM antibody testing by ELISA method [4]. We calculated proportions for attack rates. We calculated relative risk(RR) with 95% confidence interval (95% CI) to identify risk factors.

**Results**

Based on routine weekly measles surveillance reports, we identified an excess number of probable measles cases and deaths in Jogapatti block from October 2018 to February 2019 compared to the same time interval in the previous three years. Among 12,132 persons in eight villages of Jogapatti block, we identified 256 measles cases between 1 October 2018 and 31 March 2019, for an attack rate (AR) of 2% (Figure 1). Among the cases, 131 (51%) were female, the median age was 45 months (range: 6 months to 35 years), and the AR was 7% for children aged less than five years and 1% for those >5 years of age. Seven children with measles died. The overall case fatality rate (CFR) was 3%, but the CFR for children aged greater than or equal t five years was 11%. All seven children who died were from the Mushar community and resided in Dhadhwa village. Three (60%) of the five serum specimens collected were positive for measles IgM antibodies. Dhadhwa village had a population of 1525 with four communities represented, i.e., Mushar, Dhobi, Yadav, and Rajput. In Dhadhwa village, we identified 81 probable measles cases, all in children aged less than or equal to 10 years. The overall AR was 15% but 21% among children aged less than or equal to five years. There were seven deaths for a CFR of 9%. Only 17 (21%) cases received vitamin A, and 2 (2%) were hospitalized. Among the cases, 47 (58%) were female, and the median age was 27 months (range: 6 months–10 years). The most common symptoms reported were cough (81%) and coryza (74%), and the most common complications were diarrhoea (35%) and pneumonia (10%). Among 227 children less than or equal to 5 years of age in the Dhadhwa village cohort with vaccination data, coverage with MCV first dose (MCV1) was 64% and MCV second dose (MCV2) was 35%. Vaccine effectiveness for MCV1 was 77%. Reasons given for not vaccinating children included fear of illness following vaccination (38%) and lack of vaccination sessions (30%). Not receiving any MCV dose (42/127) compared to being vaccinated (15/145) (RR=3.2; 95% confidence interval=1.9–5.5), not receiving vitamin A (43/147) compared to receiving vitamin A (14/125) (RR= 2.6; 95% CI=1.5–4.5), and being part of the Mushar community (48/108) compared to other communities (9/164) (RR=8.1; 95% CI=4.1–15.8) were the risk factors for the measles outbreak.

**Conclusion**

There was a measles outbreak in Jogapatti block between 1 October 2018 and 31 March 2019. Children aged less than five years and residents of Dhadwa village were more affected. Non-vaccination and not receiving vitamin A supplements were risk factors for the measles outbreak. We recommend improving vaccine coverage. We also recommend improving Vitamin A administration.

**Acknowledgements**

We thank Dr Ann M. Buff, U.S. Centers for Disease Control and Prevention, for her technical assistance and scientific review.

Figure 1. Probable measles cases by week of rash onset in Jogapatti block, West Champaran district, Bihar, India from 1 October 2018 to 31 March 2019

**References**

[1]. World Health Organization (WHO). Strategic plan for measles and rubella elimination in the WHO South-East Asia Region: 2020–2024. New Delhi: WHO, Regional Office for South-East Asia, 2019. [https://apps.who.int/iris/handle/10665/330356]

[2]. World Health Organization (WHO) and the Ministry of Health and Family Welfare (MOHFW), Government of India (GOI). Measles and rubella surveillance bulletin, December 2019. New Delhi: WHO and MOHFW, GOI, 2019.

[3]. International Institute for Population Sciences (IIPS) and ICF. National family health survey (NFHS-4), 2015-16: India. Mumbai: IIPS, 2017.

[4]. Ministry of Health and Family Welfare (MOHFW), Government of India (GOI). Measles surveillance and outbreak investigation: a field guide. New Delhi: MOHFW, GOI, September 2006.

**Fig. 1 (abstract A24).**
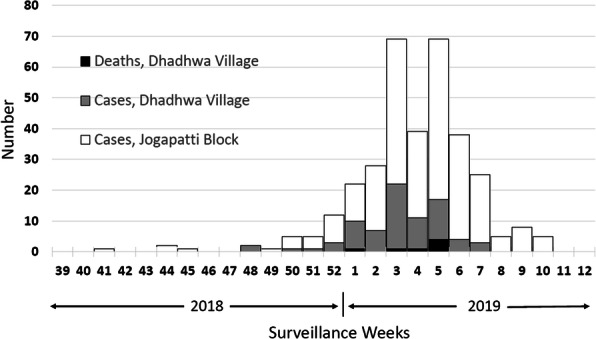
Probable measles cases by week of rash onset in Jogapatti block, West Champaran district, Bihar, India from 1 October 2018 to 31 March 2019

## A25 Evaluation of the Measles Component of the Integrated Disease Surveillance Programme in Mirzapur District, Uttar Pradesh, India, March 2018

### Hamid Sayeed^1^, Pankaj Bhatnagar^1^, Pauline Harvey^1,2^

#### ^1^National Public Health Surveillance Project, World Health Organization, New Delhi, India; ^2^Global Immunization Division, U.S. Centers for Disease Control and Prevention, Atlanta, GA, USA

##### **Correspondence:** Hamid Sayeed (srcallahabad@npsuindia.org)

**Background**

In 2017, India reported 45,773 suspected cases of measles and 678 outbreaks [1]. Uttar Pradesh, the most populous state in India, reported 9,094 cases and 343 outbreaks, and Mirzapur district in Uttar Pradesh said seven of these measles outbreaks, resulting in 64 case-patients (Surveillance Report 2018, WHO State Office Lucknow, unpublished data). India relies on a robust measles surveillance system to detect and respond to outbreaks, identify programmatic gaps, and monitor progress towards the 2023 measles elimination goal [2]. Measles surveillance is part of the Integrated Disease Surveillance Programme (IDSP) in India. We evaluated the measles component of the IDSP to identify strengths and weaknesses of the system and provide recommendations to improve the system and achieve the elimination target.

**Methods**

We evaluated the measles surveillance system using the U.S. Centers for Disease Control and Prevention guidelines for surveillance system evaluation [3]. There were 298 health sub-centers in Mirzapur district that reported weekly measles surveillance data on a syndromic (S) report form to 12 blocks (sub-district administrative unit). Each block had a primary health center (PHC) with a nodal person responsible for collating the S forms and filling the weekly presumptive (P) report form. The block PHCs submitted a weekly collated S form, a weekly P report form and a weekly laboratory (L) report form to the district by Monday. Also, 67 primary or community health centers, hospitals, laboratories, private health facilities, P, and L reports directly to the district. We randomly selected two-block PHCs and two health sub-centers that reported to each of the chosen block PHCs for a total of six health sub-centers. We reviewed measles surveillance data from the selected sub-centers, block PHCs, and districts for March 2018. We verified 16 weekly S report forms (one S form per week per health sub-center), eight P report forms (one P form per week per block PHC) and eight L report forms (one L form per week per block PHC). Using a semi-structured questionnaire, we interviewed key informants, including the district surveillance officer, epidemiologist, data manager, data entry operators, health center medical officers-in-charge and auxiliary nurse-midwives. We assessed simplicity by the proportion of staff who knew the reporting form submission timeline, the officer responsible for receiving the form, and the ease of reporting. We considered the system flexible if team submitted reports through a mode other than paper. For data quality, we checked the forms for measles data completeness for a place, person, and time fields and cross-checked the data by matching the S, P, and L reports on the IDSP web portal with original hard copies retained at the district. We considered the staff’s willingness to participate in surveillance activities for acceptability, and the proportion of private health facilities reporting representativeness. We evaluated the timeliness of form submission at the first three levels of the surveillance system. We assessed system stability using standard definitions and the availability of reporting forms. Lastly, we evaluated the overall usefulness of the surveillance system. The district held weekly meetings to review and compile all the surveillance forms submitted; all data forms were uploaded into the IDSP web portal for review by state and central levels. The district used standard definitions for reporting suspect, probable, and confirmed measles cases for identifying potential outbreaks consistent with measles surveillance guidance [4]**.** The district surveillance officer and epidemiologist used data from the IDSP measles surveillance system to investigate potential outbreaks.

**Results**

Of the nine staff interviewed in March 2018, 8 (89%) knew both the reporting form submission timeline and the officer responsible for receiving report forms (Table 1). All 16 S and eight L report forms, and four weekly district reports reviewed were submitted on time. Fields for a place, person, and time information were complete for 2 (13%) of the 16 S report forms, and none of P or L report forms. Three (75%) of four health sub-centers had no paper S forms. Both electronic and paper reporting forms were available at the block PHCs and district; one staff out of nine staffs (11%) reported sending P and L report forms via email from the block PHC to the district. Of the 67 facilities sending P and L forms directly to the district, five (7%) were from private health facilities.

**Conclusions**

We concluded that the IDSP measles surveillance system in Mirzapur district was simple and acceptable to the users. Although the timeliness of data reporting was good, the data quality was poor for reporting completeness. Based on the indicators measured, the surveillance system was not flexible, neither representative nor stable. Despite the gaps identified, the IDSP measles surveillance system was useful overall. In 2017, health authorities investigated seven potential outbreaks and confirmed 64 case-patients based on measles surveillance data reported in both the state and national surveillance bulletins [1]. To improve data quality and completeness of reporting, we recommended that the district surveillance officer and IDSP officers conduct surveillance sensitization activities for auxiliary nurse midwives, medical officers, and nodal persons at least annually. The district surveillance officer should ensure that paper S forms are printed and distributed to health sub-centers to prevent stock-outs. Representativeness could be improved by enrolling more private health facilities into the IDSP for measles surveillance.

**References**

1. Epidemiologist D. A monthly Surveillance Report from Integrated Disease Surveillance Programme National Health Mission March 2017. 2017;2(March):1–22. Available from: https://idsp.nic.in/. Accessed 09 February 2021.

2. SEAR India Communications. Measles & Rubella in South-East Asia [Internet]. Available from: https://www.who.int/southeastasia/health-topics/measles. Accessed 09 February 2021.

3. German RR, Westmoreland D, Armstrong G, Birkhead GS, Horan JM, Herrera G, et al. Updated Guidelines for Evaluating Public Health Surveillance Systems [Internet]. Atlanta; 2001. Available from: https://www.cdc.gov/mmwr/preview/mmwrhtml/rr5013a1.htm#top. Accessed 09 February 2021.

4. Surveillance Guide for Vaccine-Preventable Diseases in the WHO South-East Asia Region. 2017. Available from: https://apps.who.int/iris/bitstream/handle/10665/277459/Module1-Measles%26Rubella.pdf?sequence=1&isAllowed=y. Accessed 09 February 2021.

**Table 1 (abstract A25).** Number and proportion of indicators describing attributes of the measles component of the Integrated Disease Surveillance Programme in Mirzapur District, Uttar Pradesh, India in March 2018

Attribute	Indicator	N	N	(%)
Simplicity	Aware of S, P, and L report form submission day	8	9	(89)
	Aware of the officer to whom S, P, and L report forms submitted	8	9	(89)
	Reported filling of S, P, and L report forms was easy	7	9	(78)
Flexibility	Submitted S, P, and L report forms via an alternative mode (not paper)	1	9	(11)
Data Quality	S report forms with the place, person, and time fields completely	2	16	(13)
	P report forms with the place, person, and time fields complete	0	8	(0)
	L report forms with the place, person, and time fields complete	0	8	(0)
	Availability of submitted S, P, and L report forms retained by the district	28	32	(88)
	Availability of S, P, and L report forms on IDSP web portal	32	32	(100)
Acceptability	Weekly district surveillance meetings	4	4	(100)
Representativeness	Private health facilities submitting P and L report forms	5	67	(7)
Timeliness	S report form submitted by Monday	16	16	(100)
	P report form submitted by Tuesday	4	8	(50)
	L report form submitted by Tuesday	8	8	(100)
	District report submitted by Wednesday	4	4	(100)
Stability	Availability of printed S, P, and L report forms at the health facility on interview day	9	10	(90)
	Stock out of S, P, and L report forms at the health facility in the last three months	4	10	(40)

S = syndromic; P = presumptive; L = laboratory; IDSP = Integrated Disease Surveillance Programme

## A26 Investigation of the measles outbreak, Amoja village, Phulparas block, Madhubani, Bihar, India, October–November 2018

### Sambit Pradhan^1^, Sushma Choudhary^2^, Pankaj Bhatnagar^1^

#### ^1^National Public Health Surveillance Project, World Health Organization, Delhi, India; ^2^South Asia Field Epidemiology and Technology Network, Delhi, India

##### **Correspondence:** Sambit Pradhan (sambitpradhan2500@gmail.com)

**Background**

In the World Health Organization (WHO) South-East Asia Region, measles has been targeted for elimination by 2023[1]. In 2018, a total of 34,741 laboratory-confirmed cases were reported from this region, of which 19,474 (56%) were in India[2]. On October 25, 2018, seven case-patients of fever with rash were reported from Amoja village by the auxiliary nurse midwife of Routiniya sub-centre of the Phulparas block Madhubani, Bihar. We investigated suspected cases to describe the epidemiology, identify risk factors for illness, and provide evidence-based recommendations for prevention and control.

**Methods**

We defined a suspected measles case as the onset of fever and rash in Amoja village resident during June 28–December 10, 2018. We defined a probable case of measles as the onset of fever with maculopapular rash and any of the following symptoms: cough, coryza, or conjunctivitis in a resident of Amoja village during June 28–December 10, 2018. We defined a confirmed case as a probable case for which enzyme-linked immunosorbent assay was reactive for measles IgM. Four field monitors each conducted house-to-house searches for cases in the five tolas of the village. The India Epidemic Intelligence Service officer from the WHO National Public Health Surveillance Project verified the types of rash (maculopapular, vesicular) and other clinical features. We conducted a retrospective cohort study in which we defined a cohort as children <15 years of age residing in Amoja village during June 28–November 30, 2018. We collected information about clinical features, complications, hospitalizations, immunization against measles, vitamin A supplementation, post-rash vitamin A administration, travel history, and contact history using a semi-structured questionnaire. Immunization information was collected from the immunization card; if a card was not available, we relied on the mother's recall. We calculated proportions, attack rates, and vaccine effectiveness. We also reviewed immunization coverage and the routine immunization micro plan for the affected village. We collected information about the measles vaccine's administrative coverage for Amoja village for 2017–2018 and compared it with WHO concurrent monitoring coverage during September 2018. Serum samples from five children 5–12 years of age who had rash onset within 4–28 days before the date of sample collection were tested for measles and rubella IgM by enzyme-linked immunosorbent assay at Patna Medical College and Hospital (Patna, India).

**Results**

We identified 17 case-patients. The median age was seven years (range 1–19 years), and 11 out of 17 (64%) were female. In addition to fever and maculopapular rash, all case-patients had cough; 14 had coryza, 13 had conjunctivitis, eight had lymphadenopathy and itching, and six had submandibular pain. The complication of diarrhoea and pneumonia occurred in four and three case-patients, respectively. Two case-patients were hospitalized. No deaths were reported. Seven case-patients each received measles-containing vaccine first dose (MCV1) and second dose (MCV2) during their routine immunization. No case-patients received post-rash vitamin A due to its non-availability. The overall attack rate in the affected village was 0.5%; the highest attack rate (8%) occurred among children 5 to <10 years of age. Cases began on September 22, 2018, and peaked on October 20; the last case occurred on November 2. Cases clustered (65%, 10/17) in a Muslim tola. In the retrospective cohort study of 349 children <15 years of age, 177 (51%) were female, and 67 (19%) belonged to the Muslim community. An immunization card was available for 105 (30%) children. Among the cohort, MCV1 coverage was 85%; MCV2 coverage, 73%; and vitamin A coverage, 59%. Vaccine effectiveness was calculated at 83%. Not receiving MCV1 (relative risk [RR] 7.2 [95% confidence interval (CI) 2.8–18.5]) and belonging to the Muslim community (RR 7.2 [95% CI 2.7–18.9]) were associated with illness (Table 1). Among the 7 (21%) incompletely vaccinated children, reasons reported for incomplete immunization were fear of post-immunization fever (37%) and lack of awareness about the need for immunization (24%). Our review of the routine immunization micro plan found three sites where routine immunization sessions were conducted every month. During April 2017–March 2018, a total of 29 sessions were conducted at 36 planned sites. In Amoja village during 2017–2018, reported MCV1 coverage was 46%, and MCV2 coverage was 34%. Four of the five serum samples were reactive for measles IgM.

**Conclusions**

Our epidemiologic investigation, supported by clinical features confirmed by laboratory findings concluded that this measles outbreak in Amoja village, Madhubani district, Bihar, was most likely attributable to low measles immunization coverage. Fear of post-immunization illness was the most common reason for non-immunization. The lack of administration of vitamin A to case-patients probably contributed to complications. Nonreceipt of MCV1 and belonging to the Muslim community were associated with illness. We recommended administering vitamin A to measles case-patients for two consecutive days by the Accredited Social Health Activist and ensuring that all routine administration sessions occurred following the Medical Officer's micro plan in charge of the Primary Health Centre. Communication activities, such as meetings with mothers, and interpersonal communication, need to be conducted to create demand for immunization and mitigate the fear of illness by the Block Communication Mobilizer.

**References**

1. World Health Organization. WHO South-East Asia Region sets 2023 target to eliminate measles, rubella. https://www.who.int/southeastasia/news/detail/05-09-2019-accelerate-efforts-to-eliminate-cervical-cancer-who. Accessed May 19 2020.

2. World Health Organization. Immunization, vaccines, and biologicals. Measles. https://www.who.int/immunization/monitoring_surveillance/burden/vpd/surveillance_type/active/measles/en. Accessed October 27 2019.

**Table 1 (abstract A26).** Univariate analysis of risk factors for 17 measles cases, Amoja village, Phulparas block, Madhubani, Bihar, India, October–November 2018*

Risk factor	Ill	Not ill	RR (95% CI)
No immunization card	13 (81%)	231 (69%)	1.7 (0.5–6.1)
No immunization with MCV1, by card	1 (6%)	15 (5%)	2 (0.19–21)
No immunization with MCV1, by card + recall	8 (50%)	48 (14%)	7.2 (2.8–18.5)
Female sex	10 (62%)	167 (50%)	1.3 (0.5–3.3)
Illiterate mother	10 (62%)	170 (51%)	1.1 (0.5–4.2)
Muslim religion	10 (62%)	56 (17%)	7.2 (2.7–18.9)

*CI, confidence interval; RR, relative risk

## A27 Malaria outbreak investigation in Badaun District, Uttar Pradesh, India, August–October 2018

### Rakesh Vishwakarma^1^, Rajesh Kumar^1^, P. Kumar^2^, Kaushal Gupta^2^, V. K. Sharma^2^, Anil Sharma^2^, Asha Ram^2^, Amol Patil^3^, Gunjan Kumar^1^, Madhup Bajpai^1^, Pankaj Bhatnagar^1^

#### ^1^National Public Health Surveillance Project, World Health Organization, New Delhi, India; ^2^Department of Health and Family Welfare, Government of Uttar Pradesh, Lucknow, India; ^3^South Asia Field Epidemiology and Technology Network, New Delhi, India

##### **Correspondence:** Rakesh Vishwakarma (srcjaipur@npsuindia.org)

**Background**

India has the third-highest burden of malaria globally and contributes to 85% of malaria cases in the WHO Southeast-Asia Region [1, 2]. The Government of India has a goal to eliminate malaria by 2030 for which a National Framework for Malaria Elimination 2016–2030 has been developed [2,3]. This framework lays out broad principles to achieve the target of malaria elimination with the district as the unit of planning and a focus on endemic areas to reduce transmission [2]. Malaria control strategies in endemic areas include universal case management with parasitological diagnosis and treatment, strengthening surveillance, vector control interventions, and effective program management and coordination to deliver interventions [2]. In August 2018, cases of acute fever and over 40 deaths were reported in the local news media from Badaun District in Uttar Pradesh, India. We conducted an outbreak investigation to describe the epidemiology, identify risk factors, and provide recommendations to prevent additional cases and future outbreaks.

**Methods**

We compared the number of malaria cases reported in 2016 and 2017 with those reported in 2018 from Badaun District, Uttar Pradesh to confirm the outbreak. Malaria surveillance data prior to 2016 was not available for review. We defined a suspected malaria case as an acute febrile illness in a resident of Badaun District between 27 August and 03 October 2018. Suspected malaria cases were tested with either a rapid diagnostic test (RDT) to detect Plasmodium falciparum and Plasmodium vivax or microscopy to confirm parasite presence in thick peripheral blood smears. We defined a confirmed malaria case as a suspected case with detection of P. falciparum, P. Vivax or both by RDT or parasite presence by microscopy. Badaun District only reported aggregated positive microscopy results (i.e., the proportion by species was not provided) until 8 September 2018. Due to an increase in cases, Badaun District used only malaria RDTs from 9 September 2018 onwards. District hospital inpatient and outpatient records and malaria surveillance data were reviewed to identify malaria cases and deaths. In addition, a house-to-house survey was conducted in the four most-affected villages based on malaria surveillance data in Badaun District, which had 2,084 villages and an estimated population of 3,186,362. Using a semi-structured questionnaire, we collected information on socio-demographics, treatment received, and personal protective practices. In addition, we also observed the potential mosquito breeding sites and drainage systems in the most-affected villages.

**Results**

Badaun District reported 8,710 confirmed malaria cases from 1 January to 3 October 2018 compared to 989 and 1,325 malaria cases for all of 2016 and 2017, respectively. From 27 August to 3 October 2018, we identified 44,720 suspected malaria cases and 28 suspected malaria deaths in Badaun District. Among the 29,645 (66%) of suspected cases tested with microscopy or malaria RDTs, 7,269 (25%) were confirmed. Of these 29,645 suspected malaria cases, 3,060 (10%) suspected cases were tested with microscopy, and 1,847 (60%) were confirmed during 27 August to 8 September 2018. The remaining 26,585 (90%) suspected malaria cases were tested with RDTs during 9 September 2018 to 3 October 2018, when the district switched to RDTs to accommodate increased test volume; 5,422 (20%) were confirmed. Of the 5,422 cases confirmed by RDT, 2,397 (44%) were positive for P. falciparum and 3,025 (56%) for P. Vivax. Almost two-thirds (18, 64%) of suspected malaria deaths were reported prior to the use of malaria RDTs. The median age of confirmed cases was 18 years (range: 6 months–86 years). Overall attack rate was 14/1,000 for suspected malaria cases and 2/1,000 for confirmed malaria cases. Of the 16 blocks (i.e., sub-districts), four (25%) had attack rates of >2 per 1,000 population, and all were in the north-eastern part of the district that adjoined Bareli district, which had a concurrent malaria outbreak. The four highest block attack rates were 8/1,000 in Jagat, 7/1,000 in Dataganj, 4/1,000 in Samrer, and 3/1,000 in Binawar. Of the total 2,397 P. falciparum cases identified by RDTs, 1,135 (47%) were from Jagat block. In the four most-affected villages in Jagat block with 2,121 cases, 1,415 (67%) were males, 1,230 (58%) were <20 years of age, and 1,508 (71%) reported not using long-lasting insecticidal bed nets (LLINs) the previous night. All confirmed cases received treatment with artesunate and pyrimethamine-sulfadoxine. In the four most-affected villages in Jagat block, we observed Anopheles mosquito breeding sites less than 20 meters from houses and open drainage with stagnant water. We interviewed family members of six cases who died. Family members reported that none of the deceased cases used LLINs or other protective measures to prevent malaria. Three (50%) of the deceased cases were reported to have taken treatment.

**Conclusions**

We confirmed a large malaria outbreak in Badaun District from August to October 2018. In the four most-affected villages, most confirmed malaria cases were <20 years of age, males, and those who reported not using LLINs. Based on our recommendations, district health officials set up a control room for daily outbreak monitoring and response efforts. Vector control measures were implemented in the most-affected villages, including fogging, anti-larval spraying, and indoor residual spraying of houses. To prevent future outbreaks in Badaun District, we recommended strengthening malaria surveillance and improving intersectoral coordination. We also recommended training frontline health workers, promoting community awareness of early referral for fevers, using malaria RDTs to test persons with fever, and using of LLINs every night during the malaria-transmission season.

**References**

[1]. World Health Organization. World Malaria Report, 2018. Geneva: World Health Organization, 2018. [available from https://www.who.int/malaria/publications/world-malaria-report-2018/en/] Assessed on 12 March 2020

[2]. National Vector Borne Disease Control Programme. National framework for malaria elimination in India 2016-2030. New Delhi: Ministry of Health & Family Welfare, Government of India, 2016. [available from: https://nvbdcp.gov.in/WriteReadData/l892s/National-framework-for-malaria-elimination-in-India-2016%E2%80%932030.pdf] Accessed on 15 June 2020.

[3]. National Vector Borne Disease Control Programme. Strategic Plan for Malaria Control in India 2012-2017. New Delhi: Ministry of Health & Family Welfare, Government of India, undated. [available from: https://nvbdcp.gov.in/Doc/Strategic-Action-Plan-Malaria-2012-17-Co.pdf] Accessed on 12 March 2020.

**Fig. 1 (abstract A27).**
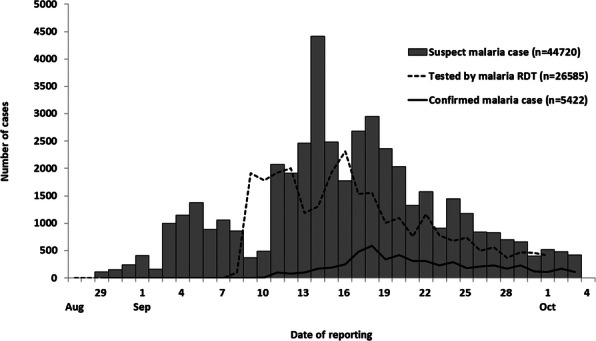
Distribution of suspected and confirmed malaria cases tested with the rapid diagnostic test in Badaun District, Uttar Pradesh, India from 27 August–3 October 2018

## A28 Profile of maternal deaths in Virudhunagar district, Tamil Nadu, India 2013-2018

### Vijay Krishnamoorthy, Muthusamy Santhosh Kumar

#### ICMR- National Institute of Epidemiology, Chennai, India

##### **Correspondence:** Vijay Krishnamoorthy (calldrvijay@gmail.com)

**Background**

Every day, approximately 830 women die from preventable causes related to pregnancy and childbirth globally [1]. About 99% of all maternal deaths occur in developing countries [1]. Maternal mortality is higher among women living in rural areas and among poorer communities [1]. Young adolescents face a higher risk of complications and death due to pregnancy than other women [1]. Skilled care before, during and after childbirth can save the lives of women and newborn babies. Between 1990 and 2015, maternal mortality worldwide dropped by about 44% [1]. United Nations Sustainable Development Goal aims to reduce the global maternal mortality ratio (MMR) to less than 70 per 100000 live births by 2030 [1]. Tamil Nadu, a southern state in India, had already achieved this target reaching 66 per 100000 live births during 2014- 16. However, in Virudhunagar district of Tamil Nadu, the MMR was 88 per 100000 live births during 2017-18. It was essential to know the causes of maternal deaths in the district, to formulate policies and interventions at the district level. Hence, we did this study to describe maternal deaths and its causes in Virudhunagar district, Tamil Nadu during 2013-2018.

**Methods**

We did a cross-sectional analysis of maternal deaths reported in Virudhunagar district during the period 2013-2018. Data were extracted from the state Health Management Information System (HMIS) and verbal autopsy reports. Data were abstracted and analysed using MS Excel. We defined the maternal mortality ratio as the number of women who die from any cause related to or aggravated by the pregnancy or its management during pregnancy and childbirth or within 42 days of pregnancy termination, irrespective of the duration and site of the pregnancy, per 100,000 live births. We calculated MMR by year, health unit district (HUD), area of residence and caste. We described the distribution of maternal deaths by age group, the order of pregnancy, level of delay, source of referral and comorbid conditions. We calculated observed cause-specific mortality rate by dividing the number of maternal deaths reported due to a particular cause by the total number of pregnant women observed and multiplying by 100000. We used the cause-specific mortality rate based on systemic analysis by WHO [2], as the expected cause-specific mortality rate.

**Results**

During the period 2013 to 2018, 87 maternal deaths were reported in the district, of which 53 maternal deaths occurred in Sivakasi HUD and 34 in Virudhunagar HUD. The MMR in the district was the highest during 2014-15 with 108 per 100000 live births. The year 2017-2018 witnessed an MMR of 95 per 100000 live births. Sivakasi HUD reported the highest MMR of 105 per 100000 live births during 2017-2018. Out of eleven blocks, seven blocks in the district were consistently having high MMR than the state average since 2014. Overall maternal mortality was high in rural areas during 2013-2018. MMR was higher among the scheduled caste community (49 per 100000 live births) than other castes (31 per 100000) during 2013-18. About 43% and 33% of the maternal deaths reported belonged to age group 25-29 and 20-24 years, respectively. Regarding the order of pregnancy, 45% of deaths occurred during the first order, 39% from the second-order, and 16% from higher-order pregnancies. About 77% of maternal deaths occurred during the postpartum period. About 65% of the maternal deaths that occurred in the postpartum period were following caesarean deliveries. The maternal deaths due to transit delay were 45%. Delay 1 which was due to recognition of danger signs was about 25%, Delay 2 which was due to delay in transportation of postpartum mother to the treatment facility was about 40%, and Delay 3 which was due to delay in initiation of treatment at the treatment facility was about 35%. About 42% of maternal deaths were among the mothers with an emergency referral from private hospitals, 35% among emergency referrals from government hospitals and 23% among emergency referrals from primary health centres. Of the 87 maternal deaths reported, 18% had hypertension, 14% had haemorrhage, 13% had embolism, 10% had cardiac disorders, 8% had disseminated intravascular coagulation, 8% had respiratory disorders, 7% had liver disorders, and 6% had anaemia. The cause-specific mortality rate was the highest for pregnancy-induced hypertension (PIH) with 13 per 100000 pregnant women (Table 1).

**Conclusions**

Of the two Health unit districts in Virudhunagar district, Sivakasi Health unit district had the highest MMR. Out of eleven blocks, seven blocks in the district consistently reported MMR higher than the state average. Maternal deaths were more common in rural areas and during the postpartum period. Pregnancy-induced hypertension was the most common cause of maternal deaths. The reason for postpartum maternal deaths both at the facility level & community level needs to be identified. High MMR reported in seven blocks, and the reasons for pregnancy-induced hypertension-related maternal deaths need further investigation

**References**

1. World Health Organization. Maternal mortality [Internet]. Available from: https://www.who.int/news-room/fact-sheets/detail/maternal mortality. [Accessed 2020 Feb 29].

2. Say L, Chou D, Gemmill A, Tunçalp Ö, Moller A-B, Daniels J, et al. Global causes of maternal death: a WHO systematic analysis. The Lancet Global Health. 2014 Jun 1;2(6):e323–33.

**Table 1 (abstract A28).** The cause-specific mortality rate of maternal deaths in Virudhunagar district, Tamil Nadu, 2013-2018

Causes of maternal death	Cause-specific mortality rate /100000 pregnant women (Observed)	Cause-specific mortality rate/100000 pregnant women (Expected) [2]
**Sepsis**	5.7	3.8
**Embolism**	9.0	8.5
**Haemorrhage**	9.8	21.2
**Pregnancy-induced hypertension**	13.0	10.3

## A29 Measles Outbreak Investigation among Children in Hamlet A*,* Nawada District, Bihar, India, December 2018

### Sanjay K. Singh^1^, Ismeet Kaur^1^, Kevisetuo A. Dzeyie^1^, Sushma Choudhary^2^, Pankaj Bhatnagar^1^

#### ^1^National Public Health Surveillance Project, World Health Organization Country Office, New Delhi, India; ^2^South Asia Field Epidemiology and Technology Network, New Delhi, India

##### **Correspondence:** Sanjay K. Singh (srcchamparaneast@npsuindia.org)

**Background**

Measles primarily affects children under 5 years of age [1]. Severe measles is more likely to occur among malnourished children, particularly those with insufficient vitamin A, or a weak immune system [2]. Although a safe and cost-effective vaccine is available, children continue to die from measles each year. In 2018, an estimated 140,000 measles deaths occurred globally, with most deaths occurring among children under 5 years of age [3]. In the same year, India reported 26,324 suspected measles cases from 1,518 suspected measles outbreaks and 139 deaths [3]. Of these, Bihar state reported 5,414 suspected measles cases, 267 suspected measles outbreaks and 25 deaths. The measles incidence rate in Bihar was 36/1,000,000 population in 2018, which was almost 3 times higher than the national incidence rate of 13/1,000,000 [4].

In December 2018, the vaccine-preventable disease surveillance system of Nawada district, Bihar state, reported a suspected measles outbreak in Hamlet A (sub-village area), Village A. We conducted an outbreak investigation to describe the epidemiology of the outbreak, identify the risk factors of the illness, assess immunization coverage with the measles-containing vaccine (MCV) in the affected area, and provide evidence-based recommendations.

**Methods**

We defined a probable measles case as fever and maculopapular rash with cough, coryza or conjunctivitis in a resident of Village A, Nawada district from 7 October 2018 to 7 February 2019. A confirmed case was defined as a probable case with detection of measles specific IgM by enzyme-linked immunosorbent assay (ELISA). Cases were identified by reviewing the outpatient, emergency department and inpatient hospital records of Narhat primary health centre. Records from private practitioners in Village A were also reviewed to find cases. Active case search through a house-to-house survey in Hamlet A of Village A, which had 91 households and 103 children ≤15 years of age was also conducted.

We also conducted a retrospective cohort study among children ≤15 years of age in Hamlet A and assessed immunization coverage among children <5 years of age. Information was collected on demographics, clinical presentation and immunization status by interviewing cases or caregivers using a semi-structured questionnaire. Five blood samples from cases within 28 days of rash onset were collected and tested for measles and rubella IgM by ELISA per Government of India measles surveillance and outbreak investigation guidelines [5].In addition, the routine immunization system of Village A including the micro-plan and the availability of logistics and cold chain was also assessed. We calculated relative risk (RR) with 95% confidence intervals (CI).

**Results**

Twenty-one probable cases (52% female) were identified, with a median age of 5 years (range: 1–15 years). The measles attack rate was 20% (21/103), with the highest attack rate among the 5–9 year-old age group at 31% (9/29). Among the cases, diarrhoea was reported by 17 (81%) of cases, and 10 (48%) had pneumonia. Cases started on 22 November 2018, peaked on 6 December 2018, and the last case was reported on 27 December 2018. The index case arrived at Hamlet A from Village B on 20 November 2018 and developed fever and rash on 22 November 2018. Village B reported an ongoing measles outbreak from 14 October to 1 December 2018.

In the retrospective cohort study of 103 children ≤15 years in Hamlet A, the following factors were associated with illness: household with less than three rooms and a kitchen (RR 12.7; 95% CI 1.8–90.9), family size >6 (RR 5.7; 95% CI: 2.31–4.3), kutcha house (i.e., house made of wood, mud, straw and dry leaves) (RR 5.44; 95% CI 1.7–14.3), and missing first MCV dose (RR 3.26; 95% CI: 1.6–6.8) (Table 1). Among 45 children aged 12–59 months, 13 (29%) had a routine immunization card, 20 (44%) received the first MCV dose, 3 (7%) received a second MCV dose, and none received a dose of vitamin A. None of the 103 children in the cohort <15 years were fully immunized for their age. All five blood samples tested positive for measles specific IgM. The most common reasons for none or partial immunization were fear of adverse event following immunization or injection and caretakers not able to take the child for immunization sessions.

In the routine immunization system assessment, we found that a micro-plan was available but had not been updated for more than a year. Nine (75%) of 12 routine immunization sessions were held at the Anganwadi or child health centre in Village A from 1 April 2017 to 31 March 2018. However, there were no specific sessions planned for Hamlet A. The vaccine supply chain was functional with regular cold chain monitoring and an adequate vaccine supply.

**Conclusions**

We confirmed a measles outbreak at Hamlet A with low measles immunization and vitamin A coverage. Missing the first dose of MCV and the large family size was associated with measles. We recommended additional outreach immunization sessions, strengthening the delivery of routine immunization to missed children, and administration of vitamin A to children in Hamlet A. To address community fears about immunization, awareness sessions for mothers at *Anganwadi* centres and dissemination of information on immunization by frontline health workers during home visits should be promoted.

**References**

1. Centers for Disease Control and Prevention (CDC). Measles: Epidemiology of Vaccine Preventable Diseases, 2019. [https://www.cdc.gov/vaccines/pubs/pinkbook/meas.html]. Accessed on 25 February 2020.

2. Mina MJ, Kula T, Leng Y, Li M, de Vries RD, Knip M, et al. Measles virus infection diminishes preexisting antibodies that offer protection from other pathogens. *Science* 2019; 366: 599. doi:10.1126/science.aay6485.

3. World Health Organization. Measles deaths estimates. [https://www.who.int/news-room/detail/05-12-2019-more-than-140-000-die-from-measles-as-cases-surge-worldwide]. Accessed on 25 February 2020.

4. World Health Organization (WHO) and Ministry of Health and Family Welfare (MOHFW), Government of India (GOI). Measles and rubella surveillance bulletin, March 2018. New Delhi: WHO and MOHFW, GOI, 2018.

5. Ministry of Health and Family Welfare (MOHFW), Government of India (GOI). Measles surveillance and outbreak investigation: field guide. New Delhi: MOHFW, GOI, September 2006.

**Table 1 (abstract A29).** Risk factors for measles in Hamlet A, Nawada District, Bihar, India, December 2018 (N=103)

Risk Factor	Frequency of exposure among measles cases N=21	Frequency of exposure among non-cases N=82	Risk Ratio	95% Confidence Interval
	n (%)	n (%)		
House <3 rooms	20 (95)	39 (48)	14.91	2.0–106.9
Family >6 persons	16 (76)	21 (26)	5.70	2.2–14.3
*Kutcha* house*	18 (86)	36 (44)	5.44	1.7–17.3
No MCV1**	5 (24)	4 (5)	3.26	1.5–6.8
Illiterate mother	14 (67)	36 (44)	1.43	0.6–3.2
Female	14 (67)	38 (46)	1.55	0.6–3.5

*Kucha = A house made up of wood, mud, straw and dry leaves

** MCV1 = measles-containing vaccine first dose

## A30 Evaluation of Adverse Events Following Immunisation surveillance system, Vidisha, Madhya Pradesh, India, March 2018

### Abhishek Jain^1^, Sushma Choudhary^2^, Pankaj Bhatnagar^1^

#### ^1^National Public Health Surveillance Project, World Health Organization, Delhi, India; ^2^South Asia Field Epidemiology and Technology Network, Delhi, India

##### **Correspondence:** Abhishek Jain (drabhishekjain@yahoo.com)

**Background**

The Universal Immunization Program of India is one of the largest public health programmes in the world. It protects against 12 vaccine-preventable diseases to 26 million children and 29 million pregnant women through 12 million immunisation sessions throughout the country each year[1]. Adverse events sometimes follow immunisation. In India in 2017, of the 5.4 million children who missed vaccine doses, 28% missed only because of the fear of adverse events [2]. To improve the quality of immunisation; detect, correct, and protect immunisation errors; and gain public trust, in 1988, the Government of India established an Adverse Effects Following Immunization (AEFI) surveillance system [3]. In 2017, approximately 1,600 AEFIs were reported by the surveillance system nationwide. Madhya Pradesh, one of India’s largest states, provides vaccine to 1.5 million children every year. In 2017, Madhya Pradesh reported 54 severe or serious AEFIs. We assessed the AEFI surveillance system in Vidisha district, Madhya Pradesh, to describe the system, assess it against selected attributes, and provide evidence-based recommendations to strengthen the AEFI surveillance system.

**Methods**

During April 2017–March 2018, we conducted the study in a randomly selected district, Vidisha, from among the 51 districts in Madhya Pradesh. Within Vidisha (population 1,662,684), we selected 2 of the 7 blocks: Pipalkheda (population 367,064) and Gyaraspur (population 146,358), using simple random technique. We conducted a cross-sectional study in March 2018. We used quantitative (review of records and reports) and qualitative (mainly interviews) methods to evaluate AEFI in Vidisha. We evaluated the AEFI surveillance system using the U.S. Centers for Disease Control and Prevention (U.S. CDC) guidelines. We assessed the system against the following attributes as defined by U.S. CDC [4].

1. Simplicity: the structure and ease of operation of an AEFI surveillance system.
2. Flexibility: the capacity of the system to adapt to changing information needs or operating conditions with little additional time, personnel, or allocated funds.
3. Acceptability: the willingness of persons and organisations to participate in the AEFI surveillance system.
4. Sensitivity: the proportion of AEFIs detected by the surveillance system.
5. Data quality: the completeness and validity of data recorded in the AEFI surveillance system.
6. Representativeness: the AEFIs distribution in the population by time, place, and person.
7. Timeliness: the AEFI surveillance system’s speed of response.
8. Stability: the ability of the AEFI surveillance system to collect, manage, and provide data properly and without failure.

We interviewed the District Immunization Officer, 4 Medical Officers, the District Data Handler, two nodal persons at the reporting units, and eight axillary nurse-midwives to collect data for evaluation of 2 qualitative attributes (simplicity, acceptability). We reviewed records (AEFI register, line list, investigation and weekly reports, case reporting forms) and the AEFI reporting Web portal (SAFE-VAC, Surveillance and Action for Events following immunisation) to evaluate the flexibility, sensitivity, data quality, timeliness, stability, and representativeness of the system. We entered and analysed data in Microsoft Excel (https://microsoft.com). We calculated each of the indicators as proportions for the attributes.

**Results**

We assessed the AEFI surveillance structure and information flow (Table 1). If a person with an AEFI contacts a health worker or Medical Officer, the health worker or Medical Officer records the adverse event in the AEFI tracking register at the block and sub-block levels. The Medical Officer classifies a registered AEFI into 1 of 3 categories: minor, severe, or serious. Severe and serious AEFIs are reported each week to the district in reporting form H002. The District Immunization Officer should investigate them within 48 hours after notification and filing of case reporting forms. The district also reports the AEFI each week through the D001 reporting form and subsequently to the state. The District Immunization Officer further investigates reported AEFIs and files a Preliminary Case Investigation Form within ten days and Final Case Investigation Form within 70 days. The district uploads all these documents to SAFE-VAC. The State Routine Immunization cell conducts a causality assessment, with help from the state AEFI committee. Within 100 days, the final classification is done by the national AEFI committee [3,5]. In Vidisha, five severe AEFIs were reported. Four were from the two assessed blocks, and two were reported by blocks in the weekly report. None of the four classified AEFIs in these two blocks was investigated. None of the five was uploaded to SAFE-VAC. The district surveillance unit investigated four additional AEFIs that were not documented at the block level. All 11 government and three private reporting units in the district reported to the system. AEFI reporting ratio (i.e., the number of AEFI reported/100,000 surviving infants) was 5 (expected 4.5). All 5 case reporting forms were filed on time. All Preliminary Case Investigation Forms were filled and submitted to the state on time, and three (60%) of the Final Case Investigation Forms were submitted on time. Although reporting units submitted 616 of 728 (85%) of the weekly reports on time, only 67 (9%) were available at the district. Among the 12 healthcare personnel interviewed, 3 (25%) were aware of AEFI reporting flow. None of the Medical Officers could classify AEFIs. Standard AEFI reporting and investigation forms were available and used at both block and district levels.

**Conclusions**

The AEFI surveillance system in Vidisha is sensitive, representative, and timely. We recommended training field workers and Medical Officers to improve simplicity and regular district meetings and reviews with block staff to avoid data discrepancies.

**References**

1. Immunization Division, Ministry of Health and Family Welfare, Government of India. Universal Immunization program. Evolution of the programme. https://main.mohfw.gov.in/sites/default/files/5628564789562315.pdf. [Accessed 2020 June 19].

2. Ministry of Health and Family Welfare, Government of India. Roadmap for achieving 90% full immunisation coverage in India. A guidance document for the states. https://nhm.gov.in/New_Updates_2018/NHM_Components/Immunization/Guildelines_for_immunization/Roadmap_document_for_90%25_FIC.pdf. [Accessed 2020 June 19].

3. Ministry of Health and Family Welfare, Government of India). Revised AEFI guidelines: executive summary. https://main.mohfw.gov.in/sites/default/files/Revised%20AEFI%20Guidelines%20Execute%20Summary.pdf. [Accessed 2020 June 19].

4. U.S. Centers for Disease Control and Prevention. Updated guidelines for evaluating public health surveillance systems. Recommendations from the Guidelines Working Group. MMWR Morb Mortal Wkly Rep. 2001: 50(RR13):1–35.

5. Ministry of Health and Family Welfare, Government of India. Operational guidelines for quality assurance in public health facilities 2013. http://www.rrcnes.gov.in/quality%20Assurance/Operational%20Guidelines%20on%20Quality%20Assurance%20%28Print%29.pdf. [Accessed 2020 June 19].

**Table 1 (abstract A30).** Results of a cross-sectional survey of the AEFI surveillance system, Pipalkheda and Gyaraspur blocks, Vidisha district, Madhya Pradesh, India, March 2018

Attribute and indicator	Numerator	Denominator	Results, n/N (%)
Simplicity			
• Proportion of ANMs aware of AEFI reporting	No. of ANMs aware of case definition and reporting	No. of ANMs Interviewed	2/8 (25)
• Proportion of MOs aware of AEFI reporting	No. of MOs aware of case definition and reporting	No. of MOs interviewed	1/4 (25)
• MOs able to classify AEFIs	No. of MOs able to classify AEFIs	No. of MOs interviewed	0/4 (0)
Flexibility			
• Proportion of reports uploaded to SAFE-VAC	No of reports of AEFIs uploaded to SAFE-VAC	No of reports of AEFIs reviewed	0/15 (0)
• Proportion of AEFI documents sent to state through email	No. of AEFI reports sent through email	No of reports of AEFIs reviewed	13/15 (87)
Acceptability			
• Use of standard formats at all levels	Availability of standard forms at RUs	No. of RUs where record reviewed	3/3 (100)
• Proportion of AEFIs reported by RUs	No. of cases reported in H002	Total AEFIs	2/4 (50)
• Proportion of reported AEFIs investigated	No. of cases investigated	No. of AEFI cases on line list	0/2 (0)
Sensitivity			
• Proportion of AEFI reported against expected	No. of AEFIs reported	No. of surviving infants in Vidisha district	5/45,068 (111/1,000,000)
	No. of AEFIs reported	No. of surviving infants in block Pipalkheda	3/9,948 (301/1,000,000)
	No. of AEFIs reported	No. of surviving infants in block Gyaraspur	1/3,808 (262/1,000,000)
Data quality			
• Proportion of reported persons with symptoms written in AEFI tracking register	No. of reported persons with symptoms in AEFI tracking register	No. of reported persons written in AEFI tracking register	232/232 (100)
• Proportion of AEFIs reported in H002	No. of AEFIs reported in H002	Total AEFIs	2/4 (50)
• Proportion of reported AEFIs investigated	No. of AEFIs investigated	Total AEFIs in line list	0/2 (0)
• Proportion of Investigated AEFIs documented at blocks	No. of investigated AEFIs documented at block	Total AEFIs investigated in block	0/4 (0)
Representativeness			
• Proportion of blocks reported AEFI cases	No. of blocks reporting AEFIs	No. of blocks	3/7 (47)
• Proportion of government RU reporting sending weekly reports	No. of government RUs sending weekly reports	No. of government RUs	11/11 (100)
• Proportion of private. RUs sending weekly reports	No. of private RUs sending weekly reports	No. of private RUs	3/3 (100)
Timeliness			
• Proportion of reports (H002, CRF, PCIF, FCIF) sent to appropriate level within expected timeline	No. of H002 sent	No. of AEFIs with H002 to be filled	616/728 (85)
	No. of CRF sent	No. of AEFIs with CRF to be filled	5/5 (100)
	No. of PCIF sent	No. of AEFIs with PCIF to be filled	5/5 (100)
	No. of FCIF sent	No. of AEFIs with FCIF to be filled	3/5 (60)
Stability			
• Proportion of weekly reports physically present at district	No. of H002 available at district	No. of AEFIs with H002 to be filled	67/728 (9)
• Proportion of blocks having AEFI tracking register,	No of blocks having AEFI tracking register	No. of blocks	2/2 (100)
• H002 booklet and CRF	No of blocks having H002 booklet and blank CRF	No. of blocks	1/1 (100)
• Availability of PCIF and FCIF at district	No of districts having blank PCIF and FCIF available	No. of districts	1/1 (100)

## A31 Assessment of the Measles Surveillance System in East Champaran District, Bihar State, India, April 2017–March 2018

### Md. Subhan Ali^1^, Rajesh Yadav^2^, Kevisetuo A. Dzeyie^1^, Shanmukhappa^3^, Pankaj Bhatnagar^1^, Pauline Harvey^1,4^

#### ^1^National Public Health Surveillance Project, World Health Organization, New Delhi, India; ^2^Division of Global Health Protection, U.S. Centers for Disease Control and Prevention, New Delhi, India; ^3^Department of Microbiology, Mandya Institute of Medical Sciences, Mandya, Karnataka, India; ^4^Global Immunization Division, U.S. Centers for Disease Control and Prevention, Atlanta, Georgia, USA

##### **Correspondence:** Md. Subhan Ali (smomotihari@npsuindia.org)

**Background**

In 2018, India reported 52,308 measles cases and 165 measles deaths, which accounted for 67% of the measles cases and 86% of the measles deaths in the World Health Organization (WHO) South-East Asia Region [1]. The largest number of measles cases and deaths were reported from Uttar Pradesh, Bihar, and Madhya Pradesh [1]. India has a measles elimination goal for 2023 that relies on a robust surveillance system to detect and respond to measles outbreaks [2]. India started measles surveillance in 2005–2006. Bihar state launched measles surveillance in 2011 across 626 health sub-centers. In 2018, Bihar reported 5,236 measles cases (Surveillance Report 2018, WHO State Office Patna, unpublished data). The East Champaran district of Bihar has 28 blocks (administrative sub-units), and the district reported 17 measles outbreaks in 2018 compared to seven in 2017 (Surveillance Report 2018, WHO State Office Patna, unpublished data). We evaluated the measles surveillance system in the East Champaran district to identify the system's strengths and weaknesses and provide recommendations to improve the health system and achieve the elimination target.

**Methods**

We assessed the measles surveillance system in East Champaran district between April 1, 2017, and March 31, 2018, using the U.S. Centers for Disease Control and Prevention guidelines for evaluating surveillance systems [3]. We adopted the purposive sampling technique to include the four reporting units: a district hospital, a private hospital, a primary health center, and a health sub-center so that the participation of key informants from various levels of health facilities. We interviewed the following cadres of key informants: surveillance officers, nodal persons, frontline health workers, and private practitioners. We reviewed district surveillance records to assess simplicity, data quality, timeliness, acceptability, representativeness, and overall system usefulness. We reviewed forms received by the district office from the 28 block-level primary health centers. We also analyzed the number of measles weekly forms (i.e., WHO vaccine-preventable disease [VPD] health form 002 or WHO VPD-H002) sent by the primary health center and private hospital to the East Champaran district office and the number of complete forms during the one year under review.

**Results**

Measles surveillance in East Champaran district is a passive surveillance system, which has been functional since 2011. The reporting units, comprised of both government and private health facilities, report suspected measles cases to the nodal person or surveillance officer at the block-level primary health center by Monday. The nodal person or surveillance officer enters the details of the suspected cases on the WHO VPD-H002 form. After the WHO VPD-H002 form is signed by the medical officer-in-charge of the block primary health center, it is sent to the district immunization officer and WHO surveillance medical officer by Tuesday. The district immunization officer compiles the measles data into a monthly report to flag potential outbreaks for investigation. The district holds a weekly surveillance meeting where all WHO VPD-H002 forms are reviewed and compiled into the WHO VPD-D001 form. The district sends the WHO VPD-D001 form to the state by Tuesday evening. The state reviews and sends it to the central surveillance unit by Wednesday.

We interviewed a total of 16 staff, and all were aware of the mechanism for reporting cases and the measles case definition (Table.1). Of those interviewed, 10 (63%) were aware that all age groups were included in the case definition. The reporting units used both paper and mobile phones to report to the block-level nodal person or surveillance officer. The 28 blocks submitted all weekly forms; 96% were complete, and all were submitted on time (Table.1). The district held 98% of weekly surveillance meetings. No monthly measles outbreak reports were available for review. Four percent of private health facilities reported measles data during the review period.

**Conclusions**

Based on key informant interviews and review of surveillance forms, the East Champaran district measles surveillance system was simple, as illustrated by users' knowledge level and awareness. It was acceptable to users, as evidenced by reporting regularity and weekly meetings. The measles surveillance system produced complete and timely quality data. Only a small number of private health facilities were represented in the measles surveillance system. Overall, the system was useful. The data collected were used to identify 12 potential measles outbreaks during the review period, which were investigated by district health authorities. We recommended strengthening measles surveillance by specific strategies. We recommended regular refresher training of frontline and private facility health staff, increasing the number of private facilities reporting data, and initiating a periodic evaluation of measles data by surveillance officers to inform the program and identify areas of weakness for corrective interventions.

**References**

1. Global Measles and Rubella Update. 2018. https://www.who.int/immunization/monitoring_surveillance/burden/vpd/surveillance_type/active/Global_MR_Update_November_2018.pdf. Accessed January 11 2020.

2. Measles and rubella in the South-East Asia. World Health Organization. https://www.who.int/southeastasia/health-topics/measles. Accessed January 15 2020

3. German RR, Lee LM, Horan JM, Milstein RL, Pertowski CA, Waller MN; Guidelines Working Group Centers for Disease Control and Prevention (CDC). Updated guidelines for evaluating public health surveillance systems: recommendations from the Guidelines Working Group. MMWR Recomm Rep. 2001 Jul 27;50(RR-13):1-35;

**Table1 (abstract A31).** Number and proportion of indicators describing attributes of the measles surveillance system in East Champaran District, Bihar, India, April 2017–March 2018

Attribute	Indicator	n	N	%
Simplicity	Aware of case definition	16	16	100
	Aware of age groups in definition	10	16	63
	Aware of WHO VPD-H002 and how to report	16	16	100
Acceptability	Weekly WHO VPD-H002 forms submitted by block primary health centers to district	1456	1456	100
	District surveillance meetings	51	52	98
	Outbreak reports available at district	0	12	0
Data Quality	WHO VPD-H002 forms with complete information	1398	1456	96
Timeliness	WHO VPD-H002 form received on time by district	1456	1456	100
Representativeness	Private health facilities reporting	8	217	4
	Measles outbreaks reported by private health facilities	8	12	67

WHO VPD-H002 = World Health Organization vaccine-preventable disease health form number 002

## A32 Measles outbreak investigation in Thiruvananthapuram city, Kerala, India, 2019

### Kolar Neelakanta Arun Kumar^1^, Shobha Malini^2^, Anoop Velayudhan^3^, Rajesh Yadav^3^, Kevisetuo Anthony Dzeyie^1^, Pankaj Bhatnagar^1^, Pauline Harvey^1,4^

#### ^1^National Public Health Surveillance Project, World Health Organization Country Office, New Delhi, India; ^2^Department of Community Medicine, S.L.N. Medical College & Hospital, Koraput, Odisha, India; ^3^Division of Global Health Protection, Center for Global Health, U.S. Centers for Disease Control and Prevention, New Delhi, India; ^4^Global Immunization Division, Center for Global Health, U.S. Centers for Disease Control and Prevention, Atlanta, Georgia, USA

##### **Correspondence:** Kolar Neelakanta Arun Kumar (srcchennai@npsuindia.org)

**Background**

Measles is an acute viral disease with a safe and effective vaccine. However, measles remains a major cause of morbidity and mortality in most developing countries, including India [1]. Measles outbreaks pose serious challenges to elimination efforts and indicate where health systems need strengthening [2]. In 2010, the Government of India introduced second-dose measles vaccination for children 16–24 months of age [3]. India has targeted measles elimination by 2023 [4]. To meet elimination goals, India has focused on strengthening surveillance and increasing coverage of measles-containing vaccine (MCV) to >95% [4]. Despite reportedly having 99% first-dose MCV coverage for 2018–19, Thiruvananthapuram city in Kerala state, India, reported a suspected measles outbreak during January 2019. We investigated to describe the outbreak epidemiology, calculate vaccine effectiveness, and provide recommendations to prevent additional measles cases.

**Methods**

We defined a suspected case-patient of measles as fever and maculopapular rash with cough or coryza or conjunctivitis in a resident of Thiruvananthapuram city during January 1–May 31, 2019. We defined a confirmed measles case-patient as fever and maculopapular rash with cough or coryza or conjunctivitis and a positive measles laboratory test in a resident of Thiruvananthapuram city during January 1–May 31, 2019. We searched for case-patients from outpatient and inpatient registers at Perumal Pillai Ratnaswamy Hospital and Urban Primary Health Centre–Vattiyoorkavu. We reviewed Integrated Disease Surveillance Programme reports for January–May 2019 of Urban Primary Health Centre–Vattiyoorkavu area and conducted house-to-house searches in areas which reported clustering of three or more suspected cases. We collected demographic information, clinical and vaccination history (by card and caregiver report), and post-rash vitamin A dose administration information using a semi-structured questionnaire. Serum was tested for measles by IgM enzyme-linked immunosorbent assay.

We also assessed reasons for measles non-vaccination in the eligible beneficiary age group. We calculated vaccine effectiveness using the following formula:

$$ \frac{\mathrm{Attack}\kern0.5em \mathrm{rate}\kern0.5em \mathrm{in}\kern0.5em \mathrm{unvaccinated}\hbox{-} \mathrm{Attack}\kern0.5em \mathrm{rate}\kern0.5em \mathrm{in}\kern0.5em \mathrm{vaccinated}}{\mathrm{Attack}\kern0.5em \mathrm{rate}\kern0.5em \mathrm{in}\kern0.5em \mathrm{unvaccinated}} $$

We reviewed the Urban Primary Health Centre–Vattiyoorkavu routine vaccination system to assess human resource availability, vaccine supply, training, and microplanning.

**Results**

Thiruvananthapuram city, with a population of approximately 850,000, reported 3 measles cases in 2017 and 4 in 2018. We identified 47 measles case-patients, 23 (49%) suspected and 24 (51%) confirmed, in Thiruvananthapuram city during January 1–May 31, 2019. Of these, 29 (62%) occurred in males. The median age of case-patients was 19 years (range: 6 months–65 years). Case-patients were distributed by age group as follows: 5 (11%) in infants aged <9 months, 8 (17%) in children aged 9 months–4 years, 7 (15%) in children aged 5–14 years, 21 (45%) in persons aged 15–24 years, and 6 (13%) in persons aged ≥25 years. The overall attack rate was 6 cases per 100,000 population. The age-specific attack rates per 100,000 population were 21 in children aged ≤4 years, 5 in children aged 5–14 years, 16 in persons aged 15–24 years, and 1 in persons aged ≥25 years. The epidemic curve by date of rash onset showed a rise in the number of cases during January–February (23 cases) (Figure). No deaths were reported. Cough was the predominant clinical presentation for 40 (85%) of cases, followed by coryza in 35 (74%), and conjunctivitis in 29 (62%). Enlarged lymph nodes were observed in 3 (6%) of cases, diarrhoea in 5 (11%), and pneumonia in 1 (2%). Post-rash vitamin A prophylaxis was administered to 74% (35/47) of cases. For the 39 cases for whom serum samples were tested, 24 (62%) were IgM positive. The Hindu community was affected (31, 66%) in similar proportion (69%) to their population representation. Eight wards in Thiruvananthapuram city reported 3 or more cases. Nine (19%) cases were geographically clustered in VKP Nagar ward, which had a population of 2,767 for an incidence of 33 cases per 10,000. The other wards reporting three or more cases had an incidence of 5 cases per 10,000. First-dose MCV coverage among persons of 9 months–24 years of age was 36% (13/36), and second-dose MCV coverage was 14% (5/36). The attack rate was 2.3% for unvaccinated persons and 1.3% for vaccinated persons. Vaccine effectiveness was 43%. Measles vaccination among age-eligible children (9 months–15 years) was 27% (4/15). The reasons for non-vaccination were refusal because of fear of adverse events after vaccination (36%, 4/11) and lack of a nearby vaccination session (36%, 4/11). The health system assessment showed that the average population served by each auxiliary nurse midwife ranged from 13,000 to 16,000 persons (≤10,000 persons recommended). The assessment also demonstrated a lack of refresher training for routine vaccination, incomplete micro-plans and lack of lists of children due for vaccination, and suboptimal vaccination session planning that limited service delivery.

**Conclusions**

We confirmed a measles outbreak in Thiruvananthapuram city in which most cases occurred in persons ≥15 years of age. This outbreak was unusual because measles generally occurs in children 6 months to 4 years of age. The older age distribution of case-patients in this outbreak might be attributable to non-vaccination or reduction in level of protective antibodies or both. Only one third of cases had received a single dose of measles vaccine during childhood, and two thirds either refused vaccination because they feared an adverse event or were unaware of vaccination delivery sites, which could have contributed to the outbreak. Our investigation showed that proportion with history of measles vaccination was low among cases with an epidemiologic shift toward higher age groups. We recommended one dose of vitamin A for all cases and prophylactically for all children <15 years in Thiruvananthapuram city. We recommended active searches for additional case-patients for the following three months, prioritizing vaccination refresher training to auxiliary nurse midwives, and increasing community awareness activities to create vaccination demand. Based on these recommendations, health authorities administered a dose of vitamin A to case-patients who had not already received one, intensified community awareness of vaccination activities, and increased the frequency of vaccination sessions. No new measles case-patients were reported after May 2019.

**References**

1. World Health Organization. Global measles and rubella update. November 2018 https://www.who.int/immunization/monitoring_surveillance/burden/vpd/surveillance_type/active/Global_MR_Update_November_2018.pdf?ua=1. [Accessed 2019 April 30].

2. Gastañaduy PA, Banerjee E, DeBolt C, Bravo-Alcántara P, Samad SA, Pastor D, et al. Public health responses during measles outbreaks in elimination settings: strategies and challenges. Hum Vaccin Immunother. 2018; 14:2222–2238.

3. Verma R, Khanna P, Bairwa M, Chawla S, Prinja S, Rajput M. Introduction of a second dose of measles in national immunization program in India: a major step towards eradication. Hum Vaccin. 2011; 7:1109–1111.

4. Bavdekar SB, Karande S. Elimination of measles from India: challenges ahead and the way forward. J Postgrad Med. 2017; 63:75–78.

**Fig. 1 (abstract A32).**
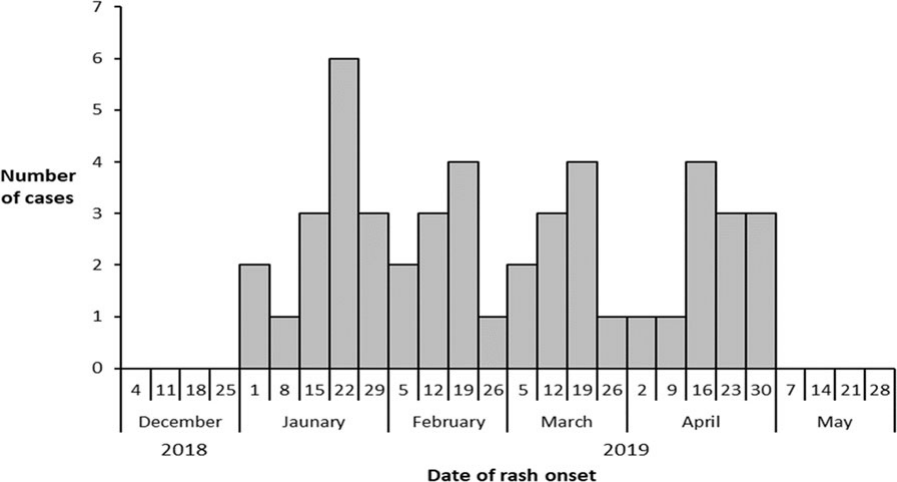
Measles cases reported by date of rash onset, Thiruvananthapuram city, Kerala, India, January 1–May 31, 2019 (N = 47)

## A33 Investigation of the Rubella outbreak, Manawar urban, Dhar district, Madhya Pradesh, India, 2018–2019

### Bhavani Gunta^1^, Ismeet Kaur^1^, Sushma Choudhary^2^, Kevisetuo A Dzeyie^1^, Pankaj Bhatnagar^1^

#### ^1^National Public Health Surveillance Project, World Health Organization Country Office, New Delhi, India; ^2^South Asia Field Epidemiology and Technology Network, New Delhi, India

##### **Correspondence:** Bhavani Gunta (smovishakapatnam@npsuindia.org)

**Background**

Rubella is a contagious viral illness that occurs most often in children and young adults. In a pregnant woman, rubella can cause congenital rubella syndrome, which is vaccine-preventable [1]. India contributed 2,328 (51%) of the 4,533 cases reported from the South-East Asia Region in 2018 [2]. India aims to eliminate measles and rubella by 2023 by strengthening its measles-rubella (MR) surveillance and MR immunization in children as its vital strategy [3]. In India, the MR vaccine is administered in 2 doses to children <5 years of age [4]. On June 17, 2019, a suspected measles outbreak was flagged in Manawar urban, Dhar district, Madhya Pradesh, based on MR surveillance guidelines [5]. We investigated the outbreak to describe the epidemiology and recommend evidence-based prevention and control measures.

**Methods**

We defined a suspected case as fever and rash during February 20–September 3, 2019, in a resident of Manawar block of Dhar district. We defined a probable case as fever and maculopapular rash accompanied by cough, coryza, or conjunctivitis during the period in a resident of Manawar urban area. A confirmed case was a suspected or probable case for which serum was reactive for rubella IgM by enzyme-linked immunosorbent assay. To detect additional cases, we interviewed the medical officer, health staff, and auxiliary nurse midwives of Community Health Centre (CHC) Manawar and reviewed outpatient records, weekly Vaccine-Preventable Disease surveillance reports, and Integrated Disease Surveillance Programme (IDSP) reports for Dhar during February–September. We interviewed private practitioners in the area to determine whether persons with fever and rash sought care and whether the practitioners had reported them to CHC. Because of the vast urban area, we searched for cases in only three wards. We searched house to house for cases in wards 1, 2, and 12 during July 11–13. We collected information about demographic and clinical characteristics, immunizations received, and laboratory tests conducted. We entered and analyzed data using Epi Info 7.2 (https://www.cdc.gov/epiinfo/index.html ) and Microsoft Excel (https://www.microsoft.com ) software to calculate frequencies and proportions. Serum samples from probable case-patients were collected within 0–28 days after rash onset and sent to the Department of Microbiology, Gandhi Medical College, Bhopal, to detect rubella IgM. We assessed the cold-chain system and vaccine and logistics management through interviews of the CHC Medical Officer, Health Extension Officer, and pharmacist; verified registers in cold-chain point; and assessed human resources' availability against sanctioned positions. We also reviewed the Universal Immunization Programme's micro plan at the CHC and Immunization registers using a checklist. We also assessed the immunization status of 10 randomly selected children at the session site. We reviewed immunization coverage reports of CHC Manawar from the Health Management Information System portal for April 2018–March 2019 to determine the coverage of MCV1 and MCV2 during routine immunization session, and MR containing vaccine during catch-up campaigns.

**Results**

We surveyed a total of 1,459 houses (population 7,359). Although frontline workers were unaware of persons in the community who had fever and rash, we identified 22 suspected cases, of which eight were probable cases. The remaining suspected cases appeared clinically to be chickenpox. Five out of eight (62%) probable cases occurred in boys. The median age of probable case-patients was 11 months (range 4–84 months). Among the eight children with probable rubella, five (62%) had cough and coryza, two (25%) had conjunctivitis and itching, and three (37%) had diarrhoea. All eight children visited a health facility. Four (80%) of the five eligible children received MCV1; 1 of 2 (50%) received MCV 2, and 1 of 3 (33%) received an MR dose during the campaign. Five of eight received two doses of vitamin A after their rash resolved. Two of eight serum samples tested positive for rubella IgM. During April 2018–March 2019, a total of 264 (98%) of 270 planned outreach immunization sessions were held. Since the introduction of MR vaccine on March 15, 2019, vaccine shortages have occurred for 2–4 days at the end of each month. Vitamin A is not always available, and its supply is frequently inadequate. Three of five auxiliary nurse midwife positions were vacant for >1 year. Alternatively, adjacent rural auxiliary nurse midwives were deployed on the first Monday and every Wednesday of each month to conduct immunization sessions in Manawar urban.

**Conclusions**

This laboratory-confirmed rubella outbreak among children <5 years of age was probably attributable to suboptimal coverage of routinely scheduled immunizations sessions and MR campaigns. None of the cases was reported by frontline workers. Patients with fever and rash visited private practitioners who did not report cases to CHC. We also found that 14 (64%) of the 22 suspected cases were chickenpox about which the health system was unaware. Besides, the supply of vitamin A was inadequate and not always available. Outreach immunization sessions were not monitored to verify survey registers and the listing of children ≤5 years of age in the survey area who were eligible for MCV.

We recommended an active search for cases in other wards and surveillance until no further cases are reported. We also recommended the need for private health facilities to report suspected MR cases.

**References**

1. World Health Organization. Fact sheets. Rubella. https://www.who.int/news-room/fact-sheets/detail/rubella. Accessed April 27 20202.

2. World Health Organization. Global Health Observatory Data Repository (South-East Asia Region). https://apps.who.int/gho/data/view.main-searo.1520_57?lang=en. Accessed June 16 2020.3.

3. World Health Organization. Global measles and rubella update November 2018 https://www.who.int/immunization/monitoring_surveillance/burden/vpd/surveillance_type/active/Global_MR_Update_November_2018.pdf?ua=1. Accessed June 16 2020.

4. World Health Organization. 28th India Expert Advisory Group Meeting for Polio Eradication. 2019 Nov 13–14. New Delhi, India. https://www.who.int/india/news/detail/14-11-2019-meeting-of-the-28th-india-expert-advisory-group-for-polio-eradication. Accessed June 16 2020.

5. Ministry of Health and Family Welfare, Government of India. Universal Immunisation Programme. Measles surveillance and outbreak investigation: field guide. https://www.nhp.gov.in/universal-immunisation-programme_pg. Accessed April 27 2020.

## A34 An outbreak investigation of Kyasanur Forest disease in villages of Shimoga district, Karnataka, India, 2018–2019

### Asish K. Satapathy^1^, Puttaraju A.K. Jetty^1^, D.M. Satishchandra^1^, Rajesh Yadav^2^, Mohan Papanna^2^, Pankaj Bhatnagar^1^, Harvey Pauline^1,3^

#### ^1^National Public Health Surveillance Project, World Health Organization Country Office, New Delhi, India; ^2^Division of Global Health Protection, Center for Global Health, U.S. Centers for Disease Control and Prevention, New Delhi, India; ^3^Global Immunization Division, Center for Global Health, U.S. Centers for Disease Control and Prevention, Atlanta, Georgia, USA

##### **Correspondence:** Asish K. Satapathy (satapathya@who.int)

**Background**

Kyasanur Forest disease (KFD) is a tick-borne viral zoonosis caused by KFD virus. It was first identified in 1957 in a sick monkey from Kyasanur Forest in Karnataka, India(1). Each year in India, 400–500 KFD cases are reported in humans; the estimated case-fatality rate is 2%–5%(2,3). KFD occurs as seasonal outbreaks during December–June with peaks during February–May(4). Vaccination with the formalin-inactivated tissue-culture vaccine has been the primary strategy for preventing KFD(5). We investigated a suspected KFD outbreak reported on November 30, 2018, from Sagara *taluk* (administrative subdivision), Shimoga district, Karnataka. We aimed to describe the epidemiology of the outbreak and identify risk factors for the illness.

**Methods**

We defined a probable KFD case as acute onset of fever or myalgia, without an alternative clinical explanation, in a resident of Shimoga district during November 1, 2018–February 16, 2019, who lived in a ≤5-km radius of a person with confirmed KFD *or* an area with a monkey *or* tick positive for KFD virus *or* in an area with a reported monkey death *or* a known KFD-endemic area. We defined a confirmed case as a probable case for which KFD virus-specific viral RNA was detected by reverse transcription-polymerase chain reaction (RT-PCR) from a blood specimen at a diagnostic reference laboratory. We identified cases through the Integrated Disease Surveillance Programme weekly reporting system. To identify risk factors for illness among persons with confirmed KFD in the most affected villages of Aralagodu and Kargal, we conducted an unmatched case-control study using 95% confidence interval (CI), power of 80%, an odds ratio of 3, 50% exposure among controls, a sample size of 50 cases and 100 controls were estimated using open epi version 3. We selected confirmed cases who were positive for KFD virus by RT-PCR test and controls who were negative by RT-PCR test. Through a semi-structured questionnaire, we captured data on exposures (i.e., visits to farm or forest, cattle handling, exposure to a dead monkey, presence of rodents around the house, a distance of farm or forest from residence, tick bite history, vaccination history), and demographic, clinical, and laboratory data. We collected blood from persons identified as having probable KFD, and we collected data related to KFD vaccination status from the local public health department. We conducted environmental surveillance through tick pool testing and dead monkey reporting in villages and forested areas in collaboration with the local public health and forest departments. Ticks were collected during August 2018–April 2019 by the flagging and dragging method from the villages' perimeters and adjoining fields or forests. We pooled the ticks captured into 10–20 ticks per pool. We calculated the odds ratios (OR) with 95% confidence intervals for risk factors using Epi Info 7.2 software (https://www.cdc.gov/epiinfo/index.html).

**Results**

From November 1, 2018, to February 16, 2019, we identified 1,075 probable KFD cases in Sagara *taluk*, Shimoga district. Of these, 132 (12%) cases were confirmed, and eight persons died for a case fatality rate of 6%. The KFD attack rate in Sagara *taluk* was 6/10,000 (132/206,319). The cases started in November 2018, peaked in February 2019, and gradually slowed until the end of June 2019. Half (534, 50%) of the probable cases occurred in Aralagodu and Kargal villages in Sagara *taluk*. Of the 132 confirmed cases, 74 (56%) occurred in Aralgodu and four (3%) in Kargal, near the forest but had never reported KFD cases previously. The KFD attack rate in Aralagodu was 251/10,000 (74/2,944) and 30/10,000 (4/1,120) in Kargal. Of the 78 confirmed cases, 42 (54%) occurred among females (attack rate: 3%), and 66 (85%) occurred among persons 15–59 years of age (attack rate: 3%). The median age of confirmed cases was 40 years, with a range of 4–81 years. The attack rate in Aralgagodu was highest (460/10,000) for persons aged 31–45 years. Of the 78 KFD-confirmed cases in Aralgodu and Kargal villages, all presented with fever, 85% with myalgia, 77% with headache, 56% with generalized weakness, 31% each with abdominal pain and neck stiffness, and 16 (21%) reported bleeding. Most case-patients were areca nut plantation workers (39%) or farmers (28%). None of the 78 confirmed cases had received KFD vaccination. In the case-control study, factors related to exposure to dead monkeys were most strongly associated with KFD (Table 1). A total of 349 dead monkeys were reported from August 2018 to April 2, 2019. Twenty-one (6%) dead monkeys tested positive for KFD virus RNA by RT-PCR. The first KFD confirmed dead monkey was reported on November 29, 2018. In the entomologic assessment during the same period, 34 (14%) of the 239 tick pools tested positive for KFD.

**Conclusions**

We confirmed the first KFD outbreak from Aralagodu and Kargal villages in Sagara *taluk*, Shimoga district. Adults who had exposure to dead monkeys were at highest risk for KFD. We recommended implementing an awareness program targeting communities near the forest and agricultural fields, focusing on avoiding contact with dead monkeys and using anti-tick measures. We also recommended strengthening intersectoral coordination and preparedness efforts such as tick control measures and at-risk persons' vaccination. In response to the outbreak, mass KFD vaccination of the eligible population (ages 7 to 65 years) occurred on November 30, 2018 (first dose) and December 28–29 (second dose) in the Aralagodu Primary Health Centre catchment area. Information, education, and communication activities for KFD prevention began in Aralagodu and other high-risk Sagara taluk areas on December 24, 2018. Distribution of dimethyl phthalate oil and other anti-tick protection measures began during the first week of January 2019.

**References**

1. WORK TH, RODERIGUEZ FR, BHATT PN. Virological epidemiology of the 1958 epidemic of Kyasanur Forest disease. Am J Public Health Nations Health. 1959 Jul;49(7):869-74. doi: 10.2105/ajph.49.7.869. PMID: 13661478; PMCID: PMC1372906. Available from: https://pubmed.ncbi.nlm.nih.gov/13661478/

2. Pattnaik P. Kyasanur forest disease: an epidemiological view in India. Rev Med Virol. 2006 May-Jun;16(3):151-65. doi: 10.1002/rmv.495. Erratum in: Rev Med Virol. 2008 May-Jun;18(3):211. PMID: 16710839. Available from: https://pubmed.ncbi.nlm.nih.gov/16710839/

3. Centers for Disease Control and Prevention. Fact sheet: Kyasanur Forest disease (KFD). Available from: https://www.cdc.gov/vhf/kyasanur/pdf/factsheet.pdf. Accessed on January 23 2019.

4. National Centre for Disease Control. CD alert. Kyasanur Forest disease: a public health concern. Available from: http://www.idsp.nic.in/WriteReadData/l892s/60398414361527247979.pdf. Accessed on January 23 2019.

5. Kiran SK, Pasi A, Kumar S, Kasabi GS, Gujjarappa P, Shrivastava A, Mehendale S, Chauhan LS, Laserson KF, Murhekar M. Kyasanur Forest disease outbreak and vaccination strategy, Shimoga District, India, 2013-2014. Emerg Infect Dis. 2015 Jan;21(1):146-9. doi: 10.3201/eid2101.141227. PMID: 25531141; PMCID: PMC4285264.

**Table 1 (abstract A34).** Risk factors for Kyasanur Forest disease, Aralagodu and Kargal villages, Shimoga district, Karnataka, India, 2018–2019

Exposure	Number (%)		Odds Ratio (95% CI)
	Case-patients n=50	Controls n=100	
**Location of house**			
<50 metre from forest	45 (90)	66 (66)	4.6 (1.7–12.7)
<50 metre from agricultural fields	41 (82)	54 (54)	4.0 (1.7–8.8)
Working in areca nut plantation	43 (86)	75 (75)	2.0 (0.8–5.1)
**Ticks**			
Tick seen in household/surrounding	44 (88)	74 (74)	2.5 (0.9–6.7)
History of tick bite	27 (27)	23 (23)	4.0 (1.9–8.1)
Presence of rodents in house	32 (64)	35 (35)	3.0 (1.6–6.7)
Dead monkey(s) reported near house	43 (86)	46 (46)	7.2 (2.9–17.6)
Distance from dead monkey(s) to house			
<50 m	28 (56)	29 (29)	5.9 (2.6–13.9)
50–100 m	12 (24)	9 (9)	8.2 (2.8–24.6)
>100 m	10 (6)	62 (7)	(Reference)
No exposure to dead monkey	18 (36)	62 (62)	(Reference)
**Any exposure to dead monkey**	32 (64)	38 (38)	2.9 (1.4–5.9)
Went near death site*	32 (100)	0 (0)	1287.0 (77.4–1384.6)
Cattle or dog(s) went to death site	16 (50)	2 (5)	18.0 (3.6–87.7)
Participated in monkey cremation*	6 (19)	0 (0)	10.1 (1.2–86.9)
Touched dead monkey*	5 (16)	0 (0)	8.3 (0.9–73.3)
No PPE used	1 (3)	1 (1)	1.9 (0.1–19.8)

CI confidence interval; PPE personal protection equipment

*One was added to each cell to calculate OR

## A35 Influenza A (H1N1)pdm09 outbreak in Lucknow District, Uttar Pradesh, India, January–May 2019

### Madhup Bajpai^1^, Rakesh Vishwakarma^1^, Sushma Choudhary^2^, Pauline Harvey^1,3^

#### ^1^National Polio Surveillance Project, World Health Organization, New Delhi, India; ^2^South Asia Field Epidemiology and Technology Network, New Delhi, India; ^3^Global Immunization Division, U.S. Centers for Disease Control and Prevention, Atlanta, GA, USA

##### **Correspondence:** Madhup Bajpai (bajpaim@who.int)

**Background**

In early 2015, influenza A(H1N1)pdm09 caused nearly 30,000 laboratory-confirmed cases and approximately 1,700 deaths in India[1]. In response to the upsurge in cases, the Ministry of Health and Family Welfare and state health departments undertook the following prevention and control activities: strengthening surveillance; providing laboratory support for diagnostic testing; disseminating guidelines for case management; making oseltamivir available through the public health system; and educating the public through regular information, education, and communication activities[2]. In 2018, India reported 15,266 cases and 1,113 deaths from A(H1N1)pdm09, and Uttar Pradesh reported 65 cases and eight deaths. During the last week of December 2018, Lucknow district, Uttar Pradesh, reported an increased number of persons with fever and cough[3]. The district health team began an investigation and instructed hospitals and laboratories to report suspected cases to the Integrated Disease Surveillance Programme (IDSP). We investigated to describe the epidemiologic characteristics of A(H1N1)pdm09 cases and recommend control measures to stop the outbreak and prevent future cases.

**Methods**

We compared the mean numbers of laboratory-confirmed A(H1N1)pdm09 cases during weeks 1–16 for 2016–2018 with the corresponding weeks for 2019. We calculated the mean number of cases for 2016–2018 plus two standard deviations, which we used as the outbreak threshold. We compared the number of cases for each week of 2019 to determine if cases exceeded the outbreak threshold. We defined the suspect case as a person with acute febrile respiratory illness (fever ≥ 38°C) with onset within seven days of close contact with a person who is confirmed case of pandemic influenza A (H1N1) virus infection between December 25, 2018–May 8, 2019[2]. We defined a probable case of influenza A(H1N1)pdm09 as a person with acute-onset fever and cough, sore throat, or runny nose and sought diagnostic testing while a Lucknow resident district between December 25, 2018–May 8, 2019. We defined a confirmed case as a probable case with a positive reverse transcription-polymerase chain reaction diagnostic test result for influenza A (H1N1)pdm09. We obtained a line list of probable cases from the two tertiary care hospitals testing for Influenza A (H1N1) pdm09 and reporting to IDSP for Lucknow district. We obtained data on laboratory-confirmed A(H1N1)pdm09 cases from IDSP. We also collected information about symptoms, treatment, and hospitalization.

**Results**

The confirmed A(H1N1) pdm09 cases in 2019 exceeded the outbreak threshold of the mean plus two standard deviations from 2016–2018 (Figure 1). There was an increase in the number of A(H1N1) pdm09-confirmed cases starting in week 5 of 2019 compared to the same week in previous years. Cases peaked during week nine at 104 cases and declined after that. We identified 3,196 probable, and 514 (16%) confirmed cases, including four deaths for a case-fatality rate of 8 per 1,000. The attack rate was 16 per 100,000 population, and the influenza-specific mortality rate was 0.1 per 100,000 population. The median age of persons confirmed to have A(H1N1) pdm09 was 36 years (range: 6 months–99 years). Children <5 years of age had the highest attack rate at 31 per 100,000 population, followed by persons >60 years of age at 29 per 100,000 population. Over half of (54%, 276/514) cases and 3 (75%) deaths occurred among females. 73% of the cases occurred in peri-urban areas. Among confirmed cases, the most common symptoms were fever (94%), cough (83%), and runny nose (60%), 86% received oseltamivir, and 31% were hospitalized.

**Conclusions**

During the first four months of 2019, a laboratory-confirmed influenza A(H1N1) pdm09 outbreak occurred primarily among young children and older persons in the peri-urban areas of Lucknow district. This outbreak's epidemiological characteristics were consistent with the literature for A(H1N1) pdm09 describing the young and elderly as at-risk populations[4]. Because the IDSP system only captured data from two public tertiary care hospitals, the extent of the outbreak in Lucknow district was likely under-estimated. We recommend that all persons with influenza-like symptoms during influenza season from November to April seek diagnostic testing and those with confirmed A(H1N1)pdm09 be treated with oseltamivir to prevent hospitalizations deaths. To prevent morbidity and mortality associated with seasonal influenza, India should consider adopting an influenza vaccine for at-risk groups, including young children and the elderly.

**References**

1. Pradesh M, Nadu T. The 2015 influenza A (H1N1) pdm09 outbreak in India. 2016;2015(June):821–3. Available from: https://www.ncbi.nlm.nih.gov/pmc/articles/ PMC5094123/#:~:text=The%20influenza %20A(H1N1)%20pdm09, Karnataka%20 and%20 Tamil%20Nadu2.

2. Welfare F. Pandemic Influenza A H1N1 Directorate General of Health Services Ministry of Health and Family Welfare Government of India. Available from: https://main.mohfw.gov.in/sites/default/files/2366426352.pdf

3. Ani. “H1N1 virus claimed 1,076 lives in 2019, over 26,000 infected.” Business Standards [Internet]. 2019 Jun 29; Available from: https://www.business-standard.com/ article/news-ani/h1n1-virus-claimed-1- 076-lives-in-2019-over-26- 000-infected-119062900250_1.html

4. Punpanich W, Chotpitayasunondh T. International Journal of Infectious Diseases A review on the clinical spectrum and natural history of human influenza. Int J Infect Dis [Internet]. 2012;16(10):e714–23. Available from: http://dx.doi.org/10.1016/j.ijid.2012.05.1025

**Fig. 1 (abstract A35).**
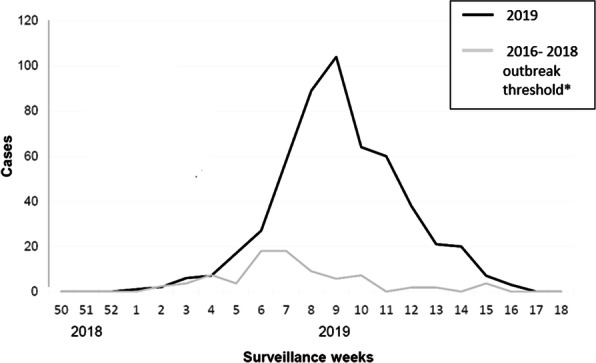
Confirmed cases of influenza A(H1N1)pdm09 by reporting week in Lucknow district, Uttar Pradesh, India, from December to May 2019 compared to the 2016–2018 outbreak threshold*. *Outbreak threshold = the mean number of cases for 2016–2018 plus two standard deviations

## A36 Assessment of the Measles-Rubella Surveillance System in Hyderabad, India, 2018

### Puttaraju Agrahara Kumbijetty^1^, Sushma Choudhary^2^, Manjunatha S Nagaraja^3^, Pankaj Bhatnagar^1^

#### ^1^National Public Health Surveillance Programme, World Health Organization Country Office, New Delhi, India; ^2^South Asia Field Epidemiology and Technology Network, New Delhi, India; ^3^Professor, Department of Community Medicine,, Mysore Medical College and Research Institute, Mysore, Karnataka, India

##### **Correspondence:** Puttaraju Agrahara Kumbijetty (srchyderabad@npsuindia.org)

**Background**

In 2015, India accounted for 36% of the 134,200 measles deaths reported globally, and 72% of rubella cases reported in the Southeast Asia Region (SEAR) [1,2]. In line with the measles and rubella elimination goal for the World Health Organization-SEAR, India has targeted measles and rubella elimination by 2023 [3]. The two essential elimination strategies are attaining >95% population immunity to measles and rubella in all states & districts and establishing & sustaining high-quality laboratory supported measles and rubella case-based surveillance in all districts [3].

Telangana State initiated state-wide case-based measles and rubella surveillance in January 2018. We assessed Hyderabad district's measles-rubella surveillance system to see whether the system can achieve and maintain the expected performance indicators, identify gaps, and support policy decisions for measles and rubella elimination in India. We assessed the measles-rubella system against selected attributes for strengths and weaknesses and provided evidence-based recommendations.

**Methods**

We used Centers for Disease Control and Prevention (CDC) guidelines for surveillance evaluation in this study [4]. The study was conducted in April 2018 in Hyderabad, Telangana, India, with a population of 6.73 million in 2011 [5]. The district is divided into 15 clusters, of which three were selected by random sampling. One urban primary health center, one government medical college hospital, and one private health care facility were chosen within each selected cluster using purposive sampling. We used a semi-structured questionnaire for interviewing the health staff to assess knowledge of the surveillance case definition, data collection, compilation, flow, analysis process, and the feedback mechanism. We drafted different sets of interview schedules based on the level of expertise needed by the staff. We reviewed surveillance documents at different levels of the system for the flow of data, analysis, and feedback provided by the district surveillance unit. We assessed system attributes such as simplicity, flexibility, data quality, acceptability, sensitivity, timeliness, representativeness, and stability as a part of our evaluation. We measured the system's sensitivity, as of week 24 in 2018, by comparing the ‘non-measles non-rubella discard rate’ (NMNR) against expected per 100,000 population.

**Results**

We interviewed 19 health staff, three at the district health (two district immunization officers and a statistical officer), five at the medical college, six at a primary health center, three at the sub-center, and three at the private health facilities. All 12 individuals interviewed about the case definition were aware of the same and the requirements and procedures for laboratory confirmation, indicating a simple system (Table 1). Twelve of 15 (80%) interviewees reported cases by completing case investigation forms, were aware of specimen collection procedures, and reported data to the higher level. As an indication of flexibility, the system has adapted to new case definitions and a switch from outbreak-based surveillance to case-based surveillance in January 2018. Newer laboratory confirmatory methods were used by 17 of 19 (89%) interviewed, and 12 of 13 (92%) knew about the change in threshold for flagging outbreaks.

We found that 27 of 34 (79%) weekly reports examined were complete, and 19 of 25 (76%) case investigation forms discussed were complete. Of the 1768 variables on the weekly reports, 565 (32%) were missing. The system is acceptable, as evidenced by the fact that 33 of 34 (97%) reporting units submitted weekly reports on time and did not find any unreported cases during our active case search in the hospitals we visited. For sensitivity, the NMNR was 0.8 as against the expected value of 2 per 100,000 per year. Seventy-six percent (1343/1768) of the weekly reports were submitted on time. The system has good representativeness. All 15 (100%) clusters in the district had a measles-rubella reporting system, and 76% of the reporting units were from private health facilities. Finally, the system is stable as all health facilities visited had identified nodal officers and required logistics.

**Conclusions**

The measles-rubella surveillance system evaluated in the present study in the district of Hyderabad operates as per the established guidelines of the Government of India. The flow of data and protocols for outbreak investigation and case-based surveillance are consistent with national policies. However, the study was able to cover only 3/15 clusters and only three facilities within each, and hence generalisability of these results is yet to be explored.

We recommend: 1) regular review of the program (e.g., analyzing surveillance indicators for reporting facilities and the district every month at the local and state level) to improve timeliness and data quality, 2) orientation and training of health personnel in government and the private reporting sites for improved reporting and response, and 3) encourage the primary health centers, front line health workers and the community to report all suspect measles and rubella cases to the surveillance system to improve sensitivity.

**Acknowledgments**

We thank Dr. Pauline Harvey, Team Leader, National Polio Surveillance Project, World Health Organization India, and U.S. Centers for Disease Control and Prevention (U.S. CDC); and Dr. Rajesh Yadav, Division of Global Health Protection, Center for Global Health, U.S. CDC, India, for their technical assistance and scientific review. We would also like to thank Niloufer hospital, Aditya Hospital, UPHCs, the district health office, Hyderabad, for supporting the study.

**References**

1. Orenstein WA, Cairns L, Hinman A, Nkowane B, Olivé JM, Reingold AL. Measles and Rubella Global Strategic Plan 2012-2020 midterm review report: Background and summary. Vaccine. 2018;36 Suppl 1:A35-A42. doi:10.1016/j.vaccine.2017.10.065

2. World Health Organization. Global measles and rubella update November 2018 https://www.who.int/immunization/monitoring_surveillance/burden/vpd/surveillance_type/active/Global_MR_Update_November_2018.pdf?ua=1.Accessed 16 June 2020.

3. World Health Organization, Regional Office for South-East Asia. (2015). Strategic plan for measles elimination and rubella and congenital rubella syndrome control in the South-East Asia Region, 2014-2020. WHO Regional Office for South-East Asia. https://apps.who.int/iris/handle/10665/205923

4. German RR, Lee LM, Horan JM, et al. Updated guidelines for evaluating public health surveillance systems: recommendations from the Guidelines Working Group. MMWR Recomm Rep. 2001;50(RR-13):1‐CE7.

5. *Census* Commissioner of India under the Ministry of Home Affairs, Government of India. *Census* of India 2011. https://www.censusindia.gov.in/2011Census/pes/Pesreport.pdf. Accessed 22 June 2020.

**Table 1 (abstract A36).** Evaluation of selected attributes of the measles-rubella surveillance system in Hyderabad, India, 2018

Attribute	Indicators	n/N	%
Simplicity	Awareness about the definition of suspected Measles-Rubella case	12/12	100
	Ease of case reporting, case investigation form filling, and data transfer to a higher level	12/15	80
	Laboratory confirmation requirements and process	12/12	100
Flexibility	Understanding and implementation of case-based surveillance	17/19	89
	New reporting and laboratory system	17/19	89
	Change in the threshold for flagging outbreaks	12/13	92
Data Quality	Completeness of case investigation forms	19/25	76
	Completeness of weekly reports	27/34	79
	Variables missing from weekly reports	565/ 1768	32
Acceptability	Number of reporting units submitting a weekly report	33/34	97
	Unreported cases detected in active case search	-	
Sensitivity	Compared ‘non-measles non-rubella discard rate’ against expected – 2 per 100,000 population per year	For week 24, 2018 = 0.8	
Timeliness	Timely submission of weekly report	1343/1768	76
Representativeness	Geographic distribution of reporting sites	15/15	100
	Private facilities	108/141	76
Stability	All health facilities had identified human resources for measles-rubella surveillance.		
	Line lists of all reported cases and weekly reports available at assessed sites.		
	No system breakdowns were identified, such as out of stock of reporting forms and specimen collection kits.		

## A37 Assessment of Kala-azar Surveillance System in Khagaria District, Bihar, August 2018

### Ashish Nawal Tigga^1^, Amol Patil^2^, Mohammad Ahmad^1^, Pankaj Bhatnagar^1^

#### ^1^National Public Health Surveillance Project, World Health Organization, New Delhi, India; ^2^South Asia Field Epidemiology and Technology Network, New Delhi, India

##### **Correspondence:** Ashish Nawal Tigga (srckhagaria@npsuindia.org)

**Background**

The National Vector Borne Disease Control Program (NVBDCP) was launched in 2003-04 as an umbrella program for preventing and controlling vector-borne diseases, which includes malaria, filariasis, and kala-azar, dengue, and chikungunya [1]. Kala-azar or visceral leishmaniasis was targeted for elimination by the year 2020. However, 54 districts in India are endemic for the disease, of which 33 belong to Bihar state. Eighty-six percentage of 485 affected blocks (administrative subunits of the district) of Bihar have achieved elimination [2]. Studying the surveillance for kala-azar in Bihar will help to understand the progress and the difficulties faced in the path to the elimination of the disease.

**Methods**

We assessed the kala-azar surveillance system in the Khagaria district of Bihar for strengths and weaknesses and proposed recommendations for improvement. Two blocks of Khagaria, Alauli, and Parbatta, with the highest and lowest number of kala-azar cases, respectively, were included in the assessment. We used the U.S. Centers for Disease Control and Prevention (CDC) guidelines for evaluating specific attributes of the public health surveillance system [3]. With a focus on 2015-2018, we interviewed the District Vector Borne Disease Control Officer, District Vector Borne Disease Consultant, four Medical Officers (MO), two kala-azar Technical Supervisors (KTS), eight private practitioners (PP), four Auxiliary Nurse Midwives (ANM) and eleven Accredited Social Health Activists (ASHA) regarding the simplicity of the case definition and the flexibility in reporting. We reviewed diagnostic methods for flexibility, block line lists for both data quality and flexibility, and district and block monthly reports for completeness and timeliness. We assessed the acceptability component by reviewing the training and engagement of the MOs, KTSs, ANMs, and ASHAs at the task force and stakeholder meetings. The proportion of cases reported by the health facilities and the proportion of private health facilities reporting cases were used to evaluate the representativeness. We evaluated the programme's stability by calculating the proportion of filled human resource vacancies in the program. For usefulness, we analyzed monthly disease trends at the block level.

**Results**

Overall, 34.5% (10 of 29) of the staff interviewed were aware of the case definition (Table 1). Different methods for reporting the cases were indicated by the ASHAs, demonstrating flexibility in the system. As additional evidence of flexibility, fields were modified or added to the line list to adapt to evolving needs, such as a diagnostic method change. We found the monthly district report was complete for 2015 – 2017; however, block line lists had 431 blank fields out of 1390 (31%).We identified that, in 2015, only 58% (14/24) of the block reports were submitted, which increased to 79% in 2016 & 2017. Only 66% (2/3) of the district reports were presented on time from 2015 to 2017.

Medical Officers & KTSs were trained at the national & state level, respectively, and considered acceptable. However, from January to July 2018, the District Task Force meeting frequency was low (1/7,14%). The meeting frequency was better at the block level (10/14, 71%). In 2018, there was documented discussion of kala-azar one time each at ANM (1/26, 3.8%) and ASHA (1/27, 3.7%) meetings at the block level. We could not assess representativeness because there were insufficient data to determine which entities reported cases and to determine denominators for those calculations. There were no human resource vacancies, as all sanctioned posts for kala-azar surveillance had been filled. The Khagaria district showed a progressive decline in reported cases: from 131 in 2015 to 63 in 2017. All blocks within Khagaria achieved elimination status in 2016.

**Conclusions**

The kala-azar surveillance system in Khagaria is flexible, stable, acceptable, and useful. It is simple for MOs and KTSs, but not for ANMs and ASHAs. However, there is a need to improve data quality (particularly at the block level) to make the system simpler for ANMs and ASHAs. Discussions at district and block level meetings of the stakeholders and the health staff should improve. It would be beneficial to have a listing of private health facilities to further act upon and ensure their surveillance involvement.

**References**

1. Government of India. National Vector Borne Disease Control Programme. Operational guidelines on Kala-azar (Visceral Leishmaniasis) Elimination in India-2015. Delhi (IN). 2015. https://nvbdcp.gov.in/Doc/opertional-guideline-KA-2015.pdf. Accessed 1 Oct 2020.

2. Directorate of National Vector Borne Disease Control Programme. Ministry of Health and Family Welfare. Government of India. Accelerated plan for Kala-azar Elimination 2017. Delhi (IN). 2017. 94p. https://www.who.int/leishmaniasis/resources/Accelerated_plan_for_Kala-azar_Elimination_2017/en/ Accessed 1 Oct 2020.

3. Centers for Disease Control and Prevention. Updated guidelines for evaluating public health surveillance systems: recommendations from the guidelines working group. https://www.cdc.gov/mmwr/preview/mmwrhtml/rr5013a1.htm Accessed 1 Oct 2020.

**Table 1 (abstract A37).** Evaluation of attributes of the kala-azar surveillance system, Khagaria, Bihar

Attribute	Indicator	n/N	%
Simplicity	MOs aware of case definition	4/4	100
	KTSs aware of case definition	2/2	100
	PPs aware of case definition	4/8	50
	ASHAs aware of case definition	0/11	0
	ANMs aware of case definition	0/4	0
Flexibility	ASHA using more than one method for reporting	More than one method, such as short message service (SMS), call or personal accompaniment, used	
	Change of diagnostic test	Changed to rapid detection of anti-recombinant 39-amino acid repeat antigen (rK-39) from rK-16	
	Additional fields in line-list (2017)	▪ Co-infection with HIV/TB ▪ Place of treatment ▪ Unique identification number ▪ Referral details – ASHA/ Doctor/other primary health centers	
	Modifications in line-list (2017)	▪ Outcome at 1, 6, and 12 months ▪ The extra field ‘lost’ added to the outcome	
Acceptability	MO Training	2017 – national-level training	
	KTS Training	2018 – state-level training	
	District Task Force Meeting frequency	2018 – 1/7	14
	District Partners’ Meeting Frequency	2018 – 1/7	14
	Block Task Force Meeting frequency	2018 – 12/16	75
	ANM Meeting discussion on kala-azar	2018 – 1/26	3.8
	ASHA Meeting discussion on kala-azar	2018 – 1/27	3.7
Data Quality	Proportion of blank fields in line-list	431/1390	31
	Completeness of Monthly district report	2017 – 3/3	100
Timeliness	Timeliness of district reports	2015 – 2/3	66
		2016 – 2/3	66
		2017 – 2/3	66
	Timeliness of block reports	2015 – 14/24	58
		2016 – 19/24	79
		2017 – 19/24	79
Stability	District Vector Borne Disease Control Officer posting vs sanctioned post	1/1	100
	District Vector Borne Disease Consultant posted vs sanctioned post	1/1	100
	Posted vs sanctioned posts - KTS	6/6	100

## A38 Assessment of the Integrated Disease Surveillance Programme Surveillance System for Measles, Ghaziabad District, Uttar Pradesh, 2018

### Rajesh Kumar^1^**,** Sushma Choudhary^2^, Madhup Bajpai^1^, Pankaj Bhatnagar^1^

#### ^1^National Public Health Surveillance Project, World Health Organization, Delhi, India; ^2^South Asia Field Epidemiology and Technology Network, Delhi, India

##### **Correspondence:** Rajesh Kumar (srcghaziabad@npsuindia.org)

**Background**

In 2016, an estimated 20.4 million people were affected by measles globally [1]**.** In 2017, India reported 45,773 measles cases and 678 outbreaks and accounted for 36% of the estimated 49,000 measles deaths reported globally [1]. Measles is targeted for elimination by 2023, and measles surveillance is one of the strategies required for elimination as it provides information for action, including identifying the strengths and weaknesses of the system. We described and assessed the Integrated Disease Surveillance Programme (IDSP) surveillance system for measles in Ghaziabad District, Uttar Pradesh, to provide evidence-based recommendations for strengthening the system.

**Methods**

We used the U.S. Centers for Disease Control and Prevention guidelines for the assessment of surveillance systems [2] for the period January-February 2018. We selected a district surveillance unit and five reporting units in Ghaziabad, one of the 75 districts of Uttar Pradesh State. The reporting units were selected randomly in order to have equal representation of rural and urban areas. Two sub-centers were selected based on the distance to the administrative primary health center one farthest from the administrative primary health center and one nearer. We interviewed key informants, including the District Immunization Officer, the District Surveillance Officer, the Medical Officer in Charge of the primary health center, two Auxiliary Nurse Midwives, and two private practitioners. We reviewed suspect (S), presumptive (P), and laboratory (L) forms and the IDSP web portal [3]. We assessed simplicity, acceptability, representativeness, flexibility, stability, data quality, completeness and timeliness attributes.

**Results**

The case definition used by the measles surveillance system defines a suspect measles case as any person with fever and rash and a probable case as any person with fever and maculopapular rash with cough, coryza, or conjunctivitis. A confirmed case has a positive serological test for measles, detection of measles virus-specific nucleic acid from a clinical specimen using polymerase chain reaction, isolation of measles virus from a clinical specimen, or direct epidemiological linkage to a case confirmed by one of the described methods. Data flows from the sub-center on the S form completed by a health worker to the primary health center every Monday. The S form captures measles as a syndrome characterized by “fever with rash.” The Medical Officers complete the P form, capturing probable measles based on clinical findings from the out-patient and emergency records. The P form is submitted to the district surveillance unit on Mondays from the reporting units, including the primary health center, the district hospital and private hospitals. The laboratories submit data on L forms completed by laboratory technical staff, based on the number of samples tested and the number of samples found positive. The P and L forms are uploaded on the IDSP web portal and are available at the state surveillance unit on Tuesday and the center surveillance unit on Wednesday [3]**.**

All seven staff interviewed were aware of the case definition and reported that compiling the measles data and reporting to the next level was simple (Table 1). The reporting units submitted 29 of 32 expected (91%) P and L forms; however, no S forms were submitted, indicating mixed acceptability. Besides, no feedback was provided by the district surveillance unit to the reporting units. At the district surveillance unit, 62 of 68 (91%) government facilities, 22 of 314 (7%) private facilities, and 22 of 228 (9.6%) private laboratories were reported, indicating that private facilities and private laboratories are underrepresented in the surveillance system. At the district surveillance unit, reports from 15 of 16 (93%) government, three of four (75%) private facilities, and all eight (100%) private labs were received on time; none were received from subcenters. All 24 of the P forms and all 24 of the L forms that we reviewed were complete; no S forms were available for review. Twenty-two of 86 (26%) reporting units submitted reports by email, and four of five (80%) staff reported being willing to report in multiple formats, indicating flexibility in the system. Printed S forms were not available at sub-centers. However, the annual budget of 12.8 lacs was fully available to support the system, all IDSP positions at the district surveillance unit were filled, and computers and internet connections were available for reporting, demonstrating a stable system.

**Conclusion**

The IDSP measles surveillance system in Ghaziabad District, Uttar Pradesh, was simple, acceptable, stable, timely, and the data quality was found to be good at all reporting units except at the sub-centers. There is a need for improvement at the sub-centers, specifically for data quality and timeliness. We recommend that S forms be made available to the sub-centers to ensure regular report submission to the Medical Officer.

**References**

1. World Health Organization. Measles and Rubella Surveillance Data.[https://www.who.int/immunization/monitoring_surveillance/burden/vpd/surveillance type/active/measles_monthlydata/en/] Accessed 2 June 2020.

2. German RR, Lee LM, Horan JM, et al. Updated guidelines for evaluating public health surveillance systems: recommendations from the Guidelines Working Group. MMWR Recomm Rep. 2001;50(RR-13):1‐CE7 [https://pubmed.ncbi.nlm.nih.gov/18634202/]. Accessed 2 June 2020.

3. National Centre for Disease Control. Integrated Disease Surveillance Project. Training manual for medical officers for hospital based disease surveillance. [https://idsp.nic.in/WriteReadData/OldSite/usermanaul/manual_for_MO.pdf]. Accessed 2 June 2020.

**Table 1 (abstract A38).** Attributes of the IDSP Surveillance System for Measles, Ghaziabad District, Uttar Pradesh, 2018

Attribute	Indicator	n/N	%
**Simplicity**	Staff aware of case definition	7/7	100
	Staff found ease of reporting	7/7	100
	Staff found ease of compilation of information	5/5	100
	Ease of data analysis	1/1	100
**Flexibility**	Proportion of reporting units sending weekly report by email	22/86	26
**Acceptability**	Staff willing to report in formats	4/5	80
	P and L forms submitted against expected by reporting units	29/32	91
	S forms submitted against expected Feedback provided by the district surveillance unit to the reporting units	0/16 0/8	0 0
**Data Quality**	Completeness of P form	24/24	100
	Completeness of L form	24/24	100
**Timeliness**	Timeliness of report from sub-centre	0/16	0
	Timeliness of report from government reporting units	15/16	93
	Timeliness of report from private facilities	3/4	75
	Timeliness of report by district	8/8	100
**Stability**	Vacant positions	0	0
	Availability of computer and internet connection for reporting	Yes	
	S forms	Not available	
**Representativeness**	Proportion of government facilities included in reporting network against expected	62/68	91
	Proportion of private facilities in reporting number against expected	22/314	7
	Proportion of registered private laboratories included in reporting network	22/228	9

## A39 Analysis of measles outbreaks in Aligarh District, Uttar Pradesh, India, 2014–2018

### Vikas Kumar Gupta^1^, Pankaj B Shah^2^, Rajesh Yadav^3^, Pankaj Bhatnagar^1^, Pauline Harvey^1,4^

#### ^1^National Public Health Surveillance Project, World Health Organization Country Office, New Delhi, India; ^2^Shri Ramchandra Medical College, Porur, Chennai, Tamil Nadu, India; ^3^Division of Global Health Protection, U.S. Centers for Disease Control and Prevention, New Delhi, India; ^4^Global Immunization Division, U.S. Centers for Disease Control and Prevention, Atlanta, GA, USA

##### **Correspondence:** Vikas Kumar Gupta (srcaligarh@npsuindia.org)

**Background**

Despite the availability of a safe, effective, and inexpensive measles vaccine, measles is a leading cause of childhood morbidity and mortality globally. In 2019, India and the other member states of the World Health Organization (WHO) South-East Asia Region committed to eliminate measles and rubella by 2023 [1]. India reported 1,471 suspected measles outbreaks and 20,925 measles cases in 2018 [2]. Achieving measles elimination by 2023 in India requires an understanding of the epidemiology of measles outbreaks and ensuring adequate outbreak preparedness and response [1]. Measles surveillance was launched in a phased manner across India in 2005. Uttar Pradesh State started outbreak-based measles surveillance in October 2013 [3]. National guidance recommends serological confirmation of all measles outbreaks [4,5]. Since outbreak-based measles surveillance was started in Uttar Pradesh, there has been no systematic descriptive analysis of outbreak trends. We aimed to describe the trends of measles cases reported through WHO’s National Public Health Surveillance Project (NPSP), Aligarh District, Uttar Pradesh, for the period 2014–2018.

**Methods**

We conducted a descriptive analysis of measles outbreak-based NPSP surveillance data submitted by reporting units in the Aligarh district for 2014–2018. Suspected measles cases were reported from government hospitals and selected private hospitals and clinics by district immunization officers to the state government through India’s Integrated Disease Surveillance Programme. Suspected outbreaks were flagged whenever five or more suspected measles cases or one death due to suspected measles were reported in a 4–week period from any community-block (i.e., sub-district level). An epidemic response team conducted a preliminary case search to assess for clustering of cases in the area. If clustering of cases was found, the response team conducted house-to-house case searches and created a line-list of all suspected measles cases or deaths. Using structured questionnaires and standard case definitions, the team collected information including age, religion, date of rash onset, vaccination status, and death. The team collected blood specimens from five suspected cases from the outbreak area to confirm measles based on detection of anti-measles virus-specific IgM antibodies by enzyme-linked immunosorbent assay (ELISA) [5]. A measles outbreak was confirmed when two or more measles cases were positive for measles IgM antibodies [5].

For the analysis, we included all suspected measles cases residing in Aligarh during 2014–2018 that were part of an outbreak and had legible case investigation forms. Case forms that were unreadable were excluded from the analysis. We analyzed the measles outbreaks for seasonality, serological confirmation, size and duration, vaccination status, median age, time lag between reporting and investigation, and distribution of cases by religion.

**Results**

During 2014–2018, 62 suspected measles outbreaks were identified in Aligarh District, and 50 (81%) were identified between November and May. Serological testing was conducted in 45 (73%) outbreaks, and of these, 36 (80%) were serologically confirmed as measles (Table 1). No measles outbreaks were confirmed in 2016. The median number of cases in confirmed measles outbreaks was 17 (range: 6–73). The median duration of confirmed outbreaks was 42 days (range: 8–93 days). The median lag from the rash onset in the first case to notification was 23 days (range: 0–90 days), and the median lag from notification to outbreak investigation was 13 (3–28 days).

Of the 36 confirmed measles outbreaks, 200 (23%) cases had serological specimens collected, and 115 (58%) cases were serologically confirmed as measles. Overall, there were 887 measles cases and six measles-related deaths during 2014-18. Five (83%) deaths occurred in 2017. The measles case-fatality rate was 0.7% (6/887). The median age was four years (range: 0–35 years), 407 (46%) of cases were aged 1–4 years, and 316 (36%) were aged 5–9 years. Of the cases, 457 (52%) were males, and 382 (43%) were religious minorities. Among the cases, 110 (12%) had received measles-containing vaccine first dose (MCV1), 80 (9%) had received MCV2, 591 (67%) did not receive the vaccine, and the vaccination status of 106 (12%) was unknown.

We only analyzed suspected outbreaks with five or more cases and cases with readable case investigation forms. Therefore, characteristics of outbreak cases might not be generalizable to all measles cases in the Aligarh district, and the number of cases per outbreak might be underestimated.

**Conclusions**

There were no clear trends or patterns observed in the number of suspected measles outbreaks identified, investigated, or confirmed in the Aligarh district during 2014-18. There were delays in both notification and investigation of suspected measles outbreaks but no trends over time.

We recommended targeted measles vaccine delivery to under-served and religious-minority communities. We also recommended expanding the reporting network to include faith healers, temples, and mosques to notify all measles cases. Training the frontline health workers to detect the cases early, timely notification, and response to suspected measles outbreaks.

**References**

1. World Health Organization (WHO). Strategic plan for measles and rubella elimination in WHO South-East Asia Region:2020–2024. New Delhi: WHO, Regional Office for South-East Asia, 2019. [https://apps.who.int/iris/handle/10665/330356]

2. World Health Organization (WHO) and Ministry of Health and Family Welfare (MOHFW), Government of India (GOI). Measles and rubella surveillance bulletin, December 2019. New Delhi: WHO and MOHFW, GOI, 2019.

3. Ministry of Health and Family Welfare (MOHFW), Government of India. National operational guidelines for measles and rubella Campaigns. New Delhi: MOHFW, GOI, 2016.

4. World Health Organization (WHO). Surveillance guide for vaccine preventable diseases in WHO South East Asia Region – 2017. New Delhi, India: WHO, Regional Office for South-East Asia, 2017. [https://apps.who.int/iris/handle/10665/277459]

5. Ministry of Health and Family Welfare (MOHFW), Government of India (GOI). Measles surveillance and outbreak investigation: field guide. New Delhi: MOHFW, GOI, September 2006.

**Table 1 (abstract A39).** Characteristics of measles outbreaks and outbreak-related measles cases in Aligarh District, Uttar Pradesh, India, 2014–2018

Outbreaks	2014	2015	2016	2017	2018	Total
	n (%)					n (%)
Identified	13	16	2	19	12	62
Investigated with serological testing	9 (69)	7 (44)	2 (100)	17 (89)	10 (83)	45 (73)
Confirmed by serologic testing	9 (69)	7 (44)	0 (—)	16 (84)	4 (33)	36 (58)
Cases, median (range)	14 (8–45)	35 (23–73)	0 (—)	17 (9–31)	15 (6–22)	17 (6–73)
	**Days (range)**					
Duration, median	29 (9–91)	60 (28–93)	60 (29–91)	42 (9–84)	34 (8–71)	42 (8–93)
Median lag from rash onset in first case to notification	23 (1–90)	46 (18–82)	53 (23–84)	53 (0–91)	22 (0–74)	23 (0–90)
Median lag from notification to investigation	13 (3–28)	14 (10–22)	8 (7–10)	8 (3–21)	12 (3–26)	13 (3–28)
**Confirmed cases**	**n (%)**					**n (%)**
Number	158	295	—	279	155	887
Age (yrs), median (range)	4 (0–35)	3 (0–25)	—	5 (0–30)	4 (0–13)	4 (0–35)
<1 year	27 (17)	30 (10)	—	31 (11)	15 (10)	103 (12)
1–4 years	76 (48)	142 (48)	—	137 (49)	52 (34)	407 (46)
5–9 years	40 (25)	107 (36)	—	92 (33)	77 (50)	316 (36)
10-14 years	12 (8)	12 (4)	—	16 (6)	11 (7)	51 (6)
≥15 years	3 (2)	4 (1)	—	3 (1)	0 (—)	10 (1)
Sex, male	70 (44)	142 (48)	—	158 (57)	87 (56)	457 (52)
Religious minority	79 (50)	166 (56)	—	116 (42)	21 (14)	382 (43)
Vaccination status						
MCV 1	26 (16)	21 (7)	—	49 (18)	14 (9)	110 (12)
MCV 2	11 (7)	5 (2)	—	44 (16)	20 (13)	80 (9)
Unvaccinated	103 (65)	246 (83)	—	146 (52)	96 (62)	591 (67)
Unknown	18 (11)	23 (8)	—	40 (14)	25 (16)	106 (12)

## A40 Unaware hypertension among persons with hypertension in the adult population of Kangra District, Himachal Pradesh, 2019

### Gurmeet Katoch^1^, Parasuraman Ganeshkumar^2^, Prabhdeep Kaur^2^ Rajesh Guleri^1^ Boopathi Kanguswamy^2^

#### ^1^Directorate of Health Services, Himachal Pradesh, India; ^2^ICMR-National Institute of Epidemiology, Chennai, Tamil Nadu, India

##### **Correspondence:** Parasuraman Ganeshkumar (ganeshkumardr@gmail.com)

**Background**

Low- and middle-income countries contributed 80% of NCD deaths worldwide. Cardiovascular diseases (CVDs) attributed to one in every three NCD death. In the past decade, CVD deaths are decreasing in high-income countries but increasing in low-middle income countries [1]. Hypertension is the single largest risk factor for CVD deaths. It caused 10.3 million deaths and 208 million disability-adjusted life years (DALYs) in 2013 [2]. The global prevalence of raised blood pressure in adults is almost equal in both genders and around 24.1% in men and 20.1% in women [2]. Based on a systematic review by Anchala R et al. in 2014 the prevalence of Hypertension in India among adults 18 years and above was 30% and the prevalence in urban and rural was 34% and 28% respectively [3]. It was estimated that one-tenth of rural Indians and one-fifth of urban Indians with high blood pressure had it under control and awareness of their hypertension status among adults was found to be 25.1% [3]. As per the District level Household Survey, round 4 (DLHS-4) conducted during 2012-13 the prevalence of hypertension among the adult population in the Kangra district of Himachal Pradesh was 38% [4]. A study by Bhardwaj R et al. in Himachal Pradesh reported that 78% of persons with hypertension were unaware of their hypertension status, and 4.3% were under control [5]. Population-level awareness of hypertension status of adults was not well understood. We conducted a community-based study among adults in Kangra district of Himachal Pradesh to estimate unaware hypertension and control status of hypertension.

**Methods**

We conducted a cross-sectional study between December 2018 and May 2019 in Kangra district of Himachal Pradesh state in India among person aged 18 years and above. We adopted a multi-stage cluster sampling technique to select individuals with hypertension from 445 health sub-centres in Kangra district with health sub-centre as a cluster unit. Health sub-centres were selected by probability proportional to the size of the adult population, and in each selected cluster, one Accredited Social Health Activist (ASHA) area of 1000 population was selected randomly. From the list of households maintained in each selected area, one household was selected randomly from which the required number of houses were included consecutively. One adult household was selected randomly from each house after enumerating all the adults 18 years and above using an android application. The selected individual was included in the interview. Assuming 50% of the hypertensives were unaware about their hypertensive status with the absolute precision of 10% with 95% confidence interval (CI) and nonresponse rate of 20%, the estimated required number of hypertensives was 120. To obtain the 120 hypertensives, we estimated to include 410 adults based on the prevalence of hypertension as 35%. Thus, we selected 25 health sub-centres and 17 adults per cluster. Unaware of hypertension status is defined as any individual who was hypertensive at the survey time and not being aware of being hypertensive. We measured blood pressure to all individuals included in the study using a clinically validated electronic blood pressure apparatus at their household. We measured two blood pressure readings for all individuals. We adopted a modified WHO STEPS Questionnaire to collect data on sociodemographic characteristics like age, gender, occupation and education and NCD risk factors like tobacco, alcohol, physical activity, unhealthy diet and added salt intake. Data were analysed using Epi Info 7 and SPSS 20 software. We estimated the prevalence of hypertension, unaware hypertension and control as proportions with 95% Confidence interval (CI). We obtained clearance from the Institutional Human Ethics Committee (IHEC) of ICMR – National Institute of Epidemiology, Chennai, before the study's initiation. We obtained written informed consent from the participants before the data collection and informed the participants about the study's objective.

**Results**

We included 427 adults in our study, with 51% in 18-45 years, and 69% were females. Of the 427 adults, we found hypertension in 133 adults. Thus, the prevalence of hypertension in 18 years and above was 31% (95% CI 26.8-35.5). Among the 133 identified hypertensives, the proportion of unaware hypertension was 54% (95% CI 49.4-58.9). Majority of the individuals unaware of their hypertension status was in the age group of 46-69 years (52.%), 65.35 were females and 63.9% with an education level of high school and above (Table 1). Of the 61 who were aware of their hypertensive status, most of them were in the age group of 46-69 years (57.4percent), 47% were females, and 47.5% were with the high school's education level above. Among the 61 who were aware of their hypertensive status, 50 individuals were under treatment, Of the 50 individuals under treatment 35 (70%) were not under control. On distributing the behavioural risk factor between aware and unaware hypertensive, added salt intake (38.9%), tobacco usage (20.8%) and alcohol intake (19.4%) were high among the individuals who were unaware hypertension status.

**Conclusion**

We observed that the prevalence of Hypertension in Kangra district of Himachal Pradesh was 31.1%. The proportion of unaware and uncontrolled hypertension was 54.1% and 70% respectively. Added salt intake and tobacco consumption were the major behavioural risk factors among the hypertensives. Females were overrepresented in our study. We look this as our limitation in our study. We suggest intensifying population-based screening of hypertension to detect unaware hypertension and initiate the treatment at the earliest. Also educating the hypertensives on importance of regular treatment and tracking them would improve the control rates. We recommend an adequate supply of drugs and consistent practice of standard treatment protocol across the health facilities to achieve these. We also suggest conducting analytical studies to determine the reasons for unaware and uncontrolled Hypertension in Kangra district.

**References**

1. World Health Organization: Global status report on noncommunicable diseases 2014 [Internet]. World Health Organization; [cited 2020 Aug 31]. Available from: http://apps.who.int/iris/bitstream/10665/148114/1/9789241564854_eng.pdf?ua=1

2. World Health Organization. A global brief on hypertension: silent killer, global public health crisis: World Health Day 2013. World Health Organization; 2013. Available from: https://apps.who.int/iris/rest/bitstreams/195800/retrieve

3. Anchala R, Kannuri NK, Pant H, Khan H, Franco OH, Di Angelantonio E, Prabhakaran D. Hypertension in India: a systematic review and meta-analysis of prevalence, awareness, and control of hypertension. J Hypertension. 2014;32 (6):1170-7.

4. District Fact Sheet KANGRA (2012-13) [Internet]. International institute for population sciences, Mumbai; [cited 2020 Aug 10]. (District Level Household and Facility survey -4). Available from: https://nrhm-mis.nic.in/DLHS4/State%20and%20District%20Factsheets/Himachal%20Pradesh/District%20Factsheets/Kangra.pdf

5. Bhardwaj R, Kandori A, Marwah R, Vaidya P, Singh B, Dhiman P, Sharma A. Prevalence, awareness and control of hypertension in rural communities of Himachal Pradesh. J Assoc Physicians India. 2010 Jul 1;58:423

**Table 1 (abstract A40).** Distribution of sociodemographic factors of the unaware population of Hypertension in Kangra district, Himachal Pradesh, 2019 (N= 72)

Factors		n (%)
Age group	18-45 years	23 (31.9)
	46-69 years	38 (52.8)
	70 years & above	11 (15.3)
Gender	Male	25 (34.7)
	Female	47 (65.3)
Occupation	Retired/Unemployed	10 (13.9)
	Homemaker	46 (63.9)
	Skilled/Unskilled	16 (22.2)
Education	No formal schooling	17 (23.6)
	Primary school	9 (12.5)
	High school & above	46 (63.9)

## A41 Data Improvement Plan for Vaccine-Preventable Disease Surveillance, Chhattisgarh, India, 2019

### Bala Ganesakumar^1^, Manishkumar Gawande^1^, Rahul Mohan Shimpi^1^, Mohankumar Raju^2^

#### ^1^National Public Health Surveillance Project, World Health Organization, Delhi, India; ^2^ICMR-National Institute of Epidemiology, Chennai, Tamil Nadu, India

##### **Correspondence:** Bala Ganesakumar (smotirunelveli@npsuindia.org)

**Background**

Vaccine-Preventable Disease (VPD) Surveillance in India is done by the Integrated Disease Surveillance Programme (IDSP) and World Health Organization - National Public health Surveillance Project (NPSP) Surveillance network. Both the system of surveillance is collecting Syndromic surveillance data for suspected measles and Acute Flaccid Paralysis (AFP). The case definition of a Suspected Measles Case is fever and Maculopapular rash of any age with any one of the following symptoms Cs (Cough, coryza, and conjunctivitis). The case definition of Acute Flaccid Paralysis Surveillance is a sudden onset of weakness/floppiness in children aged below 15 years.

The district surveillance officer (DSO) is the nodal officer for IDSP in the district. Data is collected from the field using “S” forms for the suspected cases and from the hospitals using “P” forms for presumptive cases. These data are collated at the block level and sent to the DSO on Monday, where the data from all the blocks of the district are compiled and sent to the State surveillance officer on Tuesday. The District Immunization Officer (DIO) is the nodal person for WHO-NPSP. Data from the hospitals are collected using H002 format, compiled at the block level, and sent to DIO on Mondays. Subsequently, DIO sends the consolidated report to the State Immunization officer (SIO) on Tuesdays. Both the IDSP and WHO-NPSP surveillance systems are collecting similar information on suspected measles and AFP. We aimed to compare the data sets of both systems of surveillance, identify the reasons for variations in the surveillance data, and recommend a data synchronization plan for Chhattisgarh, India

**Methods**

We abstracted the VPD and NPSP surveillance data using a data abstraction form of Chhattisgarh for the period weeks 1 to 35, 2019. We analysed the data and calculated proportions of differences in the VPD reporting between IDSP and WHO-NPSP. We conducted a cross-sectional survey among the stakeholders of the surveillance system. We selected three blocks in the seven districts of Chhattisgarh by simple random sampling. We collected information from 63 frontline workers, 42 Medical officers, 21 Block Medical officers, and seven District level authorities using a semi-structured questionnaire. We calculated the proportions of factors influencing the variation in the number of cases reported through both the system of surveillance. We also conducted focus group discussions among the DIOs and collected information. We calculated the proportion of strategies for the data improvement plan.

**Results**

The reported number of suspected measles cases up to week 35 of 2019 in Chhattisgarh was 51 under NPSP compared to 310 through IDSP. Similarly, AFP was reported as 245 cases through NPSP 498 through the IDSP System of surveillance. For both AFP and suspected measles, the number of cases reported through IDSP is more than WHO – NPSP H002 Surveillance. About 83% of field-level workers did not become aware of the dual systems of surveillance, and 66% of Medical officers stated that the suspected case definition varied in both systems. Sixty-three percent of medical officers stated that there is variation among the reporting network of both systems, and 92% of Medical officers opined that they did not have a regular feedback mechanism in the IDSP system of surveillance. The major factors which influenced the variations were lack of data entry operators at the block level, lack of synchronization of data sets block and district level, differences in the number of reporting network of both systems, non-availability of sample kits, no regular feedback and follow up in IDSP system of surveillance. Likewise, regarding the factors which synchronize and improve the Vaccine-Preventable disease surveillance data, all the Medical officers stated that there should be only one nodal person for both systems of surveillance, 94% Medical officers opined that Active case search by Block nodal officers by regularly verifying the OPD records and registers of other medical officers and 84% stated that data synchronization at district level every week during the weekly review meetings would improve the vaccine-preventable surveillance data of Chhattisgarh.

**Conclusions**

We found wide variations between NPSP and IDSP in the number of suspected measles and AFP cases reported in 2019. Factors influencing the difference in the data include lack of data integration, knowledge gap and non-uniform reporting structure, availability of sample kits, unavailability of the analytical person at the block level, and lack of feedback mechanism of the reported cases in the IDSP system of surveillance. Regular Training of Field workers and medical officers regarding basics of surveillance every year, epidemiological training to Block Medical Officers regarding descriptive analysis, Orientations about case definitions & types of samples, availability of sample kits, weekly review meetings to synchronize the data at the district level, Active case search by Block nodal officers by regularly verifying the OPD records and registers of other medical officers, mobile app-based data entry and integration of both systems of surveillance would help to synchronize and improve the data sets.

**Table 1 (abstract A41).** Factors that influence variations in both systems of VPD Surveillance and factors which could synchronize and improve VPD Surveillance data in Chhattisgarh, 2019

Categories	Indicators	(%)
Factors that influence the variations in both data (n=133)	No Verification of both data sets	92%
	Un availability of Sample kits in PHC	60%
	Variations in the number of reporting sites	63%
	Attended any Training about VPD surveillance by IDSP in the last year	21%
	No Epidemiological (Time Place Person - analysis) training to BMOs	100%
	No any Analytical person at the Block level	100%
	Don’t receive feedback or analytical report from districts regularly	92%
Factors that could synchronize and improve VPD Surveillance data (n=70)	One nodal person for both WHO and IDSP Surveillance	100%
	Weekly data synchronization at the district level	83%
	Active case search by BMOs in other MOs registers	94%
	Mobile app-based data entry	94%
	Integrating into one system	92%

## A42 Community-based intervention for blood pressure monitoring through Anganwadi centers in Viralimalai block, Pudukkottai District, Tamil Nadu, India, 2016-17

### Subash Gandhi VC^1^, Parasuraman Ganeshkumar^2^, Kamaraj Pattabi^2^

#### ^1^Department of Public Health and Preventive Medicine, Govt. of Tamil Nadu; ^2^ICMR National Institute of Epidemiology, Chennai, India

##### **Correspondence:** Parasuraman Ganeshkumar (ganeshkumardr@gmail.com)

**Background**

Complicating 10-15% of India's pregnancies, Pregnancy Induced Hypertension (PIH) is the most common medical problem encountered during pregnancy [1,2]. Early detection of PIH can prevent both maternal and fetal mortality and morbidity [3]. Globally, maternal deaths due to PIH was reported to be nearly 14% [4]. A representative sample from Tamil Nadu, a south Indian state, shows a PIH prevalence of 6.3% and maternal death due to the same as 29%. Pudukkottai district reported an 18% prevalence of women with full antenatal check-up completed, much lower than the state average for Tamil Nadu, 37%. Similarly, the proportion of women whose blood pressure (BP) was measured during the first antenatal visit to a health facility was 53% compared to the state average of 78%. PIH among this population remains unexplored, and interventions to detect the same early is the need of the hour. Measuring BP at a location closer to the community will aid in higher BP monitoring coverage and help detect PIH. Anganwadi centers could be utilized to bring these services closer to the community. The present study explores the effectiveness of community-based BP measuring by Anganwadi workers among the pregnant women in compliance to BP monitoring and describing the acceptance of the beneficiaries and the Anganwadi workers on community-based monitoring of BP.

**Methods**

After obtaining approval form ICMR-National Institute of Epidemiology ethics committee, a quasi-experimental study was conducted in the Viralimalai block in the Pudukottai district between December 2016 and March 2017 and included all the pregnant women availing services from the anganwadi. The study block had 5 Primary Health Centers, 21 Health Subcenters, 135 Anganwadi Centers. Anganwadi workers and health care providers were trained on PIH causes, prevention, complications, and to measure BP using an electronic BP instrument. The field workers informed pregnant women in the community about the availability of BP measurements at Anganwadi centers. BP monitoring cards were designed and distributed to the pregnant women, and records were maintained at the Anganwadi centers. Thirteen trained field investigators interviewed all pregnant women, anganwadi workers, and health providers in the study block with a structured questionnaire. Interviews were conducted in the community using a structured questionnaire in regional language after getting informed consent from the pregnant women, healthcare providers and Anganwadi workers. Social-demographic characteristics, obstetric and gynecologic characteristics, and history of pre-existing hypertension were recorded. Details of the antenatal check-up were also recorded. Data was entered and analyzed in Epi info software. We estimated the level of compliance to BP monitoring in a pre and post-intervention setting and used Mc Nemars' s chi-square test to bring out the significance in the difference between the same. The proportion of beneficiaries who showed acceptance to the new community-based BP screening were also represented with its 95% confidence interval.

**Results**

A total of 739 pregnant women were included from the study block. A majority (599, 81%) of the participants belonged to the age group 21 to 29 years. Fifty-seven percent (421) were primigravida, and 522 (66 %) women had an educational qualification equal to or above high school. Nearly half (48%) were staying at a distance less than 5 Kms from the anganwadi. Ninety-four percentage (693 participants) reported to the health facility for BP monitoring, of which 80% were screened at the primary health centers. Out of 739 pregnant women, 33% were aware of BP recordings during recommended visits, and 27% of pregnant women were aware of possible BP elevation during pregnancy and PIH. 25% of pregnant women were aware of PIH complications too. Out of 739 pregnant women, 37% preferred Anganwadi workers, while the rest 32% preferred doctors, and 4% preferred self-monitoring of BP. In the study block, out of the 739 pregnant women recruited, 132 (18%) mothers before the intervention and 735 (99%) post-intervention were complaint to the recommended frequency of BP recording, and it was statistically significant (P<0.05). Acceptance of intervention was observed among 721(98%) beneficiaries, all healthcare providers, and more than half (43(52%)) of the Anganwadi workers. Univariate analysis revealed that distance from residence to primary health center less than 5 km [OR 13.3; 95% CI 7.5-23.8,p < 0.05] and to Health Sub-center less than 2 km [OR 12.5,95% CI 6.9-22.6,p < 0.05) were significant determinants for accessibility of BP monitoring among pregnant women. Nearly half (39, 48%) of the Anganwadi workers opined difficulties accepting the community-based intervention package among Anganwadi workers due to increased workload.

**Conclusions**

The community-based intervention package for BP monitoring among pregnant women was useful through Anganwadi centers. Acceptance was found to be high among beneficiaries and healthcare providers. We recommend implementing and evaluating this intervention package at a larger scale and for a broader period.

**References**

1. Sajith M, Nimbargi V, Modi A, Sumariya R, Pawar A. Incidence of pregnancy induced hypertension and prescription pattern of antihypertensive drugs in pregnancy. Int J Pharma Sci Res. 2014;23:4.

2. Vigil-De Gracia P, Montufar-Rueda C, Ruiz J. Expectant management of severe preeclampsia and preeclampsia superimposed on chronic hypertension between 24 and 34 weeks' gestation. Eur J Obstet Gynecol Reprod Biol. 2003 Mar 26;107(1):24-7. doi: 10.1016/s0301-2115(02)00269-5. PMID: 12593889.

3. Dekker G, Sibai B. Primary, secondary, and tertiary prevention of pre-eclampsia. Lancet. 2001 Jan 20;357(9251):209-15. doi: 10.1016/S0140-6736(00)03599-6. PMID: 11213110.

4. Nyfløt LT, Ellingsen L, Yli BM, Øian P, Vangen S. Maternal deaths from hypertensive disorders: lessons learnt. Acta Obstet Gynecol Scand. 2018 Aug;97(8):976-987. doi: 10.1111/aogs.13357. Epub 2018 May 8. PMID: 29663318.

5. International Institute of Population Sciences. District Level Houshold and Facility Survey-4, State Fact Sheet Tamil Nadu 2012-13. http://rchiips.org/pdf/dlhs4/report/TN.pdf Accessed 10 Aug 2020.

**Table 1 (abstract A42).** Effectiveness of BP monitoring for pregnant women in Viralimalai block, Pudukottai district, Tamil Nadu, 2016-17

		After intervention		
		Complaint	Non-complaint	Total
Before intervention	Complaint	128	4	**132 (18%)**
	Non-complaint	607	0	**607**
		735 (99%)*	**4**	**739**

*(p<0.0000) statistically significant

## A43 Evaluation of Kala-Azar Surveillance System in Muzaffarpur District, Bihar, India, 2019

### Abhishek Mishra^1^, Amol Patil^2^, Nupur Roy^3^, Sushma Choudhary^2^, Tanzin Dikid^1^, Sudhir Jain^1^, Sujeet Singh^1^

#### ^1^National Centre for Disease Control, New Delhi, India; ^2^South Asia Field Epidemiology and Technology Network (SAFETYNET), India; ^3^National Vector Borne Disease Control Programme, New Delhi, India

##### **Correspondence:** Abhishek Mishra (abhishekmishra85@gmail.com)

**Background**

*Visceral leishmaniasis*, also known as Kala-Azar, is a neglected tropical disease targeted for global elimination by 2030. In 2005, the Government of India, Bangladesh and Nepal supported by WHO launched a regional Kala-Azar elimination initiative to reduce the annual incidence of Kala-azar to less than 1 case per 10,000 population at block PHC level [1], In 2014 the penta-lateral MoU was also signed between India, Bangladesh, Nepal, Bhutan and Thailand in this regard. Currently in India, 54 districts from four states namely Bihar, Jharkhand, West Bengal and Uttar Pradesh are endemic for Kala Azar.

Kala-Azar Surveillance which is the mainstay of Kala-Azar elimination programme in India is coordinated by the National Vector-Borne Disease Control Programme (NVBDCP), headquarters at Delhi. The District Kala-Azar surveillance system captures cases through active house-to-house survey and passively by laboratory reporting through Kala-azar Management Information System (KAMIS) portal. Health facilities reports the cases through paper-based reporting system to the District vector borne disease control officer every month. Active case detection is done by the block primary health center (PHC), before and after indoor residual spray (IRS) twice a year. Passive case detection is done by reporting rk39 positive cases through KAMIS portal and monthly by paper-based reporting format to district vector-borne control officer. Data collected through KAMIS portal is compiled and analysed at district level on monthly basis. Cases in the system reports to the block PHC /District hospitals, where they are screened for Kala Azar as per the NVBDCP guidelines. The first drug of choice for treatment is single dose Liposomal Amphotericin B followed by capsule Miltefosine and injection Amphotericin B.

Muzaffarpur District of Bihar is endemic for Kala-Azar since 1972. We evaluated Kala-Azar surveillance system of Muzaffarpur with the objectives to assess the Kala-Azar surveillance system against selected attributes, and to provide evidence-based recommendations.

**Methods**

Based on Kala-azar endemicity, we selected two blocks (A and B) of Muzaffarpur District. We conducted key informant’s interview and reviewed surveillance reports, KAMIS portal, reports from private health facilities, and patient treatment card. We evaluated simplicity of surveillance system by calculating proportion of interviewed staff having knowledge of Kala-Azar case definition and reporting formats. For stability we calculated proportion of Kala-Azar Technician (KTS) filled positions against sanctioned and availability of RK 39 at the time of visit. Acceptability was assessed by the proportion of health facilities conducting surveillance. For data quality we calculated the proportion of surveillance reports with incomplete information. Timeliness was assessed by proportion of reports submitted before deadline i.e. First day of every month. For representativeness attribute, we calculated proportion of sub-centres reporting Kala-Azar suspected cases and proportion of Government and private health facilities reporting Kala-Azar suspected/ confirmed cases. Flexibility attribute was determined by change/update in the reporting forms and KAMIS web portal as per the needs. Positive-Predictive value (PPV) was calculated by positivity rate by rk39 kit i.e. Proportion of probable cases out of total suspected cases during January 2018-December 2018. Usefulness was assessed by proportion of Kala-Azar cases detected within one-month of fever onset during January-December 2018. We used Epi-Info version 7.2 for analysis.

**Results**

Around 90% (12/13) of the staff interviewed were aware about Kala-Azar case definition and 100% about reporting formats. All the KTS sanctioned positions (2/2) were filled. RK39 kits were not available at block A but available at Block B. All the 20 healthcare facilities conducted active case surveillance before each round of Indoor Residual Spray. Missing data was observed in 80% (52/65) reporting formats at the district level. Seventeen-per cent (5/29) and 97% (35/36) of the reports were received on time from Block-A and Block-B respectively. PPV was <1% (1/86) and 11% (80/708) for Block A and B respectively. All the 33 subcenters were reporting suspected Kala-Azar cases but the proportion of private healthcare facilities reporting Kala-Azar cases was <1% (2/256). Surveillance system was flexible as data reporting portal was updated three times. In 2018, more than 75% of the cases were captured by surveillance system within one month of onset of symptoms.

**Conclusions**

Surveillance system in Muzaffarpur District was simple, acceptable, flexible, and useful. Stability, data quality, timeliness, predictive-value-positive and representativeness (private health facilities) needs improvement (Table 1). We recommended rapid diagnostic kit for Kala-Azar must be made available throughout the year in all Kala-Azar endemic blocks. The District Vector-Borne Disease Control Officer during programme review meetings should sensitize the Medical Officer in charge and KTS regarding importance of timeliness of sending report, even if it is “Nil”. Private healthcare facilities and laboratories should be encouraged to report the cases.

**Reference**

1) World Health Organization, India. Accelerated plan for Kala-Azar elimination 2017|National Vector Born Disease Control Programme| Govt. of India. [https://www.who.int/leishmaniasis/resources/Accelerated-Plan-Kala-azar1-Feb2017_light.pdf] Accessed 9 August 2019.

**Table 1 (abstract A43).** Summary of Kala Azar surveillance system evaluation against selected attributes, Muzaffarpur, Bihar, 2016-18

Attributes	Summary
Simplicity	Surveillance system simple to operate
Stability	Not stable, especially in block A due to logistics and HR constrains
Acceptability	Acceptable to government health facility
Data quality	Average
Timeliness	Poor for Block A because of lack of priority and resources, like net connectivity
Representativeness	Representative only for government health facilities
Flexibility	Flexible
Predictive Value Positive	Low
Usefulness	Useful in detecting early cases but scope for improvement

## A44 Description and Evaluation of the recording and reporting component of Programmatic Management of the Drug resistance Tuberculosis (PMDT) in one district, Himachal Pradesh India 2017

### Vishal Thakur^1^, Parasuraman Ganeshkumar^2^

#### ^1^Directorate of Health Services, Himachal Pradesh, India; ^2^ICMR National Institute of Epidemiology, Chennai, Tamil Nadu, India

##### **Correspondence:** Parasuraman Ganeshkumar (ganeshkumardr@gmail.com)

**Background**

India tops the 22 countries in WHO’s 30 high TB burden countries, which accounted for 87% of the world’s tuberculosis cases. India is among 30 countries with a high TB burden, high HIV/TB burden, and high MDR-TB burden. Ten countries accounted for 80% of the 3.6 million global gaps of reported tuberculosis cases compared to estimated new cases, the top three being India (26%), Indonesia (11%), and Nigeria (9%)[1]. Regarding the reporting mechanism, a systematic delay in data transmission in the program is predominantly based on the paper-based transmission of information. There are gaps across the patient care cascade on account of under-reporting, diagnostic delays, unsupported treatment, and catastrophic out of pocket expenditure to patients[2]. Himachal Pradesh is a northern state of India with most parts covered with hills. The prevalence of MDR TB was more than 9% in Himachal Pradesh than the global prevalence of 3-5%[3]. To combat MDR TB, the Government of India launched programmatic management of drug resistance tuberculosis(PMDT)[4]. One of the five components of PMDT is the Recording and Reporting system for PMDT services, which would monitor the performance and Evaluation of treatment outcome. National Strategic Plan for TB emphasizes the role of a functional reporting system with information technology tools.[5] Hence we conducted a program evaluation with the following evaluation question, “What is the status of the Recording and Reporting System for the Programmatic Management of the Drug resistance Tuberculosis (PMDT) in one district, Himachal Pradesh India?” We described the Programmatic Management of Drug-Resistant Tuberculosis and evaluated the recording and reporting component of Programmatic Management of Drug-Resistant Tuberculosis in one district of Himachal Pradesh.

**Methods**

We conducted a cross-sectional study in seven tuberculosis units (TU) of one of the Himachal Pradesh districts selected by systematic random sampling out of 13 tuberculosis units. The population covered by each TU ranged from 85,100 to 153,282. We included the designated microscopy centers (DMC), tuberculosis units, and the district TB center. Quarterly reports, registers, and records in these centers were reviewed and data was collected by data abstraction form. We interviewed the stakeholders of the data management and program managers in the TUs using a semi-structured questionnaire. We conducted a facility survey using checklist verification related to the reporting and recording system.

Input, process, and output Indicators were identified in the reporting and recording system of a TU. We estimated the median duration for sample collection, case confirmation, and treatment initiation. We estimated the proportion of MDR TB patients on detection and initiation of treatment. Availability of registers and records in each facility was expressed as proportions. We obtained ethics approval from the Institutional ethics committee of ICMR National Institute of Epidemiology. Written informed consent was obtained from each participant before the initiation of the interview.

**Results**

We observed paper-based forms and registers are used in the peripheral health center and designated microscopic centers to record and report the information. The data collated at the level of the tuberculosis unit was in electronic format. In each center, seven registers were maintained to generate six-monthly reports related to MDR TB. During our study, there were 20 MDR TB patients under treatment in the selected TUs. We observed that treatment details were updated in eight out of 20 MDR-TB patients at the designated microscopic center. The majority of the Culture and drug sensitivity registers (82%), laboratory registers (72%), and stock registers (75%) were found to be complete. Of these 20 patients, we observed there were delays in the duration of sample collection (0-7 days), confirmation of diagnosis (0-21 days), and initiation of treatment (0-45 days) in the selected study sites. The maximum delay for sample collection of suspected MDR TB was seven days, and confirmation was 14 days (Figure-1). The maximum delay observed to initiate the treatment of MDR TB was 45 days. In the study, TUs 13 out of 17 centers had a functional computer. Treatment cards were updated for 12 out of 20 patients in the selected TUs.

**Conclusion**

Treatment registers were not frequently updated, and there was a delay in reporting MDR TB testing diagnosis and initiation of treatment. Lack of computers with internet observed in certain DMCs. Treatment cards were available in the center, but more than one-third of the patient's information was not updated. We recommend strengthening the recording and reporting component of PMDT needs by intensified training and prompt reporting. We suggest introducing an information technology system at all levels of reporting promptly and generate the indicator on time.

**References**

1. World Health Organization. Global tuberculosis report 2018. Geneva: World Health Organization; 2018. Licence: CC BY-NC-SA 3.0 IGO.

2. India TB Report 2020; National Tuberculosis Elimination Program. Central TB Division, Ministry of Health and Family Welfare, Nirman Bhawan; New Delhi: 110011. March 2020. Available from:http://www.tbcindia.gov.in Annual Report.

3. Kumar A, Singh AK, Upadhyay V, Pandey J. Epidemiology of multi-drug-resistant tuberculosis in Northern India. Biomedical and Biotechnology Research Journal (BBRJ). 2018 Apr 1;2(2):112.

4. Central TB Division. Guidelines on Programmatic Management of Drug Resistant TB (PMDT) Ministry of Health and Family Welfare, Government of India, New Delhi; 2017 [cited 2020 Aug 10]. Available from: https://tbcindia.gov.in/WriteReadData/Guideline%20for%20PMDT%20in%20India%202017.zip

5. Central TB Division, Directorate General of Health Service, Ministry of Health and Family Welfare, et al. National strategic plan for tuberculosis elimination 2017–2025 [Internet]. New Delhi, India; 2017 [cited 2020 Aug 10]. p. 1–105. Available from: https://tbcindia.gov.in/WriteReadData/NSP/Draft/20.02.2017/1.pdf.

**Fig. 1 (abstract A44).**
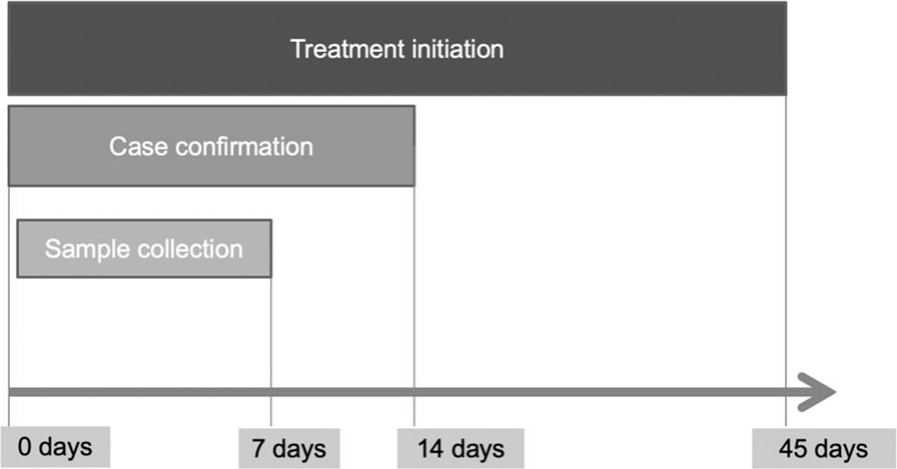
Range of Time lag observed in MDR TB patients from confirmation to onset of treatment (in days) in one district of Himachal Pradesh

## A45 Trend of Infant Mortality Rate in Dindigul district, Tamil Nadu, India, 2014-19

### Shanmuganathan Shankarprasath^1,2^, Manickam Ponnaiah^1^, Polani Rubeshkumar^1^, Manoj Murhekar^1^

#### ^1^ICMR-National Institute of Epidemiology, Chennai, Tamil Nadu, India; ^2^Directorate of Public Health and Preventive Medicine, Tamil Nadu, India

##### **Correspondence:** Shanmuganathan Shankarprasath (shankarprasath87@gmail.com)

**Background**

Infant Mortality Rate (IMR) is considered a primary measure of public health for countries worldwide. It is associated with various factors such as maternal health, quality and access to medical care, socioeconomic conditions, and public health practices [1]. The Sample Registration System (SRS) of India reported a decline in the IMR from 72/1000 live births in 1998 to 57/1000 live births in 2006, which further declined to 39/1000 live births in 2014. This slow reduction in IMR in the last couple of decades is a significant concern for India, as it is still high compared to the other developing nations. There is a considerable variation in performance between the states in India [2]. IMR of the states such as Tamil Nadu and Kerala are lower than the national average. In comparison, states like Uttar Pradesh, Madhya Pradesh, Bihar, and Rajasthan reported higher IMR than the national value [3]. In Tamil Nadu, the Dindigul district is one of the districts with a high IMR. Dindigul reported an IMR of 14 per 1000 live births during April 2018 - January 2019, while the state IMR was 9 per 1000 live births for the same period. The epidemiological analysis of Infant deaths could be useful in making policy decisions. We aimed to analyze infant deaths in the district of Dindigul and describe the causes and trends of the same between 2014 & 2019.

**Methods**

We conducted a cross-sectional study in Dindigul, Tamil Nadu, and included infants born in the study place between 2014-19 into the study. Dindigul district had a population of 2,349,184 in 2019 and has 63 Primary Health Centres and13 secondary health care centers. Dindigul District has 14 community blocks. We gathered the information about the demographics, infant deaths, causes of deaths from the Public Health Department's records in the district. The Tamil Nadu Health Management Information System Portal was assessed for live births data.

Death of an infant before its first birthday was defined as an infant death. We categorized these deaths into three categories as follows: 1) Early Neonatal death (≤7 days), 2) Late neonatal death (8-28 days), and lastly, Post neonatal death (29-365 days) based on the age of the infant at the time of death. We also categorized the causes of infant deaths into 11 categories. The categories were as follows: 1. Birth Asphyxia, 2. Low birth weight (LBW) and prematurity 3. Congenital heart diseases (CHD), 4. Septicemia, 5. Aspiration of Milk, 6. Acute Respiratory Distress Syndrome (ARDS), 7. Congenital Anomalies, 8. Bronchopneumonia, 9. Central Nervous system (CNS) cause 10. MAS, and 11. Accidents. We calculated Infant Mortality Rate (IMR) with the number of infant deaths during the year as a numerator and total live births during the period as denominator multiplied by 1000. We also computed IMR by age, gender, cause of death, and days of life. We calculated proportions for the infant deaths by community block and place of death. We used epi-info (Ver. 7.2) for data management and analysis.

**Results**

There were 1,35,190 live births and 1643 infant deaths reported from 2014 to 2019 in Dindigul District, Tamil Nadu. Among the 1643 casualties, 927 (56%) were male infants, 816 (50%) were early neonatal deaths, 303 (18%) were post-neonatal deaths. 1148 (69.9%) deaths occurred in hospitals, while 338 (20.6%) deaths were reported from home. IMR for the Dindigul district was 12 per 1000 live births during the period 2014-19. The IMR were 14, 12, 10, 12, 14, 12 per 1000 live births from 2014 to 2019. Throughout the reference period, the IMR of the male child (Range: 11 to 15 per 1000 live births) was higher than the female child (Range: 8 to 14 per 1000 live births) except in 2019 (11 vs. 12 per 1000 live births). IMR was persistently higher in the blocks of Vadamadurai (19 per 1000 live births) and Natham (17 per 1000 live births) throughout the reference period. The deaths were higher during the early neonatal period (6 per 1000 live births) followed by the post-neonatal period (4 per 1000 live births) (figure 1). Among the causes of infant deaths, birth asphyxia and LBW prematurity were higher than others. Birth asphyxia (19.5%) and LBW prematurity (19.4%) were the leading causes of early neonatal deaths. Milk aspiration syndrome (8.5%) was the leading cause of late neonatal deaths during septicemia (10%) for the post-neonatal deaths.

**Conclusions**

Early neonatal deaths were considerably higher in the Dindigul District. Birth Asphyxia, low birth weight & prematurity were the most common causes of deaths in the early neonatal period. Bronchopneumonia, Sepsis, and Aspiration pneumonitis were the most common causes of deaths in the post neonatal period. We recommended strengthening the early neonatal care and post-neonatal care in the secondary care centers.

**References**

1. Reidpath DD, Allotey P. Infant mortality rate as an indicator of population health. J Epidemiol Community Health. 2003 May;57(5):344-6. doi: 10.1136/jech.57.5.344. PMID: 12700217; PMCID: PMC1732453.

2. Bhatia M, Dwivedi LK, Ranjan M, Dixit P, Putcha V. Trends, patterns and predictive factors of infant and child mortality in well-performing and underperforming states of India: a secondary analysis using National Family Health Surveys. BMJ Open. 2019 Mar 20;9(3):e023875. doi: 10.1136/bmjopen-2018-023875. PMID: 30898805; PMCID: PMC6475182.

3. Singh A, Pathak PK, Chauhan RK, Pan W. Infant and child mortality in India in the last two decades: a geospatial analysis. PLoS One. 2011;6(11):e26856. doi: 10.1371/journal.pone.0026856. Epub 2011 Nov 2. PMID: 22073208; PMCID: PMC3206872.

**Fig. 1 (abstract A45).**
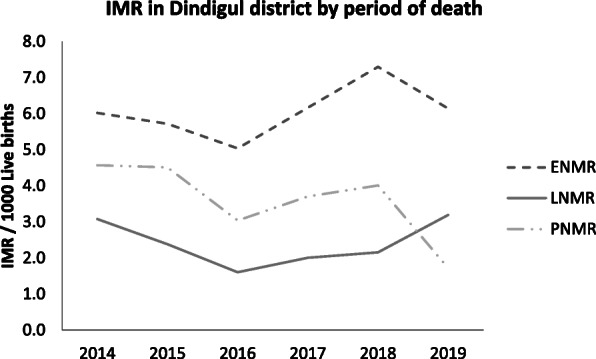
Trend of Infant Mortality Rate, Dindigul District, Tamil Nadu, India, 2014-19

## A46 Evaluation of a symptom-based surveillance model for Ebola virus disease at Indira Gandhi International Airport, Delhi, India 2014–2015

### Chandrakant Moghe^1^, Prabha Arora^1^, Meera Dhuria^1^, Sujeet Kumar Singh^2^, Sanjay Mattu^2^, Naresh Jakhar^2^, Ekta Sahora^3^, Samir Sodha^3^, Srinivas Venketesh^1^

#### ^1^National Centre for Disease Control, Delhi, India; ^2^Ministry of Health and Family Welfare, Delhi, India; ^3^U.S. Centers for Disease Control and Prevention, Delhi, India

##### **Correspondence:** Chandrakant Moghe (c_moghe@rediffmail.com)

**Background**

Ebola virus disease (EVD) is a zoonotic disease that presents as an acute illness in humans with a case fatality rate ranging from 25% to 90% [1]. From 2014–15, the EVD outbreak in Guinea, Liberia, Nigeria, and Sierra Leone resulted in 28,616 illnesses and 11,310 deaths [2]. Because of the initial difficulty in containing the EVD outbreak, the World Health Organization declared a Public Health Emergency of International Concern on 8 August 2014 in accordance with the 2005 International Health Regulations [3, 4].

To prevent EVD importation to India by people travelling from affected countries, the Government of India implemented the WHO-recommended screening guidelines for EVD. All passengers from EVD-affected countries completed a self-declaration form upon arrival and were screened using thermal scanners at points of entry, including at Indira Gandhi International (IGI) Airport, New Delhi, India. We evaluated the EVD surveillance system IGI Airport from August 2014 to October 2015 to assess the attributes and recommend measures to strengthen the system.

**Methods**

The evaluation was conducted at IGI Airport, New Delhi, from January to February 2016. The U.S. Centers for Disease Control and Prevention evaluation framework for public health surveillance was used to evaluate the EVD surveillance system attributes of usefulness, simplicity, flexibility, data quality, acceptability, timeliness, and stability [5]. We interviewed key informants at the Ministry of Health and Family Welfare (MOHFW), National Centre for Disease Control (NCDC), and IGI Airport including directors, immigration officers, medical officers, laboratory and data collection staff. We reviewed surveillance protocols and evaluated the operation of the system via reporting patterns from the airport and NCDC laboratory and oversight mechanism at the MOHFW.

We reviewed 312 randomly selected self-declaration forms, flight records, and suspected case records, and all daily and weekly surveillance reports and laboratory reports from 1 August 2014 to 31 October 2015. Passengers were classified into one of four categories based on the paper self-declaration form: high risk if the person had history of travel to an EVD-affected country (i.e., Guinea, Liberia, or Sierra Leone) in the past 21 days *and* signs or symptoms of EVD; medium risk if the person had history of travel to an EVD-affected country in the past 21 days *and* contact with a known EVD case *and* no signs or symptoms of EVD; low risk if the person had history of travel to an EVD-affected country in the past 21 days only; and no risk if the person had no history of travel to an EVD-affected country in the past 21 days.

A suspected case of EVD was defined as person who had an elevated body temperature by thermal scan *or* reported subjective fever *or* signs or symptoms of EVD (i.e., severe headache, fatigue, muscle pain, vomiting, diarrhoea, abdominal pain, unexplained haemorrhage) *and* history of travel to an EVD-affected country in the past 21 days. High risk, suspected cases were referred for examination, Ebola virus testing, and hospital admission. Clinical samples were sent to NCDC, New Delhi, and National Institute of Virology, Pune, for Ebola virus testing.

**Results**

There were no non-stop flights arriving at IGI Airport from the four EVD-affected West African countries. Between 1 August 2014 and 31 October 2015, 798,756 passengers were screened at IGI Airport. A total of 18,518 (2.3%) reported travel to an EVD-affected country in the past 21 days. There were 83 high-risk suspected cases of EVD, 5 medium-risk passengers, and 18,430 (99.5%) low-risk passengers. One (1.2%) of the 83 high-risk suspects had laboratory-confirmed Ebola virus. The month with the largest number of passengers with an EVD exposure risk was December 2014; the number dramatically declined after March 2015 (Figure 1).

The EVD surveillance system was simple and flexible. The self-declaration form was concise, uniform, but only available in English. All informants found the form easy to use, including recording passenger temperatures. The MOHFW used the surveillance data collected and analysis to make changes to the system in real time. The number of airlines identified for passenger screening was revised twice during the surveillance period, and the reporting frequency was revised from daily reporting to weekly reporting when the number of passengers at risk of exposure decreased after March 2015. Screening was initially completed after immigration, but it was changed after 2 weeks prior to immigration to make the process more efficient. When the number of passengers increased, the number of screening counters were increased through the deployment of additional medical officers. Staffing was maintained by commissioning medical officers from New Delhi-area municipal hospitals.

Of the 312 self-declaration forms we reviewed, 98% (n=306) were complete. All 249 daily and 28 weekly reports reviewed were found to be complete for the 17 daily and 10 weekly variables collected, and 100% of reports prepared by IGI Airport were sent to the MOHFW and received within the specified timeframe. All 83 high-risk suspect case files and laboratory results were complete. The average time for reports from the designated laboratories to IGI Airport was less than 24 hours.

Our evaluation had limitations. We were not able to interview cabin crews or airport ground staff due to security and unavailability. Contracted medical officers were not available for interview. Records to assess down time of the thermal camera and computers were not available for review. We could not calculate sensitivity due to lack of a denominator nor could we calculate predictive value positive for cases.

**Conclusions**

Overall, the surveillance system implemented to prevent importation of Ebola virus to India was useful for detecting high-risk, suspected cases. Surveillance allowed for the identification, diagnosis, treatment, and isolation of an imported case of EVD and prevented secondary transmission. The EVD surveillance system was simple, flexible, timely, and captured quality data. The surveillance system was well supported by the IGI Airport Immigration Department and NCDC laboratory. The use of the self-declaration form in English posed problems for some passengers. During future airport surveillance system implementation, we recommend making forms available in local languages to encourage reporting by passengers.

**References**

1. World Health Organization. Fact sheets: Ebola virus disease. Geneva: World Health Organization, 2020. [https://www.who.int/en/news-room/fact-sheets/detail/ebola-virus-disease] Accessed on 23 February 2020.

2. World Health Organization. Situation report: Ebola virus disease,10 June 2016. Geneva: World Health Organization, 2016. [https://www.who.int/csr/disease/ebola/en/] Accessed on 23 February 2020.

3. World Health Organization. Statement on the 1st meeting of the IHR Emergency Committee on the 2014 Ebola outbreak in West Africa. Geneva: World Health Organization, 8 August 2014. [https://www.who.int/mediacentre/news/statements/2014/ebola-20140808/en/] Accessed on 23 March 2020.

4. World Health Organization. International Health Regulations (2005). Third edition. Geneva: World Health Organization, 2016. [https://www.who.int/ihr/publications/9789241580496/en/] Accessed on 23 March 2020.

5. Guidelines Working Group, Centers for Disease Control and Prevention. Guidelines for Evaluating Public Health Surveillance Systems: Recommendations from the Guidelines Working Group. MMWR. 2001; 50(RR13):1–35). [https://www.cdc.gov/mmwr/preview/mmwrhtml/rr5013a1.htm] Accessed on 23 February 2020.

**Fig. 1 (abstract A46).**
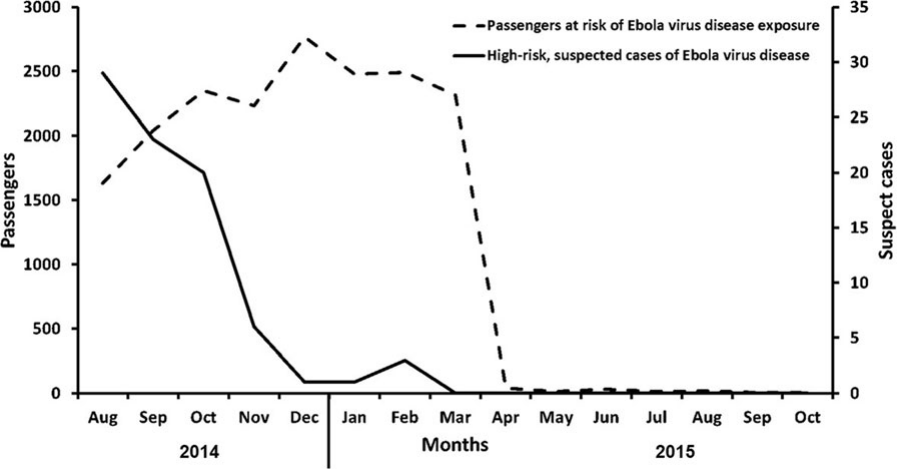
Number of passengers with an Ebola virus disease exposure risk and number of high-risk, suspected Ebola virus disease cases identified at Indira Gandhi International Airport, New Delhi, India from 1 August 2014 to 31 October 2015

## A47 Malaria outbreak with Plasmodium vivax preponderance in a tea garden despite high usage of LLINs in Kumargram block of Alipurduar district, West Bengal, India, 2019

### Puran K Sharma, Subarna Goswami, Kousik Choudhury, Ananta Maji, Golam Mortuja Sk

#### Office of the Chief Medical Officer of Health, Department of Health & Family Welfare, Government of West Bengal

##### **Correspondence:** Puran K Sharma (puran.sharma611@gmail.com)

**Background**

Malaria outbreaks occur due to various reasons such as an increase in mosquito breeding sites, breakdown of surveillance, migration of infected persons, arrival of new vectors, breakdown of vector control measures, and parasite and vectors [1]. In India, the National Malaria Control Programme (NMCP) interventions include indoor residual spray with Dichloro-diphenyl-trichloroethane (DDT) and other insecticides, insecticide-treated nets, larval control and case detection and effective treatment [2]. The Alipurduar district, located in the northern part of West Bengal (India) is endemic for Malaria, has 63 tea gardens with a predominantly tribal population. Along with Jalpaiguri district from which it was carved out, the district contributed to 50-60% of the state's malaria case and deaths in the past [3]. Rydak tea garden in Kumargram block of Alipurduar district had reported several malaria outbreaks in the previous years, with predominantly Plasmodium falciparum. As a part of the NMCP, all the households of Rydak were saturated with Long Lasting Impregnated Nets (LLIN), as it was one of the high-risk areas in the Alipurduar district. Seasonal DDT spray works were not taken up since 2017 in Rydak as per the NMCP, given the near-complete coverage of LLIN. During September 2019, the district surveillance unit observed increase in malaria cases at Rydak. We investigated the outbreak to assess its magnitude, review the retention and consistent use of LLINs in the households, and identify the vector responsible for the outbreak to guide appropriate control measures.

**Methods**

We conducted a case search at Rydak by a house-to-house survey. We used the standard definition of the suspect and confirmed case of Malaria, given by the Integrated Disease Surveillance Program (IDSP). We defined a confirmed case-patient of Malaria as an acute febrile illness with a peripheral blood smear (thick and thin) positive for malaria test or a bi-valent rapid diagnostic antigen kit (RDK) positive for Plasmodium vivax or falciparum or both in Rydak from 19 September 2019 to 9 December 2019. We collected blood from individuals with fever and conducted the microscopic examination of blood slides after standard staining procedures, or RDK test of fever case-patients to confirm malaria cases and detect species of parasite. The attack rate of Malaria by age and sex were calculated using the 2011 census data as denominators. The annual blood examination rate (ABER) for the past five years, was calculated by dividing the total number slides examined for malaria parasites in each year plus the number persons tested using the RDKs in that year divided by the total population and expressed as a percentage. We visited all the households and physically verified the presence of LLINs in the households in the Rydak in 2017 and interviewed residents of the tea garden for consistent usage of the same. We collected adult Anopheles mosquitoes from human dwellings and cattle sheds in the most affected areas using the suction method and identified mosquito species using standard mosquito identification keys, without estimating density. We also collected mosquito larvae from potential vector breeding sites and reared them in the laboratory for mosquito species identification. We conducted parasite incrimination from gravid female Anopheles mosquitoes

**Results**:

We visited 1647 households at Rywad for the survey and interviewed 9002 individuals. Overall, 300 Plasmodium vivax and one mixed infection (Plasmodium vivax – Plasmodium falciparum) were reported with no deaths during the current outbreak. The overall attack rate was 3% with male preponderance (55%). Attack rates in the age group 0-4, 5-14 and 14-29 years were 4%, 6% and 4% respectively (Table 1). The annual blood examination rate was consistently above 20% for the last five years. Among the 102 mosquitoes collected, 64 (63%) were Anopheles annularis, 28 (27%) were Anopheles vagus, 7 (7%) were Anopheles aconitus, and 3 (3%) were Anopheles minimus. Parasite incrimination did not yield any parasite. Of the 4441 LLINs distributed in 2017, 4428 (99.7%) were retained in the households, and 4406 (99.9%) LLINs were consistently used.

**Conclusion**

The attack rate distributed across different age groups points toward the district being in the elimination phase. The current Malaria outbreak is predominantly due to Plasmodium vivax, similar to three previous outbreaks in Alipurduar. This phenomenon of an increasing proportion of Plasmodium vivax cases with decreasing total Malaria cases has been observed in other elimination settings [4]. In the NMCP campaigns, Plasmodium falciparum is often the first malaria species to show a decline in incidence with Plasmodium vivax being slower to respond. It could be due to the longer extrinsic incubation period of Plasmodium falciparum than Plasmodium vivax, making the former more susceptible to LLINs and Indoor Residual Spray (IRS) [4]. Also, the infectious gametocytes of Plasmodium vivax appear early in blood, in contrast to Plasmodium falciparum, in which mature gametocytes take ten days to appear in the peripheral blood after asexual parasite patency. Thus, by the time a Plasmodium vivax infected patient presents for diagnosis and treatment, the onward transmission of the parasite could already have occurred. Thus, the early diagnosis and treatment strategies highly effective for Plasmodium falciparum may not be so for Plasmodium vivax [4]. Hence, it is recommended that malaria transmission strategies, which have so far been directed more towards the control of Plasmodium falciparum, be directed towards effective and sustained interventions against Plasmodium vivax. The current outbreak of Malaria despite high retention (99.7%) of the distributed LLINs and their reported consistent usage (99.9%) could be due to outdoor infection or resistance of vectors to pyrethroids. The NMCP recommends that IRS and LLINs be implemented considering major vectors to be mainly indoor biting, indoor resting and endophage [5]. However, reports from several countries, including India, show a drift in mosquito behaviour to rest outdoors due to insecticide interventions, especially LLINs [5]. Majority of the mosquito vectors identified in this study were also exophilic and exophagic, suggesting the need for alternative vector control measures.

**Acknowledgements**

We are grateful to the Department of Health & Family Welfare, Government of West Bengal, the District & Kumargram Block administration in Alipurduar and all Health Workers of Alipurduar for the support during the study

**References**

1. Nájera JA, Kouznetsov RL, Delacollette C, Programme WHOMP and C. Malaria epidemics: detection and control, forecasting and prevention. 1998 [cited 2021 Feb 8]; Available from: https://apps.who.int/iris/handle/10665/64427

2. Sharma PK, Ramanchandran R, Hutin YJ, Sharma R, Gupte MD. A malaria outbreak in Naxalbari, Darjeeling district, West Bengal, India, 2005: weaknesses in disease control, important risk factors. Malaria journal. 2009 Dec;8(1):1-8.

3. Sharma PK, Sen T, Ramakrishnan R, Hutin Y, Murhekar M. The shift from public to private health care providers and malaria deaths in Jalpaiguri district, West Bengal, India, 2006. International health. 2009 Dec 1;1(2):148-53.

4. Howes RE, Battle KE, Mendis KN, Smith DL, Cibulskis RE, Baird JK, Hay SI. Global epidemiology of Plasmodium vivax. The American journal of tropical medicine and hygiene. 2016 Dec 28;95(6_Suppl):15-34.

5. Subbarao SK, Nanda N, Rahi M, Raghavendra K. Biology and bionomics of malaria vectors in India: existing information and what more needs to be known for strategizing elimination of malaria. Malaria journal. 2019 Dec;18(1):1-1.

**Table 1 (abstract A47).** Attack rates of Malaria by age and gender, Rydak, Kumargram block, Alipurduar, West Bengal, India 19 September 2019 – 9 December 2019

Characteristics		Population	Malaria cases	
			#	Attack rate per 100 population
Age group	0 – 4	588	25	4.3
	5 – 14	1440	89	6.2
	15 – 29	2430	99	4.0
	30 – 44	2070	37	1.8
	45 – 59	1440	32	2.2
	60+	901	19	2.1
Gender	Male	4592	166	3.6
	Female	4410	135	3
Total		9002	301	3.3

## A48 Capacity building in public health emergency & hospital preparedness in India, 2019 - An experience sharing

### Kapil Goel^1^, Pinnaka Venkata Maha Lakshmi^1^, Manish Chaturvedi^2^, Jarnail Singh Thakur^1^

#### ^1^Post Graduate Institute of Medical Education & Research (PGIMER), Chandigarh, India; ^2^National Institute of Health & Family Welfare, New Delhi, India

##### **Correspondence:** Kapil Goel (drkapil123@gmail.com)

**Introduction**

Natural disasters are a problem in India as three fourth (27/36) of the states and union territories of India are disaster prone [1]. India is prone to earthquakes (>59%), floods and river erosions (12%) and 76% of its coastline to cyclones and tsunamis. About 68% of its cultivable land is vulnerable to droughts and its hilly areas are at risk from landslides and avalanches [1].

India is also exposed to chemical, biological, radiological and nuclear (CBRN) emergencies and other man-made disasters [2]. Public health emergencies (PHE) can arise from a wide range of causes, including outbreaks of contagious, life-threatening disease, natural disasters, as well as chemical contamination of the environment and the release of radiation [3]. The central government has made it mandatory to train at-least two district health officials from each district for disaster preparedness and response.

The Government of India mandates PHE training of district health Officers (DHO) to enhance their knowledge and skills for preparedness of public health emergencies. Post Graduate Institute of Medical Education & Research (PGIMER), Chandigarh was identified as one of the nodal centres for conducting this activity. A multi-disciplinary approach was used with 21 resource persons from various fields (public health, epidemiology, environmental health, hospital administration, geography, defence studies, education, army, air force, community medicine and medicine) and institutes [PGIMER, Punjab University, National Disaster Response Force, Air Force Medical Services, Directorate of Health Services (Punjab), Department of Education (Chandigarh) and Fire Station (Chandigarh)]. Teaching faculty of PGIMER Chandigarh along with resource persons were allotted specific topics for lectures. The two weeks intensive training had 48 sessions (1.5 hours each) including 8 field visit sessions (3 hours). Training consisted of lectures, field visits, mock drills, table top exercises and disaster related videos. Participants were exposed to mock drills by National Disaster Response Force, Fire Station Chandigarh, PGIMER Nehru hospital and School evacuation drills. We conducted PHE training and an evaluation to determine if the training improved the PHE knowledge of participants.

**Methods**

We conducted a pre and post-test evaluation by using quantitative method scoring with multiple choice questions (MCQs) for knowledge assessment. We also used a self-assessment tool for self-grading competencies before and after the training course on a five point score (1-5). We did qualitative evaluation with feedback forms consisting of open ended questions and agreement with statements (using a Likert scale). All the participants after obtaining their consent, were asked to take the knowledge assessment pre-test on day one containing 35 questions on public health emergency & hospital preparedness, and the same 35 questions were provided at the end of the training course as a post-test questionnaire to assess the effectiveness of the training. One mark was given for each correct response to MCQ and no marks were deducted for wrong responses. Total pre-test scores of all participants were averaged and similarly total post test scores of all participants were averaged. Paired Student’s t-test was used for comparing pre and post-test scores. Pre and Post-test papers with MCQs, self-assessment tool and feedback forms were evaluated on the last day of training on score basis, average scores were calculated and data recorded, interpreted, and analysed in Microsoft Excel and the results were expressed as proportions.

**Results**

The first batch training (out of total 3 batches) included 30 participants from Punjab (21) and Himachal Pradesh (9) conducted between 14-26 October 2019. A total of 30 participants participated in pre- and post-test.

The mean Pre-test score was 11.6 (33.14%) out of 35 and mean Post-test score was 27.8 (79.42%). There was a net knowledge gain of 46.3% of scores before and after the training (Figure 1). Total post-test scores were highly significant (P < 0.001) than pre-test scores.

The mean self-assessment score before training was 1.24 (24.8%) out of 5 and after training was 4.00 (80%) which were statistically significant (p<0.05). There was a net gain of 55.2% of self-assessment scores before and after the training. Topics which showed maximum gain (80%) in knowledge were basic steps & format for District Disaster Management Plan, roles & responsibilities of various officers and hospital evacuation plan. Majority (63%) of participants gave feedback for conducting more field visits and reducing the duration of lectures.

**Conclusions**

There was significant improvement in the participants knowledge after post-test evaluation when compared to pre-test. The two weeks training on management of public health emergencies was based on multi-disciplinary approach. The training enhanced the knowledge of the District health officers in the area of management of Public Health Emergencies for managing and improving services in their districts. Investing in capacity building for management of public health emergencies is going to contribute substantially to safer development by considerably reducing the disaster related losses and costs in the country.

**References**

1. India – Disaster Risk Profile. [cited 2020 Mar 24]. Available from: https://nidm.gov.in/easindia2014/err/pdf/country_profile/India.pdf

2. Vulnerability Profile of India. [cited 2020 Mar 23]. Available from: https://ndma.gov.in/en/vulnerability-profile.html

3. Public health emergencies in Advancing the right to health: the vital role of law. Geneva: World Health Organization; 2017 Licence: CC BY-NC-SA 3.0 IGO. Cataloguing-in-Publication:165-176 [cited 2020 Mar 23]. Available from: https://www.who.int/healthsystems/topics/health-law/chapter11.pdf

**Fig. 1 (abstract A48).**
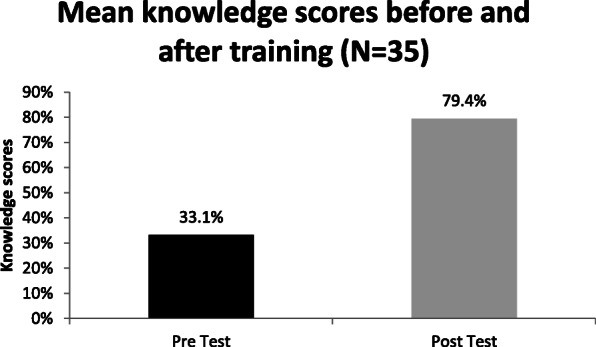
Knowledge scores before and after training program, Chandigarh, 2019

## A49 Assessment of delays in TB diagnosis and treatment – Revised National TB Control Program, India, 2014

### Prashant Bhat^1,2^, Mohan Kumar Raju^2^, Niraj Kulshrestha^3^

#### ^1^Department of Health and Family Welfare, Government of Karnataka, India; ^2^School of Public Health, ICMR-National Institute of Epidemiology, Chennai, India; ^3^Ministry of Health, Government of India, New Delhi

##### **Correspondence:** Prashant Bhat (bhatp74@gmail.com)

**Introduction**

Every year, 10.4 million people get Tuberculosis with 1.5 million deaths globally. While 22 countries contribute to more than 75% of the TB burden, India leads with 2.5 million incident cases and 480,000 deaths. TB has a major financial implication for India ($170/- direct and indirect costs per patient) [1]. Although the Revised National TB Control Program (RNTCP) has followed World Health Organisation (WHO) endorsed DOTS Strategy, averted millions of TB deaths and accomplished remarkable milestones, TB continues to be one of the worst public health problems in India.

Owing to the large population size and inadequate public-health infrastructure, Private healthcare providers provide a significant level of healthcare to such patients. TB care in the private sector is at times erratic, unethical, costly, and not compliant to WHO Standards of TB Care [2]. This leads to inconsistent and poor compliance to the treatment and hence increases infectiousness.

TB relapse rates and treatment failures are quite high in the public sector in India compared to other high burden countries [3]. These relapse and failures are speculated to be due to delays in care. Although studies showed varied delays in both the private and public sectors, there is limited information on the nationwide scenario. We aimed to assess the nationwide diagnosis and treatment delays in TB management in India with the primary objective of assessing the patient sided delay in seeking care after the onset of symptoms. We also looked at the mean delay by the provider to offer TB test and to initiate TB treatment.

**Methods**

We conducted a cross-sectional study of RNTCP data RNTCP has routine program evaluation protocol [4]. Periodically, every State evaluates the program at randomly selected districts. Central TB Division (CTD) also undertakes the program evaluations at the state level with two randomly selected districts from the selected State. RNTCP has a predesigned tool for the patient interview at home, which captures gaps in services to the patient. For monitoring purposes, RNTCP has divided the country into four zones, North, South, East and West [5]. All the Central and State evaluations of RNTCP in 2014 were included in the study. Data variables included time, place, and person attributes, questions on the number of days between the onset of symptoms to the first consultation, diagnosis and treatment initiation, number of visits between the first consultation to TB diagnosis and type of first care provider. Program evaluation data of 2014 available in CTD were doubly entered in EpiData version 3.1 in a pre-tested data capture instrument. Ten percent of the data were randomly checked for errors by comparing to the original document, corrected if any.

We obtained the Departmental approval to conduct the study. Personal identifiers were removed soon after the data collection. The data file was kept secured with password protection.

**Results**

Of 2658 abstracted interview formats, 2240 (84%) TB patients who were recorded as personally responded were included for analysis. Of them, 1411 (63%) were aged between 15-44 years. There were 1450 (64.1% [95%CI: 62.1-66.0]) males, 1611 (71.3% [95%CI: 69.4-73.1]) new smear-positive TB patients. Over half (1215, 53.8% [95%CI: 51.8-55.9]) had a cough as their chief complaints and two thirds (1434, 63.4% [95%CI: 61.4-65.3]) didn’t have any comorbidities. In the Public sector, patients approached late to the doctor (29.4 days, SD: 45.42 [95%CI: 26.3-32.5]) and treatment initiation was delayed (5.03 days, SD: 17.83 [95%CI: 3.8-6.3]). In the private sector, patients had to hop between providers before diagnosis (2.45 providers, SD: 1.62 [95%CI: 2.3-2.5]) delaying diagnosis (23.91 days from symptom onset, SD: 45.67 [95%CI: 20.5-27.3]). Overall, mean patient delay (from symptom onset to visiting clinic) varied from 20 to 31 days among RNTCP zones, the diagnostic delay from 12 to 15.5 days, treatment initiation delay from 5 to 6.5 days, and patients visited 3 to 4 providers. Patient delays were more marked in East zone (30.9 days, SD: 69.4 [95%CI: 22.0-39.7]) followed by West zone (23.6 days, SD: 37.9 [95%CI: 21.0-26.2]) (Table 1).

**Conclusion**

The nationwide analysis of delays in TB care in India using program data is one of the pioneer studies in India. The study found that while the patient sided delay and treatment initiation after diagnosis were slightly higher in the public sector, patients had to visit more doctors, and thereby the diagnosis was delayed in the private sector. Also, the patient sided delay was more marked in the East Zone.

People seek care in the private health-sector expecting better care or to avoid delays in care. Provider delay in TB diagnosis and treatment is a grave issue. Especially delayed diagnosis of TB in the for-profit private sector, forcing patients to change doctors frequently, needs attention. Several reports highlight the suboptimal TB care in private-sector in India. Recent Joint Effort to End TB initiative by the Government of India needs to emphasize the capacity building of private providers in the provision of Standard TB Care.

Economically weaker sections often seek care from the public sector. The north-East part of the country is proclaimed to be administratively neglected part of the country. Extreme delays are noted in this region. Arguably, economically weaker sections and politically backward regions severely lack health awareness leading to more delays in seeking health. Recent emphasis on Advocacy, Communication, and Social Mobilization activities through various Non-Governmental Organizations need to be consolidated to modify the health-seeking behavior in these people.

The study had certain strengths. It included the interview-data of all the Internal Evaluations of the program conducted in a year across the country. The study followed STROBE (STrengthening Reporting of OBservational studies in Epidemiology) Guidelines and adhered to sound Ethical considerations. The program secondary data used for the study, possible recall bias and the uneven state coverage are few limitations

The RNTCP internal evaluations revealed unacceptable levels of delays from the patient side to seek care and from the provider side in TB diagnosis and treatment. Focused interventions to curtail these delays need to be chalked out guided by the routine internal evaluation data.

**References**

1. Rajeswari R, Balasubramanian R, Muniyandi M, Geetharamani S, Thresa X, Venkatesan P. Socio-economic impact of tuberculosis on patients and family in India. Int J Tuberc Lung Dis. 1999: 869-877

2. Uplekar MW, Shepard DS. Treatment of tuberculosis by private general practitioners in India. Tubercle 1991;72(4):284-90.

3. Mehra RK, Dhingra VK, Nish A, Vashist RP. Study of relapse and failure cases of CAT I retreated with CAT II under RNTCP—an eleven year follow up. Indian J Tuberc. 2008 Oct 20;55(4):188-91.

4. Central TB Division, New Delhi, Supervision and Monitoring strategy in Revised National Tuberculosis Control Program 2012. Available from https://tbcindia.gov.in/showfile.php?lid=3030 (Accessed: 03/01/2018)

5. Sharma SK, Mohan A, Chauhan LS, Narain JP, Kumar P, Behera D, et al. Contribution of medical colleges to tuberculosis control in India under the Revised National Tuberculosis Control Programme (RNTCP): lessons learnt & challenges ahead. Indian J Med Res. 2013;137(2):283-294.

**Table 1 (abstract A49).** Delays in provision of TB care services, Internal Program Evaluation data, Revised National TB Control Program, 2014 (N=2240)

Particulars	Patient sided delay Mean (SD) [95%CI]	Diagnostic Delay Mean (SD) [95%CI]	Treatment delay Mean (SD) [95%CI]	Number of visits before diagnosis Median (Range)
Type of Provider				
Private	27(52)[23-31]	24(46)[21-27]	4(10)[3-4]	2 (1-6)
Public	29(45)[26-33]	11(30)[8-13]	5(18)[4-6]	1 (1-4)
RNTCP Zone				
North	21(39)[18-24]	15(35)[11-18]	6(14)[5-7]	2 (1-9)
South	20(32)[16-24]	14(22)[11-16]	5(4)[4-5]	3 (1-9)
West	24(38)[21-26]	14(30)[12-16]	6(16)[4-7]	2 (1-9)
East	31(69)[22-40]	11(47)[5-18]	5(8)[4-6]	2 (1-9)

TB: Tuberculosis; SD: Standard Deviation; CI: Confidence Interval

## A50 Evaluation of Hospital Management System in secondary care hospitals of Thiruvarur district, Tamil Nadu, 2015

### Prakash Venkatesan^1^, Prabhdeep Kaur^2^

#### ^1^Directorate of Public Health and Preventive Medicine, Tamil Nadu; ^2^ICMR- National Institute of Epidemiology, Chennai

##### **Correspondence:** Prakash Venkatesan (drprakash85@gmail.com)

**Introduction**

The paper-based health record maintenance in secondary care hospitals of Tamil Nadu was replaced by electronic record-keeping by the introduction of an IT-based Hospital Management System (HMS) from the year 2012 onwards. The HMS was part of the Tamil Nadu Health Systems Project for Health systems strengthening in the secondary care hospitals, funded by the World Bank. The government of Tamil Nadu implemented the project in a phased manner in 200+ secondary care hospitals across the state. Although the project was operationalised, there were challenges in the quality of data and functioning of HMS based on district and state-level reviews. We conducted a survey to evaluate the operationalization, maintenance, efficiency, and acceptance of HMS in Thiruvarur district, Tamil Nadu.

**Methods**

A cross-sectional study was done at eight secondary care hospitals of Thiruvarur district, including 166 HMS counters, from January 2015 to June 2015. The study population included all doctors available at the time of the survey in the health facilities. We collected data regarding the deployment of hardware, software, and training. We estimated the duration of the breakdown of HMS concerning hardware, connectivity, and power. In-depth interviews were done with selected users to understand the challenges in using HMS. The proportion was computed for various indicators related to operationalization, maintenance, efficiency, and completeness of the information. We computed the median duration of HMS breakdown time.

**Results**

*Operationalization:* The HMS was implemented in eight hospitals in the Thiruvarur district from 2012 onwards. HMS implementation had the following components: A centralized web-based application was developed (TCS), procurement of computers and other IT infrastructure, TNSWAN (Tamilnadu State Wide Area Network) for Primary connectivity, VPN (Virtual Private Network) from BSNL for secondary connectivity, and data backup at State Data Centre, Perungudi. The state-level team established a Helpdesk and Server administration group in Chennai.

Most (90%) of the HMS counter in the hospitals functioned in terms of hardware and connectivity (Table 1). 65% of HMS counters were used to capture patient details in hospitals on the day of the survey. The clinical OP module was the most useful in the HMS platform. In the previous six months, 84% of the outpatient visits were registered, 69% received an HMS-based prescription, 59% received drugs from pharmacists through HMS, and 94% of the lab investigations were entered in the system.

Of the 166 counters studied, only 2/3rd were utilized on any given day. Utilization was higher for ‘OP department counters’ and lower for ‘other department counters’. There was a progressive decrease in the capture of patient details in HMS at successive OP flow stages, beginning with registration and ending at the pharmacy.

*Maintenance:* Each HMS counter faced a breakdown for hardware issues at least once in six months, and it took four days on average to rectify it (Table 1). The median duration of connectivity loss was 23 minutes for primary and 32 minutes for secondary connectivity. The users lost nearly 10% of the time in OP hours due to connectivity breakdown. There was no systemic documentation of HMS breakdown issues at the hospital level. At least one breakdown occurred in every GH per day due to connectivity alone as monitored by state headquarters.

*Efficiency:* We estimated HMS efficiency in terms of time taken for an outpatient to get registered at the registration counter. Time taken was 18 seconds compared to 22 seconds in paper-based system. The utility of various data columns in doctors clinical module showed that no other fields were filled in most of the records except for treatment. The users appreciated ease, accuracy, and speed; however, slower connectivity was a major barrier. The other challenges were adverse doctor-patient relationships and lack of technical team support.

*Acceptance:* We surveyed 49 doctors to understand the perceptions regarding the use of HMS. Most of the doctors reported that HMS was easy, accurate, and reliable. Nearly 70% said that HMS replaces paper records and e-prescribing was fast. Almost half of all doctors felt that the doctor-patient relationship was affected due to time spent on HMS.

**Conclusion**

Operationalization of HMS was complete; however, maintenance of connectivity and timely technical support was inadequate for HMS. Users utilized only a few modules in the software. We recommend systemic documentation of breakdown issues of HMS at the hospital with feedback from users for a detailed evaluation of maintenance. The program managers should take the necessary steps to improve network connectivity at the hospitals. Clinical modules of doctors must be reviewed in terms of usefulness, and unnecessary fields need to be identified in various forms and removed.

**Table 1 (abstract A50).** Operationalization and maintenance of Hospital Management System (HMS) in Government Hospitals of Thiruvarur district, Tamil Nadu, 2015

Key Indicators	N	n	Proportion (%)
**HMS operationalization**			
Doctors using HMS in the district	61	61	100
HMS counters with functional CPU and Monitor	166	148	89
HMS counters with functioning printers	76	55	78
HMS counters with functioning Internet	166	138	83
HMS counters in the hospitals utilized to capture patient details on the day of the survey	166	107	65
HMS Counters in the 'OP Department' utilized to capture patient details on the day of the survey	113	82	73
HMS Counters in the hospital other than 'OP Department' utilized for HMS on the day of the survey	53	25	42
Outpatients registered in the HMS for 6-month period	753769	634674	84
Outpatients who received HMS based prescriptions	753769	522918	69
Outpatients for whom the pharmacists issued drugs through HMS	753769	445851	59
Outpatients for whom the Lab investigations results were entered in the HMS	334525	314675	94
**HMS maintenance**			
Days for which the IT coordinator was available in the field (GH) in past six months	135 days	40 days	29.6%
Occasions by which a HMS counter in the hospital is ‘down’ due to hardware problem in six month period	166	203	1.22 occasion / counter
‘HMS Downtime’ hours in the hospital due to ‘Primary connectivity failure’ during OP hours (slowness) in six-month period	9412 hrs	3128 hrs	33%
‘HMS Downtime’ hours in the hospital due to both ‘Primary and Secondary connectivity failure’ during OP hours	9412 hrs	744 hrs	8%
‘HMS Downtime’ hours in the hospital due to ‘Power failure’ during OP hours	9412 hrs	0	0
Mean days required to rectify a breakdown in a hospital HMS counter due to a hardware problem	93 occasions	408 days	4.4 days

## A51 Epidemiology of Leptospirosis infection in the state of Kerala, India during 2018-19

### Ajan Maheswaran Jaya^1^, Sarita Ragini Lohithakshan^1^, Meenakshi Vasu^1^, Parasuraman Ganeshkumar^2^

#### ^1^Directorate of Health Services, Kerala; ^2^ICMR National Institute of Epidemiology, Chennai, India

##### **Correspondence:** Parasuraman Ganeshkumar (ganeshkumardr@gmail.com)

**Introduction**

Leptospirosis is an infectious disease of public health importance with outbreak potential in developing countries [1]. In India, outbreaks of leptospirosis were reported in Kerala, Gujarat, Tamil Nadu, Karnataka, and sporadic cases have been reported in Goa, Andhra Pradesh, and Assam every year [2]. Kerala, one of the southern states in India, reports leptospirosis cases throughout the year. Even though leptospirosis is a common infection in India, prime importance is not given despite high mortality [3]. Leptospirosis accounts for about 12.7% of acute febrile illness cases responsible for attendance at hospitals [2]. The highest rates occur during October and November, with seroprevalence of up to 55% in the general population [4]. The disease is mainly seen among the manual labourers with sporadic cases, among others [5]. Interestingly, the predominance of leptospirosis correlates with the presence of semi-domestic brown rats, cattle and dogs [2]. The disease is more commonly associated with natural disasters, especially during the monsoon period. Leptospirosis has long been a major threat to the State of Kerala with more than 1,000 cases being reported annually. Nationally, it causes the highest number of deaths among all communicable diseases. However, the epidemiology of leptospirosis in Kerala was not understood completely. Hence, we did this study to describe the epidemiology of leptospirosis in Kerala.

**Methods**

We used data on surveillance of communicable diseases in Kerala to estimate leptospirosis incidence per hundred thousand population. With its network of institutions in Integrated disease surveillance program (IDSP), Kerala obtains data and reports of infectious diseases. At the district and state level, this data is collated, analysed and reports are prepared under IDSP. We collected the details of confirmed leptospirosis case-patients from IDSP data maintained at the State IDSP cell from January 2018 to December 2019. We estimated the projected population for 2018 and 2019 based on Census 2011. We used the standard case definition recommended by IDSP. We defined a confirmed case of leptospirosis as an individual who was tested and turned positive of IgM or Microscopic agglutination test for leptospiral antibodies. We plotted the number of case-patients by the date of reporting in Epicurve for the years 2018 and 2019 and mapped incidence by the district. We calculated the incidence by age-group and sex. We also calculated the case-fatality rate (CFR) using the formula (number of deaths due to leptospirosis/number of leptospirosis case-patients) x 100.

**Results**

Overall cumulative incidence per hundred thousand population for the year 2018 and 2019 was 6.2 and 3.6 respectively. Incidence was high during September for the year 2018 and during August for the year 2019 (Figure 1). Incidence was high among adults (> 60 age group) during both 2018 (8.2 per 100 thousand population) and 2019 (7.1 per 100 thousand population). Incidence was high among males in 2018 (8.6 per 100 thousand population) and 2019 (5 per 100 thousand population). Incidence was high among adult males, 21.8 and 13.2 in 2018 and 2019, respectively. Among the administrative districts, Incidence was high in Pathanamthitta (25.6 per 100 thousand population), Wayanad (13.4 per 100 thousand population) and Alappuzha (11.6 per 100 thousand population) in 2018. These districts were worst affected by floods during 2018. There were 156 deaths reported due to leptospirosis in Kerala between 2018 and 2019. CFR was 4.6% and 4.5% during 2018 and 2019 respectively. The deaths were reported more in the age group 20 - 60 years and more among males. But the CFR was 8.2 % and 7% among males and 5.7% and 8.4% among females of age group more than 60 in 2018 and 2019 respectively.

**Conclusion**

We observed that the incidence of leptospirosis was 1.7 times more during 2018 than 2019, which was probably due to heavy rainfall and floods across most of the administrative districts of Kerala. Administrative districts such as Pathanamthitta, Wayanad and Alappuzha reported high incidence compared to other districts. Adult males reported a high incidence of leptospirosis. Post-monsoon months August to September reported a high incidence of leptospirosis compared to other months. High incidence of leptospirosis during 2018 was probably due to unprecedented rainfall and heavy floods. The case fatality rate was higher in 2018 than in 2019, with a higher risk of fatality among adult males. This was probably due to higher exposure and higher mobility in this group of individuals during floods. We recommended public health preparedness such as surveillance, testing and doxycycline prophylaxis with focused health education. We also suggested exploring rodent control measures in the pre-monsoon period to reduce the risk of leptospirosis infection in Kerala.

**References**

1. Bharti AR, Nally JE, Ricaldi JN, Matthias MA, Diaz MM, Lovett MA, et al. Leptospirosis: a zoonotic disease of global importance. Lancet Infect Dis. 2003 Dec;3(12):757–71.

2. Basker P, Kannan P, Kolandaswamy KG. Study on the Prevalence of Leptospirosis among Fever Cases Reported from Private Clinics in the Urban areas of Villupuram District, Tamil Nadu, India. Osong Public Health Res Perspect. 2014 Feb;5(1):54–67.

3. Sehgal SC, Sugunan AP, Vijayachari P. Leptospirosis disease burden estimation and surveillance networking in India. Southeast Asian journal of tropical medicine and public health. 2003 Sep 25;34:170-7.

4. Victoriano AFB, Smythe LD, Gloriani-Barzaga N, Cavinta LL, Kasai T, Limpakarnjanarat K, et al. Leptospirosis in the Asia Pacific region. BMC Infect Dis. 2009 Dec;9(1):147.

5. Sharma S, Vijayachari P, Sugunan AP, Natarajaseenivasan K, Sehgal SC. Seroprevalence of leptospirosis among high-risk population of Andaman Islands, India. The American journal of tropical medicine and hygiene. 2006 Feb 1;74(2):278-83.

**Fig. 1 (abstract A51).**
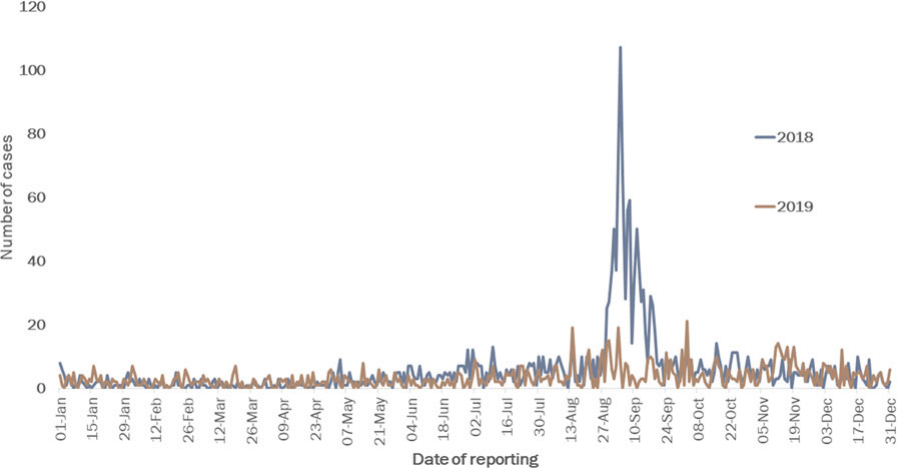
Distribution of Leptospirosis cases by date of reporting, Kerala, 2018 and 2019

## A52 Deaths due to noncommunicable diseases among the tribal population in Mokokchung, Nagaland, 2017

### Aonungdok Tushi Ao^1^, Prasanta Kumar Borah^2^, Nabajit Kumar Das^2^, Pankaj Uike^3^, Subongtemjen Sangtemkaba Longchar^1^, Prabhdeep Kaur^3^

#### ^1^Health & Family Welfare Department, Nagaland; ^2^ICMR-Regional Medical Research Center, Dibrugarh, Assam; ^3^ICMR-National Institute of Epidemiology, Chennai, Tamil Nadu

##### **Correspondence:** Aonungdok Tushi Ao (nungdok@yahoo.com)

**Introduction:**

Over 46 million of the estimated 60 million deaths worldwide occur in developing countries. Over three-quarters of deaths in India occur at home, and more than half of these deaths do not have a certified cause [1]. Mortality data helps prioritize the health problems and appropriately allocate health resources. A recent survey in Mokokchung documented a high prevalence of noncommunicable disease risk factors [2]. However, there was limited data regarding mortality patterns in the district. We estimated the proportion of deaths due to noncommunicable diseases (NCD) among people above 15 years in Mokokchung district, Nagaland, India. We also described all causes of deaths overall and by gender.

**Methods:**

We did a cross-sectional survey between April 2017 to October 2107 in the Mokokchung district of Nagaland. The study population included all deaths above 15 years of age during the previous year. As per the census 2011, 91.7% of the residents were scheduled tribes (mostly Ao tribe) with an average literacy rate of 91.6%. Nearly three fourth (71.4%) of the population lived in rural areas. We calculated the sample size assuming 30% anticipated frequency of deaths due to NCD; 5% absolute precision; 95% confidence interval; and a design effect of 1.4. The sample size was 452 participants. We used the cluster sampling method for selecting the 45 villages using probability proportional to size linear systematic sampling [PPSLSS] from the villages' list as per the census 2011. We obtained a list of all deaths above 15 years of age in the selected clusters. We used the Register General of India / Center for Global Health Research (RGI / CGHR) Verbal Autopsy (VA) tool for ascertaining the cause of deaths in the study [1].

We collected information about the sociodemographic characteristics, medical history of pre-existing diseases, treatment history, and risk behaviors. We also recorded the narrative history in the local language, which included illness or events leading to death. We classified the deaths as NCD, Infectious Diseases, and Others using ICD-10 classification. NCD included the following codes-Circulatory system (100-199), Neoplasm (C00-D49), Chronic respiratory diseases (J00-J99), Digestive system diseases (K00-K93), Kidney diseases (N00-N99), Type 1 / Type 2 Diabetes Mellitus (E00-E90), Nervous system diseases (G0-G99), and Mental, behavioral disorder F00-F99). Infectious diseases (A00-B99) included the following codes - Major conditions were Malaria (B54), Tuberculosis (A15.0), Infectious gastroenteritis (A09), and Acute viral Hepatitis (B19.9). Others included - External causes (injuries/suicides) (S00-Y98), Diseases that are not elsewhere classifiable (R00-R99), including age-related senility (R54), Abdominal pain unspecified (R10.9), Unspecified fever (R50.9), and unknown etiology (R99).

The cause of death & ICD-10 codes were assigned independently by two medical doctors. We used epi Info 3.5.3 to compute proportions with 95% confidence interval (CI) for categorical variables. We estimated the overall and proportion of deaths in each category by gender. We obtained approval from the Institutional Ethics Committee of Regional Medical Research Center, Dibrugarh, and National Institute of Epidemiology, Chennai. We obtained an informed consent obtained from participants.

**Results**

We surveyed 527 deaths above 15 years of age. Among the 527 deceased, 65% of the were above the age of 60, 23% never attended school, 72% were BPL cardholders, 81% lived in kachcha / semi kachcha houses, and 99% belonged to the scheduled tribe. The home was the most common place of death (67%). Alcohol use was prevalent among 23% and smoking among 31% of the deceased in the five years before death, with the proportion being higher among males than females. Hypertension was the most common pre-existing disease (46%), followed by stroke (28%), heart disease (24%), and chronic respiratory disease/asthma (21%).

Overall, NCD accounted for 79% of deaths, followed by other causes (13%) and infectious diseases (8%). Three-fourths of the deaths among smokers (76%) and alcohol users (73%) were noncommunicable diseases. Cerebrovascular diseases (31%) were the leading cause of death, followed by neoplasms (13%) and infectious diseases. Among infectious diseases, tuberculosis caused 5% of the deaths. Kidney diseases accounted for 7%, and digestive system diseases accounted for 7% of deaths. Injuries or accidents (external causes) were responsible for 6% of deaths. Respiratory system diseases accounted for 4% of death. The proportion of deaths due to circulatory system diseases was significantly higher among females than males (<0.05) (Figure 1). In contrast, the proportion of deaths due to neoplasm, respiratory system, kidney disease, and external causes was higher among males than females. The overall percentage of deaths due to digestive system diseases was significantly higher among men than women (<0.05)**.**

**Conclusion**

NCD was the leading cause of death in the study population, with cardiovascular diseases being the leading cause of death and hypertension was the commonest pre-existing disease. Noncommunicable diseases should be the foremost health priority in the Mokokchung district. We should initiate intersectoral interventions targeting tobacco and alcohol use. As there is no cancer hospital in the district, a referral system for early detection and cancer referral needs to be developed. We must strengthen primary Health care facilities to provide screening and regular treatment for hypertension to prevent mortality due to complications. Secondary Health care facilities should have the infrastructure and resources for managing acute coronary syndromes and strokes. Health care providers can be trained to provide treatment and counseling for smoking cessation and alcohol use disorder.

**Acknowledgement**

We would like to thank the Health & Family Welfare Department, Nagaland and the Chief Medical Officer, Mokokchung for granting us the permission to conduct study in Mokokchung district of Nagaland.

**References**

1. Jha P, Gajalakshmi V, Gupta PC, Kumar R, Mony P, Dhingra N, Peto R, RGI-CGHR Prospective Study Collaborators. Prospective study of one million deaths in India: rationale, design, and validation results. PLoS Med. 2005 Dec 20;3(2):e18.

2. Tushi A, Rao SR, Pattabi K, Kaur P. Prevalence of risk factors for noncommunicable diseases in a rural tribal population of Mokokchung, Nagaland, India. The National medical journal of India. 2018 Jan 1;31(1):11.

**Fig. 1 (abstract A52).**
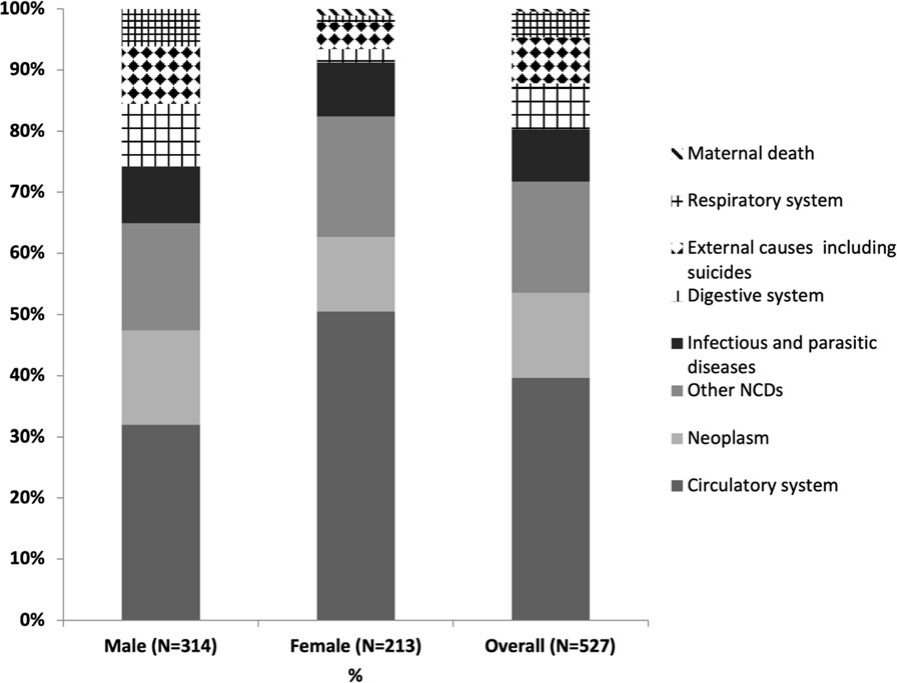
Cause of death overall and by gender in Mokokchung, Nagaland, 2017.

## A53 Review of hypertension management services in Primary Public Health facilities in Puducherry district, 2019

### Lakshmanasamy Ravivarman, Prabhdeep Kaur

#### ICMR-National Institute of Epidemiology, Chennai, India

##### **Correspondence:** Lakshmanasamy Ravivarman (drravivarman@gmail.com)

**Background**

The burden of Noncommunicable diseases is very high with 70% of the global deaths due to it.(1) Among them, the deaths in premature age group in considerably high. The leading global metabolic risk factor is elevated blood pressure, attributed to 19% of global deaths. Premature mortality due to heart diseases is 40% in India.(2)

Puducherry is a highly urbanised territory in Southern India with robust health infrastructure. Prevalence of hypertension is the highest in Puducherry among the states/UTs in India. A recent study recorded the prevalence of hypertension as 40%, with untreated as 36% and uncontrolled as 38% in a specific age group.(3) National Programme for Prevention and Control of Cancer, Diabetes, Cardiovascular diseases and Stroke (NPCDCS) is implemented by Government of India, addressing the screening and treatment aspects of the hypertension. We did a study with the objective to describe the hypertension component of NPCDCS programme in Puducherry District and to evaluate the effectiveness of opportunistic screening and treatment in achieving hypertension control in primary public health facilities of Puducherry District, India, 2019.

**Methods**

We did a cross sectional study. We visited all the public primary health facilities in Puducherry district and observed the operations with respect to hypertension services management. We did the study between June 2019 and August 2019, with a reference period of the January to December 2018). Ten public health facilities were selected by probability proportional to size (Hypertensive persons of November 2018) systematic sampling (PPSS). To calculate the number of hypertensive patients to be studied, we considered 75% blood pressure control(4) with 10% relative precision, design effect of 2, for 95% confidence interval and 20% non-response rate. Thereby we studied thirty-two hypertensive patients by consecutive sampling in each of the ten selected public health facilities.

We described the Programme component as per the NPCDCS manual. We designed a logic model (Table 1) with input, process and output indicators to study the effectiveness of the programme. Structured questionnaires were used to collect data from the medical officers, staff nurse, and pharmacists about the facilities, trainings and resources. Further, to assess the hypertension control ratio at facility level, we interviewed the registered hypertensive patients attending the facilities and recorded their blood pressure using standardised blood pressure apparatus. For this purpose, we did a two-stage cluster sampling. We calculated Input, process and output indicators as proportions. Data was analysed using epi-info software version 7.5.3

**Results**

NPCDCS was started in Puducherry district in 2014-15, by which all adults above 30 years of age are screened for specific non communicable diseases. Opportunistic screening was done in all the health facilities, where adults above 30 years of age visiting the health facilities for routine health care will be screened for the non-communicable diseases. This is operational on two days a week (Tuesdays and Fridays). Population based screening, by which the entire population above 30 years of age will be screened at community level for NCDs was implemented as a pilot project in four health facilities.

All health facilities have staff nurse (non-designated) for NCD operations and basic equipment like BP apparatus and weighing scale were available. Screening protocol was displayed (81%), treatment protocol not available, whereas IEC awareness displays (89%) done. There was shortage of one antihypertensive drug for four months, due to supply constraints. All patients receive drugs for one month duration and all are counselled for healthy life style by staff nurse.

Almost 94% of the hypertension patients took the prescribed drugs regularly in the previous 1 month and 98% with self-report salt reduction. Among the screened patients, 38% had high blood pressure. Blood pressure control across health facilities was 51%.

On nurse’s performance assessment, height and weight were recorded in 44% of the patients records and Body Mass Index (BMI) in 32% patients. Screening register was maintained by 89% of staff nurse at the health facilities. Around 63% doctors mentioned correctly the cut off value to diagnose hypertension and 81% doctors mentioned calcium channel blockers as first drug of choice.

Half of the hypertensive patients included in the study were above sixty years age (54%), 61% were female, 45% had no formal school education and 70% were unemployed. Regarding drug regimen, 43% were on single drug, 42% on dual drug and 15% patients were on more than two drugs

**Conclusion**

NPCDCS is a programme designed at the National level and being implemented at field. The programme addresses the at-risk age group population. Clear and defined roles are assigned to different cadres of health workers. The programme also provides a uniform screening and treatment protocols to be followed. At implementation level, necessary infrastructure, equipment, trainings, manpower and other resources have to be ensured. Adherence to protocol by health care providers and treatment adherence by patients should be ensured.

Also, factors influencing blood pressure control rates may be identified and periodic monitoring of blood pressure control ratios should be monitored. More realistic picture may be obtained by performing community level hypertension surveys.

**References**

1. Non communicable diseases [Internet]. [cited 2021 Feb 22]. Available from: https://www.who.int/news-room/fact-sheets/detail/noncommunicable-diseases

2. Hay S, Suliankatchi R, Afshin A, Agarwal S, Aggarwal A, Aggarwal R, et al. India: Health of the Nation’s States - The India State-level Disease Burden Initiative. 2017.

3. Prenissl J, Manne-Goehler J, Jaacks LM, Prabhakaran D, Awasthi A, Bischops AC, et al. Hypertension screening, awareness, treatment, and control in India: A nationally representative cross-sectional study among individuals aged 15 to 49 years. PLoS Med. 2019 May;16(5):e1002801.

4. Nagappa B, Thekkur P, Majella MG, Nair D, Ramaswamy G, Chinnakali P. Failure to achieve goal blood pressure and its associated factors among hypertensive patients registered in a primary health centre in South India. J Fam Med Prim Care. 2018;7(1):81–6.

**Table 1 (abstract A53).** Logic Framework model to evaluate the effectiveness of hypertension component of the NPCDCS in Puducherry district, 2019

Logic Model of the Programme		
Framework	Opportunistic Screening	Treatment & Counselling
Input	Human Resources, Training manual, BP apparatus, weighing scale, stadiometer, protocol for diagnosis	Hypertensive Drugs, Funds, Protocol for treatment, IEC materials
Process	Training for NCD nurses & medical officers Hypertension screening as per protocol	Prescribing drugs as per protocol, Counselling for long term treatment and life style modification
Output	Hypertension cases screened Hypertension cases detected	Patients adhering to taking drugs for 1 month, Patients following lifestyle modification measures
Outcome	Hypertension control among patients screened and treated at the facilities	

## A54 Diarrhoeal disease, Water and Sanitation facility in rural Dharmapuri district, Tamil Nadu, India, 2011 – 2014

### Nallathambi Yogananth^1^, Tarun Bhatnagar^2^

#### ^1^Directorate of Public Health and Preventive Medicine, Chennai, India; ^2^ICMR School of Public Health, ICMR-National Institute of Epidemiology, Chennai, India

##### **Correspondence:** Nallathambi Yogananth (dryogananth@gmail.com)

**Background**

Provision of improved water supply, sanitation services, and hygiene behaviour, supported by an enabling environment, will enhance child survival and development [1]. In Dharmapuri district, Acute Diarrhoeal Disease Control Programme aims to decrease the incidence of diarrhoea. The National Rural Drinking Water Programme, Hogenakkal Water Supply and Fluorosis Mitigation Project and Village Health Water Sanitation and Nutrition Committee [2] improved the quality and quantity of water supply. Improved sanitation facilities are provided by Nirmal Bharat Abhiyan and Self Sufficiency Scheme [3]. In spite, Census 2011 reported open defecation practice in 88.2% of households of rural Dharmapuri district. About 52% of the rural and 73% of the urban households had sustainable access to an improved water source [4]. Only 12% of the rural and 65% of the urban households had access to improved sanitation facility [4]. In 2010, 88% of drinking water sources tested were bacteriologically contaminated, and 28% with faecal coliforms(1). Our objective was to identify correlations between the incidence of acute diarrheal disease (ADD), unimproved sanitation, water facility, bacteriological contamination of water and open defecation practice in rural Dharmapuri district.

**Methods**

A cross-sectional study across eight blocks with Integrated Disease Surveillance Programme and National Rural Drinking Water Programme data from 2011 to 2014 was conducted to calculate block wise diarrhoea incidence and water contamination. For improved water and sanitation facilities, census 2011 data was used. We adopted the operational definition for unimproved sanitation and unimproved water source from the World Health Organisation and UNICEF manual on Water and Sanitation. We drew Epi maps for Incidence rates and proportions across eight blocks. We conducted an ecological analysis and the estimated correlation coefficient and coefficient of determination to assess their relationship. For ecological analysis, we selected the block as the unit of analysis. We used Excel and SPSS version 22 for analysis.

**Results**

The decreasing trend of ADD incidence was found in all eight Dharmapuri district blocks from 2011-2014. The highest incidence of ADD was observed in Pappireddipatti block during 2011, 2012 and 2014. In 2013, it was Karimangalam block. Similarly, Bacteriological contamination across the water sources decreased all the eight blocks from 2011 – 2014. Bacteriological contamination was high in Pappireddipatti, Palacode and Harur blocks during 2011, 2012 and 2013. No water sources tested were bacteriologically contaminated in 2014. Pappireddipatti block recorded the maximum reduction in both indicators. Harur, Dharmapuri blocks had the highest and lowest proportion of households with unimproved water sources, respectively. Concerning unimproved sanitation, Pennagaram and Dharmapuri blocks had the highest and lowest proportion of households, respectively. No significant correlation was observed between ADD incidence and sanitation, water bacteriological contamination and open defecation in 2011. In the National Rural Drinking Water Project, the Town panchayat water sources were not tested. We have observed certain discrepancies in the house listing and housing data of 2011 between revenue village level and block level.

**Conclusion**

We found a decreasing trend in ADD incidence and bacteriological contamination of water from 2011 to 2014. About four-fifths of households had unimproved sanitation, and half had unimproved water. No correlation was observed between ADD incidence and households with unimproved sanitation and water facility, bacteriological contamination. No significant correlation was observed between bacteriological contamination and open defecation. We recommend identifying the reasons for poor water and sanitation facilities at the community and programme level. We recommend improving water and sanitation facilities and studying the association between diarrhoea, water and sanitation at the individual level.

**Reference**

1. sanitation and Programming Guidance [Internet]. Water Supply and Sanitation Collaborative Council and the World Health Organization. Geneva; 2005. Available from: https://www.who.int/water_sanitation_health/hygiene/sanhygpromo.pdf. Accessed 15 February 2021.

2. TWAD Board. Tamil Nadu Water Supply and Drainage Board. Available from: https://www.twadboard.tn.gov.in/. Accessed 15 February 2021.

3. Guidelines Nirmal Bharath Abhiyan. Ministry of Drinking Water and Sanitation. Government of India. Available from: http://mdm.nic.in/mdm_website/Files/WASH/Final%20Guidelines%20(English).pdf. Accessed 15 February 2021.

4. District census Handbook Dharmapuri. Census of India 2011. Available from: https://censusindia.gov.in/2011census/dchb/3329_PART_B_DCHB_DHARMAPURI.pdf. Accessed 15 February 2021.

## A55 Public health budget analysis of India- A measure to attain NCD sustainable developmental goal

### Ashish Krishna^1^, Anupam Khungar Pathni^1^, Bhawna Sharma^1^, Parasuraman Ganeshkumar^2^, Savitha Kasiviswanathan^2^

#### ^1^Resolve to Save Lives; ^2^ ICMR – National Institute of Epidemiology, Chennai, Tamil Nadu, India

##### **Correspondence:** Ashish Krishna (akrishna@resolvetosavelives.org)

**Background**

Non-communicable diseases (NCDs) are also known as chronic diseases because they tend to be long and sometimes even a lifetime for patients. The main types of NCDs are cardiovascular diseases (like heart attacks and stroke), cancers, chronic respiratory diseases (such as chronic obstructive pulmonary disease and asthma), and diabetes [1]. The Low-middle income countries account for approximately half (7.2 million) of the 15 million premature deaths in 30 to 70 years due to non-communicable diseases (NCDs). Despite the considerable burden of NCDs, the global financing for them is highly insufficient, receiving only 2% of overall health funding. WHO's best buy policy states that in low middle-income countries, for every US$1 (76 INR) spends on scaling up the interventions on NCDs on this strategy will give at least US$7 (533 INR) return in terms of longer life, increased productivity and employment. As an effective measure to control the burden of NCDs in a low middle-income country, WHO recommends an expenditure of US$1.27 (83 INR) per person per year. The impact of this investment will be huge as it will save 8.2 million lives by 2030 and create US$350 billion through an increase in productivity and reduction in health costs. If all countries conjointly implement this policy, the world can quickly achieve Sustainable Development Goal (SDG) 3.4 of reduction in premature deaths from NCDs by one-third by 2030 [2]. In India, states receive funds in state PIPs(Program implementation plan) under NCD Flexi-pool. The proportion of center & state share ratio is 60:40, except for northern-eastern states where this ratio is 90:10. In this study, we attempted to understand the budget deficit for non-communicable disease programs in different states of India for the financial year of 2016-2017. We will also further investigate in detail what proportion of approved NCD budget under the National Health Mission was utilized for implementation of the programs in the same financial year [3].

**Methods**

We extracted health budget data from the heath government website available in the public domain for the financial year of 2016-2017. As per the national health mission guidelines, the center allocated 60% of the NCD program budget to states, except for the north-eastern state, contributing 90% of the share. The details of center contribution are available in detail in the recording of proceedings (ROPs), but there was no public document available about state contribution to NCDs. But as per National Health Mission (NHM) guidelines, the total public health budget for NCD was extrapolated, adding 40% contribution from most states and 10% from north-eastern states. After that, the total approved budget allocated for NCD programs was analyzed per capita for every state. Since the state-specific health budget expenditure was not available in the public domain, we calculated the proportion of left out NCD budget against approved NHM budget for NCD programs was in-depth reviewed for every state.

**Result**

The median estimated per capita expenditure of the approved public health budget for NCD programs in 2016- 2017 for all states was 0.3 $( 23 INR). Only seven states in India, like Uttarakhand, Nagaland, Goa, Sikkim, Manipur, Arunachal Pradesh, and Mizoram, have planned an adequate NCD budget per capita 1.05$ (above 80 INR). These states meet the norms of WHO's "best buy policy". We observed 70% of the states have inadequately planned their budget with less than half a dollar (below 35 INR) per capita for their NCD programs. Adequate budget expenditure indicates the quality and operational efficiency of health programs. We also calculated the actual expenditure of the approved NHM budget for the NCD program for different states. This expenditure analysis of different states shows that an average of 39% of the NCD budget remained unutilized. Almost twenty states were utilizing less than fifty percent of the total approved budget under NHM. The aggregated data of the whole utilized budget was further analyzed under the key budget heads as per NHM budget documents' financial framework. The maximum unutilized budget was shown under training, untied funds, community interventions & infrastructure development, innovation, and IEC components.

**Conclusion**

India is less dependent on donor funding for most of its health programs. Research evidence illustrates that India does not have enough resources for the management of NCD programs at the primary care level. In the absence of these basic healthcare facilities, the burden on out-of-pocket expenditures will rise significantly because of the costly treatment of NCD-related illnesses and their chronic nature. Existing health programs are not enough to reduce the burden of NCDs. The political determination coupled with adequate financing to support the NCD program is mandatory to meet SDG 3.4 goal.

**References**

1. Non-communicable diseases Key facts. World Health Organization. 2018. https://www.who.int/news-room/fact-sheets/detail/noncommunicable-diseases Accessed 12 Jan 2020.

2. 'Best Buys' and other recommended interventions for the Prevention and Control of Noncommunicable Diseases. 2017. https://www.who.int/ncds/management/WHO_Appendix_BestBuys.pdf. Accessed 13 Jan 2020.

3. 'Best Buys' and other recommended interventions for the Prevention and Control of Noncommunicable Diseases. 2017. https://www.who.int/ncds/management/WHO_Appendix_BestBuys.pdf. Accessed 13 Jan 2020.

**Fig. 1 (abstract A55).**
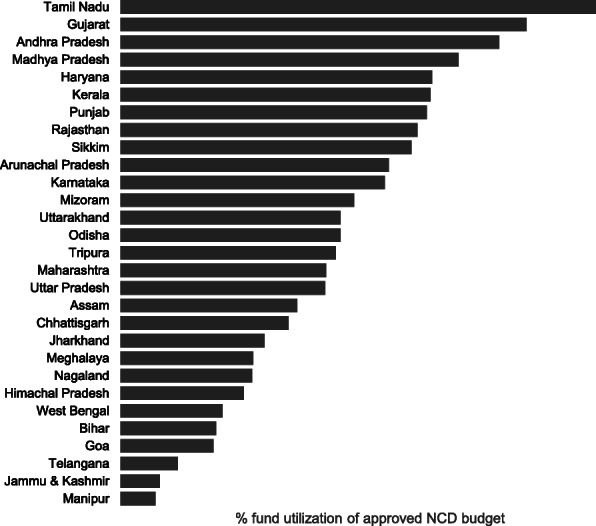
Utilization of approved NCD budget under NHM in 2016-2017

## A56 A critical discourse analysis of outbreak investigation process: A study on outbreak investigation reports of Field Epidemiology Training Programme (FETP) Scholars, ICMR School of Public Health, India, 2019

### Nuzrath Jahan^1^_,_ Harshal Sonekar^1^_,_ Manickam Ponnaiah^1^_,_ Mathew George^2^

#### ^1^ICMR School of Public Health, ICMR-National Institute of Epidemiology, Chennai, Tamilnadu, India; ^2^Centre for Public Health, School of Health Systems Studies-Tata Institute of Social sciences, Mumbai, India

##### **Correspondence:** Nuzrath Jahan (dr.nuz23@gmail.com)

**Background**

Any outbreak investigation is an act of conducting a systematic inquiry of an epidemic or a public health problem as it affects a population. The purpose is to examine and understand the nature, context and cause of an outbreak to launch effective control measures and serve as valuable feedback to prevent future outbreaks in similar sociocultural contexts. India FETP by ICMR school of public health has been training public health officers in outbreak investigations. As the role of outbreak investigation carried out by scholars acquires a prominent place in the overall curriculum and is guided by faculty of public health institutions across India, these studies can represent the outbreak investigations carried out in the country. Outbreak reports are not mere documentation of facts and observations. They are produced through a synthesis of multi-level actions and mediations from confirmation of diagnosis and outbreak, additional case search, environmental assessment followed by communicating the findings to the community and authorities concerned, culminating in a final study report. These processes involve many actors and agents, who carry their own beliefs and motivations to do something, which influences the drafting, reviewing and publishing the evidence and suggestions in the report. In sociological terms, an analysis of the outbreak investigation reports of students of Public health will help understand the discourse surrounding the specific outbreak investigations and more importantly, the processes adopted by the society. Discourse is a patterned way of thinking that can be understood through the manifestation of power, legitimacy, and social structures analysed through texts, talks, and symbols in day-to-day social interactions [1]. Discourse analysis is a framework used to analyse the outbreak investigation reports of FETP scholars to understand how outbreaks and the evidence for causality are presented, and how the agencies of communities and power, domination, legitimacy are contextualised.

**Methods**

We used Critical discourse analysis [2], to delve into ten outbreak investigation reports submitted by India FETP scholars during 2008-2018. During this period, 171 outbreak investigations had been conducted, of which detailed manuscripts were available only for 44 outbreak investigations. Initially, we selected five reports purposively for the current study, and added another five reports to achieve “information power”. To optimise relevance, we chose reports from the most recent years. As part of inter discursive analysis, we analysed each report in detail with constant comparison to identify discourses, wherein the texts were considered at three levels: 1) Inter-textuality and genre 2) semantics and meaning 3) Representation of social events and discourses. We looked for actions, representations and identifications, and how these were realised in the text by using specific lexicons, grammar, sentence structures and clauses situated within the study context.

**Results**

There is a clear generic structure to the reports as they stick to the 10-point steps [3], with less variability. They begin with reporting disturbances in normality and wrap up by the partial or complete rectification of the disturbances. Thus, there is a rigid staging to the reports. The sequence of elements is usually topical, starting from global to the local context. The rigid structuring shows substantial inequalities of power and distance among the actors involved: the authors, the investigators and the study participants, which risks potential problems of legitimacy and alienation. The report's format is multimodal as they combine different semiotic modalities like graphs, maps, and tools like questionnaires used during investigations. The genre transfers from the narrative genre in the background, methods, and results section to the discussion section's argumentative. These genre chains link the different scales of discourses: global, national to local, majority of which refers to institutions' documents in whom the authority is vested. This rationalisation process by reference to institutionalised prescriptive actions and suggestions seeks to ensure or maximise community acceptability for the same. Indirect intertextuality is applied while quoting references from other texts or documents of the same or different scale as a means to relate or contradict context and to validate the assumptions that delve below the surface of the texts. Semantically speaking, we have an elaborative pattern (listing of variables understudy), additive (and, also) and purposive clauses. The sentences in the discussion and conclusion sections have a problem-solution structure. Sentences in the limitation section have a contrastive/concessive structure. Grammatically, passivated sentences are employed as frequently as active sentences. Subjects or authors (E.g. we) disappear and reappear strategically throughout the reports. This serves a pattern to portray the authors as facilitators of transparent and democratic decision-makers, while the reality may not be so.

**Conclusion**

Outbreak causality and evidence for it are presented in specific ways within the outbreak reports shaped by the authors' ideologies and stances. Though these are academic reports, they are given and discussed among audiences directly or indirectly involved in the decision-making process. They can be influenced to balance the power and hierarchy between government agencies and the community. A balanced report will also help us understand how different courses of action or intervention might be valuable. Hence, a less rigidly staged report that acknowledges decision-makers' agencies and includes the voices of the individuals/ actors could be more successful in achieving its purpose. Additionally, how the setting/ context in which the events occur to influence actors' health behaviour needs special attention as it is the context of life and work that decides the opportunities/ capability for people to adopt healthy behaviours.

We hope the study will serve as valuable feedback to the current and future public health researchers while investigating the outbreak and help comprehend the community and health system within its functional context.

**References**

[1] Discourse analysis: a new methodology for understanding health and illness ideologies - Lupton - 1992 - Australian Journal of Public Health - Wiley Online Library. Available on https://onlinelibrary.wiley.com/doi/abs/10.1111/j.1753-6405.1992.tb00043.x (accessed Jun. 21, 2020).

[2] Analysing Discourse: Textual Analysis for Social Research, *CRC Press*. Available on https://www.routledge.com/Analysing-Discourse-Textual-Analysis-for-Social-Research/Fairclough/p/book/9780415258937 (accessed Jun. 21, 2020).

[3] Principles of Epidemiology: Lesson 6, Section 2|Self-Study Course SS1978|CDC. Available on https://www.cdc.gov/csels/dsepd/ss1978/lesson6/section2.html (accessed Jun. 21, 2020).

**Table 1 (abstract A56).** Identified elements of CDA with representative texts

Elements	Representative texts
Genres	Narrative: Mid-March 2018, we noted reports of blurred vision among school event attendees in a village in South India
	Argumentative: *Claim*-Contamination of drinking water could be the source of the outbreak. *Warrant*- Such contaminated source has been implicated to be the source of E. coli in many diarrheal outbreaks in India and elsewhere in the world. *Premises-* The floods following the cyclone may be a reason for the contamination of drinking water.
Scales of discourse	Global: Globally, an estimated 11-20 million people become ill from typhoid and between 128-161,000 people die from it annually.
	National: Typhoid fever is endemic in many parts of India with an estimated incidence of 377 (178-801) per 100,000 person-years
	Local: In May 2018, the microbiology department of KAP Viswanatham medical college of Trichy, Tamil Nadu detected and reported a cluster of four isolates of S. Typhi resistant to Ceftriaxone.
Indirect intertextuality	"It has been estimated in a study that 27,486,636 DAL YS will be lost in the year 2016 in India due to diarrheal diseases."
Clauses	Elaborative: “...about common exposures to food, water or place (travel history, mass gathering, social activity)”.
	Temporal: "Subsequently, they devised an operational case definition to diagnose cases individually."
	Additive: “They complained of overcrowding *and* lack of cleanliness during their stay; they *also* mentioned the practice of drinking raw water daily."
Categorical assertion	“It is *projected that* by 2050, the number will increase to 10 million deaths per year.”
Modalised assertions	“Our report *may b*e the first report of Photokeratitis due to the indoor exposure to unshielded mercury vapour and metal halide lights from South India.”
Passivated sentence	“Overall cleanliness *was graded* as good when there was no litter.”
The omission of subjects/actors	“It is of importance *to educate food handlers”.* “The indoor school *event occurred on*..”
Strategic disappearance and appearance of authors	“Firstly*, the bias in selection could have led to an underestimation* of the attack rate. However, it is less likely that *we* would have under-estimated the incidence.”
Nominalisation	“Environmental investigation indicated that the water of the wells used at the ice factory and the hotel were positive for non-faecal contamination”. "This new public health problem has repercussions for the health system."
Problem-solution structure	“Drinking contaminated water from borewell was associated with the diarrhoeal disease. Repairing the water pipelines led to the control of the outbreak”.
Contrastive/concessive structure	"We could have had information bias for the ascertainment of exposure as well as the outcome. However, despite such bias, the magnitude of the association was beyond chance.”

## A57 Cholera Outbreak Investigation in Sangli District, Maharashtra, India, 2019

### Purvi Patel^1^, Mohamed Azarudeen^1^, Sushma Choudhary^2^, Suneet Kaur^1^, Milind Pore^3^, Tanzin Dikid^1^, Sudhir Kumar Jain^1^, Sujeet Singh^1^

#### ^1^National Centre for Disease Control, Delhi; ^2^South Asia Field Epidemiology and Technology Network; ^3^District surveillance unit, Sangli

##### **Correspondence:** Purvi Patel (eis6ppatel@gmail.com)

**Introduction**

In India, 0.6million cholera cases occur annually [1]. There are 13 endemic states and 78 cholera hotspots districts [2]. Contamination of drinking water, poor sanitation and flooding are among known risk factors for cholera outbreaks [3]. Maharashtra is an endemic state and had recent cholera outbreaks in 2016 and 2018 [4]. In October 2019, Sangli, Maharashtra, reported sudden rise in acute watery diarrhea (AWD) cases from Shivajinagar village to the central unit, Integrated Disease Surveillance Programme. Sangli, a cholera hotspot district, was declared open defecation free in 2017 under country-wide Swachh Bharat Mission (SBM) [5]. Shivajinagar had population of 2,563 and was served by sub-centre, Vihapur (3km), primary health centre (PHC) Nevari (30km) and rural hospital (RH) Kadegaon (2km). Epidemic Intelligence Service officers from National Centre for Disease Control were deployed to conduct an epidemiological investigation of the outbreak. We present time, place and person distribution of cases, associated risk factors and recommendations for outbreak control and prevention.

**Methods**

For case search, suspect cholera was defined as ≥3 watery stools in 24hr duration (AWD) in a Shivajinagar resident (age ≥2year) from October 12 to November 8, 2019. Confirmed cholera was culture-positive stool of suspect case for *V. cholerae.* A line-list of cases was prepared through house-to-house search and from RH in-patient records on symptoms, treatment and toilet availability. We conducted an unmatched case-control study (1:1). We calculated sample size 80 assuming odds ratio (OR) 4, 40% exposure in controls, 95% confidence interval (CI) and 80% power. Cases were selected randomly from the line-list. Controls were Shivajinagar residents (age ≥2year) without AWD selected randomly from case-free households using village household registers. Data on socio-demography, exposures (suspected AWD case, mass gathering events), treatment-seeking, household sanitation, and hygiene practices was collected from all participants with Epicollect5 software and analysed using STATAv.15. We conducted descriptive statistics and applied chi-square, OR, and logistic regression for confounding and inference. Stool samples were collected on October 23-24 and sent for culture, sensitivity and serotyping at government medical college, Miraj. Public and private wells were tested for faecal contamination on October 23 and 30, and residual chlorine on November 8. A common well, main village water source, was surveyed and water supply maintenance records were sought for September-October, 2019.

**Results**

We identified 131 cholera cases (96% suspect, 4% confirmed) with median age 40years (range:2-86), 76 (56%) females and 5% (131/2,563) attack rate. Ninety-eight (75%) were hospitalized and case fatality was 2.3% (3/131). Cases were reported between October 13 and November 1, 2019 with a peak (30 cases) on 23 October. Among 40 cases interviewed, symptoms reported were abdominal pain (56%), vomiting (51%) and fever (23%). Twenty-one (53%) cases sought treatment on the day of illness. Primarily, 18 (43%) cases were treated at a village health camp while 12 (33%) and 8 (20%) were treated at RH and nearby private facilities, respectively. Subsequently, twenty-seven cases visited another health facility–12 (44%) private, 11 (41%) RH and 4 (15%) visited the health camp. Notably, no patient visited PHC Nevari reportedly due to distance. Thirty-seven (93%) cases received antibiotics and 36 (90%) received rehydration therapy.

Cases (40) and controls (40) were similar demographically except for education (<5^th^ standard, *p* value=0.02) and occupation (farmer and labourer, *p* value=0.02). Greater odds of AWD were associated with drinking water supplied from the common well (OR:47.6, 95% CI:6.4-2015.6). These odds grew (OR:69.5, 95% CI:7.13-658) after adjusting for age, education and gender. On subset analysis, odds of receiving drinking water through old pipeline system compared with others (new pipeline and private sources) were 16 times higher among cases compared to controls (OR=16.33, 95%CI=4.68-64).

Exposure to common food or water source during a local mass gathering event (assembly election on October 21) was found not significant. Among case-control study participants, handwashing practices with soap and water before cooking (OR=2.6, 95%CI=0.54-16.75), household water treatment (OR=1.3 95%CI=0.42-4.12) and safe drinking water retrieval practices (OR=4.6, 95%CI=0.41-234) were reported low. Additionally, among 14 households that treated drinking water, process of chlorination 7 (50%) and boiling 2 (14%) were found inadequate for water purification. Of 80, seven households (9%) did not own a toilet (OR=1.4, 95%CI=0.21-9.97) and used other’s or in one case practiced open defecation. And, 44 (56%) households did not have soap in handwashing area (OR=1.3, 95%CI=0.48-3.48) when surveyed.

Five of nine stool samples cultured positive for *V. cholerae*, El Tor O1, Ogawa. The common well water was found contaminated (>16 Coliforms/100ml) on October 23 and potable on October 30. Three private wells were found potable both times. The common well was found uncovered and situated on sloping terrain. Waste water drainage from upstream communities flowed within 1meter of it and drained downhill into a stream. This area was reportedly waterlogged due to heavy rains on October 18-20, 2019. Routinely, well water was pumped into overhead water tanks, chlorinated manually and distributed in the village through old and new pipeline systems established in 1982 and 2018, respectively. Routine chlorination was reported irregular however, records of it and other maintenance activities were not available.

**Conclusion**

Shivajinagar village in Sangli, Maharashtra witnessed laboratory confirmed outbreak of El Tor *V. cholerae* (Ogawa) likely due to supply of contaminated drinking water. Local authorities-initiated control measures on October 22 after two deaths. It included arranging alternate water supply following stoppage of the regular one, a health camp, active case search and health promotion activities. We report limitations due to recall bias in participants, absent water testing for cholera, and possible inclusion of baseline diarrhea cases as only nine cases were tested for cholera.

We recommended health education on handwashing, household water treatment, safe storage, and promoting temporary latrines as short-term control measures. In long term, we recommended to ensure routine water chlorination, testing and their record keeping, to continue health education, and to construct toilets under SBM after needs assessment. Replacement of old pipeline and improvement of common well surroundings were advised. Better coordination between serving government health facilities was recommended for early detection of case surge considering low utilization of PHC.

**References**

1. Ali M, Nelson AR, Lopez AL, Sack DA. Updated global burden of cholera in endemic countries. PLoS Negl Trop Dis. 2015;9(6):1–13.

2. Ali M, Gupta S Sen, Arora N, Khasnobis P, Venkatesh S, Sur D, et al. Identification of burden hotspots and risk factors for cholera in India: An observational study. PLoS One. 2017;12(8).

3. Griffith DC, Kelly-Hope LA, Miller MA. Review of reported cholera outbreaks worldwide, 1995-2005. Am J Trop Med Hyg. 2006;75(5):973–7.

4. Diseases IS for I. Promed Post – ProMED-mail [Internet]. 20191113.6774475. 2019 [cited 2020 Mar 23]. Available from: [https://promedmail.org/promed-post/?id=6774475]

5. ODF Sangli focuses on sustainability [Internet]. 2018 [cited 2020 May 3]. Available from: [https://sujal-swachhsangraha.gov.in/node/2448]

## A58 Outdoor Air Pollution and respiratory diseases, Faridabad, Haryana 2018

### Manoj Bajaj, Manikandanesan Sakthivel, Tarun Bhatnagar

#### ICMR-National Institute of Epidemiology, Chennai, Tamil Nadu, India

##### **Correspondence:** Tarun Bhatnagar (drtarunb@gmail.com)

**Background**

Air pollution has become one of the major public health problems across the globe. According to the World Health Organization (WHO), in 2016, about 4.2 million premature deaths worldwide were due to ambient air pollution [1]. Exposure to PM_2.5_ was the 5^th^ highest-ranking risk factor for death, evident from the analysis of the Global Burden of Diseases (GBD) 2015, and it was responsible for 4.2 million deaths from heart disease and stroke, lung cancer, chronic lung disease, and respiratory infections [2]. Out of 4.2 million, 91% deaths occurred in low-and-middle-income countries. India was listed in the table of countries with the high level of estimated population-weighted mean concentrations of PM_2.5_ in the report submitted by the Lancet commission on air pollution [3]. The same report pointed out that China and India combined had the largest numbers of attributable deaths and DALYs due to air pollution: 52% and 50% of the respective global total. As per the Centre for Science and Environment report, air pollution was the third-highest cause of all premature deaths in India [4]. Air pollution has detrimental effects on most of the organs of the human body. Besides aggravating cardiovascular and respiratory disease, it has an impact on the nervous system, urinary system, and digestive system as well.

Faridabad, a city in Haryana state, India, has been declared as the second most polluted city in the world by WHO through monitoring of ambient air pollution from 2008-2017 represented by the annual mean concentration of particulate matter PM_10_ and PM_2.5_. In this study, we aimed to study the seasonal trend of PM_2.5_ in Faridabad, 2016 – 18 and to estimate the correlation between air pollutants and the number of out-patients & in-patients in Faridabad, 2016-18.

**Methods**

We did a cross-sectional analysis of the level of air pollutants and respiratory illness in Faridabad District, Haryana, India, 2016-2018. Haryana Pollution Control board monitors the pollution parameters- PM_10_, PM_2.5_, SO_2_, NO_2,_ and CO in the ambient air daily. We abstracted the mean monthly average of these pollution parameters from January 2016 to December 2018 from the records maintained at the monitoring station. For the same period, we abstracted data on the monthly census, in-patients, and out-patients with respiratory illness from the Medical Records Department of one public and private sector healthcare facility in Faridabad.

We plotted a line graph to describe the monthly trend of PM_2.5_ levels from 2016–2018. We did Spearman’s rank correlation to test the association between pollution parameters and the number of patients with respiratory illness between 2016 and 2018. Since the pollution parameters were collinear and the correlation coefficient was greater than 0.5, we used the principal component analysis to extract the common factors among the pollution parameters. We identified PM_2.5_, CO, NO_2_ (Factor 1), and CO, NO_2_ (Factor 2) as component factors and used them in Spearman’s rank correlation.

**Results**

We found a seasonal trend in pollution parameters, decreasing from May to September months. The mean monthly concentration of PM_2.5_ remained above the permissible annual time-weighted average level for all three years, in contrast to other pollutants under investigation, below the permissible limits. We found a significant positive correlation between factor 2 (CO, NO_2_) and the total number of patients, the total number of out-patients, and the number of patients with asthma, bronchitis, and acute respiratory infection (ARI). Factor 1 (PM_2.5_, CO, NO_2_) had a significant positive correlation with the total number of patients who visited the facilities and the number of patients with bronchitis (Table 1).

**Conclusions**

The level of PM_2.5_ was consistently above permissible limits, except during the monsoon months of July – August every year. The level of PM_2.5_ was poor (201-300) during the winter months of October – January. There was a moderate correlation between pollutant levels and the total number of out-patients and patients with ARI.

**References**

1. World Health Organization. Ambient (outdoor) air pollution [https://www.who.int/news-room/fact-sheets/detail/ambient-(outdoor)-air-quality-and-health]. Accessed 9 Feb 2021.

2. Forouzanfar MH, Afshin A, Alexander LT, Anderson HR, Bhutta ZA, Biryukov S, et al. Global, regional, and national comparative risk assessment of 79 behavioural, environmental and occupational, and metabolic risks or clusters of risks, 1990–2015: a systematic analysis for the Global Burden of Disease Study 2015. The Lancet. 2016 Oct;388(10053):1659–724.

3. Landrigan PJ, Fuller R, Acosta NJR, Adeyi O, Arnold R, Basu N (Nil), et al. The Lancet Commission on pollution and health. The Lancet. 2018 Feb;391(10119):462–512.

4. Anumita Roychowdhury, Vivek Chattopadhaya, Gaurav Dubey, Swati Singh Sambyal, Avikal Somvanshi, Shambhavi Shukla and Tanushree Ganguly 2019, 5 June: At the Crossroads, Centre for Science and Environment, New Delhi. [https://www.cseindia.org/content/downloadreports/9464]. Accessed 9 Feb 2021.

**Table 1 (abstract A58).** Correlation between air pollutant levels and number of patients attending two tertiary care hospitals, Faridabad, Haryana, India, 2016-2018

Factors		Total patients	Total OPD	Total IPD	Asthma	Bronchitis	ARI
CO and NO_2_	Correlation Coefficient	.404^*^	.348^✝^	-.078	.287^✝^	.344^✝^	.366^✝^
	Sig. (1-tailed)	.007	.019	.326	.045	.022	.014
	N	36	36	36	36	35	36
CO, NO_2_ and PM_2.5_	Correlation Coefficient	.323^*^	.223	.001	.199	.313^*^	.192
	Sig. (1-tailed)	.027	.095	.497	.123	.034	.131
	N	36	36	36	36	35	36

*Significant at 0.01 level (1-tailed)

^✝^Significant at 0.05 level (1-tailed)

## A59 Quality of delivery services in public sector primary care health facilities in Saidapet Health Unit District, Kanchipuram District, Tamil Nadu, India, 2017-18 - A cross-sectional quality assessment evaluation

### Arun Nagamuthu, Kamaraj Pattabi, Prabhdeep Kaur

#### ICMR-National Institute of Epidemiology, Chennai, Tamil Nadu, India

##### **Correspondence:** Arun Nagamuthu (arun23moscow@gmail.com)

**Background**

India contributes to 19% of the global maternal deaths, despite a 59% decline in maternal deaths during the last decade. Despite the significant increases in institutional deliveries, there was limited evidence to support that the JSY program has contributed to improved maternal or neonatal survival [1,2]. In India, deliveries are conducted by staff nurses and Auxiliary Nurse Midwives at the primary care level. Despite the rapid improvement in skilled birth attendants and facility births, progress in achieving desired outcomes and impact remained slow [1,3]. Evidence shows that the highest number of maternal and newborn deaths occur during delivery and in the early postpartum phase. One of the key factors which influence maternal and child death is the Quality of Care of service. In recent years, more focused attention has been given to the quality of care for maternal and newborn care, particularly in low- and-middle-income countries (LMICs) [4,5].

In Tamil Nadu, most of the deliveries are institutional, and the MMR is below the national average. Saidapet Health Unit District (HUD), Kanchipuram in Tamil Nadu has a Maternal Mortality Rate of 92/100,000. The state aims to reduce MMR further, and therefore the program managers want to improve the quality of delivery and postpartum care. There is limited data regarding the quality of services in the state. A new tool has been introduced by the Government of India to monitor the quality of care. We estimated the overall quality of delivery services based on the ‘National Quality Assessment Standards’ (NQAS) tool in primary health centers and community health centers in Saidapet HUD, Tamil Nadu, India.

**Methodology**

We conducted facility based cross-sectional surveys in all public sector health facilities during December 2017 to March 2018 in Saidapet HUD, Kanchipuram district, Tamil Nadu. We included 20 primary health centres (PHCs) and six community health centres (CHCs) in the survey. We collected data for six quality domains including Service Provision, Patient Rights, Input, Support services, Clinical Care, Infection Control, quality management and outcomes. We used the National Quality Assessment Standard Tool in an android app named “Guide for NQAS and kayakalp (GUNAK). We reviewed the records and interviewed nurses on duty. For scoring, two marks were given for full compliance (>= 70%), one mark for partial compliance (< 70%) and 0 Marks for Non-Compliance. The score of 70 and above were considered as full compliance. All checkpoints were given equal weight and the final score was given for comparison purposes. Descriptive statistics were used for analysis.

**Results**

The overall mean score of PHCs (n= 20) was 79.4 (SD 3.6). Among eight domains, the mean score of six domains were more than 70, and the mean score for Quality management and Outcome was 46.8 (SD 20.2) and 45.4 (SD 10.6) respectively. Among the eight domains, in Quality management only two PHCs scored more than 70, and in Outcome, none of the PHCs scored 70 and above (Table 1).

The overall mean score of CHCs (n= 6) was 73.8 (SD 5.0). The mean score for Quality management and Outcome was 37.3 (SD 2.8) and 44.0 (SD 5.0) respectively, and all the remaining six domains scored more than 70. None of the CHCs scored more than 70 in Quality management and Outcome.

The overall mean score of PHCs and CHCs combined was 78.1 (SD 4.5). Out of eight domains, the mean score for six domains was more than 70. Only Quality management and Outcome domains had 44.6 (SD 18.1) and 45.0 (9.6) mean scores. Majority (24/26) of the facilities achieved the overall score of 70 and above. Only two (8%) facilities scored more than 70 in Quality management and none scored above 70 in outcomes.

**Conclusion**

Overall score of PHCs and CHCs were above 70 and 92% of facilities in Saidapet Health Unit District functioned with full compliance. The low scores in Quality management and outcome domains were a cause of concern.

The main reason for low score in quality management was the lack of training about new standard operating procedures (SOPs). In addition, in CHCs, there was no designated person for Quality assurance activities. Facilities require establishment of an organizational framework for quality improvement. The low scores in outcome domain were possibly due to low number of deliveries conducted at night time which was one of the criteria for scoring. Another area which lowered the score was lack of assisted deliveries at CHC level. This may be due to lack of training for Medical officers and non-availability of specialists. Other indicators which contributed to poor scores were lack of maintenance of records for data such as episiotomy site infection rate and culture surveillance sterility rate.

The NQAS tool was the best available option to measure the quality, but there were some limitations because we interviewed only the staff nurse on duty when we visited. The skills/competence of other staff in facilities could not be assessed. National Quality Assessment Standards training for service providers should be conducted to further improve the performance in Quality Management and Outcomes.

**References**

1. Randive B, Diwan V, De Costa A. India’s Conditional Cash Transfer Programme (the JSY) to promote institutional birth: Is there an association between institutional birth proportion and maternal mortality?. PloS one. 2013 Jun 27;8(6):e67452.

2. Powell-Jackson T, Mazumdar S, Mills A. Financial incentives in health: New evidence from India's Janani Suraksha Yojana. Journal of health economics. 2015 Sep 1;43:154-69.

3. Lim SS, Dandona L, Hoisington JA, James SL, Hogan MC, Gakidou E. India's Janani Suraksha Yojana, a conditional cash transfer programme to increase births in health facilities: an impact evaluation. The Lancet. 2010 Jun 5;375(9730):2009-23.

4. Souza JP, Gulmezoglu AM, Vogel J, Carroli G, Lumbiganon P, Qureshi Z, Costa MJ, Fawole B, Mugerwa Y, Nafiou I, Neves I. Moving beyond essential interventions for reduction of maternal mortality (the WHO Multicountry Survey on Maternal and Newborn Health): a cross-sectional study. The Lancet. 2013 May 18;381(9879):1747-55.

5. Tripathi V, Stanton C, Strobino D, Bartlett L. Development and validation of an index to measure the quality of facility-based labor and delivery care processes in sub-Saharan Africa. PLoS One. 2015 Jun 24;10(6):e0129491

**Table 1 (abstract A59).** Domain-specific scores of the QoC using NQAS tools in public sector primary care health facilities, Saidapet Health Unit District, Kanchipuram District, Tamil Nadu, India, 2017-18

Domains	Primary Health Centers (N=20)	Community Health Centers	All (N=26)
	Mean (SD)	Mean (SD)	Mean (SD)
Service Provision	78.0 (6.1)	78.5 (8.3)	78.1 (6.5)
Patient Rights	83.4 (9.7)	83.0 (5.7)	83.2 (8.9)
Inputs	79.5 (3.4)	77.8 (5.1)	79.1 (3.8)
Support Services	84.5 (6.5)	84.6 (6.7)	84.5 (6.4)
Clinical Services	91.7 (6.3)	77.8 (6.3)	88.5 (6.9)
Infection Control	75.9 (7.9)	77.1 (4.9)	76.1 (7.3)
Quality Management	46.8 (20.2)	37.3 (2.8)	44.6 (18.1)
Outcome	45.4 (10.6)	44.0 (5.0)	45.0 (9.6)
Overall	79.4 (3.6)	73.8 (5.0)	78.1 (4.5)

## A60 Referral mechanism in breast cancer screening program in Tiruchirappalli district, Tamil Nadu, India, 2012-15

### Vidhya Viswanathan^1^, Parasuraman Ganeshkumar^2^, Jerard Maria Selvam^1^, TS Selvavinayagam^1^

#### ^1^Department of Public Health and Preventive Medicine, Government of Tamil Nadu, Chennai, India; ^2^Scientist D, ICMR- National Institute of Epidemiology, Chennai, India

##### **Correspondence:** Parasuraman Ganeshkumar (ganeshkumardr@gmail.com)

**Background**

Breast cancer is the most common cancer among Indian women, affecting nearly 22 per one lakh women population [1]. Early detection through screening makes the disease easier to treat, achieve better cure rates, avoid late-stage complications, and reduce mortality. Tamil Nadu, a southern state in India with a population of 72 million, had launched a Noncommunicable diseases (NCDs) intervention program supported by the World Bank during 2011 targeting four major NCDs viz hypertension, diabetes, cervical cancer, and breast cancer. Under this mass screening program, all women aged 30 years and above were screened for cervical and breast cancer and further evaluated at various levels for confirmation and treatment. Referral from the first to the second level of care hinders continuity of diagnosis or treatment. Poor referral and low adherence rate remain a challenge to the cancer prevention programme across the world [2-4]. Ensuring proper referral of at-risk individuals and adherence of beneficiaries to the referral following screening decides the success of such screening program. So, we evaluated the program with the objectives to describe the referral mechanism in breast cancer screening program, estimate the adherence of beneficiaries and identify the programmatic strengths and challenges of the referral mechanism.

**Methods**

We conducted a cross-sectional study between November 2015 and October 2016 in the Tiruchirappalli district with 27.2 lakhs in Tamil Nadu. Our study population was women aged 30 years and above, screened for breast cancer between April 2012 to March 2015 in 75 government health care facilities. We reviewed the registers at government health facilities and prepared the line list of women screened positive for breast cancer. We collected reports on further evaluation, diagnosis, and treatment available at government facilities and private facilities empanelled under the state insurance scheme. We did a secondary analysis of the women screened positive line list for breast cancer using Epi info version 7 and SPSS version 22. A master line list of the screened positive women was prepared from the screening facilities, and duplicates were removed. Individual data on referral and diagnostic details from public and empanelled private health facilities were matched using a Java program in consultation with a software data analyst. We reviewed program monitoring and evaluation reports and interviewed programme managers on human resources, training, and equipment. The primary outcome measures were screening coverage and dropout rates at different stages of the referral mechanism. We also identified the strengths and weakness of the referral mechanism.

**Results**

Sixty-four PHCs, 10 CHCs, and one tertiary government facility was screening women aged 30 years and above for breast cancer using Clinical Breast Examination (CBE). Women screened positive were referred for further evaluation by procedures like Mammogram, USG breast, Fine Needle Aspiration Cytology (FNAC), or biopsy at secondary or tertiary care or any of the empanelled private care facilities. Screened negative women were advised to come for repeat screening after two years. The estimated target women population was 703,583, and 500,651 were screened, resulting in an overall screening coverage of 71.2% for three years (Figure 1). Of those 500,651 women screened, 4312(0.86%) were found positive and referred for further diagnostic evaluation through mammography/USG breast/FNAC or biopsy. Only 1,668 (38.7%) of the women underwent further evaluation, and 2644 (61%) were lost to follow-up. Among those who have undergone diagnostic evaluation, data was not available for 361(22%) women, and of the remaining 1,307(88%) mammogram or USG breast was done for 275(16%) and FNAC or biopsy for 1,032(62%). Among those 1,307 women who underwent an evaluation, 87(6.7%) were diagnosed with cancer, and 719 (55%) with benign and inflammatory lesions, and 232(18%) were normal. The details of 269(20%) were not available. Tertiary care facilities failed to maintain treatment details adequately owing to the mass screening and intervention approach. Data on repeat screening for those tested negative were also not available.

The strengths observed in the programme were fair geographical distribution, the strong network of the health care facilities, and dedicated, trained staff nurses at all screening centers. The diagnostic and treatment services from the private sector were linked through the state health insurance scheme. Other strengths include community-based IEC activities and multi-sectoral coordination with the education department, such as creating awareness among students through chapters on cancers included in the middle school curriculum and the rural development department in creating awareness among women through activities by the intermediary committee formed by a group of local women. We observed that the programme`s significant challenges included deficiency of a case-based tracking system, unlinked data systems between the different referral units, partial digitalization of data, incomplete diagnostic and treatment details, and weak monitoring & evaluation. The program managers opined that the acceptance and adherence of women towards services were also low.

**Conclusions**

The present study identified that nearly more than half of the target population was screened for breast cancer in public facilities. But, more than half of the screened positives were lost to follow-up for further evaluation. The data of around one-fifth of women were incomplete at both assessments. The data management at the tertiary center was inadequate and needed additional focus. Nevertheless, the system had a strong trained and dedicated human workforce. The major challenges included insufficient documentation of individual patient data. Our review was based on the secondary analysis of the data obtained from the public health facility records.

The referral mechanism was envisaged to have linkages at various levels of care but the same could not be operationalized at the field level. Efforts to improve data management and to track individual data with unique ID across three levels of care should be consistently applied to strengthen the referral mechanism.

**References**

1. India State-Level Disease Burden Initiative Cancer Collaborators. The burden of cancers and their variations across the states of India: the Global Burden of Disease Study 1990-2016. Lancet Oncol. 2018 Oct;19(10):1289-1306. doi: 10.1016/S1470-2045(18)30447-9. Epub 2018 Sep 12. Erratum in: Lancet Oncol. 2018 Oct 3;: PMID: 30219626; PMCID: PMC6167407.

2. Percac-Lima S, Aldrich LS, Gamba GB, Bearse AM, Atlas SJ. Barriers to follow-up of an abnormal Pap smear in Latina women referred for colposcopy. J Gen Intern Med. 2010 Nov;25(11):1198-204. doi: 10.1007/s11606-010-1450-6. Epub 2010 Jul 21. PMID: 20652647; PMCID: PMC2947627.

3. Lacey L, Whitfield J, DeWhite W, Ansell D, Whitman S, Chen E, Phillips C. Referral adherence in an inner city breast and cervical cancer screening program. Cancer. 1993 Aug 1;72(3):950-5. doi: 10.1002/1097-0142(19930801)72:3<950::aid-cncr2820720347>3.0.co;2-s. PMID: 8334648.

4. Jeong SJ, Saroha E, Knight J, Roofe M, Jolly PE. Determinants of adequate follow-up of an abnormal Papanicolaou result among Jamaican women in Portland, Jamaica. Cancer Epidemiol. 2011 Apr;35(2):211-6. doi: 10.1016/j.canep.2010.07.004. Epub 2010 Aug 4. PMID: 20688592; PMCID: PMC3062074.

**Fig. 1 (abstract A60).**
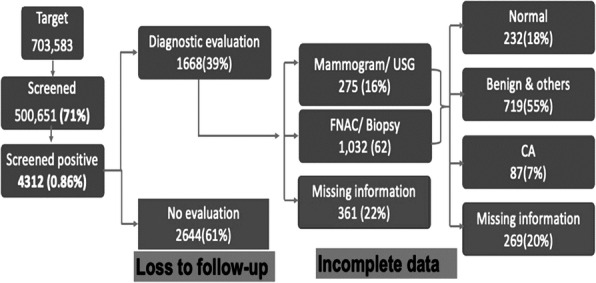
Referral mechanism in screening and evaluation of breast cancer, Tiruchirappalli District, Tamil Nadu, India 2015-16

## A61 Investigation of an acute gastroenteritis outbreak in a hilly village of Bilaspur district, Himachal Pradesh, May 2019

### Harit Puri^1^, Polani Rubeshkumar, Parasuraman Ganeshkumar^2^

#### ^1^Directorate of Health Services, Himachal Pradesh, India; ^2^ICMR- National Institute of Epidemiology, Chennai India

##### **Correspondence:** Parasuraman Ganeshkumar (ganeshkumardr@gmail.com)

**Background**

In India during 2014 more than 300 foodborne outbreaks were reported in Integrated Disease Surveillance Program [1]. As only a fraction of the people who become sick after consuming contaminated food seek medical care, measuring burden of foodborne diseases is a challenge. Thus, foodborne diseases would always be underreported [2]. Absence of a well-structured outbreak investigation would lead to incomplete control of it and would not generate knowledge for prevent future outbreaks [3]. Most of the foodborne outbreaks are reported in gatherings and feasts. Data on foodborne outbreaks and its etiological agents were limited in literature [4]. In the night of May 25, 2019, few people from village Kog in Himachal Pradesh presented to the emergency department of Regional hospital located in the Bilaspur district, Himachal Pradesh with symptoms of diarrhea, vomiting and abdominal pain. An acute gastroenteritis outbreak was suspected. On May 26, 2019 40 patients with similar complaints from same village were admitted in the hospital. Patients reported that they all had attended a death anniversary in the village. We further investigated this suspected outbreak to describe the epidemiology, identify the potential exposure and recommend control measures.

**Methods**

A retrospective cohort study was conducted and active case search was done with the case definition of acute gastroenteritis as, “occurrence of loose stools or vomiting with or without nausea, abdominal pain among the attendees of death anniversary ceremony dinner in Kog village since 7 PM on 25 May 2019”. We conducted door to door active case search with the above case definition to search for more cases and framed the line-list of cases. Medical records were reviewed and interviews were conducted for the cases. We also conducted interviews among food handlers. We obtained information on sociodemographic details, date and time of onset of illness, symptoms, hospitalization, outcome, history of food items consumed and food handling practices. We collected stool samples from five patients and sent for analysis to the Regional hospital of Bilaspur district. We also sent water samples for microbiological analysis. We described the time distribution of cases by data of onset of symptom as epicurve and distribution by age and gender as proportions. We estimated the attack rate by age and gender with total number of attendees of dinner as denominator. We calculated the food-specific attack rates, relative risks with 95% confidence intervals using epi info software.

**Results**

We identified that all cases admitted in the Bilaspur hospital shared a commonality of attending a dinner of death anniversary on May 25, 2019. There were 72 persons attended the death anniversary dinner and of which 44 were identified by case definition by active case search with attack rate of 61% (44/72). The food was served at 7 PM of May 25,2019 and all the patients reported the acute gastro-enteritis between 4 AM to 2 PM of May 26, 2019 with peak being at 9 AM to 10 AM. Attack rate was high among the age-group 6-14 years (14 out of 19 persons) and among females (31 out of 41 persons). We hypothesized that the outbreak could be due to dinner served in the dinner of death anniversary. Five food items were served in the dinner viz. Rajma (Red Kidney beans curry), Dal (watery dish made of pulses), Cooked rice, Kadhi (Gram flour-based gravy with vegetable fritters) and Meetha (sweet dish). During the interview of attendees, many expressed that Rajma was undercooked and the food handlers opined that outdated corn flour was used to prepare the Rajma. Attack rate of Rajma (Red Kidney beans) was 77%(40/77) and those who ate Rajma were nearly three times (RR: 2.7; 95% CI: 1.1-6.2) more risk of developing gastro-enteritis. No organism was isolated among the water samples and E.coli was isolated from all the five stool samples.

**Conclusions**

An outbreak of Acute Gastroenteritis at village Kog of Himachal Pradesh was due to consumption of contaminated Red Kidney beans served in the dinner of a death anniversary. No food samples available from the dinner served for laboratory tests. Safe food preparation and handling practices were educated among the food handlers and regulatory action on violation of hygiene were also disseminated. Local health authority mandated registration of all food handlers under their jurisdiction and training on hygienic food handling practices with hand washing technique were conducted.

**References**

1. NCDC Quaterly Newsletter. New Delhi, India: National Centre for Disease Control Directorate General of Health Services Ministry of Health and Family Welfare, Government of India; 2015. https://www.ncdc.gov.in/linkimages/NewsletterMarch20153160226752.pdf. Accessed 20 Aug 2020.

2. Bhaskar SV.Foodborne diseases—disease burden. In Food Safety in the 21st Century.Academic Press; 2017. pp. 1–10

3. Murhekar M, Moolenaar R, Hutin Y, Broome C. Investigating outbreaks: practical guidance in the Indian scenario. Natl Med J India. 2009 Sep-Oct;22(5):252-6. PMID: 20334049.

4. Rao Vemula S, Naveen Kumar R, Polasa K. Foodborne diseases in India – a review. British Food Journal. 2012; 114:661-80.

**Table 1 (abstract A61).** Food-specific attack rates and the relative risks in an acutre gastroenteritis outbreak, Kog village, Himachal Pradesh, India 2019

Food Items	Those who ate			Those who didn’t eat			RR	95% CI
	Cases	Total	Attack rate (%)	Cases	Total	Attack rate (%)		
Mitha	44	66	67	0	6	0	-	-
Kadi	44	67	66	0	5	0	-	-
Rajma	40	52	77	4	14	28	2.7	1.1-6.2
Dal	34	62	55	8	10	80	0.7	0.5-1.0
Rice	44	72	61	0	0	0	-	-

## A62 Kyasanur Forest Disease in Wayanad District, Kerala, India, 2019

### Noona Marja K.M^1^, Mohan Kumar R^2^, Dineesh P^3^, Dileep^4^,

#### ^1^District Surveillance officer, Wayanad, Kerala; ^2^Consultant epidemiologist ICMR-NIE Chennai; ^3^MPH Scholar NIE Chennai; ^4^District epidemiologist, Veterinary, Wayanad, Kerala

##### **Correspondence:** Noona Marja K.M (noonu28@yahoo.in)

**Background**

Kyasanur forest disease (KFD) is a hemorrhagic febrile illness characterized by fever, and prevalent in Shimoga district of Karnataka. It is caused by the virus of the family flaviviridea. Monkey is the predominant reservoir animal. The virus is transmitted from the vector to human by Haemaphysalis spinigera, a forest tick. Human is a terminal host and no human-to-human transmission is documented. This disease is endemic in Karnataka state.

Wayanad district is in north eastern part of Kerala state. It is set high on the Western Ghats with altitude ranging from 700-2100. About 886 Square Kilometer (42%) of the area of the district is under forest. There are many indigenous tribes in the area. Wayanad district is bordered by Karnataka to north and north east and Tamil Nadu to south east. Elephant, dear, bear, Tiger and different monkey species from the neighboring wild life sanctuaries of Karnataka and Tamilnadu stray into the forest range of Wayanad. According to 2011 census Wayanad district had a population of 817,420. Wayanad district is having 17% of tribal population. Population migration of monkey troops and other reservoir hosts is noticed during summer season. Change in rainfall pattern and change in vegetation also occurred.

The first case of KFD was reported in 2013. This triggered KFD surveillance in the district since 2013. The surveillance in included as part of Integrated disease surveillance programme (IDSP), where 23 primary health centres, eight community health centres and three major hospitals report. In addition to the routine surveillance, initiatives like inter sectoral co-ordination with forest, police, tribal, health, etc., Strengthening of fever reporting in both public and private, laboratory surveillance, were strengthened for better disease surveillance. Wayanad reported higher number of KFD cases in the year 2015. We analysed the KFD surveillance of 2019 and compared the disease trend of Wayanad.

**Methods**

We did the study in Wayanad district, Kerala. We did a secondary data analysis of KFD cases reported in Wayanad district for the year 2019. We collected the data for the period from January to December 2019. We defined suspected cases of KFD as any person with fever from areas where monkey deaths were reported in Wayanad district from January 2019. A confirmed case is defined as suspected case with Reverse transcriptase (RT- PCR) positive for KFD. We abstracted data from the line list of KFD cases from the office of DMO, Wayanad by using a data abstraction format. We entered the data in Microsoft excel. We did descriptive analysis of the cases in terms of time, place and person

**Results**

KFD surveillance system captured 12 suspected cases of KFD in the year 2019. The median age of cases was 30 years with a range of 18-50 years. Males (84%) were more affected than females. Symptoms presented by the cases include fever (100%), headache (33%), vomiting (25%), loose stools (25%) and disorientation (8%). The first case was reported on 25th January 2019. The cases increased in March with 33% (n=8) cases identified till the peak. The last case was reported on 8th June 2019. Almost all cases were reported from the tribal village of Wayanad district. Of the 12 probable cases tested, seven (58%) were (RT PCR) positive for KFD. Two (16%) died. The trend is similar to the previous years (Figure), but not peaked as 2005.

**Conclusion**

KFD is reported in the tribal areas of Wayanad district with mostly adult males affected. This is probably due to the population accessing the forest area for their livelihood. Increasing awareness among tribal population to follow precautionary measures to avoid tick bite may prevent them from KFD. Studies need to be conducted to find reservoir host and transmission cycle of KFD.

**Fig. 1 (abstract A62).**
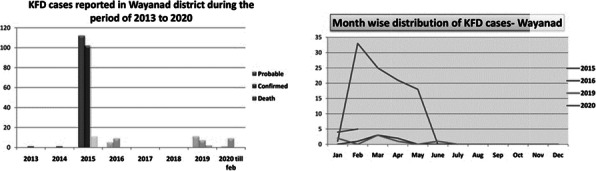
Kyasanur Forest Disease Surveillance in Wayanad, Kerala 2013-2020

## A63 Dengue epidemiology in Kancheepuram district, Tamil Nadu, India, 2016 to 2018.

### Sangeetha Subramaniyan, M SreeKalpana, Mohankumar Raju

#### National Institute of Epidemiology, Chennai, India

##### Sangeetha Subramaniyan (sangsubramaniyan@gmail.com)

**Introduction**

Dengue infection is a major mosquito-borne viral illness. It is reported more in tropical and subtropical countries. Prevalence of Aedes aegypti and Aedes albopictus together with circulation of dengue virus of more than one serotype in an area leads to an outbreak of dengue. There has been a global increase in the incidence of dengue fever over the past three decades. An estimated 2.5 billion people i.e. two-fifths of the world’s population in the tropics and subtropics are at risk of the disease. An estimated 50 million dengue infections occur worldwide annually and about 500,000 people with dengue haemorrhagic fever require hospitalization. Outbreaks of dengue are increasing in frequency.

Dengue and Dengue Haemorrhagic Fever are endemic in more than 100 countries in the World Health Organisation regions of Africa, America, Eastern Mediterranean, South East Asia, and Western Pacific [1]. In India, dengue risk has increased in recent years due to rapid urbanization, lifestyle changes and, poor water management. The improper water storage practices in urban, peri-urban, and rural areas have led to increased mosquito breeding sites [2]. The disease has a seasonal pattern, it peaks after the monsoon. It shows a decrease in trend with the implementation of control measures.

The dengue burden in Tamil Nadu increased from 2531in 2016 to 20,597 in 2017 and has declined in 2018. Tamil Nadu has seen a periodic occurrence of dengue cases with varying severity from August to December. In 2017 the number of case-patients steeply increased in Tamil Nadu. Kancheepuram district showed an increase in the incidence of cases in 2017 and with the implementation of timely control measures, the number of cases declined in 2018.

We analyzed the dengue data of the Kancheepuram district of Tamil Nadu to describe the dengue epidemiology to give recommendations.

**Methods**

We did a descriptive study among the residents of Kancheepuram district in November 2019. Our reference period for the study was between January 2016 and December 2018. We defined a confirmed case of dengue as any resident of Kancheepuram district, whose serology was positive for either NS1 ELISA or IgM ELISA in any of the recognized laboratories all over Tamil Nadu. We collected the data from the district surveillance unit segregated by age, gender and block. The MS excel sheet of the patient line list was cleaned and coded. We used the 2011 census data for the distribution of the population by age group and gender. The attack rates were calculated based on age, gender and place of residence.

**Results**

There were 1871 confirmed dengue cases. Attack rate was 47 per 1 lakh population (1871/3998252) for the year 2017. Median age of the cases was 23 years (range: 1 month to 84 years). Attack rate was higher in the age group lesser than the median age 23 years, (AR 61 / lakh (968/1585415)) than the age group more than 23, (37 / lakh (891/2407256)). Attack rate was higher among the males (55 / lakh, (1111/2010068)) than females (38 / lakh (760/1982603)). There was an increasing trend in 2017 (AR = 32 / lakh, (1297/3998252)). Attack rate was (5/ lakh (193/3998252)) in 2016 and 10 /lakh {381/3998252} in 2018. Attack rate was higher in rural areas (AR =80/lakh (691/ 1479353)) when compared to urban (AR= 27 /lakh, (1179 /2518899)).

**Conclusion**

In 2016 dengue cases increased in incidence from July and peaked in November 2016. The age group affected was from 1 year to 84. The median age was 23. The attack rate was calculated for the years from 2016 to 2018. 2016 the attack rates showed a steep increase. The median age of attack was 23 years. The cases in the age group lesser than 23 were higher than the cases in the age group greater than 23. The attack rate was higher in males than in females. The attack rate was higher in rural areas.

With the strengthening of dengue control activities showed a gradual decrease in December 2016 with the decreasing trend continuing in 2017 upto April and thereafter maintained at low levels upto 2018. We recommend further studying the risk areas, identify hotspot areas, undertake continuous surveillance and investigate the impending outbreaks in the risk areas. The initiation of control measures well in advance will help in averting outbreaks.

References

1. World Health Organization. Dengue: Guidelines for Diagnosis, Treatment, Prevention and Control. Geneva: World Health Organization and the Special Programme for Research and Training in Tropical Diseases, 2009.

2. Gubler DJ. Dengue, Urbanization and Globalization: The Unholy Trinity of the 21(st) Century. Trop Med Health. 2011 Dec;39(4 Suppl):3-11. doi: 10.2149/tmh.2011-S05. Epub 2011 Aug 25. PMID: 22500131; PMCID: PMC3317603.

## A64 Family Planning Services of Tamil Nadu – An understanding from National Family Health Survey - 4, 2015-16

### Mohankumar Raju, Vidhya Vishwanathan, Savitha AK

#### Consultant Epidemiologist, National Institute of Epidemiology, Indian council of medical research, Chennai

##### **Correspondence:** Mohankumar Raju (rmkhari2000@yahoo.com)

**Background**

Family welfare (FW) services are emphasised as one of the community interventions strategy for better health care of the women. India is the pioneer to launch the National programme on Family planning in 1952. FW services are centrally funded to the states and union territories, focusing on reducing the crude birth rate and infant mortality rate by promoting family welfare services. The main focus of the FW services include increase in awareness among the community, availability and accessibility of Significant achievements of the FW services include a reduction in crude birth rate from 40.8 (1951 Census) to 27.4 in 1996 (SRS 96), reduction in infant mortality rate from 146 in 1951 to 72 in 1996 (SRS). The increase in couple protection rate from 10.4% (1970-71) to 45.4% (31.3.1997) is also witnessed [1].

India is a signatory to Millennium declaration at UN general assembly in 2000 and affirmed its commitment to better maternal health care. The strategy of availability of trained medical professionals at the field level, infrastructure development for maternal health care and financial schemes made a significant impact in better maternal health care of the community [2,3].

Tamil Nadu is one of the better healthcare providing state in India with better MCH care. Temporary and permanent sterilisation methods are offered free of cost by the public health department for both men and women. There is a decline of contraceptive prevalence rate from 61% in NFHS-3 in 2005-06 to 53 % in NFHS 4. We analysed the family welfare data of NFHS-4 to understand the family welfare services of Tamil Nadu.

**Methods**

International Institute of Population Sciences, Mumbai conducted the NFHS in August 2015, collaborating with other national and international agencies [4]. For Tamil Nadu, NFHS 4 covered a total population of 28,820 in the survey.

We did a secondary data analysis of NFHS 4 data for the districts of Tamil Nadu. We collected the information from the NFHS 4 fact sheets report [5] and extracted in an excel sheet. Similarly, we obtained information on the districts of Tamil Nadu covered in the NFHS survey. We used epi-info for analysis. We calculated proportions of family welfare services and compared between districts.

**Results:** Population of 28,820 were surveyed in 32 districts of Tamil Nadu. The literacy rate of the surveyed women was 79%, with higher proportion among the urban (84.8%) than rural (73.2%). Two districts had prevalence more than 90 (Nagercoil (95.4%) and Chennai (90.2%)). The districts with lower literacy rate of less than 70% include Ariyalur (69%) and Villupuram (69.5). The literacy rare is higher among the males (89%) compared to females, with higher proportion among the urban (90.8%) than rural (86.9%). One in three (n=12) districts had more than 90% literacy rate among males; Chennai (96.7%) topped followed by Tirunelveli (96%). Villupuram (72.7%) showed lowest proportion among the male literacy.

The contraceptive prevalence rate of Tamil Nadu is 50% equal prevalence among the urban and rural population. Around 9 districts have more than 60% prevalence. Coimbatore (65.4%) showed higher prevalence on any method of sterilisation followed by Vellore and Tiruvallur (64% each). Two districts, Virudhunagar (23.3%) and Ramanathapuram (26%) showed poor coverage of any method of sterilisation (Fig 1). Use of modern method was 49.5% with 8 districts show more than 60% prevalence. Virudhunagar (23%), Ramanathapuram (25.6%) and Thoothukudi (29.7%) showed coverage of any modern method of sterilisation below 30%. Female sterilisation for permanent methods is 46% with higher rate in rural (47%) compared to urban (45%). Male sterilisation was not reported in 91% (29/32) of the districts. Tirunelveli (0.2%), Coimbatore (0.1%) and Cuddalore (0.1%) are the districts showed prevalence of male sterilisation. The utilisation of temporary methods of sterilisation is 2.1% by females and 0.8% by males. Use of Intra uterine devices (1.2%) was the commonest temporary method of sterilisation followed by condom (0.8%) and pills (0.2%). The use of temporary methods is higher among the urban population than rural population. Health worker ever talked to female non-users about family planning was 30.2% with similar coverage in rural (30.8%) and urban (29.7%). One in ten women in Tamil Nadu is not using contraception methods but want to postpone childbearing or stop altogether.

**Conclusion:** The contraceptive coverage of Tamil Nadu is covered for half of the needed. Although half of the eligible women underwent any form of sterilisation, coverage of sterilisation among men needs improvement. The coverage of sterilisation methods is higher among the districts with higher education status. Improving the focus of the health workers on family welfare services will appreciably alleviate the unmet needs of contraception among women and improve their health status.

**References**

1. Aayog N. Planning Commission-Annual Report 2014-15

2. Ministry of statistics and program implementation, Government of India. Millennium Development Goals India Country Report 2015; India 2015.

3. United Nations. India and the MDGS: Towards a sustainable future for all. UN India. 2015.

4. IIPS I. National Family Health Survey (NFHS-4), 2015–16: India. Mumbai: International Institute for Population Sciences. 2017.

5. International Institute for Population Sciences. National Family Health Survey-4, State fact sheet Tamil Nadu, 2015 -16: India. Mumbai: IIPS; 2016. Retrieved from: http://rchiips.org/NFHS/pdf/NFHS4/TN_FactSheet.pdf

**Fig. 1 (abstract A64).**
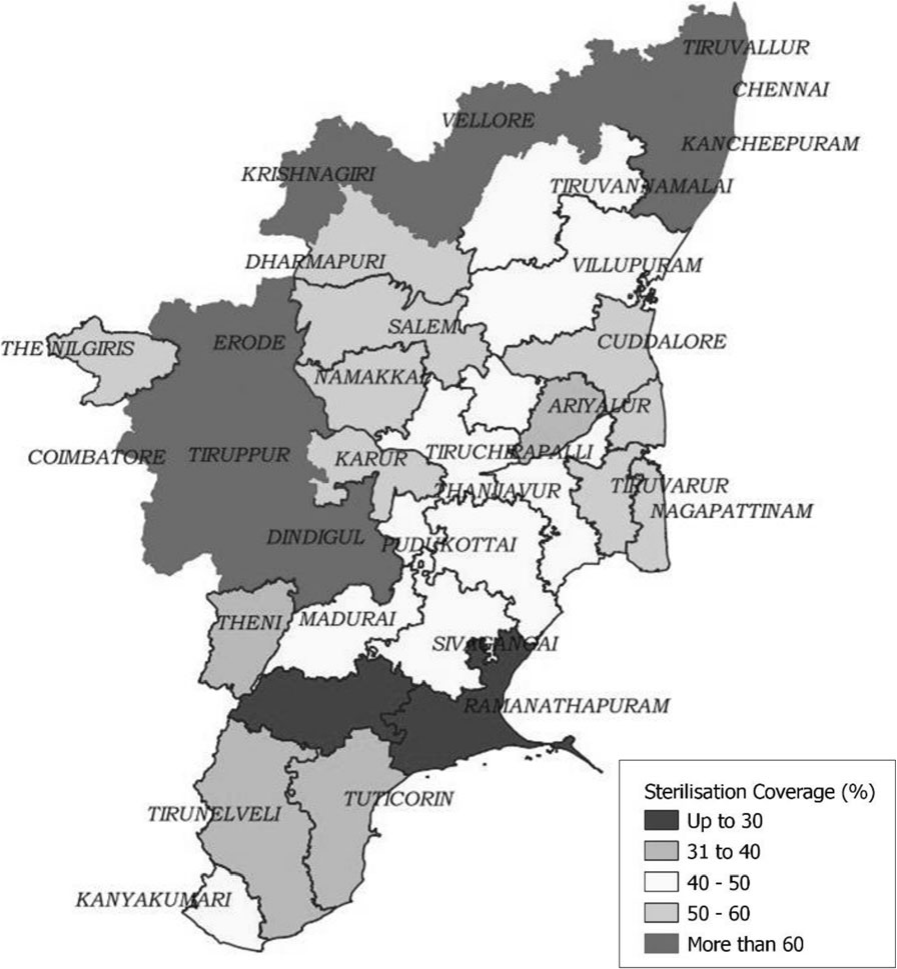
Coverage of any method of sterilisation in Tamil Nadu, NFHS 4, 2015-16

## A65 Description and Evaluation of Influenza Like Illness (ILI) Component of Daily Disease Surveillance System in Coimbatore District Tamil Nadu, India 2018-19

### Ramanathan Sarangapani^1^, Jerome Wesley Vivian Thangaraj^2^

#### ^1^MPH Scholar 11^th^ Cohort, ICMR-SPH, NIE, Chennai, Tamil Nadu, India; ^2^Scientist, ICMR-National Institute of Epidemiology, Chennai, Tamil Nadu, India

##### **Correspondence:** Ramanathan Sarangapani (Srnathan16@gmail.com)

**Background**

Influenza A(H1N1) causes substantial morbidity and mortality. Global annual burden of the disease is around 3 to 5 million cases and reported 0.29 to 0.69 million deaths [1]. India witnessed first Influenza pandemic in the year 2009 and thereafter seasonal outbreaks were reported in 2015 and 2017. 30,000 laboratory confirmed cases and 1700 deaths were reported in India in 2015.Tamil Nadu, a state in India reported 2212 cases and 43 deaths in 2018[2] and Coimbatore, a district in Tamil Nadu reported 2nd highest number of cases and deaths in 2018

Influenza Like Illness (ILI) cases are reported through Integrated Disease Surveillance Programme (IDSP) from a network of sentinel hospitals and accredited laboratories across various states of India as part of routine surveillance system [3]. However, in the state of Tamil Nadu, (IDSP) [3] being a weekly reporting system was supplemented by a Daily Disease Surveillance System(DDSS). In Coimbatore, 21 Government hospitals and 27 private hospitals were part of the DDSS. Of these, only 3 Government hospitals and 20 Private hospitals were mandated to report ILI. ILI component of the DDSS has not been evaluated since its inception. Hence, we propose to evaluate ILI component of the DDSS, in Coimbatore district, Tamil Nadu, India.

**Methods**

A cross sectional study was conducted from 2018 to 19. The study participants included clinicians 135/201, hospital managers 23/201, laboratory managers 5/201, lab technicians 17/201 and 21/201 stake holders in Office of Deputy Director of health services. We interviewed all these participants from 3 government and 25 private health facilities. We used structured questionnaires and information was abstracted from records using abstraction form. Reviewed all the relevant documents like registers, records, reports, reporting formats, case definition used at various levels.

The objective was to describe the ILI component of daily disease surveillance system in Coimbatore and evaluate ILI component of daily disease surveillance system by attributes such as simplicity, acceptability, completeness, timeliness and positive predictive value in Coimbatore, Tamil Nadu 2018-2019.

Daily Disease Surveillance System has a separate daily reporting format for ILI component. Suspected ILI cases visiting the surveillance hospitals and confirmed ILI cases from laboratories in the last 24 hours are reported through field staffs by emails and hard copies to District Surveillance Unit (DSU). Reports are then entered in an excel sheet. Final data is sent electronically to state surveillance unit daily before 3pm by the District Surveillance Unit. List of cases shared with concerned local authorities to initiate field control measures. District reports including place and time analysis were prepared on daily, weekly, monthly, quarterly and yearly basis. Feedback were shared in the monthly review meetings as well.

Evaluation of ILI component of DSSS [4,5] was carried out by looking at key attributes completeness which was defined as the proportion of completeness of variables in the form, registers and reports reviewed in the District surveillance unit. Timeliness was defined as proportion of reports submitted by the reporting units on time. Acceptability was proportion of reporting units willing to participate and submit complete reports every day on time. Positive predictive value was defined as proportion of health care facilities that detected the cases correctly. Proportion of health care providers using the case definition to identify the case. This information was obtained by reviewing hospital records. The data was entered in MS Excel and analysed using Epi-info software. Proportion of all the indicators calculated using EPIINFO version 7.0. The study was approved by the NIE Institutional Ethics Committee.

**Results**

Out of 3087 forms 2056(67%) forms had all variables filled. Out of 28 reporting units, 12 (43%) reporting units submitted the form with all variables filled. Out of 28 reporting units 24 (86%) received feedback from DSU. Of the 28 reporting units, 27 (97%) submitted the report on time. Acceptability was 100% as all reporting units (28 out of 28) all the clinicians, hospital Managers (180 out of 180) were willing to be part of the reporting system. All 201 participants (100%) found no difficulty in carrying out the activity and reporting. The data manager and the data entry operator found no difficulties to do the online reporting. For Positive Predictive Value, out of 23 hospitals,18 (78%) hospitals had correctly captured the cases. Of the 158 clinicians,154(97%) were aware of case definition. Of the 158 clinicians,154(97%) were using the case definition to identify cases.

**Conclusion**

ILI component of DDSS coverage was only 48 percent (23/48) of the reporting facility of DDSS. All the health facilities should be included in reporting on ILI. Nearly all the clinicians, Managers, Lab technicians and surveillance health inspectors prefer to report the ILI component of DDSS reporting system. Though all the health facilities submitted the reports on time, only 43% submitted the report with all the variables duly filled. Date of admission, name of the referral hospital, date of initiation of treatment were the commonly missing variables in reporting form which must be filled-in. Majority of the clinicians were aware of the case definition. Current ILI component of Daily disease surveillance system in Coimbatore district was timely and incomplete.

**References**

1. Garske T, Legrand J, Donnelly CA, Ward H, Cauchemez S, Fraser C, et al. Assessing the severity of the novel influenza A/H1N1 pandemic. BMJ [Internet]. 2009 Jul 14 [cited 2020 Jun 22];339. Available from: https://www.bmj.com/content/339/bmj.b2840

2. Murhekar M, Mehendale S. The 2015 influenza A (H1N1) pdm09 outbreak in India. Indian J Med Res. 2016 Jun;143(6):821–3.

3. Home :: Integrated Disease Surveillance Programme(IDSP) [Internet]. [cited 2020 Jun 22]. Available from: https://idsp.nic.in/

4. Updated Guidelines for Evaluating Public Health Surveillance Systems [Internet]. [cited 2020 Jun 22]. Available from: https://www.cdc.gov/mmwr/preview/mmwrhtml/rr5013a1.htm

5. European Centre for Disease Prevention and Control. Data quality monitoring and surveillance system evaluation: a handbook of methods and applications. [Internet]. Stockholm: ECDC; 2014 [cited 2020 Jun 22]. Available from: http://bookshop.europa.eu/uri?target=EUB:NOTICE:TQ0414829:EN:HTML

## A66 Causes of non-compliance to the continuum of care among patients screened for hypertension or diabetes, Sundergarh district, Odisha, 2016

### Biswa Prakash Dutta^1^, S P Padhi^3^, D Bachanni^4^, Sameer Sodha^2^, Rajesh Yadav^2^, S Gupta^1^, Mohamed Shaukat^4^, PKB Pattnaik^3^, C S Aggarwal^1^, S Venkatesh^1^

#### ^1^National Centre for Disease Control, Delhi, India; ^2^Centers for Disease Control and Prevention, Delhi, India; ^3^Department of Health & Family Welfare, Government of Odisha, India; ^4^Department of Health & Family Welfare, Government of India, Delhi, India

##### **Correspondence:** Biswa Prakash Dutta (dutta_bp@rediffmail.com)

**Background**

Non-communicable diseases in 2015 contributed to 58.7 lakh deaths in India, with Hypertension (HTN) and Diabetes Mellitus (DM) being among the leading causes [1]. The overall prevalence of diabetes, hypertension is 62.47and 159.46 respectively per 1000 population [2]*.* In 2011, India implemented the National Programme for Prevention and Control of Cancer, Diabetes, Cardiovascular disease and Stroke (NPCDCS) for screening, referral, diagnosis, and management of HTN and DM with the existing health infrastructure. The lack of compliance with long-term medication for chronic conditions and the resulting consequence of compromise in health benefits has raised global concerns [3]. In addition, it has also been identified as the predominant reason for the failure of medical therapy and disease progression.

To determine the extent of non-compliance to the continuum of care provided to the patients (under NPCDCS) with hypertension or diabetes mellitus and to identify the factors contributing to non-compliance, we conducted a cross-sectional study among the patients at the community level in the Sundargarh district of Odisha, India in 2016

**Methods**

We defined a non-compliant to referral as a case-patient, screened positive for HTN or DM, skipped referral for disease confirmation, non-compliant to treatment as a case-patient diagnosed as HTN or DM failed to consume <80% of medicines in last month as per pill count or prescription verification. Non-Compliant follow-up is defined as a case-patient who had no follow-up in the previous six months. For study purposes, we described each health sub center as one cluster. We selected 30 clusters by probability proportional to the size of the population in the Sundergarh district. We randomly selected seven participants (age >30 years) from every 30 clusters from the list of screened positives for HTN or DM screened during April 2014-March 2016. We used a pretested, semi-structured questionnaire for the data collection from the study participants. The study questionnaire captured information on demographics, socioeconomic status, screening, referral, treatment, follow-up, counseling received under NPCDCS, and reasons for not availing the services under NPCDCS. To identify causes of non-referral after initial screening, we interviewed the health worker (who had done the screening) of the respective health sub center using a structured questionnaire. The data collected was compiled and analyzed using Epi Info version 7.2 after removing all study participants' personal identifiers, and proportions were calculated with a 95% confidence interval (CI).

**Results**

Among 209 (99%) screened positive for HTN or DM; median age was 50 years (range: 31-90 years); 131 (63%) were females, 143 (68%, 95% Confidence Interval: 62%-75%) were referred as shown in (Fig1).

Among 143 referred, 28 (20%) not visited the referral centre. The reported reasons for not visiting the referral centre were 83% felt healthy, 21% had no escort, 17% reported accessibility difficulties, and 10% feared diagnosis. Among 66 (32%) not referred by health workers after initial screening, 6 attended the referral center independently. Of the121 visiting referral centre (115 referred and six self-referred), 104 (86%) were diagnosed with HTN or DM and placed on medication. Among the 104 put on treatment, 80 (77%) purchased medicines, and 61%* were counseled for treatment duration, 23%* for complications, and 23%* for follow-up. Forty-nine (47%) were non-compliant to medications either due to a better sense of health (57%)*, or cost of drugs (45%)*, unable to take the medication in their busy schedules (12%)*, and inconsistent medical supply (10%)*. Sixty-five (62%) were non-compliant to follow-up as they felt healthy (60%)*, lacked a caregiver (14%)*, did not see the follow up to be necessary (14%)*, and unable to make an appointment in their busy schedule (11%). The major reported cause of non-referral after the initial screening was lack of knowledge on referral (92%) and lack of training (6%)

**Conclusions:** This study identifies the reasons for non-compliance to referral medication and follow up examination for diabetes or hypertension even in the availability of services and recommended the need to put efforts into providing patient centric services. Patient counseling, identifying community caregivers, and ensuring a free consistent supply of medicines could improve the situation. This finding was in line with the findings of the MEDLINE database literature search of 127 articles published between January 1, 1990, and March 31, 2010 [4], which shows that many patients with chronic illnesses have difficulty adhering to their recommended medication regimen

*- All data are mutually exclusive.

**Reference**

1. National Health Portal of India https://www.nhp.gov.in/healthlyliving/ncd2019. Accessed June 20, 2020

2. Annual report (Indian council for Medical Research, 2006. https://main.icmr.nic.in/sites/default/files/annual_repoorts/Annual-Report_2005-2006_ICMR_Headquarters-Delhi-English.pdf. Accessed June 20, 2020

3. World Health Organization. Non-communicable diseases fact sheet, http://www.who.int/mediacentre/factsheets/fs355/en/. Accessed June 20, 2020

4. Brown, Marie T., and Jennifer K. Bussell. 'Medication Adherence: WHO Cares?' Mayo Clinic Proceedings 86, no. 4 (April 2011): 304–14. 10.4065/mcp.2010.0575. Accessed June 20, 2020

**Fig. 1 (abstract A66).**
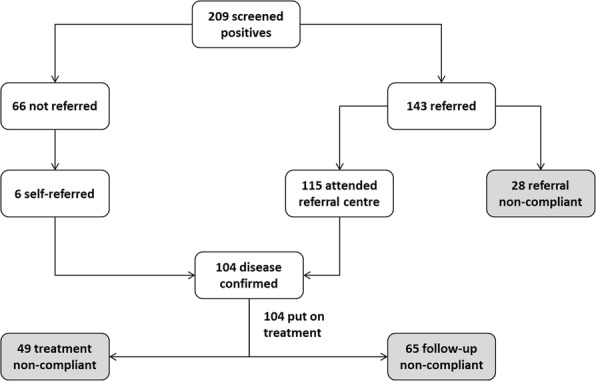
Distribution of non-compliance among screened positives for hypertension and diabetes, Sundargarh, Odisha 2016

## A67 Descriptive epidemiology of dengue infection, Madurai District, Tamil Nadu, 2013-2017

### Yazhini Madurapandian^1^, Parasuraman Ganeshkumar^2^, Polani Rubeshkumar^2^

#### ^1^Department of Public Health and Preventive Medicine, Govt. of Tamil Nadu; ^2^ICMR National Institute of Epidemiology, Chennai India

##### **Correspondence:** Parasuraman Ganeshkumar (ganeshkumardr@gmail.com)

**Background**

Dengue fever is endemic in more than 100 countries in the WHO regions of Africa, the Americas, the Eastern Mediterranean, South-East Asia, and the Western Pacific. The America, South-East Asia, and Western Pacific regions are the most seriously affected [1]. In India, dengue is endemic in almost all states and is the leading cause of hospitalization [2]. Dengue fever had a predominant urban distribution a few decades earlier but is also reported from peri-urban and rural areas [2]. In the year 2015, According to the National Vector Borne Disease Control Programme (NVBDCP) of the government of India, maximum numbers of cases were reported from Delhi, followed by Punjab, Haryana, West Bengal, Gujarat, Karnataka, Maharashtra, Kerala, Tamil Nadu [3]. In 2017, Tamil Nadu contributed 12% of the dengue cases in India [3]. In this regard, we aimed to review the epidemiology of dengue infection in Madurai District, Tamil Nadu, 2013-17.

**Methods**

Madurai is one of the districts in central Tamil Nadu, with a projected population of around 3.5 million in 2017. The district has a population density of 823 per sq. km. It has seven taluks, 13 community blocks, and one municipal corporation. We conducted a cross-sectional study on reported dengue cases in 13 reporting units of the National Vector-borne Disease Control Program (NVBDCP) from 2013 to 2017 who were residents of Madurai District. We defined a case of dengue as the occurrence of fever and positive IgM antibody or NS1 antigen by Enzyme-linked immunosorbent assay (ELISA) among hospitalized case-patients. We checked the data quality and completeness. We computed the population of Madurai District during 2013-17 based on the census 2001 and 2011. We estimated the cumulative incidence of dengue infection per hundred thousand population. We described the incidence of dengue cases by month and year of reporting as Epicurve to describe time distribution and mapped incidence by blocks to describe place distribution using Epi-info maps. We tabulated incidence by age-group and sex to describe the person distribution.

**Results**

There were 6404 dengue cases reported between 2013-17, 3341 (52%) were male, and 3070 (48%) were between 15-44 years. The dengue cases reported between 2013 and 2017 were 738, 823, 399, 326, and 4118. Incidence of dengue varied between 31 per lakh population to 403 per one lakh population for 2013-2017. (Figure 1) The year 2016 recorded the lowest incidence of 31 per one lakh population, and the year 2017 recorded the highest incidence 403 per one lakh population. The dengue incidence was 71, 80, and 39 per one lakh population in 2013, 2014, and 2015 respectively.

During 2013-16, cases started increasing gradually from August and attained their peak during October and declined after that. In 2017, cases began to rise from June onwards, peaked during November, and rapidly declined in December. Throughout the study period, dengue incidence was higher among males, which ranged from 10 per one lakh to 130 per one lakh population. Incidence was higher among the age group 5-14 years during 2013-17 (ranged between 18 to 240 per one lakh population) except in 2015. In 2015, the incidence was higher among the age group under-5 years (33 per one lakh population). Dengue cases were reported consistently high in the blocks of Thiruparakundram, Chellampati, and Madurai west throughout the study period.

**Conclusion**

The dengue incidence increased around ten times in 2017, and the cases were reported during the fourth quarter of the calendar year. We recommended intensifying the dengue control activities during the last quarter of the calendar year.

**References**

1. Dengue and severe dengue [Internet]. [cited 2020 Oct 2]. Available from: https://www.who.int/news-room/fact-sheets/detail/dengue-and-severe-dengue

2. Ganeshkumar P, Murhekar MV, Poornima V, Saravanakumar V, Sukumaran K, Anandaselvasankar A, et al. Dengue infection in India: A systematic review and meta-analysis. PLOS Neglected Tropical Diseases. 2018 Jul 16;12(7):e0006618.

3. DENGUE/DHF SITUATION IN INDIA :: National Vector Borne Disease Control Programme (NVBDCP) [Internet]. [cited 2020 Oct 2]. Available from: https://nvbdcp.gov.in/index4.php?lang=1&level=0&linkid=431&lid=3715

**Fig. 1 (abstract A67).**
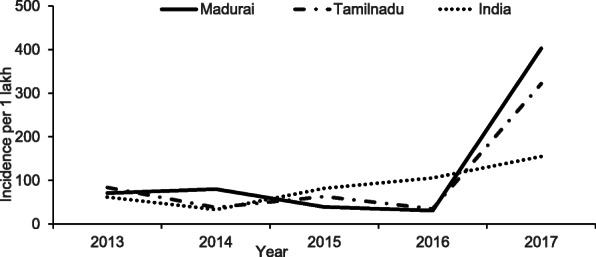
Incidence of Dengue in Madurai, Tamil Nadu, and India during 2013 – 2017

## A68 Evaluation of Swachh Bharat Mission (Urban) in Tirunelveli city, Tamil Nadu, India, 2014-2018

### Porchelvan Shanmugiah, Manickam Ponnaiah

#### ICMR – National Institute of Epidemiology, Chennai, India

##### **Correspondence:** Manickam Ponnaiah (manickamp@nieicmr.org.in)

**Background**

One billion people still practice open defecation globally, of which 59% reside in India [1]. Sixty percentage of the municipal solid waste collected here is dumped in landfill sites untreated [2]. Forty-five percent of the urban population is still without access to improved sanitation. It is noteworthy that 15% of the urban households in India practice open defecation [3]. India launched Swachh Bharat Mission (SBM) in 2014 to accelerate sustainable solid waste management and sanitation coverage [4]. The main objectives of SBM are to eliminate open defecation, eradicate manual scavenging, and invest in modern and scientific municipal solid waste management. Affecting the behavioral change regarding healthy sanitation practices, generating awareness about sanitation and its linkage with public health, and capacity building for ULBs (Urban Local Body) to create an enabling environment for the same are also listed as the major work areas of SBM. The National Family Health Survey (NFHS) – 4 reports showed that only 54 % of the population had improved sanitation access in Tirunelveli city.[5] In the nationwide survey for cleanliness assessment conducted by the Ministry of Housing and Urban Development (MoHUD), Swachh Survekshan -2019, Tirunelveli slipped to 238^th^ rank in 2019 from 178 in 2018. The present study was conducted to evaluate Swachh Bharat Mission (urban) activities in Tirunelveli city, Tamil Nadu, India, from 2014 to 2018.

**Methods:**

We developed a logic model to evaluate solid waste management practices, sanitary toilet construction, and awareness arms of the Swachh Bharat Mission with input, process, and output indicators. A cross-sectional study was conducted in the Tirunelveli Municipal Corporation during the May-August 2019 and collected data on solid waste management from 2014 to 2018. The sample size was calculated as 140 with an expected awareness among sanitary workers as 90%, keeping absolute precision as 5%, confidence interval as 95% & non-response as 10%. We did a simple random sampling of the sanitary workers for evaluation of knowledge on solid waste management. We calculated the sample size as 275, with 82% houses preferring to segregate waste, with a 5% absolute precision, 95% confidence interval & 10% non-response. Households were selected in a simple random fashion for the assessment of awareness on SBM activities. We selected five households randomly from each of the available 55 wards. From within each selected household, the member primarily involved in segregation and handling waste was chosen for the interview. We did a review of records using data abstraction form. Data on human resources availability, waste segregation and processing, materials and funds for sanitary latrine construction, and awareness activities done on the programme were collected. We did a community-based survey of sanitary workers for knowledge on solid waste management using a semi-structured questionnaire. We also did a community-based study of residents for awareness of SBM activities using a semi-structured questionnaire. We calculated input, process, and output indicators for the three arms as proportions using appropriate denominators. We also computed population awareness of SBM initiatives, their perception, willingness, and participation in SBM initiatives as proportions.

**Results:**

We reviewed records available at the Municipal commissioner's office, 17 sanitary unit offices, compost yard, and 41 Micro Composting Centers (MCC) of Tirunelveli city. We interviewed 256 residents from 55 wards and 140 hygienic workers. Sanitary workers in position against norms were 77% (1116/1450), and vehicles for primary collection of solid waste functional were 85% (524/619). Among MCC, 75% (31/41) were fully equipped for the processing of bio-degradable wastes. Household toilets constructed against the target were 90% (3746/4150) but incentives to construct the toilet were provided to only 71% (2658/3746). All new community toilets were completed as per target, and 85% (137/161) of the total community toilets available were functional. Fund utilization for awareness activities on SBM initiatives in the community was only 31%. Households aware of activities done under SBM were 88% (226/256). Household segregation of waste is 65% (165/256), contrary to records claim of 85%. Households aware of health impacts due to non-segregation of wastes were 89% (225/256), and 98% (248/256) residents interviewed were aware that open defecation is harmful to health. Among the interviewed households, 90% (231/256) had a functional toilet at the household level, 9% (24/256) were using community sanitary complex, and <1% (1/256) practiced open defecation. Among the sanitary workers interviewed, 98% (137/140) were trained in solid waste management. Sanitary workers who felt adequate sanitary equipment is not available were 73% (104/140). Sanitary workers not using Personal Protective Equipment (PPE) while the waste collection was 41% (57/140). Sanitary workers with adequate knowledge on the segregation of waste at the household level were 97% (136/140).

**Conclusion:**

Resources available for solid waste management are inadequate. Toilet construction and functionality were adequate, which may be the reason for the practice of open defecation being low. The utilization of funds for awareness activities was low, but awareness of SBM activities was high. There was a mismatch in household segregation of waste between record claim and field. Though sanitary workers were trained adequately, usage of PPEs among them during waste collection was low. Though most of the mission initiatives are functioning adequately, there is scope for improvement. Adequate workforce and equipment may help in improving solid waste management. Awareness activities on household segregation of wastes can be improved by adequate utilization of funds. Incentives to sanitary workers for IEC activities can be given. Sanitary workers can be educated on the importance of PPE usage. Record and field mismatch in waste segregation shall be studied further.

**References**

1. UNICEF. Progress on Sanitation and Drinking Water: 2015 Update and MDG Assessment. 2015. https://www.unicef.org/reports/progress-sanitation-and-drinking-water. Accessed 20 Sep 2020.

2. Central Pollution Control Board. Guidelines and Check-list for evaluation of MSW Landfills proposals with Information on existing landfills. 2008. http://www.indiaenvironmentportal.org.in/files/MSW-REPORT.pdf. Accessed 9 Dec 2019

3. Tamil Nadu Pollution Control Board. Annual reports and accounts. 2016-17. https://tnpcb.gov.in/pdf/Annual_Report/Annual_ReportEng16_17.pdf. Accessed on 1 Oct 2020.

4. Ministry of Housing & Urban Affairs, Government of India. Swachh Bharat Mission. http://swachhbharaturban.gov.in/writereaddata/SBM_Guideline.pdf. Accessed 1 Oct 2020.

5. International Institute for Population Sciences. National Family Health Survey- 4 India. 2015-16. http://rchiips.org/nfhs/NFHS-4Reports/India.pdf. Accessed 1 Oct 2020.

## A69 Description and evaluation of High-risk pregnancy detection and management of public facilities in Tiruchirappalli district, Tamil Nadu, India, 2016

### Sathishkumar Ramadass^1^, Parasuraman Ganeshkumar^2^, Pattabi Kamaraj^2^

#### ^1^Directorate of Public Health and Preventive Medicine, Tamil Nadu, India; ^2^ICMR-National Institute of Epidemiology, Chennai, Tamil Nadu, India

##### **Correspondence:** Parasuraman Ganeshkumar (ganeshkumardr@gmail.com)

**Introduction**

Maternal death is the death of a woman while pregnant or within 42 days of pregnancy termination, irrespective of the duration and site of the pregnancy, from any cause related to or aggravated by the pregnancy or its management but not from accidental or incidental causes [1]. Sustainable Development Goals (SDG) 3.1 denotes the reduction of global maternal mortality ratio (MMR) to < 70 per 100,000 live births [2] by 2030, In India, the maternal mortality ratio was 113 per 100 000 live births, as compared to Tamil Nadu, was 60 per 100,000 live births [3]. As per the State reports, in Tiruchirappalli, a central district of Tamil Nadu, MMR was 84 per 100,000 live births, higher than the State MMR. A high-risk pregnancy is defined as pregnancy complicated by a factor or factors that adversely affect the pregnancy outcome-maternal or perinatal or both. Worldwide, 20 million women have high-risk pregnancies, and more than 800 die daily from perinatal conditions. Among the mothers seen in the antenatal period, 10-30% of mothers have been classified as high-risk. While assessing the risk of any pregnancy, some medical history like age, parity, social class, and past obstetric history should be considered.

High-risk pregnancies contribute 70- 80% of maternal morbidity and mortality. Treating high-risk pregnancies with adequate care will give a significant reduction in maternal mortality and morbidity. In Tamil Nadu, the proportion of high-risk pregnancies among antenatal mothers is 10-30%. Tamil Nadu issued guidelines for early detection and management of high-risk pregnancy and set to achieve a maternal mortality ratio less than 45/100,000 live births by 2017 [4]. Despite measures, the High-risk pregnancy percentage of the Tiruchirappalli health unit district was static. Hence, we envisaged evaluating the status of implementing high-risk pregnancy guidelines in the Tiruchirappalli health unit district of Tamil Nadu, India.

**Methods**

We conducted a cross-sectional study among high-risk mothers and health care providers for three months with a reference period of registered mothers between October and March 2015. We reviewed the guidelines of the Government of Tamil Nadu and documents from selected Primary healthcare facilities and interviewed the healthcare providers. With an anticipated prevalence of high-risk pregnancy as 20% in the population size of 20,000, absolute precision level of 7% and design effect 2, confidence interval- 95%, calculated sample size was 250 antenatal mothers. We adopted the Cluster sampling technique where the primary sampling unit was health subcentres. We used probability proportion to the population of each HSC and linear systematic sampling (PPSLSS) technique for cluster selection.

We selected 25 clusters under which to achieve 250 mothers, and we selected ten mothers more than 20 weeks of gestation per cluster. We selected the healthcare providers from the corresponding Primary Health Centre (PHC) of the selected Health subcentres (HSC) where the selected mothers were registered. Therefore, we selected 50 medical officers, 75 staff nurses, 25 Auxiliary nursing midwives, 25 village health nurses by cluster sampling. We interviewed Pregnant women through a structured questionnaire regarding Pregnancy status & awareness on high-risk pregnancy and Interviewed health care providers through a structured questionnaire to assess their knowledge on high-risk pregnancy. Review of records by data abstraction form for service delivery and facility survey by checklist for infrastructure, human resource, equipment, and logistics were completed. We obtained approval from the Ethics committee of ICMR-National Institute of Epidemiology, Chennai, India. We entered the data in Epi Info version 7. We computed proportions for the input, process, and output indicators.

**Results**

Among sanctioned posts, medical officers in position were 54 (75%), and staff nurses were 73 (85%), Auxiliary nursing midwives were 15 (60%), Laboratory technicians were 16 (64%), and Village health nurses were 23 (92%). Of the 54, 46(85%) were trained in basic emergency obstetric and neonatal care (BEmONC). Of the 73, 24 (33%) staff nurses were trained in skilled birth attendants, and 14 (93%) Auxiliary nursing midwives were trained in skilled birth attendants. Of the 16 interviewed laboratory technicians, 8(50%) were trained in refresher training on antenatal screening, and all village health nurses were trained in Maternal and child health.

During the facility survey compared to the guidelines, 12 (48%) primary health centers had a mother examination room, and 4 (16%) primary health centers had a blood storage unit. Twelve (49%) primary health centers had maternal child health registers, and all primary health centers were having a weighing machine, stadiometer, BP apparatus, stethoscope, laboratory reagents.

We included 11,039 pregnant women registered in PHCs and 791 from HSCs. Of them, 11023 (99%) and all 791 (100%) mothers’ blood pressure were measured in PHCs and HSCs, respectively. In PHCs, hemoglobin estimation, glucose challenge test, Urine albumin examination, and ultrasonogram examination were done in 9627 (87%), 4580 (41%), 9798 (88%), and 4929(44%) pregnant women, respectively.

The following high-risk pregnancies were identified during the study period (Table 1), anemia in 1278 (13%), Pregnancy-induced hypertension (PIH) in 28 (0.3%), Gestational diabetes mellitus (GDM) in 15 (0.3%), and Foetal anomalies identified in 3 (0.06%). Of the 1278 mothers with anemia complicating pregnancy, 628 (49%) were started on parenteral Iron sucrose therapy, 19 (5%) started on blood transfusion, and 44 (3%) were referred to higher centers. None of the GDM mothers was treated at PHCs, and 12 (80%) were referred to higher centers. 22 (78%) pregnant women with PIH were started on therapy with labetalol at PHCs, and 12 (43%) of them were referred to higher centers.

**Conclusion**

Vacancies of key healthcare providers, lack of functional equipment required for high-risk mothers seem to be the area for improvement in primary health care facilities. We recommend equipping the trained workforce with essential equipment at primary healthcare facilities for early detection and adequate management of high-risk pregnancies.

**References**

1. World Health Organization. Maternal mortality. 2019. Available at: [http://www.who.int/mediacentre/factsheets/fs348/en/] Accessed August 29, 2020

2. United Nations Development Programme. Sustainable Developmental Goals. Goal 3: Good health and well-being [Internet]. Available from:[https://www.undp.org/content/undp/en/home/sustainable-development-goals/goal-3-good-health-and-well-being.html#targets] Accessed August 29, 2020

3. Ministry of Home Affairs, Govt. of India. 2020. Sample Registration System: Special Bulletin on Maternal Mortality in India 2016-18 [Internet]. Office of the Registrar General, Ministry of Home Affairs, Govt. of India. Available from: [https://censusindia.gov.in/vital_statistics/SRS_Bulletins/MMR%20Bulletin%202016-18.pdf] accessed August 29, 2020.

4. National Health Mission, State Health Society. Tamil Nadu, India. 2014. Circular 5/2014 Reduction of MMR Observation of High Risk Mothers - Guidelines. [Internet]. Available from: [https://www.nhm.tn.gov.in/sites/default/files/2019-12/Circular_5.pdf] accessed August 29, 2020.

**Table 1 (abstract A69).** Evaluation of High-risk pregnancy detection & Management among AN mother of selected PHCs, Tiruchirappalli, 2016

Level	Output	Indicators	n	N	%
PHC	High-risk pregnancy Identified	Anaemia (Moderate)	1278	9627	13
		Pregnancy-induced hypertension (PIH)	28	11023	0.3
		Gestational diabetes mellitus (GDM)	15	4580	0.3
		Foetal anomalies identified	3	4929	0.06
	Treatment started	Iron sucrose injection to anaemic Mothers	628	1278	49
		Blood transfusion to anaemic mothers	19	628	5
		Insulin to GDM Mothers	0	15	0
		Labetalol to PIH Mothers	22	28	78
	Referred to CEmONC Centres	Anaemic mothers referred	44	1278	3
		PIH Mothers referred	12	28	43
		GDM Mothers referred	12	15	80
HSC	High-risk pregnancy followup	Anaemia (Moderate)	144	791	18
		Pregnancy-induced hypertension (PIH)	15	791	2
		Gestational diabetes mellitus (GDM)	7	791	0.8
	Treatment follow up	Iron and folic acid tablets to anaemic mothers	144	144	100
	Referred to CEmONC	Anaemic mothers referred	21	144	15
		PIH Mothers referred	12	15	80
		GDM Mothers referred	2	7	29

## A70 Determinants of Place of childbirth in Chamba District, Himachal Pradesh, India

### Jalam Singh Bharadwaj^1^, Parasuraman Ganeshkumar^2^, Kamaraj Pattabi^2^

#### ^1^Directorate of Health Services, Himachal Pradesh, India; ^2^ICMR-National Institute of Epidemiology, Chennai, Tamil Nadu, India

##### **Correspondence:** Parasuraman Ganeshkumar (ganeshkumardr@gmail.com)

**Background**

Maternal mortality reduction is a priority under “Goal 3: Ensure healthy lives and promote well-being for all at all ages” in the new Sustainable Development Goals (SDGs) [1]. Safe delivery care is vital for both maternal and perinatal health outcomes, and skilled attendance of birth at the institution is the central goal of safe motherhood and child survival. Institutional delivery of babies by the mother’s help reduce the risk of complications that may cause death or illness to mother and child [2]. Place of delivery has been influenced by cultural, social practices, economic factors [3]. As per the NFHS-4 report, Himachal Pradesh institutional births were 76% [4], and in Chamba district, it was 56% [5]. The National Rural Health Mission (NRHM) and now the National Health Mission was implemented in India with the main focus to strengthen rural health services. Since 2005, public health institutions are being strengthened to increase the utilization of it and so that basic maternal care would be provided closer to the population. Despite these measures, it was unclear the reasons for poor institutional births at Chamba district of Himachal Pradesh. In the background of the paucity of studies on the decision-making process with regards to the place of delivery, the present study is proposed to delineate the decision-making process with regards to the place of delivery and to identify the factors for low utilization of health facilities for institutional deliveries. We intended to identify the determinants of place of delivery in the selected villages of Chamba district for the deliveries that occurred between June. 2017 and November 2017.

**Methods**

An unmatched case-control study was conducted in Chamba district between December 2017 and April 2018 in seven health Blocks where 7500 deliveries take place annually, with the percentage of institutional deliveries being 58%. Women who gave birth at home between June 2017 and November.2017 were considered as cases, and those who gave birth at health institutions within the same period were categorized as the controls. We included all the mothers who delivered during the period between June 2017 and November 2017 in the Chamba district. This study did not consider women who had a complication during pregnancy and pertained to only normal deliveries. This consideration was done to have a homogeneous sample as women in the above-mentioned category would have had a different experience of care. The proportion of non-utilization of Govt. vehicle for transportation among institutional deliveries in Chamba district, which is 72%, which was used as the exposure variable. For odds Ratio of 2.5 with 95% CI and power of 80% with a ratio of 1:1, we needed 135 home deliveries and 135 institutional deliveries, and after 10% of non-response, we required 150 birth deliveries in each group for our study. By using simple random sampling, 150 home deliveries were selected with the proportional allocation in five blocks of the Chamba district. In each of the home deliveries selected in the village, the same numbers of institutional deliveries were selected from that village or the adjacent village. We used a structured, pre-coded questionnaire to collect the data on the demographic characteristics of the respondent, socio-economic status of the household, and obstetric characteristics of the participant mothers. Supervision was carried out by three supervisors and the principal investigator. Data were collected by the field assistants and periodically supervised by the three supervisors and investigator himself. The data were entered and analyzed using EPI info (version 7.2).

**Result**

Of the included 300 mothers, 150 were cases who delivered at home and 148 controls who delivered at the institution, accepted to participate in the study giving a response rate of 99.3%. The mean age of mothers among cases was 24.6 ±3.3 years and among control 24.3±3.6 years, mean age of marriage among cases was 20.7±1.5 years and among control 20.8±1.6 years. Median income among cases and control was INR 5000 with IQR of cases (4000-10000) and for controls also INR 5000 with IQR (3000-15000). Median family members among cases were 6 (IQR: 5-7) and for controls also 6 (IQR: 6-7). The majority of the cases (98.7%), as well as controls (95.9%), were from rural areas. Among cases, 52% were not having access to the motorable road to the health facility as compared to 41.2% of controls. Nearly 58% of controls accessed health facilities on foot, and nearly 30% and 23% of control mothers accessed health facilities by private and 108 ambulance respectively. Both case and control mothers reported the average time taken to reach the health facility during antenatal visits was 20 minutes. The odds of home delivery among housewives was 4.7 times (Table 1) higher than the working mothers with an adjusted odds ratio of 4.5 with 95% CI (1.9-10.4), Odds of home deliveries among multipara was 6.3 times higher than the primipara with an adjusted odds ratio of 6.3 and 95% CI (3.7-10.7). The odds of home delivery among decisions taken by the joint family regarding deliveries was 1.7 times higher than the decision taken by self (mothers) on deliveries with an adjusted odds ratio of 1.7 and 95% CI (1-2.9). Among cases, 46% opined that home delivery was easy and convenient, 46.7% cases reasoned cost at the hospital, 35.3% as lack of transport, 38.7% family preference were the reasons for preferences to home delivery. Two out of 150 mothers who delivered at home reported following unsafe delivery practices. Odds of home delivery were not affected by the person accompanied during ANC, place of delivery, and awareness related to the free antenatal checkup scheme. Both cases and controls reported hemorrhage, pre-eclampsia, and prolonged labor as commonest obstetric complications.

**Conclusion**

A mother being multiparous, unemployed, and the decision of the family members influenced the place of delivery as home. We recommend identifying the high-risk mothers based on multiparity, unemployment, lack of self-decision making of the place of delivery to encourage institutional delivery by accredited social health activists (ASHA’s) and community-based educational activities on institutional deliveries.

**References**

1. United Nations Development Program. Sustainable Developmental Goals: Goal 3: Good health and well-being [https://www.undp.org/content/undp/en/home/sustainable-development-goals/goal-3-good-health-and-well-being.html#targets]. Accessed 20 August 2020.

2. Campbell OM, Graham WJ, on behalf of The Lancet Maternal Survival Series steering group: Strategies for reducing maternal mortality: getting on with what works. The Lancet. 2006, 368 (9543): 1284-1299. 10.1016/S0140-6736(06)69381-1.

3. Thind A, Mohani A, Banerjee K, Hagig F. Where to deliver? Analysis of choice of delivery location from a national survey in India. BMC Public Health. 2008;8:29

4. National Family Health Survey -4. 2015-16. State fact sheet, Himachal Pradesh, India. International institute for population sciences, Mumbai; [http://rchiips.org/nfhs/pdf/NFHS4/HP_FactSheet.pdf]. Accessed 20 August 2020]. Accessed 20 August 2020.

5. National Family Health Survey -4. 2015-16. District fact sheet, Chamba, Himachal Pradesh, India. International institute for population sciences, Mumbai; [http://rchiips.org/nfhs/FCTS/HP/HP_Factsheet_23_Chamba.pdf]. Accessed 20 August 2020.

**Table 1 (abstract A70).** Predictors of home delivery in Chamba district, Himachal Pradesh, 2017-18

Characteristics	Categories	Unadjusted odds ratio	95 % CI	Adjusted odds ratio	95% CI
Maternal Occupation	Working	1.0 (ref)		1.0 (ref)	
	Unemployed	4.7	2.1 - 10.1	4.5	1.9 - 10.4
Parity	Primipara	1.0 (ref)		1.0 (ref)	
	Multipara	6.3	3.8 - 10.4	6.3	3.7- 10.7
Decision regarding place of delivery	Self	1.0 (ref)		1.0 (ref)	
	Jointly by Family	1.7	1.1- 2.8	1.7	1.0- 2.9

## A71 Blood pressure control among hypertension patients in the public-sector health facilities in four Indian states - India Hypertension Control Initiative, 2018

### Abhishek Kunwar^1^, Prabhdeep Kaur^2^, Meenakshi Sharma^3^, Raviteja Dharamsoth^4^, Saurabh Purohit^5^, Bipin Gopal^6^, Gurinder B Singh^7^, Fikru T Tullu^1^, Balram Bhargava^3^

#### ^**1**^WHO Country Office for India, New Delhi; ^2^ICMR-National Institute of Epidemiology, Chennai; ^3^Indian Council of Medical Research, New Delhi; ^4^State NCD Cell, Govt of Telangana, Hyderabad; ^5^State NCD Cell, Govt of Madhya Pradesh, Bhopal; ^6^State NCD Cell, Govt of Kerala, Thiruvananthapuram; ^7^State NCD Cell, Govt of Punjab, Chandigarh

##### **Correspondence:** Abhishek Kunwar (abhishekk@who.int)

**Background**

In India, it is estimated that at least one in four adults have hypertension, but only about 10% of them have their blood pressure (BP) under control. To strengthen hypertension management under the National Programme for Prevention and Control of Cancer, Diabetes, Cardiovascular Diseases and Stroke (NPCDCS)[1], the Government of India launched the India Hypertension Control Initiative (IHCI) to reduce cardiovascular diseases deaths by improving control of high blood pressure. The project aimed to implement five evidence-based strategies adapted from the WHO HEARTS package in primary and secondary health care facilities [2]. The strategies included drug and dose-specific protocol, streamlining drug procurement and distribution systems, patient-centric care, task sharing, and an information system to monitor the patient cohort. Blood pressure control was the most important indicator for monitoring the progress of the project. We estimated the BP control of hypertension patients registered in 2018 under IHCI in public sector health facilities of Punjab, Madhya Pradesh, Kerala, and Telangana.

**Methods**

We implemented the project in 21 districts in four states in India. All states identified and implemented a dose and drug-specific protocol. The drugs were procured through the state procurement agencies. The nurse enrolled all patients who had hypertension and visited any of the public sector facilities. The doctor prescribed medications according to the protocol. The blood pressure was measured during all follow-up visits, and drugs were issued for 30 days. The data was maintained in cards in three states, and Punjab had an app-based system to document the patient visits. We analyzed the data for patients who were enrolled between 1^st^ January-31^st^ December 2018. We analyzed three indicators proportion with BP control, the proportion with uncontrolled BP, and the proportion who missed visits based on the most recent visit between 1^st^ January – 31^st^ March 2019. Blood pressure control was defined as systolic BP of less than 140 mmHg and diastolic BP of less than 90 mmHg during the most recent visit between 1^st^ January-31^st^ March 2019. The missed visit was defined as a lack of any follow-up visit during the same period.

**Results**

We enrolled 179,618 patients in 765 public health facilities in 21 districts of 4 states, Punjab, Madhya Pradesh, Kerala, and Telangana, between 1 January-31 December 2018. Kerala registered nearly two-thirds of all patients. We enrolled in four types of health facilities, namely Primary health centers (494), Community health centers (186), and district/ sub-district hospitals (44). In Kerala, a category of Family health centers (41) also registered the patients. Overall, 36% had BP control, 31% had uncontrolled BP, and 33% did not visit the health facility in the first quarter of 2019 (Figure 1). The proportion of patients with BP control varied from 15% in Punjab to 65% in Telangana. The overall BP control was 43%, 30%, and 27% among patients treated in primary health centers (PHC), community health centers (CHC), and district/sub-district hospitals, respectively. The proportion of patients who missed visits was higher in hospitals (56%) than PHCs (25%).

**Conclusion**

The project demonstrated the feasibility of hypertension management in primary care settings using simple protocols. Blood pressure control was highly variable across states. This was due to the variable availability of human resources of drugs. The control was higher in the PHC as compared to higher facilities. Uncontrolled blood pressure for one-third of the patients might be due to lack of titration of drugs by physicians and lack of adherence by patients. The major challenge was the lack of follow-up visits for nearly one-third of the patients. We recommend enrolling patients in the primary care facilities closer to the home and transferring patients from higher to lower-level facilities. We should actively engage the outreach health workers to retrieve the patients who did not return for follow up and dispensing of drugs in the sub-centers or Health Wellness Centers.

**References**

**1. National Programme for Prevention and Control of Cancer, Diabetes, Cardiovascular Diseases and Stroke (NPCDCS)** [https://main.mohfw.gov.in/Major-Programmes/non-communicable-diseases-injury-trauma/Non-Communicable-Disease-II/National-Programme-for-Prevention-and-Control-of-Cancer-Diabetes-Cardiovascular-diseases-and-Stroke-NPCDCS]

**2. Cardiovascular disease -HEARTS technical package** [https://www.who.int/cardiovascular_diseases/hearts/en/]

**Fig. 1 (abstract A71).**
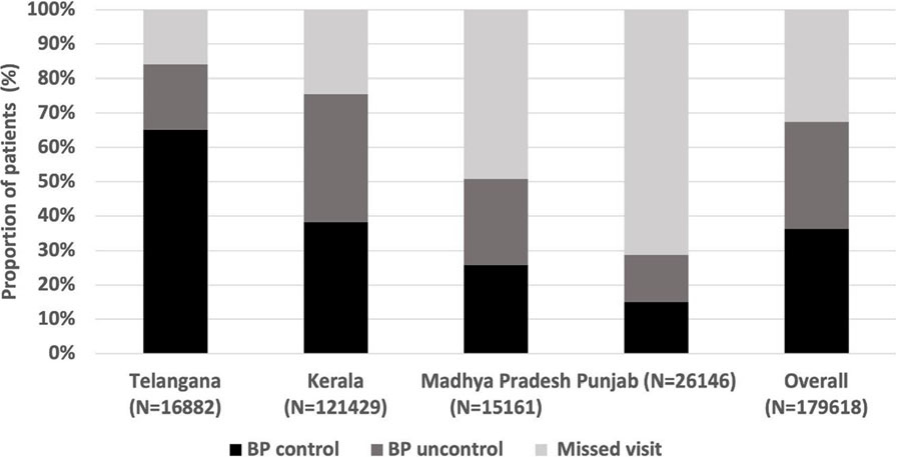
Proportion of patients with blood pressure control, uncontrolled blood pressure and missed visits during a most recent visit in Jan-Mar, 2019 among all registered patients in four states, India

## A72 Epidemiology of H1N1 Influenza in Hyderabad district, Telangana state, January 2015 – December 2017

### Sri Harsha Dasari Yadav, Prabhdeep Kaur

#### ICMR-National Institute of Epidemiology, Chennai

##### **Correspondence:** Sri Harsha Dasari Yadav (drharshadasari@gmail.com)

**Background**

H1N1 is an acute respiratory illness caused by a virus belonging to Orthomyxovirus family and subtype of Influenza A Virus. In 2009 H1N1 Influenza virus had two genes from flu viruses that normally circulate in pigs in Europe and Asia, plus avian, and human genes. This virus was named Swine Flu because laboratory testing showed that its gene segments were similar to influenza viruses that were most recently identified and circulating in pigs [1]. H1N1 is airborne, respiratory droplet infection that spreads when an infected person coughs or sneezes through contaminated hands and fomites. The symptoms vary from flu-like illness to acute respiratory failure, pneumonia sometimes requiring ventilatory support and can lead to death. The Pandemic of H1N1 occurred in April 2009, affecting more than 200 million population resulting in the first influenza pandemic of the 21^st^ century [2]. On 11^th^ June 2009, the World Health Organization (WHO) raised a pandemic alert phase 6, the highest alert indicating evidence of sustained human to human transmission and global spread of the virus [3]. Impact of disease is more in resource-limited environments due to decreased access to health care limited health care infrastructure and shortage of health care personnel, poor availability of vaccination, critical care support and high prevalence of comorbid conditions like malnutrition. During the pandemic in 2009, India reported 27,236 cases and 981 deaths. During 2015 epidemic India reported > 30,000 lab-confirmed cases,1700 deaths [4]. The first case of H1N1 in India during 2009 pandemic was reported in Hyderabad. In 2009, 6805 samples were tested, and 1329 positive cases reported. During 2015-2017, 2218 cases positive and 86 deaths were reported in Hyderabad district. The objective of this study was to describe the epidemiology of H1N1 influenza between 2015 – 2017, in terms of time place and person reported in government and private health facilities of Hyderabad district.

**Methods**

We analyzed the data on laboratory surveillance of H1N1 influenza in Hyderabad district. We included in the analysis, all the H1N1 influenza case-patients admitted in government and private health facilities in Hyderabad district of Telangana state between 2015-2017. We defined a confirmed case of H1N1 influenza as an episode of flu-like illness, tested positive for H1N1 by RT-PCR test. We collected the line list of H1N1 positive case-patients from the Institute of Preventive Medicine laboratory (IPM), the only lab in the district to conduct the RT-PCR Test. We abstracted the data into MS Excel and analyzed using Epi Info version 7.3.2.1. The inclusion criteria were all cases. We described the distribution of H1N1 influenza case-patients by time using a line graph, by plotting the number of case-patients against the reporting month. We also described the distribution by age, gender and place. We calculated the incidence of H1N1 per 100000 population for each year. We also estimated case fatality rate (CFR) by dividing the number of deaths due to H1N1 influenza by the total number of H1N1 influenza case-patients and multiplying by 100.

**Results**

In 2015, the total number of H1N1 influenza case-patients reported was 820, of which, 54% (691/129) were females. In 2016, 84 case-patients were reported, of which 62% (32/52) were females. The year 2017 witnessed 1308 cases, of which 50% (434/874) were males. About 82% of (1054/1291) H1N1 cases in 2015, 60% (27/52) in 2016 and 81% (713/874) cases in 2017 were reported between January-March (Figure 1). Moreover, 12% (150/1292) cases in 2015, 27% (14/52) in 2016, 24% (214/874) cases in 2017 were between August-October. Areas like King-Koti (11%, 247/2218) and Malakpet (8%, 169/2218) reported the highest number of cases. About 13% cases (293/2218) belonged to the 0-4-year age group (98% males), and 9% (191/2218) to ≥60 years age group (97% males). Incidence was 19/100000 population in 2015, 0.1/100000 in 2016 and 13/100000 in 2017. The Case Fatality Rate (CFR) was 3.8% in 2015, and 4.1% in 2017. No deaths were reported in 2016. CFR was highest (9%) among males between 45-59 age group in 2015.

**Conclusion**

Our findings indicate a seasonal trend for H1N1 with bimodal peaks. Incidence was highest in 2015 and CFR highest in 2017. We recommended vaccination for children <5 years and elderly ≥60 years, from December month and sensitization of people regarding hand hygiene during respiratory infections, and when travelling and attending gatherings.

**References**

1. World Health Organization. Influenza (seasonal) [Internet]. Available from: **https://www.who.int/westernpacific/health-topics/influenza-seasonal**.[Accessed 2021 Feb 9]

2. Al Hajjar S, McIntosh K. The first influenza pandemic of the 21st century. Annals of Saudi medicine. 2010 Jan;30(1):1-0.

3. WHO: World now at the start of 2009 influenza pandemic.2009. **https://www.who.int/mediacentre/news/statements/2009/h1n1_pandemic_phase6_20090611/en/**. [Accessed 2020 August 27].

4. Murhekar M, Mehendale S. The 2015 influenza A (H1N1) pdm09 outbreak in India. The Indian journal of medical research. 2016 Jun;143(6):82.

**Fig. 1 (abstract A72).**
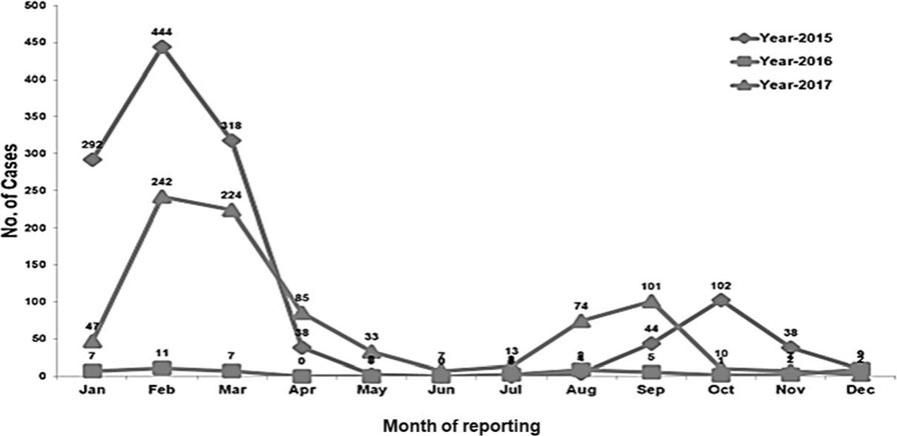
Distribution of H1N1 Influenza case-patients by month and year of reporting, Hyderabad district, Telangana, India, 2015 to 2017

## A73 Secondary data analysis of diabetes mellitus and hypertension among persons attending district hospital NCD clinics in 51 districts of Madhya Pradesh, India in 2017

### Saurabh Purohit^1^, Tapas Chakma^2^

#### ^1^Deputy Director, Directorate of Health Services, Government of Madhya Pradesh, India; ^2^Scientist 'G' (Director Grade), ICMR-National Institute for Research in Tribal Health(NIRTH), Jabalpur, Madhya Pradesh, India

##### **Correspondence:** Saurabh Purohit (saurabhpurohit81@gmail.com)

**Background**

Non-communicable diseases (NCDs) are a significant public health issue, responsible for 48% of healthy life years lost and 63% of all deaths worldwide. India is also experiencing rapid demographic and epidemiological transitions from communicable diseases to NCDs, causing significant disability, morbidity, and mortality in urban and rural populations across all socioeconomic status. By 2020, it will increase up to 73% deaths and 60% of the global disease burden. In India, NCDs contribute to 5.8 million deaths per year, almost 60% of total deaths. In India, diabetes mellitus (DM) and hypertension (HTN) are two major risk factors for NCDs, increasing morbidity and mortality. According to the Annual Health Survey (AHS) 2012-13, in Madhya Pradesh, the burden of these two major risk factors was, 7% for diabetes mellitus and 22% for hypertension [1]. Hence, we conducted a study with the available NCD data under the National Program for Prevention and Control of Cancer, Diabetes, CVD and Stroke (NPCDCS) implemented by the Government of India from all district hospitals in 51 districts Madhya Pradesh. In Madhya Pradesh, NPCDCS program was launched in 2010 in 5 districts and subsequently escalated to all 51 districts by 2015-16. We did a secondary data analysis of existing data obtained from all district hospitals of 51 districts to estimate the distribution of expected cases versus registered DM and HTN in the study population.

**Methods**

We did a secondary data analysis of data obtained through monthly reporting formats of NPCDCS program in all government district hospitals of state Madhya Pradesh. We analyzed data from form '6' of NPCDCS Program from all hospitals from January to December 2017. We calculated the expected number of DM and HTN cases in the study population based on DM and HTN as per AHS 2012-13. We considered district-wise population from census 2011 for all 51 districts extrapolated for 2017 as per the decadal growth rate based on the prevalence of DM and HTN. Data analysis was done using Microsoft Office Excel tool. The cut-off value to identify individuals at NCD clinic with HTN was >=140 mmHg for systolic BP and >=90mmHg for diastolic BP. Similarly, to identify individuals with DM, the cut-off values were, fasting blood sugar >=126 mg/dl and postprandial blood sugar>=200 mg/dl NCD [2]. For the study purpose, the expected number of cases was defined as 20% of the total prevalence of DM and HTN in the respected district, as only 20% of the total with the disorder visited government health facility and registered cases were those who visited government health facility and got diagnosed for DM and HTN [3].

The formula used for calculating the expected number of cases

– Assuming 37% of the population over 30 years.

– Total population x 37 / 100 = N

– N x 22% / 100 = (suspected HTN) = A

– N x 7% / 100 = (suspected DM) = B

– Cases visiting Government Health Facility 19.7% (*)

– A x 19 / 100 = Total HTN Visiting Government Health Facility

– B x 19 / 100 = Total DM Visiting Government Health Facility

**Results**

Based on the data analysis, in Madhya Pradesh, the expected number of hypertension cases visiting government health facilities were 12,83,976 against that of total cases registered in the government health facility, which was only 67,252, which is only 5.2% of the expected number. District Indore shows a maximum number of expected hypertension cases, which was 62,195 in 2017. Similarly, district Agar-malwa shows the minimum number of expected cases with hypertension which was 10,301, by the end of 2017. However, the least number of cases were reported in district Burahanpur, which was 923, the expected number of cases with hypertension in district Burahanpur was13,181 (Figure 1)**.** The expected cases of diabetes mellitus visiting government health facility were 4,08,538 against that of total cases registered in a government health facility which was only 71,420, i.e. only 17.48% of the expected number. Maximum numbers of expected Diabetic cases were again from Indore, which was 19,789 and the minimum number of expected Diabetic cases were in district Agar-Malwa which was 3,192, and district reported 642 (20%) patients registered by the end of the year 2017. The maximum percentage of diagnosed patients out of expected patients were noticed in Tikamgarah 3512 (43%) while the lowest percentage documented in Dhar 545 (4%) (Figure 1).

**Conclusion**

Almost all the cases diagnosed were put on treatment. However, there was a wide variation among districts within the state. The data quality is questionable in some districts as treatment initiation rate was more than 100%, which could be due to individuals who got diagnosed for HTN from private health facility but initiated their treatment from government facility or duplication of data was another possible reason. There was no data on patients' follow-up to check adherence and control among HTN and DM patients. We recommend a flowchart to screen all adults attending a health facility, capacity building of data operator, update recording format to track follow-up cases, education campaigns for population and caregivers to increase hypertension & diabetes awareness.

**References**

1. Annual Health Survey (AHS) 2012-13, Office of registrar general & census commissioner India. https://www.censusindia.gov.in/vital_statistics/AHSBulletins/AHS_Factsheets_2012-13/FACTSHEET-MP.pdf. Accessed 09 February 2021.

2. NPCDCS program operational guidelines by GOI 2013-17. https://main.mohfw.gov.in/sites/default/files/Operational%20Guidelines%20of%20NPCDCS%20%28Revised%20-%202013-17%29_1.pdf. Accessed 09 February 2021.

3. Kujawski SA, Leslie HH, Prabhakaran D, et al. Reasons for low utilization of public facilities among households with hypertension: analysis of a population-based survey in India. BMJ Glob Health2018;3:e001002. doi:10.1136/bmjgh-2018-001002.

**Fig. 1 (abstract A73).**
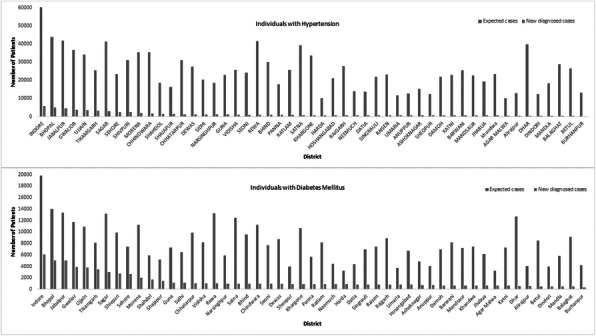
Individuals with hypertension and Diabetes Mellitus: Expected vs Diagnosed

